# Bioinformatics comparisons of RNA-binding proteins of pathogenic and non-pathogenic *Escherichia coli* strains reveal novel virulence factors

**DOI:** 10.1186/s12864-017-4045-3

**Published:** 2017-08-24

**Authors:** Pritha Ghosh, Ramanathan Sowdhamini

**Affiliations:** National Centre for Biological Sciences, Tata Institute of Fundamental Research, Bellary Road, Bangalore, Karnataka 560 065 India

**Keywords:** *Escherichia coli*, RNA-binding proteins, Genome-wide survey, Pathogen, Virulence, Ribonuclease PH, PELOTA, Uncharacterised

## Abstract

**Background:**

Pathogenic bacteria have evolved various strategies to counteract host defences. They are also exposed to environments that are undergoing constant changes. Hence, in order to survive, bacteria must adapt themselves to the changing environmental conditions by performing regulations at the transcriptional and/or post-transcriptional levels. Roles of RNA-binding proteins (RBPs) as virulence factors have been very well studied. Here, we have used a sequence search-based method to compare and contrast the proteomes of 16 pathogenic and three non-pathogenic *E. coli* strains as well as to obtain a global picture of the RBP landscape (RBPome) in *E. coli*.

**Results:**

Our results show that there are no significant differences in the percentage of RBPs encoded by the pathogenic and the non-pathogenic *E. coli* strains. The differences in the types of Pfam domains as well as Pfam RNA-binding domains, encoded by these two classes of *E. coli* strains, are also insignificant. The complete and distinct RBPome of *E. coli* has been established by studying all known *E. coli* strains till date. We have also identified RBPs that are exclusive to pathogenic strains, and most of them can be exploited as drug targets since they appear to be non-homologous to their human host proteins. Many of these pathogen-specific proteins were uncharacterised and their identities could be resolved on the basis of sequence homology searches with known proteins. Detailed structural modelling, molecular dynamics simulations and sequence comparisons have been pursued for selected examples to understand differences in stability and RNA-binding.

**Conclusions:**

The approach used in this paper to cross-compare proteomes of pathogenic and non-pathogenic strains may also be extended to other bacterial or even eukaryotic proteomes to understand interesting differences in their RBPomes. The pathogen-specific RBPs reported in this study, may also be taken up further for clinical trials and/or experimental validations.

**Electronic supplementary material:**

The online version of this article (doi:10.1186/s12864-017-4045-3) contains supplementary material, which is available to authorized users.

## Background

*Escherichia coli* is one of the most abundant, facultative anaerobic gram-negative bacterium of the intestinal microflora and colonises the mucus layer of the colon. The core genomic structure is common among the commensal strains and the various pathogenic *E. coli* strains that cause intestinal and extra-intestinal diseases in humans [1]. In the pathogenic strains, novel genetic islands and small clusters of genes are present in addition to the core genomic framework and provide the bacteria with increased virulence [2–4]. The extracellular intestinal pathogen, enterohemorrhagic *E. coli* (EHEC), which cause diarrhea, hemorrhagic colitis and the haemolytic uremic syndrome, is the most devastating of the pathogenic *E. coli* strains [5, 6].

Pathogenic bacteria have evolved various strategies to counteract host defences. They are also exposed to environments that are undergoing constant changes. Hence, in order to survive, bacteria must adapt themselves to the changing environmental conditions by altering gene expression levels and in turn adjusting protein levels according to the need of the cell. Such regulations may occur at the transcriptional and/or post-transcriptional levels [7].

RNA-binding proteins (RBPs) are a versatile group of proteins that perform a diverse range of functions in the cell and are ‘master regulators’ of co-transcriptional and post-transcriptional gene expression like RNA modification, export, localization, mRNA translation, turnover [8–12] and also aid in the folding of RNA into conformations that are functionally active [13]. In bacteria, many different classes of RBPs interact with small RNAs (sRNA) to form ribonucleoprotein (RNP) complexes that participate in post-transcriptional gene regulation processes [14–23]. In eukaryotes, noncoding RNAs (ncRNAs) are known to be important regulators of gene expression [24–26]. Hence, bacterial RBPs that are capable of inhibiting this class of RNAs, are also capable of disrupting the normal functioning of their host cells, thus acting as virulence factors. Roles of RBPs like the Hfq [27–36], Repressor of secondary metabolites A (RsmA) [36–41] and endoribonuclease YbeY [42] as virulence factors, have also been very well studied.

Here, we describe the employment of mathematical profiles of RBP families to study the RBP repertoire, henceforth referred to as the ‘RBPome’, in *E. coli* strains. The proteomes of 19 *E. coli* strains (16 pathogenic and three non-pathogenic strains) have been studied to compare and contrast the RBPomes of pathogenic and non-pathogenic *E. coli*. More than 40 different kinds of proteins have been found to be present in two or more pathogenic strains, but absent from all the three non-pathogenic ones. Many of these proteins are previously uncharacterised and may be novel virulence factors and probable candidates for further experimental validations.

We have also extended our search method to probe to all available *E. coli* complete proteomes (till the date of the study) for RBPs, and thus obtain a bigger picture of the RBP landscape in all known *E. coli* strains. The search method can also be adapted in future for comparing the RBPomes of other species of bacteria as well. In addition, our work also discusses case studies on a few interesting RBPs. The first of them is an attempt to provide a structural basis for the inactivity of the Ribonuclease PH (RNase PH) protein from *E. coli* strain K12, the second study deals with the structural modelling and characterisation of RNA substrates of an ‘uncharacterised’ protein that is exclusively found in the pathogenic *E. coli* strains, whereas the third one involves the analysis of pathogen-specific Cas6 proteins and comparison with their non-pathogenic counterparts.

## Methods

### Dataset

Protein families were grouped on the basis of either structural homology (structure-centric families) or sequence homology (sequence-centric families). A dataset of 1285 RNA-protein and 14 DNA/RNA hybrid-protein complexes were collected from the Protein Data Bank (PDB) (May 2015) and were split into protein and RNA chains. The RNA-interacting protein chains in this dataset were classified into 182 Structural Classification of Proteins (SCOP) families, 135 clustered families and 127 orphan families (a total of 437 structure-centric families), on the basis of structural homology with each other. Sequence-centric RNA-binding families were retrieved from Pfam, using an initial keyword search of ‘RNA’, followed by manual curation to generate a dataset of 746 families. The structure-centric classification scheme, the generation of structure-centric family Hidden Markov Models (HMMs) and retrieval of sequence-centric family HMMs from the Pfam database (v 28) were as adapted from our previous study [43].

Proteomes of 19 *E. coli* strains were retrieved from UniProt Proteomes (May 2016) [44] for the comparative study of pathogenic and non-pathogenic strains. The names and organism IDs of the *E. coli* strains, their corresponding UniProt proteome IDs and the total number of proteins in each proteome have been listed in Table 1.

**Table 1:**
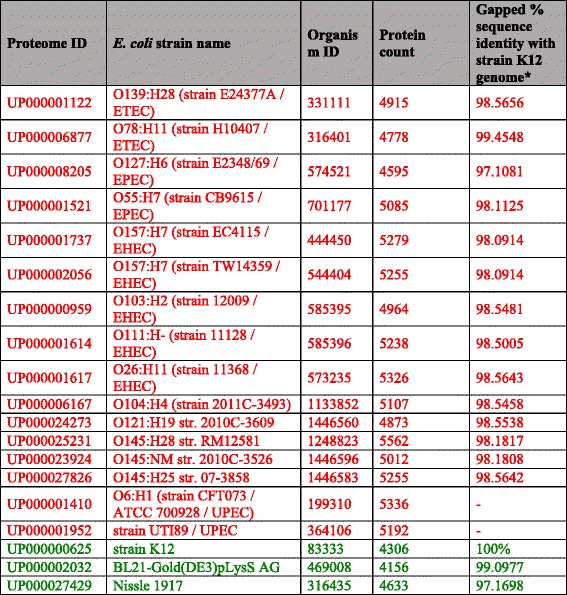
*E. coli* proteomes for comparative study. The 19 *E. coli* proteomes from UniProt (May 2016) used in the study for the comparison of RBPomes of pathogenic and non-pathogenic strains have been listed in this table. The pathogenic and the non-pathogenic *E. coli* strains have been represented in red and green fonts, respectively

^a^points to the differences among the strains at the genome level, considering the strain K12 (or lab strain) as the standard

All complete *E. coli* proteomes were retrieved from RefSeq (May 2016) [45] to study the overall RBP landscape in *E. coli*. The names of the *E. coli* strains, their corresponding assembly IDs and the total number of proteins in each proteome and have been listed in Table 2.

**Table 2:** Complete *E. coli* proteomes. The 166 *E. coli* complete proteomes from RefSeq (May 2016) that have been used in the study have been listed in this table

Organism/Name	Strain	Assembly	Proteins
*E. coli* O157:H7 str. Sakai	Sakai substr. RIMD 0509952	GCA_000008865.1	5292
*E. coli* IAI39	IAI39	GCA_000026345.1	4725
*E. coli* str. K-12 substr. MG1655	K-12 substr. MG1655	GCA_000005845.2	4140
*E. coli* O83:H1 str. NRG 857C	NRG 857C	GCA_000183345.1	4582
*E. coli* O104:H4 str. 2011C-3493	2011C-3493	GCA_000299455.1	5149
*E. coli* CFT073	CFT073	GCA_000007445.1	4897
*E. coli* BL21(DE3)	BL21(DE3)	GCA_000009565.2	4302
*E. coli* str. K-12 substr. W3110	K-12 substr. W3110	GCA_000010245.1	4410
*E. coli* SE11	SE11	GCA_000010385.1	4968
*E. coli* SE15	SE15	GCA_000010485.1	4573
*E. coli* O103:H2 str. 12,009	12,009	GCA_000010745.1	5423
*E. coli* O111:H- str. 11,128	11,128	GCA_000010765.1	5673
*E. coli* UTI89	UTI89	GCA_000013265.1	4963
*E. coli* 536	536	GCA_000013305.1	4542
*E. coli* APEC O1	APEC O1	GCA_000014845.1	5292
*E. coli* O139:H28 str. E24377A	E24377A	GCA_000017745.1	5021
*E. coli* HS	HS	GCA_000017765.1	4366
*E. coli* B str. REL606	REL606	GCA_000017985.1	4344
*E. coli* ATCC 8739	ATCC 8739	GCA_000019385.1	4434
*E. coli* str. K-12 substr. DH10B	K-12 substr. DH10B	GCA_000019425.1	4450
*E. coli* SMS-3-5	SMS-3-5	GCA_000019645.1	4908
*E. coli* O157:H7 str. EC4115	EC4115	GCA_000021125.1	5631
*E. coli* O157:H7 str. TW14359	TW14359	GCA_000022225.1	5537
*E. coli* BW2952	K-12 substr. BW2952	GCA_000022345.1	4347
*E. coli* BL21(DE3)	BL21(DE3)	GCA_000022665.2	4302
*E. coli* DH1	DH1	GCA_000023365.1	4369
*E. coli* ‘BL21-Gold(DE3)pLysS AG’	BL21-Gold(DE3)pLysS AG	GCA_000023665.1	4322
*E. coli* O55:H7 str. CB9615	CB9615	GCA_000025165.1	5262
*E. coli* IHE3034	IHE3034	GCA_000025745.1	4911
*E. coli* 55,989	55,989	GCA_000026245.1	4953
*E. coli* IAI1	IAI1	GCA_000026265.1	4450
*E. coli* S88	S88	GCA_000026285.1	4696
*E. coli* O127:H6 str. E2348/69	E2348/69	GCA_000026545.1	4924
*E. coli* 042	42	GCA_000027125.1	5131
*E. coli* O26:H11 str. 11,368	11,368	GCA_000091005.1	5833
*E. coli* KO11FL	KO11	GCA_000147855.3	4850
*E. coli* ABU 83972	ABU 83972	GCA_000148365.1	4862
*E. coli* UM146	UM146	GCA_000148605.1	4779
*E. coli* W	W	GCA_000184185.1	4825
*E. coli* ETEC H10407	ETEC H10407	GCA_000210475.1	5124
*E. coli* UMNK88	UMNK88	GCA_000212715.2	5542
*E. coli* NA114	NA114	GCA_000214765.2	4720
*E. coli* PCN033	PCN033	GCA_000219515.3	4881
*E. coli* UMNF18	UMNF18	GCA_000220005.2	5521
*E. coli* O7:K1 str. CE10	CE10	GCA_000227625.1	5152
*E. coli* str. ‘clone D i2’	clone D i2	GCA_000233875.1	4740
*E. coli* str. ‘clone D i14’	clone D i14	GCA_000233895.1	4742
*E. coli* O55:H7 str. RM12579	RM12579	GCA_000245515.1	5213
*E. coli* P12b	P12b	GCA_000257275.1	4549
*E. coli* KO11FL	KO11FL	GCA_000258025.1	4732
*E. coli* W	W	GCA_000258145.1	4831
*E. coli* Xuzhou21	Xuzhou21	GCA_000262125.1	5402
*E. coli* str. K-12 substr. MG1655	K-12 substr. MG1655	GCA_000269645.2	4405
*E. coli* DH1	DH1	GCA_000270105.1	4351
*E. coli* str. K-12 substr. MG1655	K-12 substr. MG1655	GCA_000273425.1	4404
*E. coli* LF82	LF82	GCA_000284495.1	4544
*E. coli* O25b:H4-ST131	EC958	GCA_000285655.3	5037
*E. coli* O104:H4 str. 2009EL-2050	2009EL-2050	GCA_000299255.1	5283
*E. coli* O104:H4 str. 2009EL-2071	2009EL-2071	GCA_000299475.1	5227
*E. coli* APEC O78	APEC O78	GCA_000332755.1	4598
*E. coli* str. K-12 substr. MDS42	K-12 substr. MDS42	GCA_000350185.1	3713
*E. coli* LY180	LY180	GCA_000468515.1	4586
*E. coli* PMV-1	PMV-1	GCA_000493595.1	5100
*E. coli* JJ1886	JJ1886	GCA_000493755.1	5151
*E. coli* str. K-12 substr. MC4100	K-12 substr. MC4100	GCA_000499485.1	4284
*E. coli* O145:H28 str. RM13514	RM13514	GCA_000520035.1	5524
*E. coli* O145:H28 str. RM13516	RM13516	GCA_000520055.1	5354
*E. coli*	ST540	GCA_000597845.1	4498
*E. coli*	ST540	GCA_000599625.1	4532
*E. coli*	ST540	GCA_000599645.1	4550
*E. coli*	ST2747	GCA_000599665.1	4665
*E. coli*	ST2747	GCA_000599685.1	4585
*E. coli*	ST2747	GCA_000599705.1	4547
*E. coli* O145:H28 str. RM12761	RM12761	GCA_000662395.1	5349
*E. coli* O145:H28 str. RM12581	RM12581	GCA_000671295.1	5520
*E. coli* Nissle 1917	Nissle 1917	GCA_000714595.1	4990
*E. coli* KLY	KLY	GCA_000725305.1	4478
*E. coli* O157:H7 str. SS17	SS17	GCA_000730345.1	5532
*E. coli* O157:H7 str. EDL933	EDL933	GCA_000732965.1	5530
*E. coli* ATCC 25922	ATCC 25922	GCA_000743255.1	4940
*E. coli* BW25113	K-12 substr. BW25113	GCA_000750555.1	4398
*E. coli*	ECONIH1	GCA_000784925.1	5320
*E. coli* ER2796	ER2796	GCA_000800215.1	4311
*E. coli* K-12	ER3413	GCA_000800765.1	4309
*E. coli* RS218	RS218	GCA_000800845.2	4791
*E. coli*	RM9387	GCA_000801165.1	4775
*E. coli*	94–3024	GCA_000801185.2	4792
*E. coli* str. K-12 substr. MG1655	K-12 substr. MG1655	GCA_000801205.1	4387
*E. coli* O157:H7 str. SS52	SS52	GCA_000803705.1	5489
*E. coli* APEC IMT5155	APEC IMT5155	GCA_000813165.1	4840
*E. coli*	6409	GCA_000814145.2	4893
*E. coli*		GCA_000819645.1	4996
*E. coli* O157:H16	Santai	GCA_000827105.1	4776
*E. coli* 1303	1303	GCA_000829985.1	4849
*E. coli*	C41(DE3)	GCA_000830035.1	4302
*E. coli* ECC-1470	ECC-1470	GCA_000831565.1	4673
*E. coli*	BL21 (TaKaRa)	GCA_000833145.1	4262
*E. coli*	MNCRE44	GCA_000931565.1	5137
*E. coli*	K-12 substr. RV308	GCA_000952955.1	4342
*E. coli*	K-12 substr. HMS174	GCA_000953515.1	4344
*E. coli*	HUSEC2011	GCA_000967155.1	5294
*E. coli* VR50	VR50	GCA_000968515.1	4968
*E. coli*	CI5	GCA_000971615.1	4874
*E. coli* K-12	ER3454	GCA_000974405.1	4375
*E. coli* K-12	ER3440	GCA_000974465.1	4367
*E. coli* K-12	ER3476	GCA_000974505.1	4354
*E. coli* K-12	ER3445	GCA_000974535.1	4360
*E. coli* K-12	ER3466	GCA_000974575.1	4415
*E. coli* K-12	ER3446	GCA_000974825.1	4357
*E. coli* K-12	ER3475	GCA_000974865.1	4359
*E. coli* K-12	ER3435	GCA_000974885.1	4443
*E. coli* K-12	K-12 substr. AG100	GCA_000981485.1	4394
*E. coli* O104:H4 str. C227–11	C227–11	GCA_000986765.1	5269
*E. coli*	SEC470	GCA_000987875.1	4941
*E. coli*	SQ37	GCA_000988355.1	4405
*E. coli*	SQ88	GCA_000988385.1	4403
*E. coli*	SQ2203	GCA_000988465.1	4402
*E. coli*	CFSAN029787	GCA_001007915.1	5090
*E. coli* K-12	K-12 substr. GM4792	GCA_001020945.2	4362
*E. coli* K-12	K-12 substr. GM4792	GCA_001021005.2	4368
*E. coli* PCN061	PCN061	GCA_001029125.1	4680
*E. coli*	C43(DE3)	GCA_001039415.1	4254
*E. coli*	NCM3722	GCA_001043215.1	4530
*E. coli* ACN001	ACN001	GCA_001051135.1	4671
*E. coli*	DH1Ec095	GCA_001183645.1	4345
*E. coli*	DH1Ec104	GCA_001183665.1	4342
*E. coli*	DH1Ec169	GCA_001183685.1	4342
*E. coli*	RR1	GCA_001276585.1	4337
*E. coli*	SF-088	GCA_001280325.1	5019
*E. coli*	SF-468	GCA_001280345.1	5218
*E. coli*	SF-166	GCA_001280385.1	4773
*E. coli*	SF-173	GCA_001280405.1	4936
*E. coli* O157:H7	WS4202	GCA_001307215.1	5294
*E. coli* str. K-12 substr. MG1655	K-12 substr. MG1655	GCA_001308065.1	4398
*E. coli*	K-12 substr. MG1655_TMP32XR1	GCA_001308125.1	4398
*E. coli*	K-12 substr. MG1655_TMP32XR2	GCA_001308165.1	4399
*E. coli*	2012C-4227	GCA_001420935.1	5142
*E. coli*	2009C-3133	GCA_001420955.1	5311
*E. coli*	YD786	GCA_001442495.1	4604
*E. coli*	CQSW20	GCA_001455385.1	4142
*E. coli*	uk_P46212	GCA_001469815.1	5023
*E. coli*	ST648	GCA_001485455.1	4838
*E. coli*	CD306	GCA_001513615.1	5021
*E. coli*	JJ2434	GCA_001513635.1	5099
*E. coli*	ACN002	GCA_001515725.1	4618
*E. coli*	MRE600	GCA_001542675.2	4603
*E. coli* str. K-12 substr. MG1655	K-12 substr. MG1655	GCA_001544635.1	4407
*E. coli*	JEONG-1266	GCA_001558995.1	5358
*E. coli* B	C2566	GCA_001559615.1	4209
*E. coli* B	C3029	GCA_001559635.1	4317
*E. coli* K-12	DHB4	GCA_001559655.1	4522
*E. coli* K-12	C3026	GCA_001559675.1	4731
*E. coli* str. K-12 substr. MG1655	JW5437–1 substr. MG1655	GCA_001566335.1	4405
*E. coli*	SaT040	GCA_001566615.1	4963
*E. coli*	G749	GCA_001566635.1	4983
*E. coli*	ZH193	GCA_001566675.1	5040
*E. coli*	ZH063	GCA_001577325.1	4984
*E. coli* JJ1887	JJ1887	GCA_001593565.1	5142
*E. coli* str. Sanji	Sanji	GCA_001610755.1	5172
*E. coli*	28RC1	GCA_001612475.1	5504
*E. coli*	SRCC 1675	GCA_001612495.1	5511
*E. coli*	Ecol_732	GCA_001617565.1	5243
*E. coli*	Ecol_743	GCA_001618325.1	4866
*E. coli*	Ecol_745	GCA_001618345.1	4803
*E. coli*	Ecol_448	GCA_001618365.1	4956
*E. coli* B7A	B7A	GCA_000725265.1	5384

### Search method

**Fig. 1:**
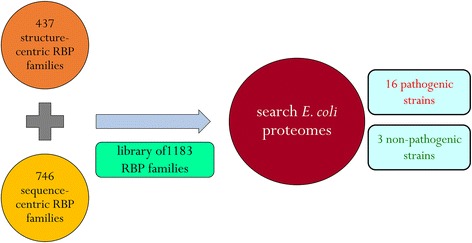
Search scheme for the genome-wide survey. A schematic representation of the search method for the GWS has been represented in this figure. Starting from 437 structure-centric and 746 sequence-centric RBP families, a library of 1183 RBP family HMMs were built. These mathematical profiles were then used to search proteomes of 19 different *E. coli* strains (16 pathogenic and three non-pathogenic strains). It is to be noted here that the same search scheme has been used later to extend the study to all 166 available *E. coli* proteomes in the RefSeq database as of May 2016 (see text for further details)

The search method was described in our previous study [43] and is represented schematically in Fig. 1. A library of 1183 RBP family HMMs (437 structure-centric families and 746 sequence-centric families) were used as start points to survey the *E. coli* proteomes for the presence of putative RBPs. The genome-wide survey (GWS) for each *E. coli* proteome was performed with a sequence E-value cut-off of 10^−3^ and the hits were filtered with a domain i-Evalue cut-off of 0.5. i-Evalue (independent E-value) is the E-value that the sequence/profile comparison would have received if this were the only domain envelope found in it, excluding any others. This is a stringent measure of how reliable this particular domain may be. The independent E-value uses the total number of targets in the target database. We have now mentioned this definition in the revised manuscript. The Pfam (v 28) domain architectures (DAs) were also resolved at the same sequence E-value and domain i-Evalue cut-offs.

### Comparison of RNA-binding proteins across strains

The RBPs identified from 19 different strains of *E. coli*, were compared by performing all-against-all protein sequence homology searches using the BLASTP module of the NCBI BLAST 2.2.30 + suite [46] with a sequence E-value cut-off of 10^−5^. The hits were clustered on the basis of 30% sequence identity and 70% query coverage cut-offs to identify *similar* proteins i.e., proteins that had a sequence identity of greater than or equal to 30%, as well as a query coverage of greater than or equal to 70%, were considered to homologous in terms of sequence and hence clustered. These parameters were standardised on the basis of previous work from our lab to identify true positive sequence homologues [47].

Associations for proteins that were annotated as ‘hypothetical’ or ‘uncharacterised’, were obtained by sequence homology searches against the NCBI non-redundant (NR) protein database (February 2016) with a sequence E-value cut-off of 10^−5^. The BLASTP hits were also clustered on the basis of 100% sequence identity, 100% query coverage and equal length cut-offs to identify *identical* proteins.

Clusters that consist of proteins from two or more of the pathogenic strains, but not from any of the non-pathogenic ones, will henceforth be referred as ‘pathogen-specific clusters’ and the proteins in such clusters as ‘pathogen-specific proteins’. Sequence homology searches were performed for these proteins against the reference human proteome (UP000005640) retrieved from Swiss-Prot (June 2016) [44] at a sequence E-value cut-off of 10^−5^. The hits were filtered on the basis of 30 percentage sequence identity and 70 percentage query coverage cut-offs.

### Modelling and dynamics studies of RNase PH protein

The structures of the active and inactive monomers of the tRNA processing enzyme Ribonuclease PH (RNase PH) from strains O26:H11 (UniProt ID: C8TLI5) and K12 (UniProt ID: P0CG19), respectively, were modelled on the basis of the RNase PH protein from *Pseudomonas aeruginosa* (PDB code: 1R6M: A) (239 amino acids) using the molecular modelling program MODELLER v 9.15 [48]. The active and inactive RNase PH monomers are 238 and 228 amino acids in length, respectively and are 69% and 70% identical to the template, respectively. Twenty models were generated for each of the active and inactive RNase PH monomers and validated using PROCHECK [49], VERIFY3D [50], ProSA [51] and HARMONY [52]. The best model for each of the active and inactive RNase PH monomers were selected on the basis of Discrete Optimized Protein Energy (DOPE) score and other validation parameters obtained from the above-mentioned programs. The best models for the active and inactive RNase PH monomers were subjected to 100 iterations of the Powell energy minimisation method in the Tripos Force Field (in absence of any electrostatics) using SYBYL7.2 (Tripos Inc.). These were subjected to 100 ns (ns) molecular dynamics (MD) simulations (three replicates each) in the AMBER99SB protein, nucleic AMBER94 force field [53] using the Groningen Machine for Chemical Simulations (GROMACS 4.5.5) program [54].

The biological assembly (hexamer) of RNase PH from *Pseudomonas aeruginosa* (PDB code: 1R6M) served as the template and was obtained using the online tool (PISA) (http://www.ebi.ac.uk/pdbe/prot_int/pistart.html) [55]. The structures of the active and inactive hexamers of RNase PH from strains O26:H11 and K12, respectively were modelled and the 20 models generated for each of the active and inactive RNase PH hexamers were validated using the same set of tools, as mentioned above. The best models were selected and subjected to energy minimisations, as described above. Electrostatic potential on the solvent accessible surfaces of the proteins were calculated using PDB2PQR [56] (in the AMBER force field) and Adaptive Poisson-Boltzmann Solver (APBS) [57]. The head-to-head dimers were randomly selected from both the active and the inactive hexamers of the protein for performing MD simulations, to save computational time. Various energy components of the dimer interface were measured using the in-house algorithm, PPCheck [58]. This algorithm identifies interface residues in protein-protein interactions on the basis of simple distance criteria, following which the strength of interactions at the interface are quantified. 100 ns MD simulations (three replicates each) were performed with the same set of parameters as mentioned above for the monomeric proteins.

### Modelling and dynamics studies of an ‘uncharacterised’ pathogen-specific protein

The structure of the PELOTA_1 domain (Pfam ID: PF15608) of an ‘uncharacterised’ pathogen-specific protein from strain O103:H2 (UniProt ID: C8TX32) (371 amino acids) was modelled on the basis of the L7Ae protein from *Methanocaldococcus jannaschii* (PDB code: 1XBI: A) (117 amino acids) and validated, as described earlier. The 64 amino acids long PELOTA_1 domain of the uncharacterised protein, has 36% sequence identity with the corresponding 75 amino acids domain of the template. The best model was selected as described in the case study on RNase PH. This model was subjected to 100 iterations of the Powell energy minimisation method in the Tripos Force Field (in absence of any electrostatics) using SYBYL7.2 (Tripos Inc.). Structural alignment of the modelled PELOTA_1 domain and the L7Ae K-turn binding domain from *Archaeoglobus fulgidus* (PDB code: 4BW0: B) was performed using Multiple Alignment with Translations and Twists (Matt) [59]. The same kink-turn RNA from *H. marismortui*, found in complex with the L7Ae K-turn binding domain from *A. fulgidus,* was docked onto the model, guided by the equivalents of the RNA-interacting residues (at a 5 Å cut-off distance from the protein) in the *A. fulgidus* L7Ae protein (highlighted in yellow in the upper panel of Fig. 7c) using the molecular docking program HADDOCK [60]. The model and the L7Ae protein from *A. fulgidus*, in complex with kink-turn RNA from *H. marismortui*, were subjected to 100 ns MD simulations (three replicates each) in the AMBER99SB protein, nucleic AMBER94 force field using the GROMACS 4.5.5 program.

### Sequence analysis of pathogen-specific Cas6-like proteins

The sequences of all the proteins in Cluster 308 were aligned to the Cas6 protein sequence in *E. coli* strain K12 (UniProt ID: Q46897), using MUSCLE [61] and subjected to molecular phylogeny analysis using the Maximum Likelihood (ML) method and a bootstrap value of 1000 in MEGA7 (CC) [62, 63]. All reviewed CRISPR-associated Cas6 protein sequences were also retrieved from Swiss-Prot (March 2017) [44], followed by manual curation to retain 18 Cas6 proteins. Sequences of two uncharacterised proteins (UniProt IDs: C8U9I8 and C8TG04) from Cluster 308, known to be homologous to known CRISPR-associated Cas6 proteins (on the basis of sequence homology searches against the NR database, as described earlier) were aligned to those of the 18 reviewed Cas6 proteins using MUSCLE. The sequences were then subjected to molecular phylogeny analysis using the above-mentioned parameters. Secondary structure predictions for all the proteins were performed using PSIPRED [64].

The structures of Cas6 proteins from *E. coli* strain K12 (PDB codes: 4QYZ: K, 5H9E: K and 5H9F: K) were retrieved from the PDB. The RNA-binding and protein-interacting residues in the Cas6 protein structures were calculated on the basis of 5 Å and 8 Å distance cut-off criteria, from the associated crRNAs (PDB codes: 4QYZ: L, 5H9E: L and 5H9F: L, respectively) and the protein chains (PDB codes: 4QYZ: A-J, 5H9E: A-J and 5H9F: A-J, respectively), respectively.

## Results

### Genome-wide survey (GWS) of RNA-binding proteins in pathogenic and non-pathogenic *E. coli* strains

**Fig. 2:**
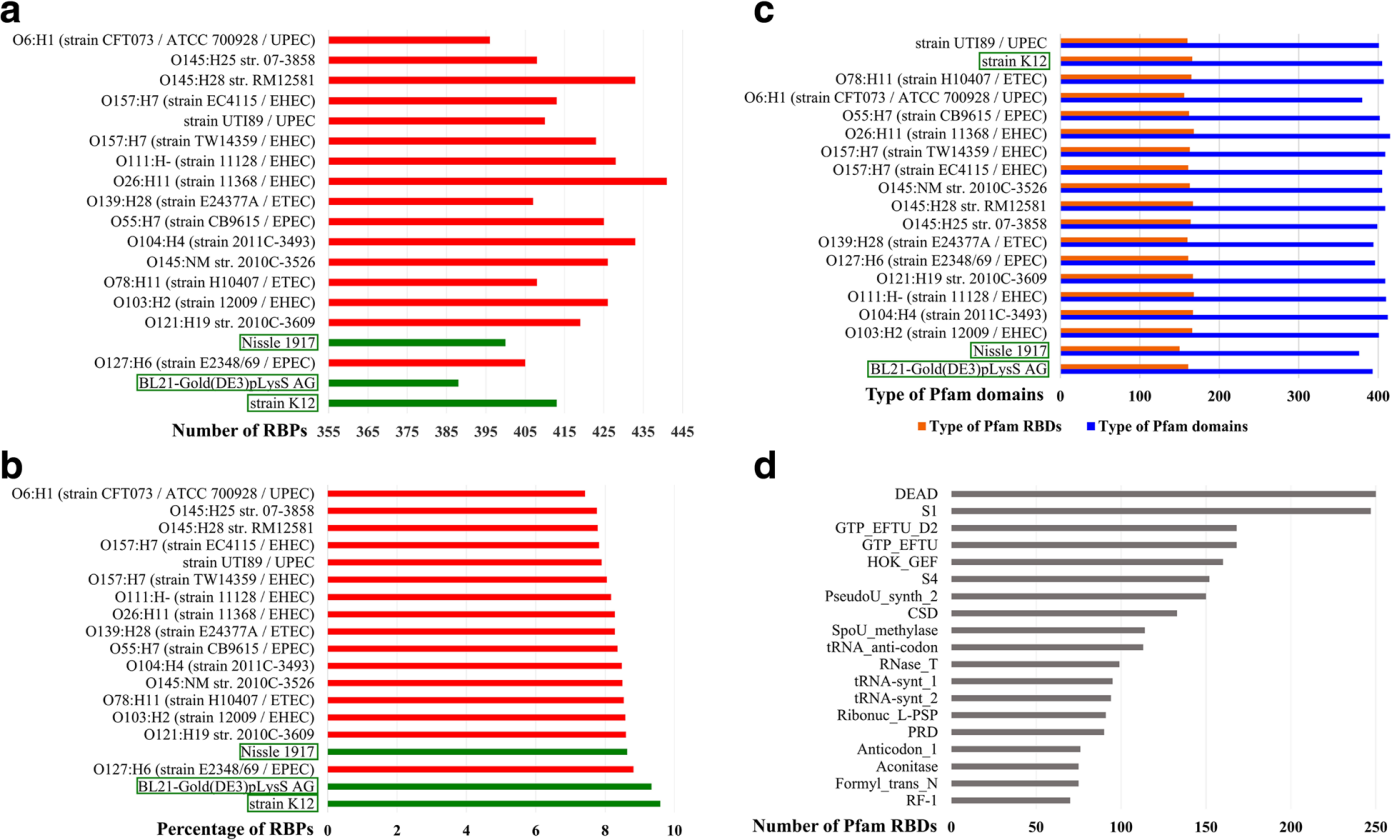
Statistics for the genome-wide survey of 19 *E. coli* strains. The different statistics obtained from the GWS have been represented in this figure. In panels **a** and **b**, the pathogenic strains have been represented in red and the non-pathogenic ones in green. The non-pathogenic strains have also been highlighted with green boxes. **a**. The number of RBPs in each strain. The pathogenic O26:H11 strain encodes the highest number of RBPs in its proteome. **b**. The percentage of RBPs in the proteome of each strain. These percentages have been calculated with respect to the proteome size of the strain under consideration. The difference in this number among the pathogenic and the non-pathogenic strains are insignificant (Welch Two Sample t-test: *t* = 3.2384, df = 2.474, *p*-value = 0.06272). **c**. The type of Pfam domains encoded by each strain. The difference in the types of Pfam domains, as well as Pfam RBDs, encoded by the pathogenic and the non-pathogenic strains are insignificant (Welch Two Sample t-test for types of Pfam domains: *t* = −1.3876, df = 2.263, *p*-value = 0.2861; Welch Two Sample t-test for types of Pfam RBDs: *t* = −0.9625, df = 2.138, *p*-value = 0.4317). **d**. The abundance of Pfam RBDs. 185 types of Pfam RBDs were found to be encoded in the RBPs, of which DEAD domains have the highest representation (approximately 4% of all Pfam RBDs)

The GWS of RBPs was performed in 19 different *E. coli* strains (16 pathogenic and three non-pathogenic strains) and a total of 7902 proteins were identified (Additional file 1: Table S1). Figure 2a shows the number of RBPs found in each of the strains studied here. The pathogenic strains have a larger RBPome, as compared to the non-pathogenic ones - with strain O26:H11 encoding the greatest (441). The pathogenic strains also have bigger proteome sizes (in terms of the number of proteins in the proteome), as compared to their non-pathogenic counterparts, by virtue of maintaining plasmids in them. Hence, to normalise for proteome size, the number of RBPs in each of these strains were expressed as a function of their respective number of proteins in the proteome (Fig. 2b). We observed that the difference in the percentage of RBPs in the proteome among the pathogenic and the non-pathogenic strains are insignificant (Welch Two Sample t-test: *t* = 3.2384, df = 2.474, *p*-value = 0.06272).

To compare the differential abundance of domains, if any, among the pathogens and the non-pathogens, the Pfam DAs of all the RBPs were resolved (to strengthen the results in this section, this study has been extended to all known *E. coli* proteomes and will be discussed in a later section). The number of different types of Pfam domains and that of Pfam RNA-binding domains (RBDs) found in each strain have been represented in Fig. 2c. We observed that the difference in the types of Pfam domains, as well as Pfam RBDs, encoded by the pathogenic and the non-pathogenic strains are insignificant (Welch Two Sample t-test for types of Pfam domains: *t* = − 1.3876, df = 2.263, *p*-value = 0.2861; Welch Two Sample t-test for types of Pfam RBDs: *t* = − 0.9625, df = 2.138, *p*-value = 0.4317). The number of different Pfam RBDs, found across all the 19 *E. coli* strains studied here, has been shown in Fig. 2d and also been listed in Table 3.

**Table 3:** Pfam RNA-binding domains. The Pfam RBDs and their corresponding occurrences in the GWS of 19 *E. coli* strains have been listed in this table. The Pfam domains listed are on the basis of Pfam database (v.28)

Pfam domain	Number of occurrences	Pfam domain	Number of occurrences
DEAD	250	MnmE_helical	19
S1	247	RNA_pol_Rpb2_7	19
GTP_EFTU	168	tRNA-synt_2e	19
GTP_EFTU_D2	168	Ribosomal_L11_N	19
HOK_GEF	160	KilA-N	19
S4	152	Ribosomal_S4	19
PseudoU_synth_2	150	RTC	19
CSD	133	Ribosomal_L2	19
SpoU_methylase	114	DnaB_bind	19
tRNA_anti-codon	113	IPPT	19
RNase_T	99	IF3_C	19
tRNA-synt_1	95	UPF0020	19
tRNA-synt_2	94	RNA_pol_A_bac	19
Ribonuc_L-PSP	91	RNA_pol_Rpb1_3	19
PRD	90	Se-cys_synth_N	19
Anticodon_1	76	RimM	19
Formyl_trans_N	75	Val_tRNA-synt_C	19
Aconitase	75	TruB_C_2	19
RF-1	70	RNA_pol_Rpb1_4	19
tRNA-synt_1c	58	dsrm	19
RNase_PH	57	RNA_pol_Rpb2_1	19
RNase_PH_C	57	Rho_N	19
Dus	57	CheR	19
HGTP_anticodon	57	SHS2_FTSA	19
tRNA-synt_2b	57	Helicase_RecD	19
tRNA_bind	56	RNA_pol_Rpb6	19
Sua5_yciO_yrdC	56	RNA_pol_Rpb2_6	19
tRNA_U5-meth_tr	56	SpoU_methylas_C	19
zf-FPG_IleRS	55	Trm112p	19
Ldr_toxin	52	RNA_pol_Rpb1_1	19
RtcB	50	CsrA	19
CorA	46	Ribosomal_S20p	19
MazE_antitoxin	44	TruB-C_2	19
ProQ	41	DALR_2	19
DbpA	39	IF-2	19
HA2	39	tRNA_Me_trans	19
PolyA_pol_RNAbd	38	KH_1	19
TruD	38	ABC1	19
IF2_N	38	tRNA-synt_1_2	19
PseudoU_synth_1	38	PUA	19
Methyltr_RsmF_N	38	CAT_RBD	19
SpoU_sub_bind	38	PNPase	19
SgrR_N	38	tRNA_m1G_MT	19
tRNA_edit	38	SelB-wing_2	19
PolyA_pol	38	RNase_H	19
FtsJ	38	PRC	19
HRDC	38	Ribosomal_L18p	19
tRNA-synt_1b	38	GIDA_assoc	19
THUMP	38	RrnaAD	19
DALR_1	38	YjeF_N	19
NusB	38	LigT_PEase	19
RNase_E_G	38	Ub-RnfH	19
ASCH	37	Nol1_Nop2_Fmu_2	19
DNA_pol_A_exo1	37	RRF	19
tRNA_SAD	36	B5	18
DHHA1	36	FDX-ACB	18
GTP_EFTU_D3	35	GidB	18
MqsA_antitoxin	27	Sigma70_r1_1	18
TGT	21	IF3_N	18
RNA_pol_Rpb1_2	20	B3_4	18
Queuosine_synth	20	tRNA-synt_2c	17
RVT_1	20	Colicin-DNase	16
tRNA_synt_2f	19	PTS_2-RNA	16
SelB-wing_3	19	CRISPR_Cse1	14
Methyltrans_RNA	19	CRISPR_Cse2	14
Ribosomal_L11	19	FinO_N	13
CRS1_YhbY	19	PIN	13
Tyr_Deacylase	19	GIIM	11
Ribosomal_L4	19	IlvGEDA_leader	11
MutS_II	19	SymE_toxin	11
RNA_pol_Rpb1_5	19	PRTase_1	10
TilS	19	PELOTA_1	10
Endonuclease_1	19	DNA_primase_S	10
Ribosomal_L25p	19	IlvB_leader	9
Sigma54_CBD	19	YafO_toxin	8
RapA_C	19	MT-A70	8
TilS_C	19	RPAP2_Rtr1	6
OB_NTP_bind	19	TisB_toxin	5
GlutR_dimer	19	MqsR_toxin	5
RNA_pol_Rpb2_3	19	Ibs_toxin	5
RtcR	19	RNA_ligase	4
TruB_N	19	N36	3
RsmJ	19	Cloacin	3
tRNA_bind_2	19	Colicin_D	2
tRNA-synt_1c_C	19	Viral_helicase1	2
RNA_pol_Rpb2_45	19	IF-2B	1
HisG	19	Colicin_immun	1
Rho_RNA_bind	19	RPOL_N	1
PNPase_C	19	RNA_pol	1
RNase_HII	19	RnlA_toxin	1
RNA_pol_A_CTD	19	NYN	1
RNA_pol_L	19	DUF3850	1
Rsd_AlgQ	19		

We found that *E. coli* encodes 185 different types of Pfam RBDs in their proteomes and the DEAD domain was found to be the most abundant, constituting approximately 4% of the total number of Pfam RBD domains in *E. coli*. The DEAD box family of proteins are RNA helicases that are required for RNA metabolism and thus are important players in gene expression [65]. These proteins use ATP to unwind short RNA duplexes in an unusual fashion and also help in the remodelling of RNA-protein complexes.

### Comparison of RNA-binding proteins across strains reveals novel pathogen-specific factors

**Fig. 3:**
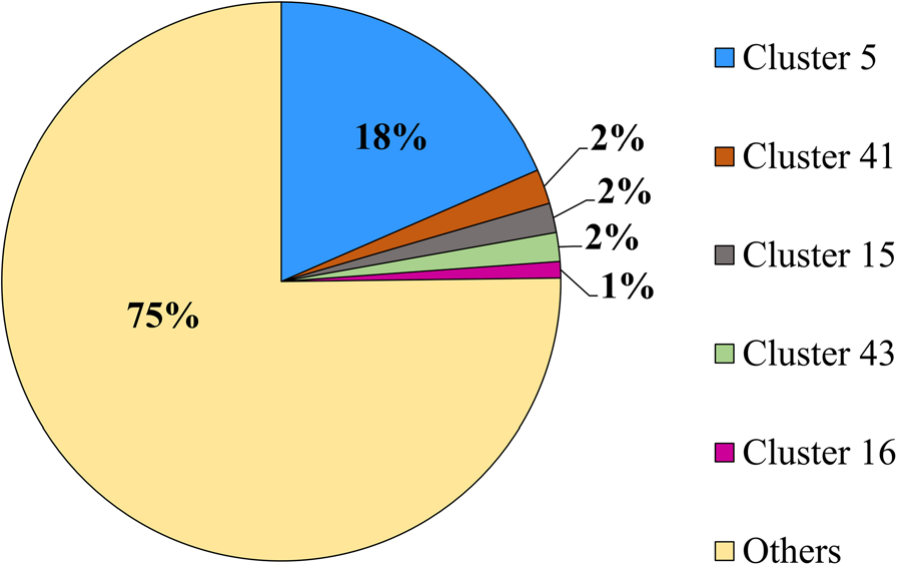
Clusters of RNA-binding proteins. The percentage of RBPs in the different clusters has been represented in this figure. The RBPs obtained from each of the 19 *E. coli* strains (16 pathogenic and three non-pathogenic strains) have been clustered on the basis of homology searches (*see text for further details*). Five of the biggest clusters and their identities are as follows: Cluster 5 (ATP-binding subunit of transporters), Cluster 41 (Small toxic polypeptides), Cluster 15 (RNA helicases), Cluster 43 (Cold shock proteins) and Cluster 16 (Pseudouridine synthases)

The proteins were clustered on the basis of sequence homology searches in order to compare and contrast the RBPs across the *E. coli* strains studied here. The 7902 proteins identified from all the strains were grouped into 384 clusters, on the basis of sequence homology with other members of the cluster (Additional file 2: Table S2). Greater than 99% of the proteins could cluster with one or more RBPs and formed 336 multi-member clusters (MMCs), whereas the rest of the proteins failed to cluster with other RBPs and formed 48 single-member clusters (SMCs). The distribution of members among all the 384 clusters has been depicted in Fig. 3.

The largest of the MMCs, consists of 1459 RBPs which are ATP-binding subunit of transporters. The *E. coli* genome sequence had revealed that the largest family of paralogous proteins were composed of ATP-binding cassette (ABC) transporters [66]. The ATP-binding subunit of ABC transporters share common features with other nucleotide-binding proteins [67] like, the *E. coli* RecA [68] and the F1-ATPase from bovine heart [69]. GCN20, YEF3 and RLI1 are examples of soluble ABC proteins that interact with ribosomes and regulate translation and ribosome biogenesis [70–72].

The other large MMCs were those of small toxic polypeptides that are components of the bacterial toxin-antitoxin (TA) systems [73–77], RNA helicases that are involved in various aspects of RNA metabolism [78, 79] and Pseudouridine synthases that are enzymes responsible for pseudouridylation, which is the most abundant post-transcriptional modification in RNAs [80]. Cold shock proteins bind mRNAs and regulate translation, rate of mRNA degradation etc. [81, 82]. These proteins are induced during the response of the bacterial cell towards temperature rise.

The majority of the SMCs (38 out of 48 SMCs) are RBPs from pathogenic strains and lack homologues in any of the other strains considered here. These include proteins like putative helicases, serine proteases, and various endonucleases. Likewise, members of the small toxic Ibs protein family (IbsA, IbsB, IbsC, IbsD and IbsE that form Clusters 362, 363, 364, 365 and 366 respectively) from strain K12 are noteworthy examples of SMCs that are in non-pathogenic strains only. These Ibs proteins cause the cessation of growth when overexpressed [83].

### Pathogen-specific proteins

In this study, the 226 pathogen-specific proteins that formed 43 pathogen-specific clusters are of special interest. Sixty-three of these proteins were previously uncharacterised and associations for all of these proteins were obtained on the basis of sequence homology searches against the NCBI-NR database. The function annotation of each of these clusters were transferred on the basis of homology. The biological functions and the number of RBPs constituting these pathogen-specific clusters have been listed in Table 4.

**Table 4:** Pathogen-specific RNA-binding protein clusters. The size of RBP clusters with members from only the pathogenic *E. coli* strains in our GWS of 19 *E. coli* strains have been listed in this table

Cluster number	Number of members	Cluster name
Cluster 283	13	KilA-N domain phage proteins
Cluster 299	13	tRNA(fMet)-specific VapC endonucleases
Cluster 176	10	DEAD/DEAH box helicases
Cluster 307	10	CRISPR type I-E/−associated proteins CasA/Cse1
Cluster 308	10	CRISPR-associated proteins Cas6/Cse3/CasE, subtype I-E
Cluster 309	10	CRISPR type I-E/−associated proteins CasB/Cse2
Cluster 310	10	CRISPR-associated proteins Cas5/CasD, subtype I-E
Cluster 60	10	Putative ATP/GTP-binding proteins
Cluster 318	9	ASCH domain-containing proteins
Cluster 122	8	Adenine DNA methyltransferases, phage-associated
Cluster 305	8	Post-segregational killing toxins
Cluster 116	7	RNA-binding proteins
Cluster 298	6	VagC, VagC-homologs
Cluster 317	6	Type I restriction-modification enzymes, M subunits
Cluster 319	6	Type I restriction-modification enzymes, R subunits
Cluster 125	5	Helicases
Cluster 246	5	DNA-binding proteins
Cluster 287	5	ATP-dependent helicases
Cluster 288	5	DEAD/DEAH box helicases
Cluster 290	5	DEAD/DEAH box helicases
Cluster 63	5	UvrD/REP helicase-like proteins
Cluster 98^a^	5	Protein kinases
Cluster 161	4	2′-5′ RNA ligases
Cluster 300	4	proQ/FINO family proteins
Cluster 313	4	ATP-dependent helicases, res subunit of Type III restriction enzyme, SNF2 family helicases
Cluster 329	4	Serine/Threonine kinases
Cluster 301	3	YajA proteins
Cluster 323	3	Putative pyridoxal phosphate-dependent enzymes, Putative transferases
Cluster 324	3	Sigma-54 dependent transcription regulators, Putative transcriptional regulators of NtrC family
Cluster 326	3	Leucin-rich repeat proteins
Cluster 330	3	Ankyrin repeat-containing domain proteins
Cluster 172	2	Zn-dependent hydrolases, including glyoxylases, Beta-lactamases
Cluster 238	2	Hypothetical proteins
Cluster 302	2	Peptidyl-arginine deiminases
Cluster 303	2	Exonucleases
Cluster 315	2	KilA-N domain phage proteins
Cluster 320	2	Pyocin, putative colicin activity proteins
Cluster 325	2	Hyothetical proteins
Cluster 327	2	KilA-N domain proteins
Cluster 332	2	Hypothetical phage associated proteins
Cluster 334	2	Hypothetical proteins
Cluster 335	2	Chromosome partitioning ParA proteins
Cluster 336	2	Serine/Threonine kinases

^a^All the proteins in this cluster have sequence homologues in humans

If these pathogen-specific proteins are exclusive to the pathogenic strains, then they may be exploited for drug design purposes. To test this hypothesis, we surveyed the human (host) proteome for the presence of sequence homologues of these proteins. It was found that, barring the protein kinases that were members of Cluster 98 (marked in asterisk in Table 4), none of the pathogen-specific proteins were homologous to any human protein within the thresholds employed in the search strategy (please see *Methods* section for details). Few of the pathogen-specific protein clusters are described in the following section.

The DEAD/DEAH box helicases that use ATP to unwind short duplex RNA [65], formed three different clusters. In two of the clusters, the DEAD domains (Pfam ID: PF00270) were associated with C-terminal Helicase_C (Pfam ID: PF00271) and DUF1998 (Pfam ID: PF09369) domains. On the other hand, in a bigger cluster, the DEAD/DEAH box helicases were composed of DNA_primase_S (Pfam ID: PF01896), ResIII (Pfam ID: PF04851) and Helicase_C domains. Four of the pathogen-specific clusters were those of Clustered Regularly Interspaced Short Palindromic Repeat (CRISPR) sequence-associated proteins, consisting of RBPs from 10 pathogenic strains each. Recent literature reports also support the role of CRISPR-associated proteins as virulence factors in pathogenic bacteria [84]. The KilA-N domains are found in a wide range of proteins and may share a common fold with the nucleic acid-binding modules of certain nucleases and the N-terminal domain of the tRNA endonuclease [85]. Fertility inhibition (FinO) protein and the anti-sense FinP RNA are members of the FinOP fertility inhibition complex which regulates the expression of the genes in the transfer operon [86–89]. tRNA (fMet)-specific endonucleases are the toxic components of a TA system. This site-specific tRNA-(fMet) endonuclease acts as a virulence factor by cleaving both charged and uncharged tRNA-(fMet) and inhibiting translation. The Activating Signal Cointergrator-1 homology (ASCH) domain is also a putative RBD due to the presence of an RNA-binding cleft associated with a conserved sequence motif characteristic of the ASC-1 superfamily [90].

### Identification of the distinct RNA-binding protein repertoire in *E. coli*

We identified identical RBPs across *E. coli* strains, on the basis of sequence homology searches and other filtering criteria (as mentioned in the *Methods* section). Out of the 7902 RBPs identified in our GWS, 6236 had one or more identical partners from one or more strains and formed 1227 clusters, whereas 1666 proteins had no identical counterparts. Hence, our study identified 2893 RBPs from 19 *E. coli* strains that were distinct from each other. Identification of such a distinct pool of RBPs will help to provide an insight to the possible range of functions performed by this class of proteins in *E. coli*, and hence compare and contrast with the possible functions performed by RBPs in other organisms.

### GWS of RNA-binding proteins in all known *E. coli* strains

We extended the above-mentioned study, by performing GWS of RBPs in 166 complete *E. coli* proteomes available in the RefSeq database (May 2016) and a total of 8464 proteins were identified (Additional file 3). It should be noted that, unlike the nomenclature system of UniProt, where the same protein occurring in different strains are denoted with different UniProt accession IDs, RefSeq assigns same or at times different accession IDs to the same protein occurring in different strains. Thus, on the basis of unique accession IDs, 8464 RBPs were identified. The 8464 RBPs were grouped into 401 clusters on the basis of sequence homology with other members of the cluster. We found that greater than 99% of the proteins could cluster with one or more RBPs and formed 339 MMCs, whereas the rest of the proteins failed to cluster with other RBPs and formed 62 SMCs.

**Fig. 4:**
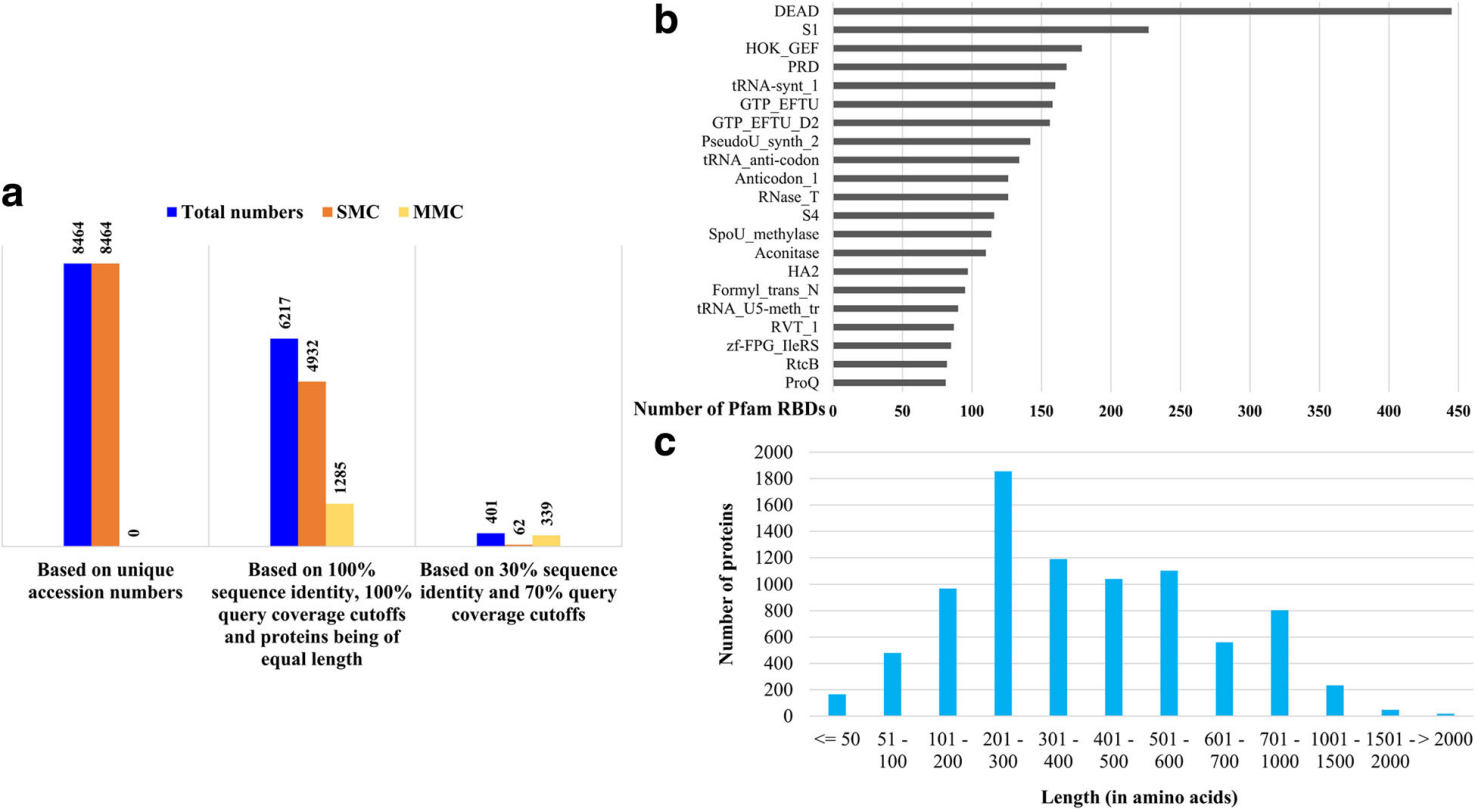
Statistics for the genome-wide survey of 166 *E. coli* strains. The different statistics obtained from the GWS have been represented in this figure. **a** The number of RBPs as determined by different methods (*see text for further details*). **b** The abundance of Pfam RBDs. 188 types of Pfam RBDs were found to be encoded in the RBPs, of which DEAD domains have the highest representation (approximately 6% of all Pfam RBDs). **c** The length distribution of RBPs

The above-mentioned GWS statistics for RBP numbers have been plotted in Fig. 4a. The number of different Pfam RBDs found across all complete *E. coli* proteomes has been shown in Fig. 4b. Similar to the afore-mentioned results, seen from the dataset of 19 *E. coli* proteomes, it was found that *E. coli* encodes 188 different types of Pfam RBDs in their proteomes and the DEAD domain was still observed to be the most abundant, constituting approximately 6% of the total number of Pfam RBD domains in *E. coli*. The length distribution of RBPs from *E. coli* have been plotted in Fig. 4c and RBPs of the length 201–300 amino acids were found to be the most prevalent.

### Identification of the complete distinct RBPome in 166 proteomes of *E. coli*

These 8464 RBPs (please see previous section) formed 1285 clusters of two or more identical proteins, accounting for 3532 RBPs, whereas the remaining 4932 RBPs were distinct from the others. Hence, 6217 RBPs, distinct from each other, were identified from all known *E. coli* strains, which is much greater than the number (2893) found from 19 *E. coli* proteomes.

It should be noted that the pathogenicity annotations are not very clear for few of the 166 *E. coli* strains for which complete proteome information are available. Hence, we have performed the analysis for the pathogen-specific proteins using the smaller dataset of 19 proteomes, whereas all the 166 complete proteomes have been considered for the analysis for the complete *E. coli* RBPome.

### Case studies

Three case studies on interesting RBPs were performed to answer some outstanding questions and have been described in the following sections. The first of the three examples, deals with a RNase PH protein that does not cluster with those from any of the other 165 *E. coli* proteomes considered in this study. This protein, which forms a SMC, is interesting in the biological context due to its difference with the other RNase PH proteins, both at the level of sequence as well as biological activity. The second case study deals with a protein that is a part of a pathogen-specific cluster, in which none of the proteins are well-annotated. This protein was found to encode a bacterial homologue of a well-known archaeo-eukaryotic RBD, whose RNA-binding properties are not as well studied as its homologues. The final study involves a sequence-based approach to analyse the pathogen-specific CRISPR-associated Cas6 proteins, and compare the same with similar proteins from the non-pathogenic strains.

### Case study 1: RNase PH from strain K12 is inactive due to a possible loss of stability of the protein

**Fig. 5:**
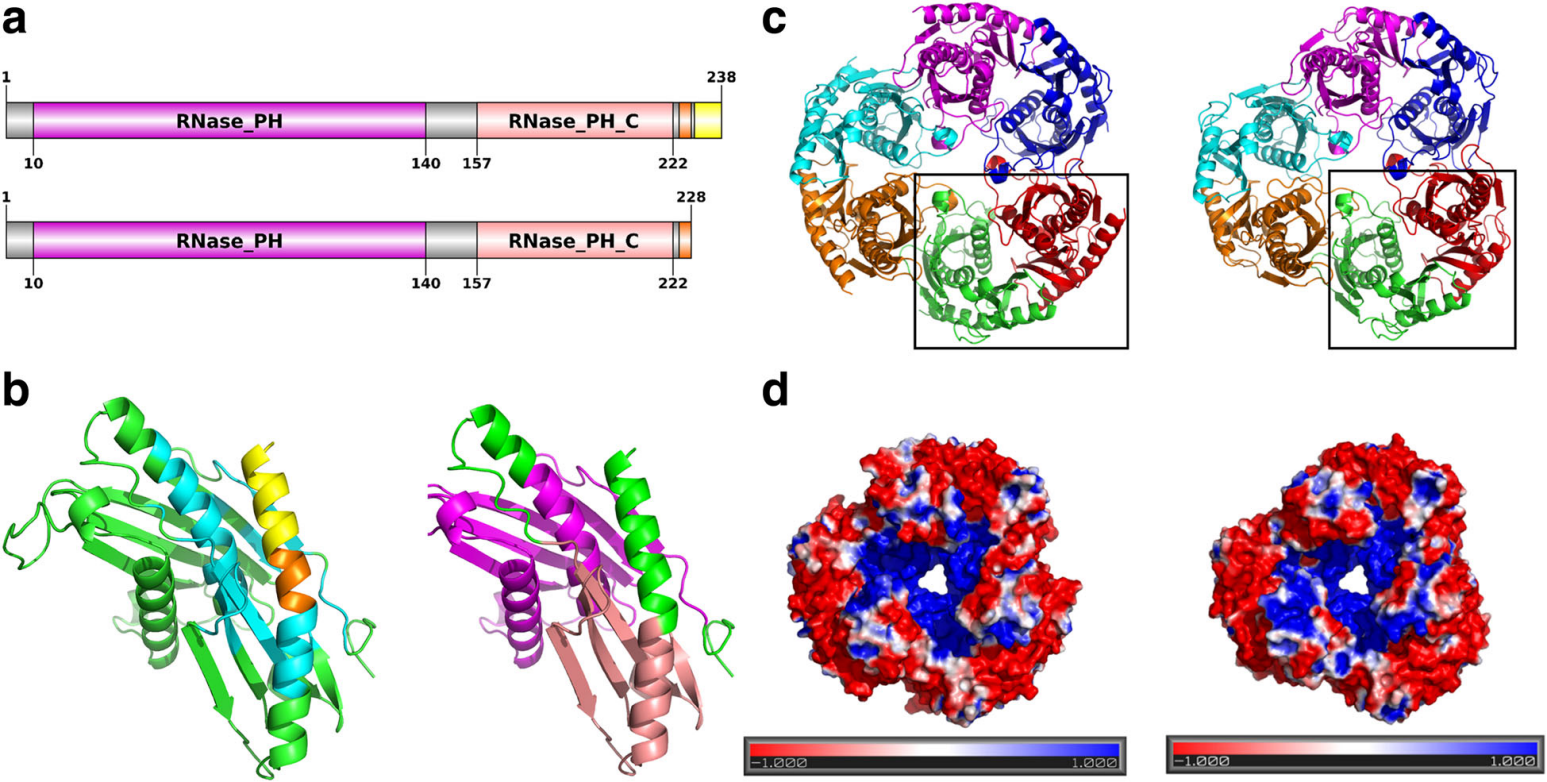
Modelling of the RNase PH proteins from two different *E. coli* strains. The structural modelling of the RNase PH protein has been represented in this figure. **a** Schematic diagram of the active (*above*) and the inactive (*below*) RNase PH proteins. The RNase PH and the RNase_PH_C domains, as defined by Pfam (v.28), have been represented in *magenta* and *pink*, respectively. The five residues that have undergone mutations due to a point deletion and the ten residues that are missing from the inactive RNase PH protein from strain K12 have been depicted in *orange* and *yellow*, respectively. These two sets of residues are the ones of interest in this study. **b** Model of the RNase PH monomer from strain O26:H11. The residues with the same colour codes as mentioned in panel (**a**), have been represented on the structure of the model. The residues that are within an 8 Å cut-off distance from the residues of interest have been highlighted in *cyan* (*left*). **c** Structure of the RNase PH hexamer from strain O26:H11 (*left*) and the probable structure of the inactive RNase PH hexamer from strain K12 (*right*). The dimers marked in *black boxes* are the ones that were randomly selected for MD simulations. **d** Electrostatic potential on the solvent accessible surface of the RNase PH hexamer from strain O26:H11 (*left*) and that of the inactive RNase PH hexamer from strain K12 (*right*)

RNase PH is a phosphorolytic exoribonuclease involved in the maturation of the 3′-end of transfer RNAs (tRNAs) containing the CCA motif [91–93]. The RNase PH protein from strain K12 was found to be distinct from all other known RNase PH proteins from *E. coli* and has a truncated C-terminus. In 1993, DNA sequencing studies had revealed that a GC base pair (bp) was missing in this strain from a block of five GC bps found 43–47 upstream of the *rph* stop codon [94]. This one base pair deletion leads to a translation frame shift over the last 15 codons, resulting in a premature stop codon (five codons after the deletion). This premature stop codon, in turn, leads to the observed reduction in size of the RNase PH protein by 10 residues. It was also shown by Jensen [94] that this protein lacks RNase PH activity. Figure 5a shows a schematic representation of the DAs of the active (up) and inactive (down) RNase PH proteins, with the five residues that have undergone mutations and the ten residues that are missing from the inactive RNase PH protein depicted in orange and yellow, respectively. These are the residues of interest in our study. The same colour coding has been used both in Fig. 5a and b.

To provide a structural basis for this possible loss of activity of the RNase PH protein from strain K12, we modelled the structures of the RNase PH protein monomer as well as the hexamer from strains O26:H11 and K12 (Fig. 5b and c). It is known in the literature that the hexamer (trimer of dimers) is the biological unit of the RNase PH protein and that the hexameric assembly is mandatory for the activity of the protein [95, 96].

**Fig. 6:**
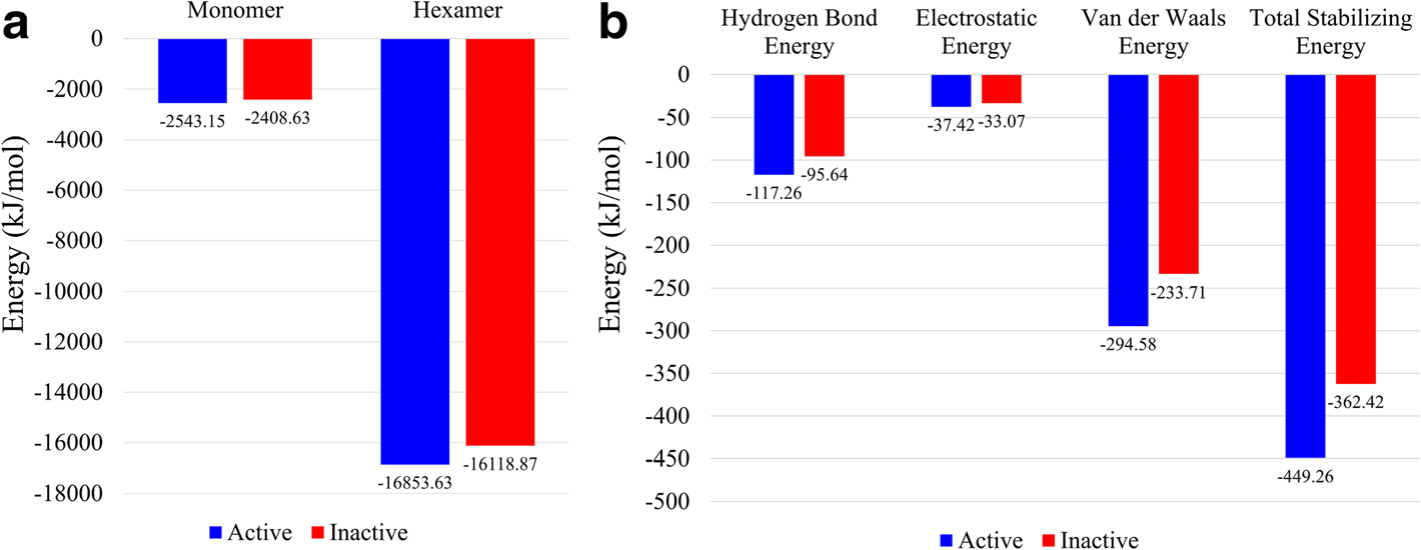
Energy values for the active and inactive RNase PH monomers, dimers and hexamers. The energy values (in kJ/mol) for the active (*blue*) and the inactive (*red*) RNase PH proteins, as calculated by SYBYL (in panel **a**) and PPCheck (in panel **b**) have been plotted in this figure. **a** The energy values for the active and the inactive RNase PH monomers and hexamers. The results show that both the monomeric, as well as the hexameric forms of the inactive RNase PH protein, is unstable as compared to the those of the active RNase PH protein. **b** The interface energy values for the active and the inactive RNase PH dimers (as marked in *black boxes* in Fig. 5c). The results show that the dimer interface of the inactive RNase PH protein is less stabilised as compared to that of the active RNase PH protein

The stability of both the monomer and the hexamer were found to be affected in strain K12, as compared to that in strain O26:H11. The energy values have been plotted in Fig. 6a. In both monomer and hexamer, there is a reduction in stability, suggesting that the absence of C-terminal residues affects the stability of the protein, perhaps more than a cumulative contribution to the stability of the protein. It should be noted that since the monomeric form of the inactive protein is less stable than that of its active counterpart, the hexameric assembly of the inactive RNase PH protein is only a putative one. Hence, the putative and/or unstable hexameric assembly of the RNase PH protein, leads to the loss of activity of the protein.

Figure 5b shows that the residues marked in cyan (left) are at an interacting distance of 8 Å from the residues of interest (left). These residues marked in cyan are a subset of the RNase PH domain, which is marked in magenta (right). Hence, the loss of possible interactions (between the residues marked in cyan and the residues of interest) and subsequently stability of the three-dimensional structure of the RNase PH domain might explain the inactive nature of the protein from strain K12. Figure 5d shows differences in the electrostatic potential on the solvent accessible surfaces of the active (left) and inactive (right) RNase PH proteins.

To test this hypothesis for the possible loss of function of the RNase PH protein due to loss of stability of the monomer and/or the hexamer, we performed MD simulations to understand distortions, if any, of the monomer and a randomly selected head-to-head dimer (from the hexameric assembly) of both the active and the inactive proteins. The dimers have been marked in black boxes in Fig. 5c. Various energy components of the dimer interface, as calculated by PPCheck, have been plotted in Fig. 6b. The results show that the inactive RNase PH dimer interface is less stabilised as compared to that of the active protein. The trajectories of the MD runs have been shown in additional movie files (Additional file 4, Additional file 5, Additional file 6 and Additional file 7, for the active monomer, inactive monomer, active dimer and inactive dimer, respectively). Analyses of Additional file 4, and Additional file 5 shows a slight distortion in the short helix (pink) in the absence of residues of interest (orange and yellow), which might lead to overall loss of stability of the monomer. Further analyses (Additional file 6 and Additional file 7) show the floppy nature of the terminal part of the helices that are interacting in the dimer. This is probably due to the loss of the residues of interest, which have been seen to be structured and less floppy in the active RNase PH dimer (Additional file 6).

For each of the systems, the H-bond traces for three replicates (represented in different colours) have been depicted. From these figures, we can observe that the replicates are showing similar H-bonding patterns. Analyses of the number of hydrogen bonds (H-bonds) formed in the system over each picosecond of the MD simulations of the active monomer, inactive monomer, active dimer and inactive dimer have been represented in Fig. 8a, b, c and d, respectively. Comparison of panels a and b of this figure shows a greater number of H-bonds being formed in the active monomer, as compared to that of the inactive monomer, over the entire time period of the simulation. Similarly, comparison of panels c and d of this figure shows a greater number of H-bonds being formed in the active dimer as compared to that of the inactive dimer, over the entire time period of the simulation. These losses of H-bonding interactions might lead to overall loss of stability of the dimer and subsequently that of the hexamer.

### Case study 2: Uncharacterised pathogen-specific protein and its homologues show subtly different RNA-binding properties

**Fig. 7:**
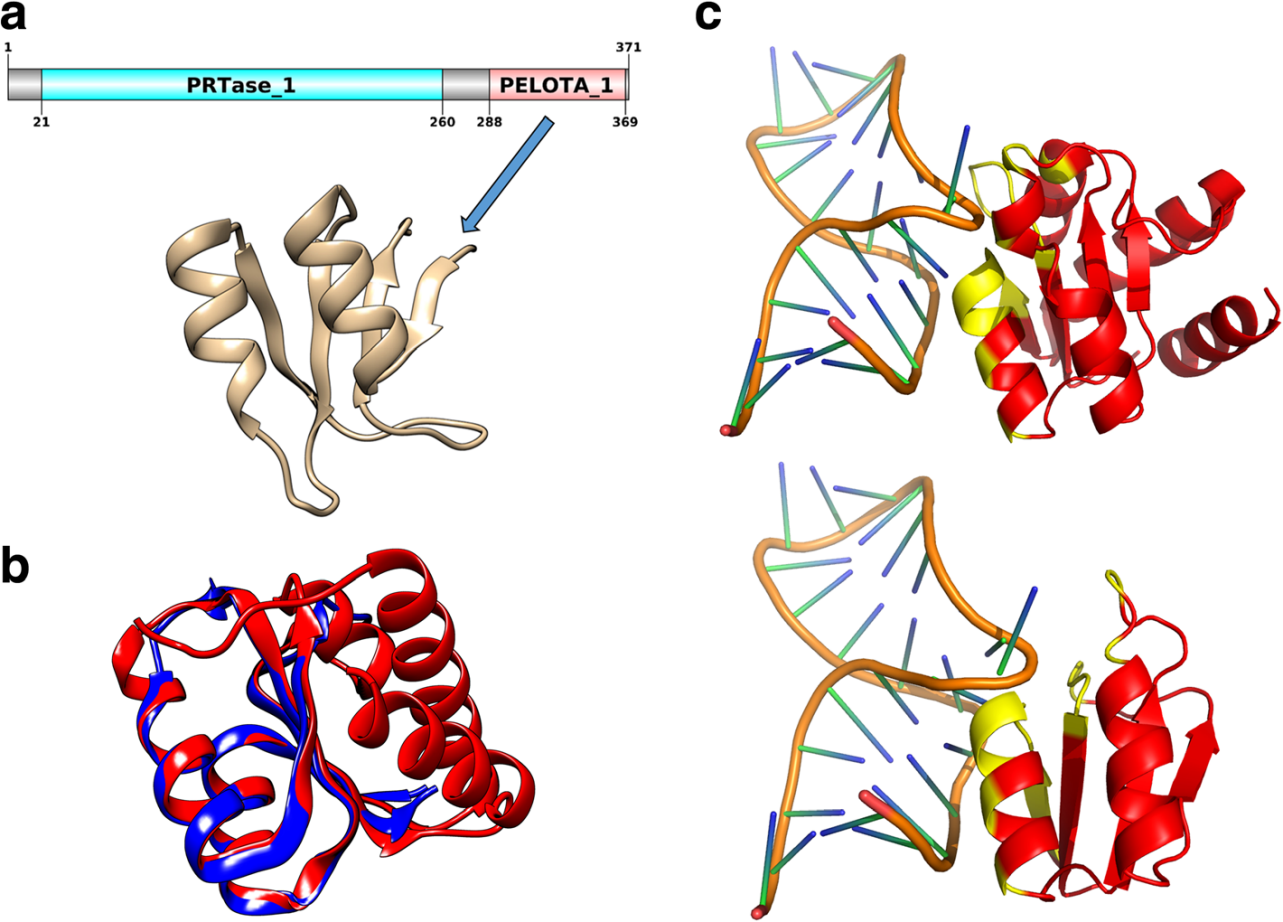
Uncharacterised pathogen-specific RNA-binding protein. The characterisation of the uncharacterised pathogen-specific RBP has been represented in this figure. **a** Schematic representation of the domain architecture of the protein. The RNA-binding PELOTA_1 domain and its model has been shown here. **b** Structural superposition of the L7Ae K-turn binding domain (PDB code: 4BW0: B) (in *red*) and the model of the uncharacterised protein PELOTA_1 domain (in *blue*). c. Comparison of the kink-turn RNA-bound forms of the L7Ae K-turn binding domain (PDB code: 4BW0: B) (*up*) and that of the model of the uncharacterised protein PELOTA_1 domain (*down*). The RNA-binding residues have been highlighted in *yellow*

In our study, we observed that Cluster 60 was composed of 10 proteins, each from a different pathogenic strain studied here. All the proteins in this cluster were either annotated as ‘putative’, ‘uncharacterised’, ‘hypothetical’ or ‘predicted’. To understand the RNA-binding properties of these orthologous pathogen-specific proteins, we resolved the Pfam DA of this protein. In particular, such an association to Pfam domains provide function annotation to a hitherto uncharacterised protein, from strain O103:H2, to RBD PELOTA_1. Hence, the structure of the RNA-binding PELOTA_1 domain of this protein was modelled on the basis of the L7Ae protein from *M. jannaschii* (Fig. 7a).

Domains that are involved in core processes, such as RNA maturation, e.g. the tRNA endonucleases, and translation and with an archaeo-eukaryotic phyletic pattern includes the PIWI, PELOTA and SUI1 domains [97]. In 2014, Anantharaman and co-workers had shown associations of the conserved C-terminus of a phosphoribosyltransferase (PRTase) in the Tellurium resistance (Ter) operon to a PELOTA or Ribosomal_L7Ae domain (Pfam ID: PF01248) [98]. These domains are homologues of the eukaryotic release factor 1 (eRF1), which is involved in translation termination. Unlike the well-studied PELOTA domain, the species distribution of the PELOTA_1 domain is solely bacterial and not much is known in literature regarding the specific function of this domain.

Structure of this modelled PELOTA_1 domain from the uncharacterised protein was aligned with that of the L7Ae kink-turn (K-turn) binding domain from an archaeon (*A. fulgidus*) (Fig. 7b). The model also retained the same basic structural unit as the eRF1 protein (data not shown). The L7Ae is a member of a family of proteins that binds K-turns in many functional RNA species [99]. The K-turn RNA was docked onto the model, guided by the equivalents of the known RNA-interacting residues from the archaeal L7Ae K-turning binding domain. Both the complexes have been shown in Fig. 7c with the RNA-interacting residues highlighted in yellow. MD simulations of both these complexes were performed and the trajectories have been shown in additional movie files Additional file 8 (PELOTA_1 domain model-k-turn RNA complex) and Additional file 9 (L7Ae K-turn binding domain-k-turn RNA complex).

**Fig. 8:**
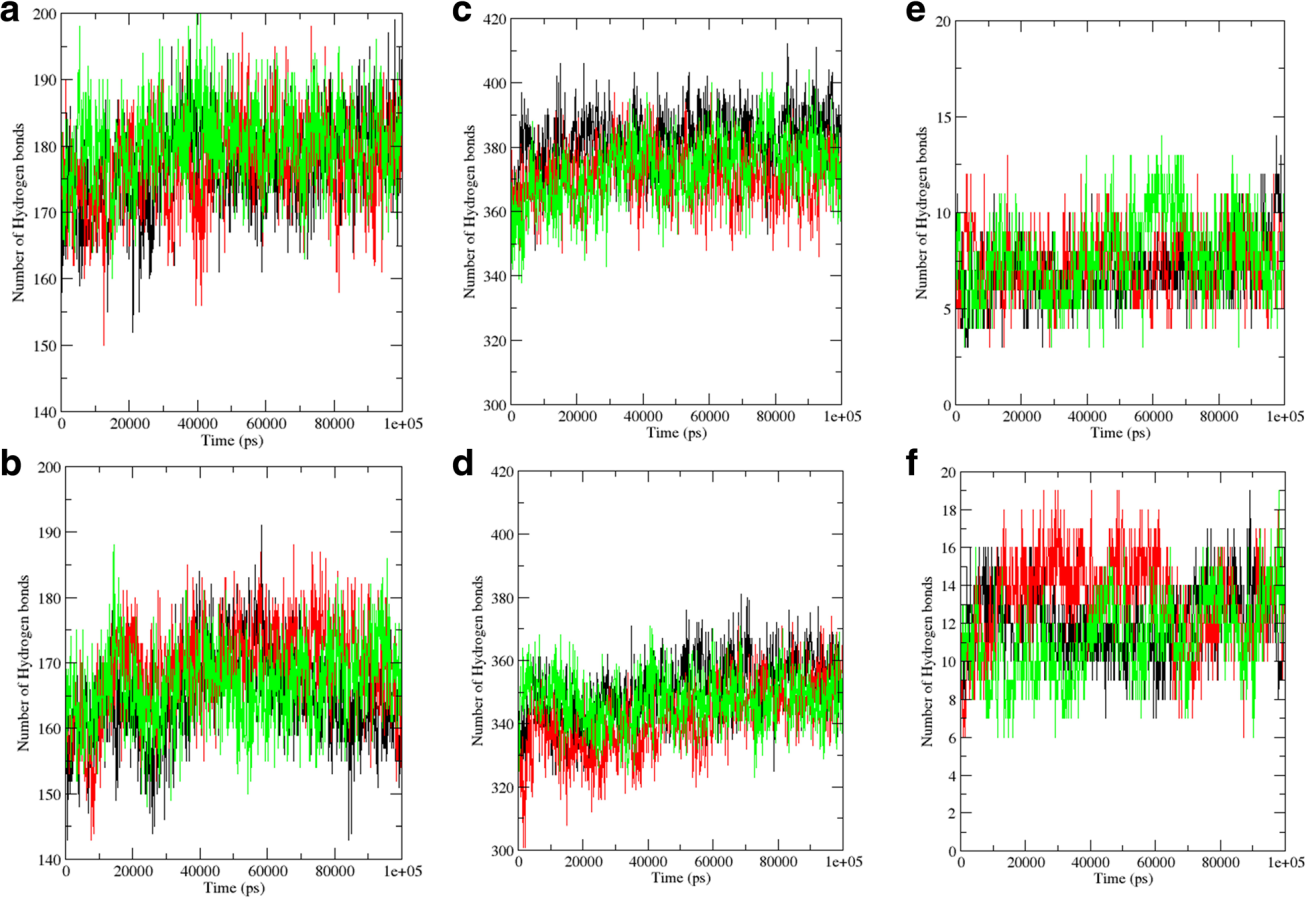
Hydrogen bonding patterns in molecular dynamics simulations. The number of H-bonds formed over each picosecond of the MD simulations (described in this Chapter) have been shown in this figure. Each of the six panels (systems) shows the H-bond traces from three replicates (represented in different colours). **a** Active RNase PH monomer. **b** Inactive RNase PH monomer. **c** Active RNase PH dimer. **d** Inactive RNase PH dimer. **e** PELOTA_1 domain from the ‘uncharacterised’ protein in complex with kink-turn RNA. **f** L7Ae K-turn binding domain from *A. fulgidus* in complex with kink-turn RNA from *H. marismortui*

For each of the systems, the H-bond traces for three replicates (represented in different colours) have been depicted. From these figures, one can observe that the replicates are showing similar H-bonding patterns. Analyses of the number of H-bonds formed between the protein and the RNA over each picosecond of the MD simulations of the PELOTA_1 domain-RNA complex and the L7Ae K-turn binding domain-RNA complex have been represented in Fig. 8e and f, respectively. Comparison of panels e and f of this figure shows a greater number of H-bonds being formed in the L7Ae K-turn binding domain-RNA complex as compared to that of the PELOTA_1 domain-RNA complex over the entire time period of the simulation. These results show that the two proteins have differential affinity towards the same RNA molecule. This hints at the fact that these proteins might perform subtly different functions by the virtue of having differential RNA-binding properties.

### Case study 3: Pathogen specific Cas6-like proteins might be functional variants of the well-characterised non-pathogenic protein

In many bacteria, as well archaea, CRISPR associated Cas proteins and short CRISPR-derived RNA (crRNA) assemble into large RNP complexes and provide surveillance towards invasion of genetic parasites [100–102]. The role of CRISPR-associated proteins as virulence factors in pathogenic bacteria has also been reported in recent literature [84]. We found that Cluster 308 consists of 10 pathogen-specific proteins, of which half of them were already annotated as Cas6 proteins, whereas the other half constituted of ‘uncharacterised’ or ‘hypothetical’ proteins. As mentioned in the *Methods* section, the latter proteins were annotated on the basis of sequence homology to known proteins in the NR database, as Cas6 proteins.

Molecular phylogeny analysis of all the proteins from Cluster 308 and Cas6 from *E. coli* strain K12 has been depicted in Additional file 10a: Figure S1, which reinstates the fact that the pathogen-specific proteins are more similar to each other, in terms of sequence, than they are to the Cas6 protein from the non-pathogenic strain K12. Furthermore, a similar analysis of two previously uncharacterised proteins (UniProt IDs: C8U9I8 and C8TG04) (red) from this pathogen-specific Cas6 proteins cluster (Cluster 308), with other known Cas6 proteins has been shown Additional file 10b: Figure S1. From the phylogenetic tree, one can infer that the pathogen-specific Cas6 proteins are more similar in terms of sequence to the Cas6 from *E. coli* strain K12 (blue) than that from other organisms.

**Fig. 9:**
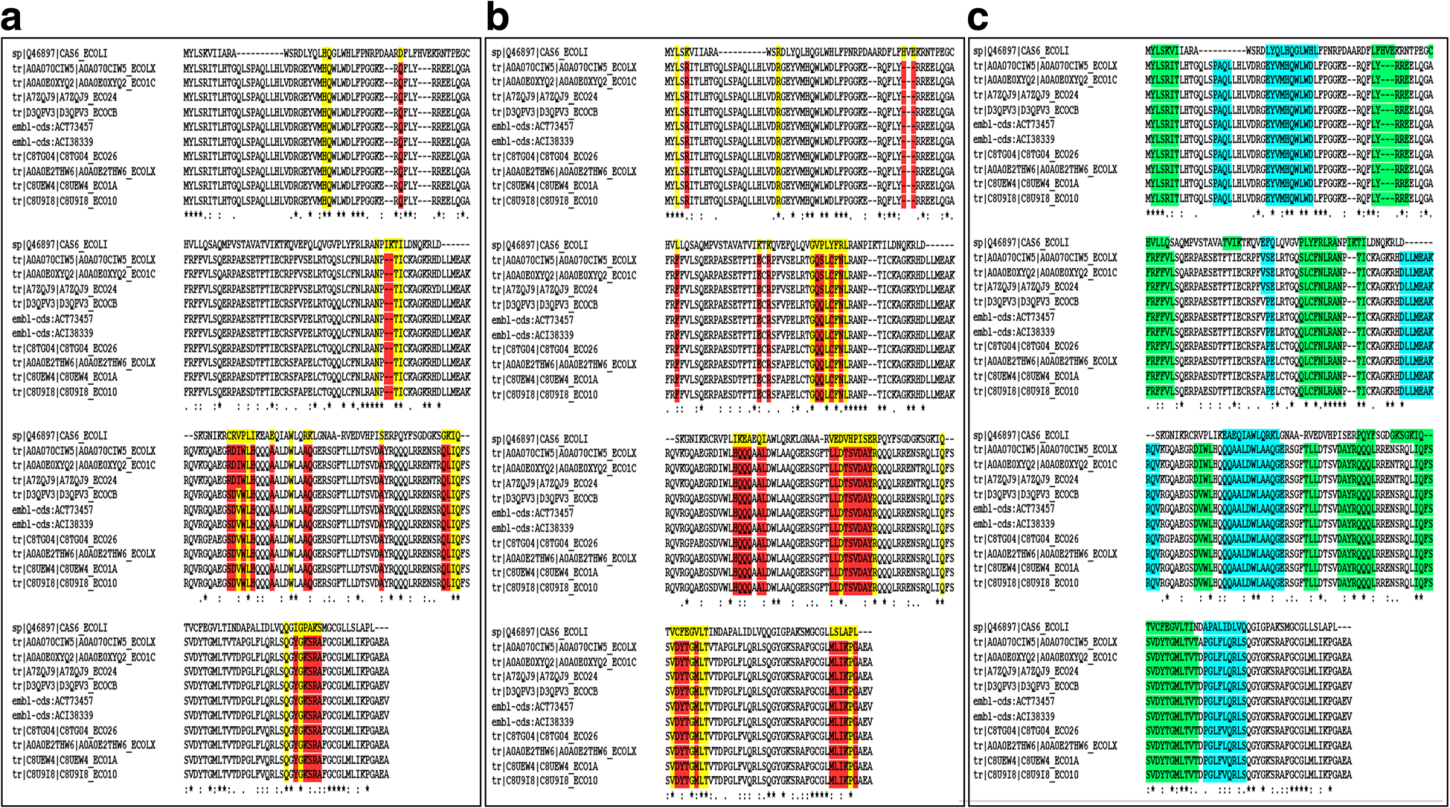
Sequence analysis of pathogen-specific Cas6-like proteins. Comparison of sequence features of Cas6 proteins from pathogenic (Cluster 308) and non-pathogenic K12 strains. **a** Comparison of RNA-binding residues. The RNA-binding residues in *E. coli* strain K12 Cas6 protein have been highlighted in *yellow* on its sequence (CAS6_ECOLI) on the MSA. The corresponding residues in the other proteins on the MSA, which are same as that in CAS6_ECOLI, have also been highlighted in *yellow*, whereas those which differ have been highlighted in *red*. **b** Comparison of protein-interacting residues. The protein-interacting residues in *E. coli* strain K12 Cas6 protein have been highlighted in *yellow* on its sequence (CAS6_ECOLI). A similar colour scheme has also been followed here. **c** Secondary structure prediction. The α-helices have been highlighted in *cyan* and the β-strands in *green*

Multiple sequence alignment (MSA) of all the proteins from Cluster 308 and Cas6 from strain K12 has been shown in Fig. 9. The RNA-binding residues in *E. coli* strain K12 Cas6 protein (union set of RNA-binding residues inferred from each of the three known PDB structures (see *Methods* section)) have been highlighted in yellow on its sequence (CAS6_ECOLI) on the MSA. The corresponding residues in the other proteins on the MSA, which are same as that in CAS6_ECOLI, have also been highlighted in yellow, whereas those which differ have been highlighted in red. From Fig. 9a, we can conclude that the majority of the RNA-binding residues in CAS6_ECOLI are not conserved in the pathogen-specific Cas6 proteins, and can be defined as ‘class-specific residues’. A similar colouring scheme has been followed in Fig. 9b, to analyse the conservation of protein-interacting residues in these proteins. From these analyses, we can speculate that due to the presence of a large proportion of ‘class-specific residues’, the RNA-binding properties, as well as protein-protein interactions, *might* be substantially different among the Cas6 proteins from non-pathogenic and pathogenic *E. coli* strains, which might lead to functional divergence. Secondary structures of each of these proteins, mapped on their sequence (α-helices highlighted in cyan and β-strands in green) in Fig. 9c, also hint at slight structural variation among these proteins.

## Discussion

We have employed a sequence search-based method to compare and contrast the proteomes of 16 pathogenic and three non-pathogenic *E. coli* strains as well as to obtain a global picture of the RBP landscape in *E. coli*. The results obtained from this study showed that the pathogenic strains encode a greater number of RBPs in their proteomes, as compared to the non-pathogenic ones. The DEAD domain, involved in RNA metabolism, was found to be the most abundant of all identified RBDs. The complete and distinct RBPome of *E. coli* was also identified by studying all known *E. coli* strains till date. In this study, we identified RBPs that were exclusive to pathogenic strains, and most of them can be exploited as drug targets by virtue of being non-homologous to their human host proteins. Many of these pathogen-specific proteins were uncharacterised and their identities could be resolved on the basis of sequence homology searches with known proteins.

Further, in this study, we performed three case studies on interesting RBPs. In the first of the three studies, a tRNA processing RNase PH enzyme from strain K12 was investigated that is different from that in all other *E. coli* strains in having a truncated C-terminus and being functionally inactive. Structural modelling and molecular dynamics studies showed that the loss of stability of the monomeric and/or the hexameric (biological unit) forms of this protein from *E. coli* strain K12, might be the possible reason for the lack of its functional activity. In the second study, a previously uncharacterised pathogen-specific protein was studied and was found to possess subtly different RNA-binding affinities towards the same RNA stretch as compared to its well characterised homologues in archaea and eukaryotes. This might hint at different functions of these proteins. In the third case study, pathogen-specific CRISPR-associated Cas6 proteins were analysed and found to have diverged functionally from the known prototypical Cas6 proteins.

## Conclusions

The approach used in our study to cross-compare proteomes of pathogenic and non-pathogenic strains may also be extended to other bacterial or even eukaryotic proteomes to understand interesting differences in their RBPomes. The pathogen-specific RBPs reported in this study, may also be taken up further for clinical trials and/or experimental validations.

The effect of the absence of a functional RNase PH in *E. coli* strain K12 is not clear. The role of the PELOTA_1 domain-containing protein may also be reinforced performing knockdown and rescue experiments. These might help to understand the functional overlap of this protein with its archaeal or eukaryotic homologues. Introduction of this pathogen-specific protein in non-pathogens might also provide probable answers towards its virulence properties. The less conserved RNA-binding and protein-interacting residues in the pathogen-specific Cas6 proteins, might point to functional divergence of these proteins from the known ones, but warrants further investigation.

## Additional files

Additional file 1: Table S1. RNA-binding proteins in 19 *E. coli* proteomes. All the RBPs obtained in the GWS of 19 *E. coli* strains have been listed in this table. The pathogenic and non-pathogenic *E. coli* strains have been highlighted in red and green, respectively. (DOC 115 kb)

Additional file 2: Table S2. Clusters of RNA-binding proteins obtained from 19 *E. coli* proteomes. The clusters of RBPs with more than one member in the GWS of 19 *E. coli* strains have been listed in this table. The RBPs were clustered based on BLASTP searches at E-value, percentage identity and percentage query coverage cut-offs of 10^−5^, 30 and 70, respectively. (DOC 376 kb)

Additional file 3: RNA-binding proteins in all complete *E. coli* proteomes. All the RBPs obtained in the GWS of 166 *E. coli* strains have been listed here. The RefSeq IDs of the proteins are listed along with the total number of strains in which the protein is present mentioned in brackets. (DOC 185 kb)

Additional file 4: 100 ns molecular dynamics simulations of the active RNase PH monomer in the AMBER99SB protein, nucleic AMBER94 force field. The protein has been colour coded as in Fig. 5b. Hydrogen bonds at distance and angle cut-offs of 3 Å and 20°, respectively, have been shown at the region of interest with black dotted lines. (MP4 50,376 kb)

Additional file 5: 100 ns molecular dynamics simulations of the inactive RNase PH monomer in the AMBER99SB protein, nucleic AMBER94 force field. The protein has been colour coded as in Fig. 5b. Hydrogen bonds at distance and angle cut-offs of 3 Å and 20°, respectively, have been shown at the region of interest with black dotted lines. (MP4 67,789 kb)

Additional file 6: 100 ns molecular dynamics simulations of the active RNase PH dimer in the AMBER99SB protein, nucleic AMBER94 force field. The protein has been colour coded as in Fig. 5b and c. Hydrogen bonds at distance and angle cut-offs of 3 Å and 20°, respectively, have been shown at the region of interest with black dotted lines. (MP4 67,624 kb)

Additional file 7: 100 ns molecular dynamics simulations of the inactive RNase PH dimer in the AMBER99SB protein, nucleic AMBER94 force field. The protein has been colour coded as in Fig. 5b and c. Hydrogen bonds at distance and angle cut-offs of 3 Å and 20°, respectively, have been shown at the region of interest with black dotted lines. (MP4 67,820 kb)

Additional file 8: 100 ns molecular dynamics simulations of the PELOTA_1 domain from the ‘uncharacterised’ protein in complex with kink-turn RNA, in the AMBER99SB protein, nucleic AMBER94 force field. The protein has been represented in blue and the RNA in red. Hydrogen bonds at distance and angle cut-offs of 3 Å and 20°, respectively, have been shown between the protein and the RNA has been shown with black dotted lines. (MP4 54,830 kb)

Additional file 9: 100 ns molecular dynamics simulations of the L7Ae K-turn binding domain from *Archaeoglobus fulgidus* in complex with kink-turn RNA from *H. marismortui* (PDB code: 4BW0: B), in the AMBER99SB protein, nucleic AMBER94 force field. The protein has been represented in blue and the RNA in red. Hydrogen bonds at distance and angle cut-offs of 3 Å and 20°, respectively, have been shown between the protein and the RNA has been shown with black dotted lines. (MP4 66,564 kb)

Additional file 10: Figure S1. Molecular phylogeny analysis of Cas6 proteins. a. All the proteins from Cluster 308 and Cas6 from *E. coli* strain K12. b. Two previously uncharacterised proteins (UniProt IDs: C8U9I8 and C8TG04) from Cluster 308, with other known Cas6 proteins, including that from *E. coli* strain K12. In both the panels, the above-mentioned two previously uncharacterised proteins from the pathogen-specific Cas6 proteins cluster (Cluster 308) have been highlighted in red and the Cas6 protein from *E. coli* strain K12 in blue. (JPEG 4531 kb)

## Abbreviations

ABC: ATP-binding cassette transporters
APBS: Adaptive Poisson-Boltzmann Solver
ASCH: Activating Signal Cointergrator-1 homology
bp: Base pair
Cas: CRISPR-associated system
CRISPR: Clustered Regularly Interspaced Short Palindromic Repeat
crRNA: CRISPR RNA
DA: Domain architecture
DOPE: Discrete Optimized Protein Energy
EHEC: Enterohemorrhagic *E. coli*
Fin: Fertility inhibition
GROMACS: Groningen Machine for Chemical Simulations
GWS: Genome-wide survey
HMM: Hidden Markov Model
i-Evalue: Independent E-value
K-turn: Kink-turn
Matt: Multiple Alignment with Translations and Twists
MD: Molecular dynamics
ML: Maximum Likelihood
MMC: Multi-member cluster
MSA: Multiple sequence alignment
ncRNA: Noncoding RNA
NR: Non-redundant
PDB: Protein Data Bank
Pfam: Protein families database
RBD: RNA-binding domain
RBP: RNA-binding protein
RNase PH: Ribonuclease PH
RNP: Ribonucleoprotein
RsmA: Repressor of secondary metabolites A
SCOP: Structural Classification of Proteins
SMC: Single-member cluster
sRNA: Small RNA
TA: Toxin-antitoxin
tRNA: Transfer RNA

## References

[1] Kaper JB, Nataro JP, Mobley HLT (2004). Pathogenic Escherichia Coli. Nat. Rev. Microbiol..

[2] Hacker J, Bender L, Ott M, Wingender J, Lund B, Marre R (1990). Deletions of chromosomal regions coding for fimbriae and hemolysins occur in vitro and in vivo in various extra intestinal Escherichia Coli isolates. Microb Pathog.

[3] Hacker J, Kaper JB (2000). Pathogenicity Islands and the evolution of microbes. Annu Rev Microbiol.

[4] Hacker J, Blum-Oehler G, Muhldorfer I, Tschape H (1997). Pathogenicity islands of virulent bacteria: structure, function and impact on microbial evolution. Mol Microbiol.

[5] Caprioli A, Morabito S, Brugère H, Oswald E (2005). Enterohaemorrhagic Escherichia Coli: emerging issues on virulence and modes of transmission. Vet Res.

[6] Garmendia J (2005). Frankel gad CVF. Enteropathogenic and Enterohemorrhagic Escherichia Coli infections. Infect Immun.

[7] Perez-Rueda E, Martinez-Nuñez MA (2012). The repertoire of DNA-binding transcription factors in prokaryotes: functional and evolutionary lessons. Sci Prog.

[8] Cusack S (1999). RNA – protein complexes. Curr Opin Struct Biol.

[9] Draper DE (1999). Themes in RNA-protein recognition. J Mol Biol.

[10] Jones S, Daley DT, Luscombe NM, Berman HM, Thornton JM (2001). Protein-RNA interactions: a structural analysis. Nucleic Acids Res.

[11] Chen Y, Varani G (2005). Protein families and RNA recognition. FEBS J.

[12] Hall KB (2002). RNA – protein interactions. Curr Opin Struct Biol.

[13] Schroeder R, Barta A, Semrad K (2004). Strategies for RNA folding and assembly. Nat Rev Mol Cell Biol.

[14] Windbichler N, von Pelchrzim F, Mayer O, Csaszar E, Schroeder R (2008). Isolation of small RNA-binding proteins from E. coli : evidence for frequent interaction of RNAs with RNA polymerase. RNA Biol.

[15] Aiba H (2007). Mechanism of RNA silencing by Hfq-binding small RNAs. Curr Opin Microbiol.

[16] De Lay N, Schu DJ, Gottesman S. Bacterial small RNA-based negative regulation: Hfq and its accomplices. J. Biol. Chem. 2013:7996–8003. 10.1074/jbc.R112.441386 PMC3605619 23362267

[17] Gaballa A, Antelmann H, Aguilar C, Khakh SK, Song K-B, Smaldone GT (2008). The Bacillus Subtilis Iron-sparing response is mediated by a fur-regulated small RNA and three small, basic proteins. Proc Natl Acad Sci U S A.

[18] Geissmann TA, Touati D (2004). Hfq, a new chaperoning role: binding to messenger RNA determines access for small RNA regulator. EMBO J.

[19] Holmqvist E, Vogel J (2013). A small RNA serving both the Hfq and CsrA regulons. Genes Dev.

[20] Van Assche E, Van Puyvelde S, Vanderleyden J, Steenackers HP. RNA-binding proteins involved in post-transcriptional regulation in bacteria. Front. Microbiol. 2015;6. 10.3389/fmicb.2015.00141 PMC4347634 25784899

[21] Liu JM, Camilli A. A broadening world of bacterial small RNAs. Curr Opin Microbiol. 2010:18–23. 10.1016/j.mib.2009.11.004 PMC2822007 20022798

[22] Oliva G, Sahr T, Buchrieser C. Small RNAs, 5′ UTR elements and RNA-binding proteins in intracellular bacteria: Impact on metabolism and virulence. FEMS Microbiol Rev. 2015:331–49. 10.1093/femsre/fuv022 26009640

[23] Sauer E, Schmidt S, Weichenrieder O (2012). Small RNA binding to the lateral surface of Hfq hexamers and structural rearrangements upon mRNA target recognition. Proc Natl Acad Sci.

[24] Prasanth KV, Spector DL (2007). Eukaryotic regulatory RNAs: an answer to the “genome complexity” conundrum. Genes Dev.

[25] Hannon GJ (2002). RNA interference. Nature.

[26] Mattick JS. The Functional Genomics of Noncoding RNA. Science (80-. ). 2005;309:1527–8. 10.1126/science.1117806 16141063

[27] Sonnleitner E, Hagens S, Rosenau F, Wilhelm S, Habel A, Jäger KE (2003). Reduced virulence of a hfq mutant of Pseudomonas Aeruginosa O1. Microb Pathog.

[28] Sittka A, Pfeiffer V, Tedin K, Vogel J (2007). The RNA chaperone Hfq is essential for the virulence of salmonella typhimurium. Mol Microbiol.

[29] Sharma AK, Payne SM (2006). Induction of expression of hfq by DksA is essential for Shigella flexneri virulence. Mol Microbiol.

[30] Ding Y, Davis BM, Waldor MK (2004). Hfq is essential for Vibrio cholerae virulence and downregulates σE expression. Mol Microbiol.

[31] Kendall MM, Gruber CC, Rasko DA, Hughes DT, Sperandio V (2011). Hfq virulence regulation in enterohemorrhagic Escherichia Coli O157:H7 strain 86-24. J Bacteriol.

[32] Chao Y, Vogel J. The role of Hfq in bacterial pathogens. Curr. Opin. Microbiol. 2010. p. 24–33. 10.1016/j.mib.2010.01.001 20080057

[33] Zeng Q, McNally RR, Sundin GW (2013). Global small RNA chaperone Hfq and regulatory small RNAs are important virulence regulators in erwinia amylovora. J Bacteriol.

[34] Christiansen JK, Larsen MH, Ingmer H, Sogaard-Andersen L, Kallipolitis BH (2004). The RNA-binding protein Hfq of Listeria monocytogenes: role in stress tolerance and virulence. J Bacteriol.

[35] Geng J, Song Y, Yang L, Feng Y, Qiu Y, Li G, et al. Involvement of the post-transcriptional regulator Hfq in Yersinia pestis virulence. PLoS One. 2009;4. 10.1371/journal.pone.0006213 PMC2704395 19593436

[36] Wilf NM, Reid AJ, Ramsay JP, Williamson NR, Croucher NJ, Gatto L (2013). RNA-seq reveals the RNA binding proteins, Hfq and RsmA, play various roles in virulence, antibiotic production and genomic flux in Serratia sp. ATCC 39006. BMC Genomics.

[37] Pessi G, Williams F, Hindle Z, Heurlier K, Holden MTG, Cámara M (2001). The global posttranscriptional regulator RsmA modulates production of virulence determinants and N-acylhomoserine lactones in Pseudomonas Aeruginosa. J Bacteriol.

[38] Liaw SJ, Lai HC, Ho SW, Luh KT, Wang WB (2003). Role of RsmA in the regulation of swarming motility and virulence factor expression in Proteus Mirabilis. J Med Microbiol.

[39] Mulcahy H, O’Callaghan J, O’Grady EP, Maciá MD, Borrell N, Gómez C (2008). Pseudomonas Aeruginosa RsmA plays an important role during murine infection by influencing colonization, virulence, persistence, and pulmonary inflammation. Infect Immun.

[40] Mulcahy H, O’Callaghan J, O’Grady EP, Adams C, O’Gara F (2006). The posttranscriptional regulator RsmA plays a role in the interaction between Pseudomonas Aeruginosa and human airway epithelial cells by positively regulating the type III secretion system. Infect Immun.

[41] Chao N-X, Wei K, Chen Q, Meng Q-L, Tang D-J, He Y-Q (2008). The rsmA -like gene rsmA Xcc of Xanthomonas campestris pv. Campestris is involved in the control of various cellular processes, including pathogenesis. Mol. Plant-Microbe Interact.

[42] Vercruysse M, Köhrer C, Davies BW, Arnold MFF, Mekalanos JJ, RajBhandary UL, et al. The Highly Conserved Bacterial RNase YbeY Is Essential in Vibrio cholerae, Playing a Critical Role in Virulence, Stress Regulation, and RNA Processing. Klose KE, editor. PLoS Pathog. 2014;10:e1004175. 10.1371/journal.ppat.1004175 PMC4047096 24901994

[43] Ghosh P, Sowdhamini R (2016). Genome-wide survey of putative RNA-binding proteins encoded in the human proteome. Mol BioSyst Royal Society of Chemistry.

[44] Bateman A, Martin MJ, O’Donovan C, Magrane M, Apweiler R, Alpi E (2015). UniProt: a hub for protein information. Nucleic Acids Res.

[45] Tatusova T, Ciufo S, Federhen S, Fedorov B, McVeigh R, O’Neill K (2015). Update on RefSeq microbial genomes resources. Nucleic Acids Res.

[46] Altschul SF, Gish W, Miller W, Myers EW, Lipman DJ (1990). Basic local alignment search tool. J Mol Biol.

[47] Kaushik S, Mutt E, Chellappan A, Sankaran S, Srinivasan N, Sowdhamini R. Improved Detection of Remote Homologues Using Cascade PSI-BLAST: Influence of Neighbouring Protein Families on Sequence Coverage. Promponas VJ, editor. PLoS One. 2013;8:e56449. 10.1371/journal.pone.0056449 PMC3577913 23437136

[48] Šali A, Blundell TL (1993). Comparative protein Modelling by satisfaction of spatial restraints. J Mol Biol.

[49] Laskowski RA, Macarthur MW, Moss DS, Thornton JM (1993). PROCHECK: a program to check the stereochemical quality of proteins structures. J Appl Crystallogr.

[50] Profiles T (1997). VERIFY3D : assessment of protein models with three- dimensional profiles. Methods Enzymol.

[51] Wiederstein M, Sippl MJ (2007). ProSA-web: interactive web service for the recognition of errors in three-dimensional structures of proteins. Nucleic Acids Res.

[52] Pugalenthi G, Shameer K, Srinivasan N, Sowdhamini R (2006). HARMONY: a server for the assessment of protein structures. Nucleic Acids Res.

[53] Wang J, Cieplak P, Kollman PA (2000). How well does a restrained electrostatic potential (RESP) model perform in calculating conformational energies of organic and biological molecules?. J Comput Chem.

[54] Berendsen HJC, van der Spoel D, van Drunen R (1995). GROMACS: a message-passing parallel molecular dynamics implementation. Comput Phys Commun.

[55] Krissinel E, Henrick K (2007). Inference of macromolecular assemblies from crystalline state. J Mol Biol.

[56] Dolinsky TJ, Nielsen JE, McCammon JA, Baker NA (2004). PDB2PQR: an automated pipeline for the setup of Poisson-Boltzmann electrostatics calculations. Nucleic Acids Res.

[57] Unni S, Huang Y, Hanson RM, Tobias M, Krishnan S, Li WW (2011). Web servers and services for electrostatics calculations with APBS and PDB2PQR. J Comput Chem.

[58] Sowdhamini R, Sukhwal A (2015). PPCheck: a Webserver for the quantitative analysis of protein&amp;ndash;protein interfaces and prediction of residue hotspots. Bioinform Biol Insights.

[59] Menke M, Berger B, Cowen L (2008). Matt: local flexibility aids protein multiple structure alignment. PLoS Comput Biol.

[60] Dominguez C, Boelens R, Bonvin AMJJ (2003). HADDOCK: a protein−protein docking approach based on biochemical or biophysical information. J Am Chem Soc.

[61] Edgar RC (2004). MUSCLE: multiple sequence alignment with high accuracy and high throughput. Nucleic Acids Res.

[62] Kumar S, Stecher G, Tamura K (2016). MEGA7: molecular evolutionary genetics analysis version 7.0 for bigger datasets. Mol Biol Evol.

[63] Kumar S, Stecher G, Peterson D, Tamura K (2012). MEGA-CC: computing core of molecular evolutionary genetics analysis program for automated and iterative data analysis. Bioinformatics.

[64] Jones DT (1999). Protein secondary structure prediction based on position-specific scoring matrices. J Mol Biol.

[65] Linder P, Jankowsky E (2011). From unwinding to clamping — the DEAD box RNA helicase family. Nat Rev Mol Cell Biol Nature Publishing Group.

[66] Blattner FR. The Complete Genome Sequence of *Escherichia coli* K-12. Science (80-. ). 1997;277:1453–62. 10.1126/science.277.5331.1453 9278503

[67] Kim S-H, Hung L-W, Wang IX, Nikaido K, Liu P-Q, Ames GF-L. No Title. Nature. 1998;396:703–7. 10.1038/25393 9872322

[68] Story RM, Steitz TA (1992). Structure of the recA protein-ADP complex. Nature.

[69] Abrahams JP, Leslie AGW, Lutter R, Walker JE (1994). Structure at 2.8 Â resolution of F1-ATPase from bovine heart mitochondria. Nature.

[70] Dong J, Lai R, Jennings JL, Link AJ, Hinnebusch AG (2005). The novel ATP-binding cassette protein ARB1 is a shuttling factor that stimulates 40S and 60S ribosome biogenesis. Mol Cell Biol.

[71] Samra N, Atir-Lande A, Pnueli L, Arava Y (2015). The elongation factor eEF3 (Yef3) interacts with mRNA in a translation independent manner. BMC Mol Biol.

[72] Rodnina MV (2010). Protein synthesis meets ABC ATPases: new roles for Rli1/ABCE1. EMBO Rep.

[73] Van Melderen L, De Bast MS. Bacterial toxin-Antitoxin systems: More than selfish entities? PLoS Genet. 2009. 10.1371/journal.pgen.1000437 PMC2654758 19325885

[74] Van Melderen L. Toxin-antitoxin systems: Why so many, what for? Curr Opin Microbiol. 2010:781–5. 10.1016/j.mib.2010.10.006 21041110

[75] Goeders N, Van Melderen L. Toxin-antitoxin systems as multilevel interaction systems. Toxins (Basel). 2013:304–24. 10.3390/toxins6010304 PMC3920263 24434905

[76] Buts L, Lah J, Dao-Thi MH, Wyns L, Loris R. Toxin-antitoxin modules as bacterial metabolic stress managers. Trends Biochem Sci. 2005:672–9. 10.1016/j.tibs.2005.10.004 16257530

[77] Gerdes K, Christensen SK, Løbner-Olesen A (2005). Prokaryotic toxin-antitoxin stress response loci. Nat. Rev. Microbiol..

[78] Jankowsky E, Fairman ME (2007). RNA helicases--one fold for many functions. Curr Opin Struct Biol.

[79] Jankowsky E. RNA helicases at work: Binding and rearranging. Trends Biochem Sci. 2011:19–29. 10.1016/j.tibs.2010.07.008 PMC3017212 20813532

[80] Hamma T, Ferré-D’Amaré AR (2006). Pseudouridine Synthases. Chem Biol.

[81] Phadtare S, Alsina J, Inouye M. Cold-shock response and cold-shock proteins. Curr Opin Microbiol. 1999:175–80. 10.1016/S1369-5274(99)80031-9 10322168

[82] Yamanaka K (1999). Cold shock response in Escherichia Coli. J Mol Microbiol Biotechnol.

[83] Fozo EM, Kawano M, Fontaine F, Kaya Y, Mendieta KS, Jones KL (2008). Repression of small toxic protein synthesis by the sib and OhsC small RNAs. Mol Microbiol.

[84] Louwen R, Staals RHJ, Endtz HP, van Baarlen P, van der Oost J (2014). The role of CRISPR-Cas Systems in Virulence of pathogenic bacteria. Microbiol Mol Biol Rev.

[85] Iyer LM, Koonin E V, Aravind L. No Title. Genome Biol. 2002;3:research0012.1. 10.1186/gb-2002-3-3-research0012 PMC88810 11897024

[86] Arthur DC, Ghetu AF, Gubbins MJ, Edwards RA, Frost LS, Glover JNM (2003). FinO is an RNA chaperone that facilitates sense-antisense RNA interactions. EMBO J.

[87] Arthur DC, Edwards RA, Tsutakawa S, Tainer JA, Frost LS, Glover JNM (2011). Mapping interactions between the RNA chaperone FinO and its RNA targets. Nucleic Acids Res.

[88] Ghetu AF, Gubbins MJ, Frost LS, Glover JN (2000). Crystal structure of the bacterial conjugation repressor finO. Nat Struct Biol.

[89] Mark Glover JN, Chaulk SG, Edwards RA, Arthur D, Lu J, Frost LS (2015). The FinO family of bacterial RNA chaperones. Plasmid.

[90] Iyer LM, Burroughs AM, Aravind L (2006). The ASCH superfamily: novel domains with a fold related to the PUA domain and a potential role in RNA metabolism. Bioinformatics.

[91] Deutscher MP, Marshall GT, Cudny H (1988). RNase PH: an Escherichia Coli phosphate-dependent nuclease distinct from polynucleotide phosphorylase. Proc Natl Acad Sci.

[92] Kelly KO, Deutscher MP (1992). Characterization of Escherichia Coli RNase PH. J Biol Chem.

[93] Wen T, Oussenko IA, Pellegrini O, Bechhofer DH, Condon C (2005). Ribonuclease PH plays a major role in the exonucleolytic maturation of CCA-containing tRNA precursors in Bacillus Subtilis. Nucleic Acids Res.

[94] Jensen KF (1993). The Escherichia Coli K-12 “wild types” W3110 and MG1655 have an rph frameshift mutation that leads to pyrimidine starvation due to low pyrE expression levels. J Bacteriol.

[95] Harlow LS, Kadziola A, Jensen KF, Larsen S (2004). Crystal structure of the phosphorolytic exoribonuclease RNase PH from Bacillus Subtilis and implications for its quaternary structure and tRNA binding. Protein Sci.

[96] Choi JM, Park EY, Kim JH, Chang SK, Cho Y (2004). Probing the functional importance of the Hexameric ring structure of RNase PH. J Biol Chem.

[97] Anantharaman V (2002). Comparative genomics and evolution of proteins involved in RNA metabolism. Nucleic Acids Res.

[98] Anantharaman V, Iyer LM, Aravind L (2012). Ter-dependent stress response systems: novel pathways related to metal sensing, production of a nucleoside-like metabolite, and DNA-processing. Mol BioSyst.

[99] Huang L, Lilley DMJ (2013). The molecular recognition of kink-turn structure by the L7Ae class of proteins. RNA.

[100] Barrangou R, Marraffini LA. CRISPR-cas systems: Prokaryotes upgrade to adaptive immunity. Mol Cell. 2014:234–44. 10.1016/j.molcel.2014.03.011 PMC4025954 24766887

[101] Jiang F, Doudna JA. The structural biology of CRISPR-Cas systems. Curr Opin Struct Biol. 2015:100–11. 10.1016/j.sbi.2015.02.002 PMC4417044 25723899

[102] van der Oost J, Westra ER, Jackson RN, Wiedenheft B (2014). Unravelling the structural and mechanistic basis of CRISPR-Cas systems. Nat Rev Microbiol.

