# Supplementary material for: Bioinformatics comparisons of RNA-binding proteins of pathogenic and non-pathogenic Escherichia coli strains reveal novel virulence factors
Source: BMC Genomics. 2017 Aug 24;18:658. doi: 10.1186/s12864-017-4045-3 (PMC5571608; doi:10.1186/s12864-017-4045-3)
Supplement: Supplementary file 1 — RNA-binding proteins in 19 E. coli proteomes. All the RBPs obtained in the GWS of 19 E. coli strains have been listed in this table. The pathogenic and non-pathogenic E. coli strains have been highlighted in red and green, respectively. (DOC 115 kb) [file 12864_2017_4045_MOESM1_ESM.doc]

Additional File 1: RNA-binding proteins in 19 *E. coli* proteomes.

All the RBPs obtained in the GWS of 19 *E. coli* strains have been listed in this table. The pathogenic and the non-pathogenic *E. coli* strains have been highlighted in red and green respectively.

| ***E. coli* strain name** | **UniProt IDs** |
| --- | --- |
| **O121:H19 str. 2010C-3609** | A0A0E2TFI2, A0A0E2TFK4, A0A0E2TG12, A0A0E2TGH9, A0A0E2TGW0, A0A0E2TH25, A0A0E2TH37, A0A0E2TH56, A0A0E2THH6, A0A0E2THJ6, A0A0E2THK1, A0A0E2THK4, A0A0E2THM6, A0A0E2THP4, A0A0E2THP6, A0A0E2THV2, A0A0E2THV7, A0A0E2THW0, A0A0E2THW6, A0A0E2THX4, A0A0E2THX7, A0A0E2THY1, A0A0E2THY4, A0A0E2THY9, A0A0E2THZ3, A0A0E2THZ5, A0A0E2THZ6, A0A0E2THZ9, A0A0E2TI04, A0A0E2TI06, A0A0E2TI09, A0A0E2TI14, A0A0E2TI33, A0A0E2TI47, A0A0E2TI66, A0A0E2TI70, A0A0E2TI91, A0A0E2TI97, A0A0E2TI99, A0A0E2TIC3, A0A0E2TIC7, A0A0E2TID2, A0A0E2TID4, A0A0E2TID6, A0A0E2TID9, A0A0E2TIE5, A0A0E2TIE8, A0A0E2TIG2, A0A0E2TIJ9, A0A0E2TIL8, A0A0E2TIP7, A0A0E2TIQ7, A0A0E2TIT3, A0A0E2TIT9, A0A0E2TIX6, A0A0E2TIX7, A0A0E2TIY0, A0A0E2TIY4, A0A0E2TJ14, A0A0E2TJ41, A0A0E2TJ96, A0A0E2TJB1, A0A0E2TJF0, A0A0E2TJF1, A0A0E2TJJ8, A0A0E2TJK2, A0A0E2TJQ1, A0A0E2TJR8, A0A0E2TJS7, A0A0E2TJT0, A0A0E2TJT2, A0A0E2TJU4, A0A0E2TJU8, A0A0E2TJX2, A0A0E2TJY2, A0A0E2TJY5, A0A0E2TJY7, A0A0E2TJZ4, A0A0E2TK21, A0A0E2TK34, A0A0E2TK69, A0A0E2TK78, A0A0E2TKE0, A0A0E2TKE8, A0A0E2TKG4, A0A0E2TKI2, A0A0E2TKJ6, A0A0E2TKJ9, A0A0E2TKL1, A0A0E2TKL6, A0A0E2TKM3, A0A0E2TKQ8, A0A0E2TKS9, A0A0E2TKU4, A0A0E2TKW9, A0A0E2TL22, A0A0E2TL53, A0A0E2TL57, A0A0E2TL93, A0A0E2TL94, A0A0E2TL97, A0A0E2TLA3, A0A0E2TLB8, A0A0E2TLD8, A0A0E2TLF1, A0A0E2TLJ2, A0A0E2TLK9, A0A0E2TLM2, A0A0E2TLP0, A0A0E2TLQ0, A0A0E2TLQ4, A0A0E2TLQ6, A0A0E2TLQ8, A0A0E2TLR1, A0A0E2TLR6, A0A0E2TLS2, A0A0E2TLS5, A0A0E2TLS9, A0A0E2TLU8, A0A0E2TM05, A0A0E2TM09, A0A0E2TM18, A0A0E2TM19, A0A0E2TM34, A0A0E2TM45, A0A0E2TM70, A0A0E2TM78, A0A0E2TM88, A0A0E2TM95, A0A0E2TM99, A0A0E2TMB3, A0A0E2TMD1, A0A0E2TME7, A0A0E2TMG8, A0A0E2TMG9, A0A0E2TMJ5, A0A0E2TML4, A0A0E2TML7, A0A0E2TMM2, A0A0E2TMM9, A0A0E2TMP1, A0A0E2TMP9, A0A0E2TMQ3, A0A0E2TMQ6, A0A0E2TMQ7, A0A0E2TMT4, A0A0E2TMX0, A0A0E2TN06, A0A0E2TN38, A0A0E2TN57, A0A0E2TN69, A0A0E2TN73, A0A0E2TN96, A0A0E2TN98, A0A0E2TNB0, A0A0E2TNB5, A0A0E2TNI5, A0A0E2TNL3, A0A0E2TNM1, A0A0E2TNM4, A0A0E2TNM9, A0A0E2TNN3, A0A0E2TNP0, A0A0E2TNS7, A0A0E2TNS8, A0A0E2TNT0, A0A0E2TNT5, A0A0E2TNT8, A0A0E2TNU6, A0A0E2TNV9, A0A0E2TNW4, A0A0E2TNZ7, A0A0E2TP14, A0A0E2TP15, A0A0E2TP17, A0A0E2TP27, A0A0E2TP32, A0A0E2TP36, A0A0E2TP38, A0A0E2TP63, A0A0E2TP73, A0A0E2TPB8, A0A0E2TPC6, A0A0E2TPD0, A0A0E2TPD7, A0A0E2TPE5, A0A0E2TPG1, A0A0E2TPH3, A0A0E2TPK6, A0A0E2TPL0, A0A0E2TPM2, A0A0E2TPN5, A0A0E2TPP1, A0A0E2TPU5, A0A0E2TPW0, A0A0E2TPX9, A0A0E2TPZ3, A0A0E2TPZ7, A0A0E2TQ19, A0A0E2TQ39, A0A0E2TQ45, A0A0E2TQ49, A0A0E2TQ76, A0A0E2TQ87, A0A0E2TQG0, A0A0E2TQI4, A0A0E2TQI9, A0A0E2TQJ3, A0A0E2TQJ4, A0A0E2TQL3, A0A0E2TQN1, A0A0E2TQN2, A0A0E2TQQ5, A0A0E2TQQ6, A0A0E2TQS8, A0A0E2TQT9, A0A0E2TQV6, A0A0E2TQV9, A0A0E2TQW7, A0A0E2TQZ0, A0A0E2TR15, A0A0E2TR24, A0A0E2TR25, A0A0E2TR31, A0A0E2TR33, A0A0E2TR53, A0A0E2TR54, A0A0E2TR61, A0A0E2TR79, A0A0E2TR84, A0A0E2TRA1, A0A0E2TRB3, A0A0E2TRE0, A0A0E2TRE4, A0A0E2TRF4, A0A0E2TRG9, A0A0E2TRI9, A0A0E2TRP0, A0A0E2TRZ0, A0A0E2TRZ1, A0A0E2TS00, A0A0E2TS01, A0A0E2TS15, A0A0E2TS16, A0A0E2TS24, A0A0E2TS39, A0A0E2TS45, A0A0E2TS63, A0A0E2TS81, A0A0E2TS91, A0A0E2TSA1, A0A0E2TSB6, A0A0E2TSC0, A0A0E2TSG6, A0A0E2TSH1, A0A0E2TSI1, A0A0E2TSK0, A0A0E2TSK1, A0A0E2TSK6, A0A0E2TSL6, A0A0E2TSM9, A0A0E2TSR7, A0A0E2TSV6, A0A0E2TSW1, A0A0E2TSW4, A0A0E2TSW8, A0A0E2TSY2, A0A0E2TSZ4, A0A0E2TT00, A0A0E2TT24, A0A0E2TT41, A0A0E2TT48, A0A0E2TT73, A0A0E2TT78, A0A0E2TT81, A0A0E2TT91, A0A0E2TTB5, A0A0E2TTJ1, A0A0E2TTJ5, A0A0E2TTP8, A0A0E2TTR1, A0A0E2TTR4, A0A0E2TTR6, A0A0E2TTR7, A0A0E2TTS2, A0A0E2TTS8, A0A0E2TTT4, A0A0E2TTU5, A0A0E2TTV2, A0A0E2TTV7, A0A0E2TTW0, A0A0E2TTW1, A0A0E2TTW4, A0A0E2TTZ9, A0A0E2TU59, A0A0E2TU63, A0A0E2TU74, A0A0E2TU92, A0A0E2TUD1, A0A0E2TUD4, A0A0E2TUE0, A0A0E2TUE7, A0A0E2TUH7, A0A0E2TUI5, A0A0E2TUM7, A0A0E2TUR5, A0A0E2TUT1, A0A0E2TUU8, A0A0E2TUV8, A0A0E2TUW3, A0A0E2TUY1, A0A0E2TV05, A0A0E2TV13, A0A0E2TV18, A0A0E2TV23, A0A0E2TV58, A0A0E2TV63, A0A0E2TV86, A0A0E2TV90, A0A0E2TVK2, A0A0E2TVV9, A0A0E2TW93, A0A0E2TWD6, A0A0E2TWG5, A0A0E2TWM0, A0A0E2TWY6, A0A0E2TX09, A0A0E2TXC6, A0A0E2TXG4, A0A0E2TXJ9, A0A0E2TXL1, A0A0E2TXL4, A0A0E2TXN3, A0A0E2TXU8, A0A0E2TXY9, A0A0E2TY29, A0A0E2TY84, A0A0E2TYC2, A0A0E2TZ09, A0A0E2TZ44, A0A0E2TZ47, A0A0E2TZ68, A0A0E2TZB3, A0A0E2TZB8, A0A0E2TZE4, A0A0E2TZF9, A0A0E2TZH1, A0A0E2TZI5, A0A0E2TZJ1, A0A0E2TZK2, A0A0E2TZM8, A0A0E2TZP3, A0A0E2TZP6, A0A0E2TZQ0, A0A0E2TZQ4, A0A0E2TZQ9, A0A0E2TZR4, A0A0E2TZR9, A0A0E2U041, A0A0E2U071, A0A0E2U0A6, A0A0E2U0G0, A0A0E2U0I1, A0A0E2U105, A0A0E2U114, A0A0E2U1B4, A0A0E2U1P2, A0A0E2U1S6, A0A0E2U1U7, A0A0E2U247, A0A0E2U259, A0A0E2U2K9, A0A0E2U2P1, A0A0E2U2U3, A0A0E2U2V5, A0A0E2U386, A0A0E2U391, A0A0E2U3B2, A0A0E2U3F1, A0A0E2U3K8, A0A0E2U3S4, A0A0E2U3Y9, A0A0E2U427, A0A0E2U435, A0A0E2U4B8, A0A0E2U4F7, A0A0E2U4L7, A0A0E2U4S2, A0A0E2U5F2, A0A0E2U5H3, A0A0E2U5K9, A0A0E2U5T1, A0A0E2U5Y1, A0A0E2U5Y5, A0A0E2U616, A0A0E2U6G4, A0A0E2U6V9, A0A0E2U774, A0A0E2U7A3, A0A0E2U7E0, A0A0E2U7K7, A0A0E2U7U6, A0A0E2U812, A0A0E2U8I3, A0A0E2U8K8, A0A0E2U8L3, A0A0E2U8N0, A0A0E2U8R1, A0A0E2U8R9, A0A0E2U8U1, A0A0E2U909, A0A0E2U9B9, A0A0E2U9I6, A0A0E2U9J6, A0A0E2U9S9, A0A0E2UA15, A0A0E2UA46, A0A0E2UA97, A0A0E2UAD4 |
| **strain K12** | A5A627, C1P607, C1P608, C1P615, C1P616, C1P617, P00579, P00582, P00954, P00956, P00957, P00959, P00960, P00961, P00962, P02358, P02359, P02413, P03007, P03061, P04079, P04805, P04983, P04993, P04994, P04995, P05055, P05523, P06611, P06616, P06961, P06992, P07012, P07109, P07118, P07364, P07395, P07649, P07813, P07821, P08179, P08312, P08365, P08956, P09155, P09833, P0A698, P0A6A0, P0A6A6, P0A6M4, P0A6M8, P0A6P1, P0A6P5, P0A6U3, P0A6U5, P0A6X1, P0A6X3, P0A705, P0A707, P0A780, P0A784, P0A7A5, P0A7B5, P0A7D1, P0A7F9, P0A7I0, P0A7I4, P0A7J3, P0A7J7, P0A7K2, P0A7K6, P0A7L0, P0A7L3, P0A7L8, P0A7M2, P0A7M6, P0A7M9, P0A7N4, P0A7N9, P0A7P5, P0A7Q1, P0A7Q6, P0A7R1, P0A7R5, P0A7R9, P0A7S3, P0A7S9, P0A7T3, P0A7T7, P0A7U3, P0A7U7, P0A7V0, P0A7V3, P0A7V8, P0A7W1, P0A7W7, P0A7X3, P0A7X6, P0A7Y0, P0A7Y4, P0A7Y8, P0A7Z4, P0A800, P0A805, P0A821, P0A832, P0A847, P0A850, P0A873, P0A887, P0A8D6, P0A8F8, P0A8J8, P0A8L1, P0A8M0, P0A8M3, P0A8N3, P0A8N5, P0A8N7, P0A8T7, P0A8V0, P0A8V2, P0A968, P0A972, P0A976, P0A978, P0A982, P0A986, P0A9H7, P0A9J0, P0A9P6, P0A9R7, P0A9S7, P0A9T8, P0A9U1, P0A9U3, P0A9V1, P0A9V5, P0A9W3, P0A9X1, P0A9X9, P0A9Y6, P0AA10, P0AA37, P0AA39, P0AA41, P0AA43, P0AAF3, P0AAF6, P0AAG0, P0AAG3, P0AAG5, P0AAG8, P0AAH0, P0AAH4, P0AAH8, P0AAI1, P0AAL3, P0AAR3, P0AAZ7, P0ABB4, P0ABF1, P0ABH0, P0ABI4, P0ABS5, P0ABT5, P0ABU2, P0ACC1, P0ACE7, P0ACG4, P0ACG6, P0ACG8, P0AD89, P0ADR6, P0ADY3, P0ADY7, P0ADZ0, P0ADZ4, P0AE01, P0AE70, P0AE72, P0AEB7, P0AEG4, P0AEK0, P0AF93, P0AFF6, P0AFQ5, P0AFR4, P0AFX4, P0AG30, P0AG44, P0AG48, P0AG51, P0AG55, P0AG59, P0AG63, P0AG67, P0AG96, P0AG99, P0AGA2, P0AGD7, P0AGJ2, P0AGJ5, P0AGJ7, P0AGJ9, P0AGK4, P0AGL2, P0AGL7, P0C018, P0C0R7, P0CE47, P0CE48, P0CG19, P10121, P10346, P10408, P10442, P10443, P10907, P11875, P11989, P14081, P14175, P15031, P15038, P15043, P15723, P16384, P16659, P16676, P16677, P16678, P16679, P17888, P21499, P21507, P21513, P21693, P21888, P21889, P21893, P22731, P23003, P23845, P23878, P23882, P23886, P23909, P24230, P24255, P25516, P25519, P25522, P25539, P25736, P25745, P25888, P25889, P27296, P27305, P28369, P28632, P29018, P30014, P30015, P30131, P30750, P30850, P30958, P31060, P31134, P31548, P31806, P31825, P31826, P32132, P32684, P32695, P32721, P33236, P33360, P33371, P33593, P33594, P33595, P33643, P33647, P33650, P33916, P33919, P33931, P33941, P36566, P36683, P36879, P36929, P36995, P37005, P37009, P37024, P37025, P37051, P37305, P37313, P37388, P37624, P37634, P37765, P37774, P38035, P38036, P38394, P39199, P39286, P39332, P39380, P39394, P40711, P42589, P42641, P43329, P43672, P45577, P45748, P45769, P46837, P46849, P46850, P46855, P46890, P50465, P52097, P52119, P52126, P52129, P52612, P54901, P55135, P60240, P60340, P60422, P60438, P60624, P60664, P60723, P60752, P60757, P60785, P60906, P61175, P62395, P62399, P62522, P63177, P63386, P63389, P64423, P64483, P67603, P68187, P68398, P68679, P68919, P69441, P69874, P69913, P75675, P75764, P75796, P75817, P75831, P75864, P75957, P75966, P76027, P76055, P76145, P76257, P76273, P76562, P76632, P76909, P77091, P77223, P77257, P77265, P77268, P77279, P77398, P77481, P77494, P77499, P77509, P77622, P77718, P77736, P77737, P77795, Q2EEQ2, Q46864, Q46865, Q46897, Q46898, Q46899, Q46901, Q47157, Q47538, Q47688, Q57261, Q6BEX0, Q6BF25, Q6BF86, Q6BF87 |
| **Nissle 1917** | AID77074, AID77080, AID77082, AID77110, AID77115, AID77116, AID77125, AID77128, AID77131, AID77151, AID77154, AID77155, AID77171, AID77180, AID77189, AID77205, AID77206, AID77209, AID77212, AID77213, AID77216, AID77225, AID77232, AID77233, AID77235, AID77245, AID77246, AID77250, AID77254, AID77260, AID77265, AID77274, AID77275, AID77363, AID77377, AID77378, AID77384, AID77397, AID77428, AID77461, AID77495, AID77496, AID77508, AID77510, AID77517, AID77531, AID77540, AID77544, AID77545, AID77568, AID77575, AID77585, AID77589, AID77613, AID77637, AID77644, AID77678, AID77696, AID77699, AID77725, AID77750, AID77783, AID77788, AID77794, AID77803, AID77818, AID77821, AID77823, AID77833, AID77843, AID77851, AID77879, AID77883, AID77888, AID77903, AID77908, AID77909, AID77915, AID77929, AID77932, AID77934, AID77938, AID77947, AID77950, AID77958, AID77959, AID77971, AID77989, AID77990, AID78007, AID78060, AID78072, AID78093, AID78108, AID78144, AID78180, AID78181, AID78184, AID78197, AID78210, AID78219, AID78244, AID78305, AID78347, AID78348, AID78356, AID78357, AID78363, AID78364, AID78365, AID78388, AID78400, AID78401, AID78419, AID78421, AID78429, AID78439, AID78447, AID78448, AID78468, AID78491, AID78492, AID78493, AID78511, AID78532, AID78577, AID78671, AID78681, AID78696, AID78704, AID78727, AID78754, AID78758, AID78759, AID78760, AID78761, AID78762, AID78796, AID78829, AID78841, AID78845, AID78846, AID78860, AID78868, AID78871, AID78880, AID78892, AID78900, AID78966, AID78973, AID78986, AID78999, AID79028, AID79080, AID79081, AID79145, AID79164, AID79170, AID79175, AID79176, AID79258, AID79271, AID79280, AID79287, AID79289, AID79319, AID79322, AID79323, AID79324, AID79338, AID79342, AID79348, AID79390, AID79404, AID79437, AID79448, AID79460, AID79511, AID79528, AID79570, AID79584, AID79591, AID79593, AID79598, AID79601, AID79619, AID79622, AID79628, AID79640, AID79647, AID79648, AID79650, AID79656, AID79657, AID79662, AID79671, AID79681, AID79682, AID79683, AID79684, AID79685, AID79692, AID79694, AID79717, AID79731, AID79752, AID79774, AID79786, AID79788, AID79794, AID79797, AID79820, AID79825, AID79842, AID79873, AID79876, AID79927, AID79928, AID79929, AID79937, AID79964, AID79981, AID79982, AID80052, AID80088, AID80172, AID80202, AID80211, AID80212, AID80218, AID80250, AID80298, AID80300, AID80301, AID80302, AID80304, AID80305, AID80317, AID80320, AID80321, AID80324, AID80326, AID80327, AID80336, AID80342, AID80343, AID80363, AID80364, AID80379, AID80391, AID80399, AID80410, AID80414, AID80419, AID80420, AID80426, AID80427, AID80428, AID80429, AID80430, AID80431, AID80432, AID80433, AID80434, AID80435, AID80436, AID80437, AID80438, AID80439, AID80440, AID80441, AID80442, AID80443, AID80444, AID80445, AID80446, AID80447, AID80448, AID80449, AID80450, AID80451, AID80452, AID80453, AID80471, AID80472, AID80473, AID80474, AID80485, AID80510, AID80526, AID80534, AID80536, AID80546, AID80547, AID80548, AID80572, AID80578, AID80584, AID80588, AID80589, AID80597, AID80598, AID80613, AID80614, AID80624, AID80634, AID80646, AID80678, AID80679, AID80681, AID80682, AID80695, AID80696, AID80697, AID80698, AID80699, AID80707, AID80728, AID80729, AID80743, AID80769, AID80770, AID80771, AID80777, AID80783, AID80785, AID80786, AID80797, AID80867, AID80868, AID80870, AID80885, AID80887, AID80894, AID80902, AID80903, AID80911, AID80932, AID80934, AID80966, AID80972, AID81006, AID81008, AID81027, AID81031, AID81038, AID81052, AID81102, AID81103, AID81150, AID81156, AID81157, AID81159, AID81160, AID81161, AID81162, AID81163, AID81164, AID81171, AID81202, AID81216, AID81227, AID81253, AID81276, AID81277, AID81291, AID81300, AID81301, AID81309, AID81330, AID81375, AID81379, AID81380, AID81382, AID81386, AID81387, AID81388, AID81394, AID81395, AID81415, AID81417, AID81418, AID81448, AID81462, AID81474, AID81507, AID81517, AID81541, AID81544, AID81560, AID81571, AID81631, AID81649, AID81681, AID81726, AID81727, AID81768, AID81783, AID81842, AID81864 |
| **O157:H7 (strain TW14359 / EHEC)** | ACT69913, ACT69919, ACT69922, ACT69951, ACT69957, ACT69958, ACT69965, ACT69969, ACT69971, ACT69992, ACT69995, ACT69996, ACT70014, ACT70023, ACT70039, ACT70040, ACT70043, ACT70044, ACT70047, ACT70056, ACT70063, ACT70064, ACT70066, ACT70077, ACT70078, ACT70082, ACT70085, ACT70088, ACT70093, ACT70102, ACT70103, ACT70130, ACT70154, ACT70156, ACT70157, ACT70163, ACT70218, ACT70253, ACT70260, ACT70263, ACT70300, ACT70307, ACT70347, ACT70348, ACT70358, ACT70360, ACT70368, ACT70381, ACT70390, ACT70393, ACT70394, ACT70418, ACT70425, ACT70434, ACT70443, ACT70448, ACT70475, ACT70508, ACT70509, ACT70516, ACT70551, ACT70569, ACT70577, ACT70593, ACT70672, ACT70677, ACT70683, ACT70734, ACT70749, ACT70752, ACT70755, ACT70765, ACT70777, ACT70784, ACT70812, ACT70816, ACT70824, ACT70841, ACT70842, ACT70847, ACT70848, ACT70854, ACT70871, ACT70874, ACT70877, ACT70881, ACT70891, ACT70894, ACT70909, ACT70910, ACT70951, ACT71006, ACT71008, ACT71027, ACT71067, ACT71083, ACT71106, ACT71120, ACT71162, ACT71190, ACT71227, ACT71229, ACT71232, ACT71247, ACT71258, ACT71261, ACT71284, ACT71327, ACT71354, ACT71356, ACT71434, ACT71435, ACT71441, ACT71442, ACT71443, ACT71447, ACT71448, ACT71463, ACT71477, ACT71478, ACT71502, ACT71616, ACT71618, ACT71627, ACT71638, ACT71646, ACT71647, ACT71672, ACT71696, ACT71699, ACT71700, ACT71718, ACT71746, ACT71778, ACT71791, ACT71792, ACT71803, ACT71820, ACT71826, ACT72006, ACT72015, ACT72030, ACT72031, ACT72041, ACT72060, ACT72087, ACT72091, ACT72092, ACT72094, ACT72095, ACT72096, ACT72097, ACT72135, ACT72170, ACT72187, ACT72191, ACT72192, ACT72207, ACT72216, ACT72220, ACT72229, ACT72243, ACT72251, ACT72261, ACT72269, ACT72283, ACT72328, ACT72352, ACT72512, ACT72554, ACT72562, ACT72567, ACT72568, ACT72702, ACT72766, ACT72783, ACT72794, ACT72807, ACT72809, ACT72842, ACT72845, ACT72846, ACT72847, ACT72861, ACT72865, ACT72871, ACT72911, ACT72925, ACT72960, ACT72972, ACT72984, ACT73051, ACT73069, ACT73121, ACT73136, ACT73180, ACT73206, ACT73214, ACT73216, ACT73218, ACT73221, ACT73243, ACT73246, ACT73257, ACT73269, ACT73277, ACT73278, ACT73281, ACT73287, ACT73288, ACT73293, ACT73303, ACT73313, ACT73314, ACT73315, ACT73316, ACT73317, ACT73324, ACT73326, ACT73385, ACT73398, ACT73399, ACT73413, ACT73436, ACT73444, ACT73446, ACT73452, ACT73457, ACT73458, ACT73459, ACT73460, ACT73461, ACT73462, ACT73463, ACT73485, ACT73486, ACT73488, ACT73493, ACT73508, ACT73518, ACT73572, ACT73602, ACT73603, ACT73611, ACT73639, ACT73640, ACT73658, ACT73659, ACT73746, ACT73768, ACT73777, ACT73778, ACT73780, ACT73786, ACT73823, ACT73874, ACT73876, ACT73877, ACT73878, ACT73880, ACT73881, ACT73885, ACT73889, ACT73890, ACT73893, ACT73895, ACT73896, ACT73905, ACT73911, ACT73912, ACT73934, ACT73935, ACT73950, ACT73964, ACT73976, ACT73980, ACT73985, ACT73986, ACT73992, ACT73993, ACT73994, ACT73995, ACT73996, ACT73997, ACT73998, ACT73999, ACT74000, ACT74001, ACT74002, ACT74003, ACT74004, ACT74005, ACT74006, ACT74007, ACT74008, ACT74009, ACT74010, ACT74011, ACT74012, ACT74013, ACT74014, ACT74015, ACT74016, ACT74017, ACT74018, ACT74019, ACT74023, ACT74024, ACT74025, ACT74026, ACT74037, ACT74059, ACT74075, ACT74083, ACT74085, ACT74098, ACT74099, ACT74100, ACT74127, ACT74132, ACT74133, ACT74144, ACT74145, ACT74177, ACT74178, ACT74190, ACT74203, ACT74220, ACT74253, ACT74255, ACT74256, ACT74273, ACT74274, ACT74275, ACT74276, ACT74277, ACT74285, ACT74305, ACT74306, ACT74325, ACT74351, ACT74352, ACT74353, ACT74359, ACT74365, ACT74367, ACT74368, ACT74404, ACT74432, ACT74467, ACT74468, ACT74470, ACT74473, ACT74484, ACT74499, ACT74507, ACT74508, ACT74516, ACT74527, ACT74539, ACT74541, ACT74573, ACT74581, ACT74592, ACT74594, ACT74612, ACT74615, ACT74625, ACT74642, ACT74698, ACT74699, ACT74733, ACT74739, ACT74740, ACT74742, ACT74743, ACT74744, ACT74745, ACT74746, ACT74747, ACT74754, ACT74783, ACT74798, ACT74829, ACT74839, ACT74873, ACT74880, ACT74881, ACT74889, ACT74913, ACT74940, ACT74944, ACT74945, ACT74947, ACT74951, ACT74952, ACT74953, ACT74959, ACT74960, ACT74980, ACT74982, ACT74983, ACT75005, ACT75006, ACT75009, ACT75025, ACT75030, ACT75039, ACT75064, ACT75065, ACT75067, ACT75094, ACT75105, ACT75107, ACT75108, ACT75128, ACT75131, ACT75148, ACT75160, ACT75179, ACT75217, ACT75224, ACT75236 |
| **BL21-Gold(DE3)pLysS AG** | ACT27152, ACT27153, ACT27155, ACT27162, ACT27168, ACT27169, ACT27170, ACT27197, ACT27216, ACT27217, ACT27251, ACT27252, ACT27255, ACT27256, ACT27257, ACT27271, ACT27272, ACT27274, ACT27276, ACT27318, ACT27330, ACT27337, ACT27338, ACT27353, ACT27354, ACT27362, ACT27363, ACT27367, ACT27375, ACT27395, ACT27396, ACT27397, ACT27408, ACT27410, ACT27418, ACT27435, ACT27467, ACT27478, ACT27479, ACT27480, ACT27481, ACT27501, ACT27502, ACT27503, ACT27504, ACT27505, ACT27506, ACT27507, ACT27508, ACT27509, ACT27510, ACT27511, ACT27512, ACT27513, ACT27514, ACT27515, ACT27516, ACT27517, ACT27518, ACT27519, ACT27520, ACT27521, ACT27522, ACT27523, ACT27524, ACT27525, ACT27526, ACT27527, ACT27528, ACT27534, ACT27535, ACT27540, ACT27544, ACT27556, ACT27570, ACT27586, ACT27587, ACT27610, ACT27611, ACT27617, ACT27626, ACT27627, ACT27629, ACT27632, ACT27633, ACT27637, ACT27641, ACT27642, ACT27644, ACT27645, ACT27646, ACT27648, ACT27696, ACT27737, ACT27743, ACT27744, ACT27745, ACT27754, ACT27786, ACT27787, ACT27862, ACT27863, ACT27905, ACT27913, ACT27914, ACT27915, ACT27973, ACT27983, ACT27988, ACT28004, ACT28010, ACT28012, ACT28013, ACT28034, ACT28039, ACT28045, ACT28047, ACT28057, ACT28079, ACT28094, ACT28095, ACT28108, ACT28133, ACT28135, ACT28142, ACT28143, ACT28144, ACT28145, ACT28146, ACT28156, ACT28165, ACT28170, ACT28171, ACT28178, ACT28180, ACT28181, ACT28189, ACT28203, ACT28215, ACT28218, ACT28238, ACT28241, ACT28243, ACT28245, ACT28254, ACT28280, ACT28324, ACT28341, ACT28392, ACT28404, ACT28416, ACT28453, ACT28467, ACT28510, ACT28517, ACT28521, ACT28534, ACT28535, ACT28536, ACT28539, ACT28570, ACT28572, ACT28579, ACT28589, ACT28604, ACT28692, ACT28693, ACT28698, ACT28704, ACT28756, ACT28776, ACT28792, ACT28805, ACT28813, ACT28823, ACT28831, ACT28847, ACT28856, ACT28860, ACT28869, ACT28884, ACT28885, ACT28889, ACT28905, ACT28935, ACT28972, ACT28973, ACT28974, ACT28975, ACT28977, ACT28978, ACT28982, ACT29007, ACT29026, ACT29034, ACT29035, ACT29050, ACT29060, ACT29121, ACT29162, ACT29179, ACT29191, ACT29192, ACT29230, ACT29258, ACT29294, ACT29300, ACT29301, ACT29303, ACT29326, ACT29353, ACT29354, ACT29358, ACT29370, ACT29380, ACT29382, ACT29401, ACT29402, ACT29416, ACT29430, ACT29431, ACT29435, ACT29436, ACT29437, ACT29443, ACT29444, ACT29487, ACT29489, ACT29496, ACT29506, ACT29509, ACT29520, ACT29534, ACT29537, ACT29538, ACT29576, ACT29606, ACT29627, ACT29628, ACT29654, ACT29668, ACT29669, ACT29684, ACT29687, ACT29696, ACT29700, ACT29703, ACT29706, ACT29724, ACT29730, ACT29731, ACT29736, ACT29737, ACT29752, ACT29757, ACT29761, ACT29787, ACT29814, ACT29823, ACT29834, ACT29844, ACT29846, ACT29849, ACT29864, ACT29908, ACT29923, ACT29962, ACT29992, ACT30018, ACT30036, ACT30074, ACT30083, ACT30132, ACT30161, ACT30166, ACT30175, ACT30182, ACT30206, ACT30207, ACT30210, ACT30221, ACT30238, ACT30245, ACT30247, ACT30255, ACT30256, ACT30295, ACT30339, ACT30361, ACT30377, ACT30385, ACT30386, ACT30388, ACT30409, ACT30410, ACT30419, ACT30424, ACT30427, ACT30431, ACT30435, ACT30436, ACT30447, ACT30449, ACT30450, ACT30458, ACT30467, ACT30470, ACT30471, ACT30474, ACT30475, ACT30491, ACT30500, ACT30518, ACT30519, ACT30522, ACT30543, ACT30547, ACT30550, ACT30557, ACT30558, ACT30563, ACT30588, ACT30591, ACT30597, ACT30613, ACT30625, ACT30642, ACT30645, ACT30675, ACT30679, ACT30693, ACT30741, ACT30765, ACT30774, ACT30777, ACT30792, ACT30818, ACT30819, ACT30821, ACT30841, ACT30842, ACT30848, ACT30849, ACT30850, ACT30854, ACT30856, ACT30857, ACT30861, ACT30888, ACT30911, ACT30919, ACT30920, ACT30929, ACT30959, ACT30969, ACT30985, ACT31000, ACT31021, ACT31029, ACT31030, ACT31031, ACT31032, ACT31033, ACT31034, ACT31036, ACT31037, ACT31042, ACT31071, ACT31072, ACT31123, ACT31139, ACT31148, ACT31170, ACT31172, ACT31198, ACT31205, ACT31238, ACT31241, ACT31261, ACT31270, ACT31271, ACT31279, ACT31286, ACT31288, ACT31304, ACT31305, ACT31308, ACT31309 |
| **O145:NM str. 2010C-3526** | A0A025BJX1, A0A025BK68, A0A025BK85, A0A025BKY3, A0A025BL00, A0A025BLA1, A0A025BLR5, A0A025BLS8, A0A025BM83, A0A025BMB0, A0A025BMB7, A0A025BME2, A0A025BMF2, A0A025BMF7, A0A025BMX8, A0A025BN04, A0A025BNL6, A0A025BPT9, A0A025BQP1, A0A025BR00, A0A025BRC5, A0A025BRD7, A0A025BRV8, A0A025BSS6, A0A025BTD6, A0A025BTE4, A0A025BTH2, A0A025BU49, A0A025BUG5, A0A025BVH1, A0A025BVW8, A0A025BW14, A0A025BW30, A0A025BX20, A0A025BX28, A0A025BXW1, A0A025BXY8, A0A025BY06, A0A025BYC4, A0A025BZ96, A0A025BZF8, A0A025BZG8, A0A025BZM8, A0A025BZP5, A0A025BZR7, A0A025BZW5, A0A025BZZ7, A0A025C020, A0A025C0E9, A0A025C1F3, A0A025C1K3, A0A025C1P0, A0A025C1U8, A0A025C207, A0A025C229, A0A025C264, A0A025C275, A0A025C282, A0A025C2N4, A0A025C2R6, A0A025C2S6, A0A025C2U7, A0A025C334, A0A025C371, A0A025C3H4, A0A025C3K7, A0A025C3L4, A0A025C3T6, A0A025C3U5, A0A025C3V7, A0A025C3W2, A0A025C3X2, A0A025C3X5, A0A025C3X9, A0A025C3Z1, A0A025C401, A0A025C419, A0A025C431, A0A025C436, A0A025C439, A0A025C485, A0A025C486, A0A025C4M8, A0A025C4N4, A0A025C4Q1, A0A025C4R0, A0A025C4R3, A0A025C4R5, A0A025C4R9, A0A025C550, A0A025C578, A0A025C5C7, A0A025C5L1, A0A025C5R7, A0A025C5S0, A0A025C5S4, A0A025C609, A0A025C639, A0A025C6D5, A0A025C6F1, A0A025C6G1, A0A025C6G6, A0A025C6H1, A0A025C6H8, A0A025C6S1, A0A025C6T2, A0A025C6Z7, A0A025C734, A0A025C771, A0A025C796, A0A025C7B6, A0A025C7E3, A0A025C7N9, A0A025C7U4, A0A025C7U9, A0A025C7W1, A0A025C7Z2, A0A025C810, A0A025C816, A0A025C827, A0A025C842, A0A025C8E6, A0A025C8H4, A0A025C8I9, A0A025C8T2, A0A025C8U7, A0A025C8W0, A0A025C8W7, A0A025C8Y7, A0A025C971, A0A025C9E1, A0A025C9N5, A0A025C9N6, A0A025C9N9, A0A025C9P4, A0A025C9S5, A0A025C9V0, A0A025C9V2, A0A025C9V8, A0A025C9X1, A0A025C9X6, A0A025C9Y7, A0A025C9Z0, A0A025C9Z4, A0A025C9Z9, A0A025CA01, A0A025CA06, A0A025CA57, A0A025CAD9, A0A025CAI2, A0A025CAM1, A0A025CAR8, A0A025CAS5, A0A025CAT2, A0A025CAU8, A0A025CB42, A0A025CBA9, A0A025CBI5, A0A025CBM9, A0A025CBP3, A0A025CBW4, A0A025CBX1, A0A025CBZ0, A0A025CC87, A0A025CCC5, A0A025CCD2, A0A025CCG7, A0A025CCP0, A0A025CCU1, A0A025CCV8, A0A025CD30, A0A025CD35, A0A025CD45, A0A025CD70, A0A025CD73, A0A025CD79, A0A025CD95, A0A025CD96, A0A025CDB5, A0A025CDC9, A0A025CDQ9, A0A025CDW3, A0A025CDZ1, A0A025CE05, A0A025CE19, A0A025CE85, A0A025CED3, A0A025CEI8, A0A025CEJ8, A0A025CEP4, A0A025CEP9, A0A025CFH6, A0A025CFK6, A0A025CFL3, A0A025CFL6, A0A025CFM6, A0A025CFN5, A0A025CFN7, A0A025CFQ5, A0A025CFR7, A0A025CFS2, A0A025CFV8, A0A025CG52, A0A025CGC3, A0A025CGD7, A0A025CGE6, A0A025CGF1, A0A025CGF5, A0A025CGG8, A0A025CGH3, A0A025CGH9, A0A025CGK4, A0A025CGK6, A0A025CGL5, A0A025CGL6, A0A025CGM6, A0A025CGR3, A0A025CGR7, A0A025CGX4, A0A025CGY9, A0A025CGZ7, A0A025CH14, A0A025CH59, A0A025CHA6, A0A025CHC7, A0A025CHD1, A0A025CHE6, A0A025CHH4, A0A025CHH7, A0A025CHJ0, A0A025CHK7, A0A025CI83, A0A025CI91, A0A025CIA3, A0A025CID0, A0A025CID6, A0A025CIG6, A0A025CIK6, A0A025CIL3, A0A025CIM9, A0A025CIN0, A0A025CIP2, A0A025CIQ9, A0A025CIU7, A0A025CIX5, A0A025CIY6, A0A025CJ38, A0A025CJC1, A0A025CJI9, A0A025CJK2, A0A025CJX7, A0A025CK28, A0A025CK32, A0A025CK82, A0A025CKA1, A0A025CKA7, A0A025CKB6, A0A025CKC8, A0A025CKG5, A0A025CKI0, A0A025CKJ2, A0A025CKK0, A0A025CKK2, A0A025CKL1, A0A025CKL9, A0A025CKN4, A0A025CKN8, A0A025CKN9, A0A025CKQ7, A0A025CKS2, A0A025CKZ8, A0A025CL07, A0A025CL22, A0A025CL44, A0A025CL45, A0A025CL50, A0A025CL60, A0A025CL61, A0A025CL63, A0A025CL76, A0A025CL80, A0A025CL88, A0A025CL89, A0A025CL90, A0A025CL99, A0A025CLB0, A0A025CLD7, A0A025CLF1, A0A025CLF4, A0A025CLI6, A0A025CLM4, A0A025CLM9, A0A025CLU9, A0A025CM22, A0A025CM37, A0A025CM69, A0A025CM84, A0A025CMA0, A0A025CMB8, A0A025CMI2, A0A025CMU1, A0A025CMW6, A0A025CMZ0, A0A025CN66, A0A025CN98, A0A025CNA8, A0A025CNC7, A0A025CND1, A0A025CNE2, A0A025CNH3, A0A025CNH4, A0A025CNN4, A0A025CNN8, A0A025CNQ9, A0A025CP05, A0A025CP33, A0A025CP73, A0A025CP91, A0A025CPC1, A0A025CPC7, A0A025CPD0, A0A025CPD5, A0A025CPE6, A0A025CPG6, A0A025CPI4, A0A025CPL0, A0A025CPL8, A0A025CQ06, A0A025CQ07, A0A025CQ11, A0A025CQ19, A0A025CQ38, A0A025CQ42, A0A025CQ45, A0A025CQ78, A0A025CQ85, A0A025CQA7, A0A025CQD8, A0A025CQE0, A0A025CQG2, A0A025CR36, A0A025CR75, A0A025CR84, A0A025CR89, A0A025CRA5, A0A025CRE6, A0A025CRH3, A0A025CRK0, A0A025CRK2, A0A025CRK8, A0A025CRN7, A0A025CRQ7, A0A025CRS7, A0A025CRT7, A0A025CRW7, A0A025CRZ9, A0A025CS59, A0A025CS79, A0A025CSH2, A0A025CSH4, A0A025CSI1, A0A025CSI4, A0A025CSR2, A0A025CST1, A0A025CT22, A0A025CT37, A0A025CT49, A0A025CT82, A0A025CTF9, A0A025CU21, A0A025CU40, A0A025CU73, A0A025CU81, A0A025CU82, A0A025CU95, A0A025CUA3, A0A025CUE0, A0A025CUF3, A0A025CUH6, A0A025CV01, A0A025CV16, A0A025CV28, A0A025CV30, A0A025CV31, A0A025CV40, A0A025CV72, A0A025CV80, A0A025CV93, A0A025CVB4, A0A025CVD5, A0A025CVN1, A0A025CVR8, A0A025CVY0, A0A025CW13, A0A025CW29, A0A025CW39, A0A025CW47, A0A025CW57, A0A025CW69, A0A025CW77, A0A025CW81, A0A025CWC5, A0A025CWL7, A0A025CWS4, A0A025CWS8, A0A025CWV2, A0A025CX01, A0A025CX35, A0A025CYC3, A0A025CYD3, A0A025CYL4, A0A025CYQ4, A0A025CYQ8, A0A025CYR9, A0A025CYT7, A0A025CYU7, A0A025CYV8, A0A025CZ30, A0A025CZW1, A0A025D013, A0A025D189, A0A025D1A1, A0A025D1Q8, A0A025D1S3, A0A025D1U4, A0A025D203 |
| **O6:H1 (strain CFT073 / ATCC 700928 / UPEC)** | A0A0H2V455, A0A0H2V477, A0A0H2V482, A0A0H2V496, A0A0H2V4J1, A0A0H2V4J2, A0A0H2V4N5, A0A0H2V4S8, A0A0H2V4U9, A0A0H2V4V3, A0A0H2V4W0, A0A0H2V4Y5, A0A0H2V4Y9, A0A0H2V546, A0A0H2V574, A0A0H2V5B2, A0A0H2V5D4, A0A0H2V5F2, A0A0H2V5F3, A0A0H2V5R1, A0A0H2V5T0, A0A0H2V5U7, A0A0H2V5V0, A0A0H2V632, A0A0H2V648, A0A0H2V657, A0A0H2V661, A0A0H2V678, A0A0H2V6N3, A0A0H2V6V0, A0A0H2V6V8, A0A0H2V6X6, A0A0H2V700, A0A0H2V707, A0A0H2V708, A0A0H2V715, A0A0H2V755, A0A0H2V778, A0A0H2V794, A0A0H2V7A9, A0A0H2V7C3, A0A0H2V7F8, A0A0H2V7F9, A0A0H2V7U6, A0A0H2V7V5, A0A0H2V812, A0A0H2V842, A0A0H2V854, A0A0H2V887, A0A0H2V8B0, A0A0H2V8C0, A0A0H2V8D9, A0A0H2V8F0, A0A0H2V8I8, A0A0H2V8T8, A0A0H2V8W2, A0A0H2V8X9, A0A0H2V901, A0A0H2V908, A0A0H2V938, A0A0H2V945, A0A0H2V965, A0A0H2V975, A0A0H2V985, A0A0H2V9B3, A0A0H2V9B9, A0A0H2V9D8, A0A0H2V9G0, A0A0H2V9I2, A0A0H2V9K2, A0A0H2V9R7, A0A0H2V9S9, A0A0H2V9T0, A0A0H2V9Z3, A0A0H2VA53, A0A0H2VAM5, A0A0H2VAR2, A0A0H2VAW4, A0A0H2VAY0, A0A0H2VB19, A0A0H2VB35, A0A0H2VB45, A0A0H2VBD7, A0A0H2VBG4, A0A0H2VBH0, A0A0H2VBI1, A0A0H2VBJ6, A0A0H2VBK7, A0A0H2VBM6, A0A0H2VBN0, A0A0H2VBP4, A0A0H2VBS2, A0A0H2VBU9, A0A0H2VBW3, A0A0H2VBW7, A0A0H2VC41, A0A0H2VC51, A0A0H2VC93, A0A0H2VCG6, A0A0H2VCM0, A0A0H2VCR3, A0A0H2VCT3, A0A0H2VCU8, A0A0H2VCX0, A0A0H2VD40, A0A0H2VD85, A0A0H2VDA1, A0A0H2VDI0, A0A0H2VDI5, A0A0H2VDK3, A0A0H2VDP7, A0A0H2VDQ7, A0A0H2VDS2, A0A0H2VDV8, A0A0H2VDW3, A0A0H2VDW4, A0A0H2VE51, A0A0H2VE71, A0A0H2VE89, A0A0H2VE97, A0A0H2VEC9, A0A0H2VEJ7, A0A0H2VEU4, A0A0H2VF40, A0A0H2VFB5, A0A0H2VFD1, A0A0H2VFH8, A0A0H2VFI8, A0A0H2VFV6, A0A0H2VG66, A0A0H2VGC9, A0A0H2VGJ2, P0A3B1, P0A440, P0A4D0, P0A4L5, P0A6A7, P0A6M5, P0A6M9, P0A6N2, P0A6P2, P0A6U4, P0A6U6, P0A6X4, P0A708, P0A781, P0A785, P0A7B6, P0A7D2, P0A7I1, P0A7I5, P0A7J4, P0A7J8, P0A7K3, P0A7K7, P0A7L1, P0A7L4, P0A7L9, P0A7M3, P0A7M7, P0A7P0, P0A7P6, P0A7R2, P0A7R6, P0A7S0, P0A7S4, P0A7T0, P0A7T4, P0A7T8, P0A7U4, P0A7U8, P0A7V1, P0A7V4, P0A7V9, P0A7W2, P0A7W8, P0A7X4, P0A7X7, P0A7Y1, P0A7Y5, P0A7Z5, P0A801, P0A806, P0A833, P0A848, P0A874, P0A8D7, P0A8J9, P0A8L2, P0A8M1, P0A8M4, P0A8N4, P0A8N8, P0A8V3, P0A969, P0A973, P0A977, P0A979, P0A983, P0A987, P0A9H8, P0A9J1, P0A9P7, P0A9R8, P0A9U4, P0A9V2, P0A9V6, P0A9X2, P0A9Y0, P0A9Y7, P0AA11, P0AA44, P0AAF4, P0AAF7, P0AAG6, P0AAH1, P0AAH5, P0AAH9, P0AAL4, P0AAZ8, P0ABB5, P0ABF2, P0ABH1, P0ABI5, P0ABT6, P0ACG9, P0AD90, P0ADY4, P0ADY8, P0ADZ1, P0ADZ5, P0AE02, P0AEB8, P0AEK1, P0AF94, P0AFF7, P0AFQ6, P0AFR5, P0AG31, P0AG45, P0AG52, P0AG56, P0AG60, P0AG64, P0AG68, P0AG97, P0AGA0, P0AGA3, P0AGD8, P0AGJ8, P0AGK5, P0AGL3, P0AGL8, P0C019, P0C076, P0C0R9, P59587, P59664, P60341, P60423, P60439, P60724, P60758, P60786, P60907, P61176, P62396, P62400, P62523, P63178, P63355, P63387, P64294, P64424, P65834, P65844, P66023, P66071, P66606, P66679, P66681, P67025, P67030, P67036, P67588, P68680, P69875, P69914, Q8CXV3, Q8CXW3, Q8CXX6, Q8CXZ8, Q8FA88, Q8FAD1, Q8FAL3, Q8FAT5, Q8FAV1, Q8FB02, Q8FB30, Q8FB37, Q8FB47, Q8FB76, Q8FB83, Q8FBI8, Q8FBJ0, Q8FBS3, Q8FBV3, Q8FBV5, Q8FC87, Q8FCC1, Q8FCE2, Q8FCJ1, Q8FCM9, Q8FCN0, Q8FCQ2, Q8FCS8, Q8FCT7, Q8FD03, Q8FD12, Q8FD13, Q8FD17, Q8FD82, Q8FD87, Q8FDG5, Q8FDZ8, Q8FE69, Q8FEE5, Q8FEF9, Q8FEG6, Q8FEJ1, Q8FEJ7, Q8FEJ8, Q8FEL3, Q8FEY5, Q8FF14, Q8FF17, Q8FF24, Q8FF59, Q8FF64, Q8FFB3, Q8FFC9, Q8FFK8, Q8FFM1, Q8FFR2, Q8FFS1, Q8FFU7, Q8FFV5, Q8FFX8, Q8FG48, Q8FG49, Q8FGQ0, Q8FGQ9, Q8FGS9, Q8FGT3, Q8FH28, Q8FH88, Q8FH96, Q8FHP6, Q8FHR3, Q8FHT6, Q8FHV4, Q8FI03, Q8FIB6, Q8FIM7, Q8FIP7, Q8FJ88, Q8FJ95, Q8FJB1, Q8FJE7, Q8FJL0, Q8FJN1, Q8FJP8, Q8FJR4, Q8FJU5, Q8FJW4, Q8FJY9, Q8FK44, Q8FK84, Q8FKA7, Q8FKB7, Q8FKC5, Q8FKF5, Q8FKL1, Q8FKZ4, Q8FL00, Q8FL05, Q8FL06, Q8FL13, Q8FL25, Q8FL61, Q8FL80, Q8FL82, Q8FL92, Q8FL93, Q8FL96, Q8FLB7 |
| **O111:H- (strain 11128 / EHEC)** | C8UB58, C8UB73, C8UB74, C8UB83, C8UBA2, C8UBC8, C8UBG9, C8UBH0, C8UBH2, C8UBH3, C8UBH4, C8UBH5, C8UBL0, C8UBN3, C8UBQ2, C8UBQ6, C8UBQ7, C8UBS2, C8UBT1, C8UBT5, C8UBU4, C8UBW5, C8UBX2, C8UBY0, C8UC53, C8UC61, C8UC74, C8UC88, C8UCB3, C8UCI9, C8UCV6, C8UCW0, C8UCW9, C8UCX4, C8UCX5, C8UD48, C8UD61, C8UD71, C8UD78, C8UD80, C8UDB0, C8UDB3, C8UDB4, C8UDB5, C8UDE6, C8UDF0, C8UDF7, C8UDM3, C8UDQ7, C8UDR9, C8UDT1, C8UDX9, C8UDZ7, C8UE41, C8UE43, C8UE44, C8UE68, C8UE75, C8UE77, C8UE79, C8UE82, C8UEA0, C8UEA3, C8UEB5, C8UEC5, C8UED2, C8UED3, C8UED5, C8UEE1, C8UEE2, C8UEE6, C8UEF5, C8UEG6, C8UEG7, C8UEG8, C8UEG9, C8UEH0, C8UEH7, C8UEH9, C8UEN5, C8UEP8, C8UEP9, C8UER4, C8UET7, C8UEV1, C8UEV3, C8UEV9, C8UEW4, C8UEW5, C8UEW6, C8UEW7, C8UEW8, C8UEW9, C8UEX0, C8UEZ0, C8UEZ1, C8UEZ8, C8UF13, C8UF24, C8UF27, C8UF96, C8UF97, C8UF98, C8UFA6, C8UFD5, C8UFD6, C8UFE6, C8UFF1, C8UFG1, C8UFG2, C8UFL5, C8UFZ0, C8UFZ1, C8UFZ5, C8UFZ8, C8UG15, C8UG24, C8UG25, C8UG26, C8UG32, C8UG73, C8UGC1, C8UGC3, C8UGC4, C8UGC5, C8UGC7, C8UGC8, C8UGD3, C8UGD7, C8UGD8, C8UGE1, C8UGE3, C8UGE4, C8UGF3, C8UGF9, C8UGG0, C8UGI1, C8UGI2, C8UGJ7, C8UGL0, C8UGL8, C8UGM9, C8UGN3, C8UGN7, C8UGN8, C8UGP4, C8UGP5, C8UGP6, C8UGP7, C8UGP8, C8UGP9, C8UGS3, C8UGS8, C8UGS9, C8UGT6, C8UGT9, C8UGU3, C8UGW5, C8UGW8, C8UGY7, C8UGZ6, C8UH11, C8UH12, C8UH15, C8UH16, C8UH19, C8UH28, C8UH36, C8UH37, C8UH39, C8UH50, C8UH51, C8UH55, C8UH59, C8UH61, C8UH66, C8UH75, C8UH76, C8UHC0, C8UHE8, C8UHE9, C8UHF0, C8UHF1, C8UHF2, C8UHF3, C8UHF4, C8UHF5, C8UHF6, C8UHF7, C8UHF8, C8UHF9, C8UHG0, C8UHG1, C8UHG2, C8UHG3, C8UHG4, C8UHG5, C8UHG6, C8UHG7, C8UHG8, C8UHG9, C8UHH2, C8UHH3, C8UHH4, C8UHH5, C8UHI4, C8UHL5, C8UHN1, C8UHN8, C8UHP0, C8UHQ0, C8UHQ1, C8UHQ2, C8UHS4, C8UHT0, C8UHT4, C8UHT5, C8UHU3, C8UHU4, C8UHV9, C8UHW0, C8UHW5, C8UHX7, C8UI19, C8UI22, C8UI23, C8UI41, C8UI42, C8UI43, C8UI44, C8UI45, C8UI56, C8UI57, C8UI63, C8UI89, C8UIB0, C8UIB6, C8UIB8, C8UIF6, C8UII6, C8UII7, C8UIJ5, C8UIJ7, C8UIK4, C8UIM0, C8UIM9, C8UIN2, C8UIN3, C8UIQ8, C8UIR5, C8UIS4, C8UIS9, C8UIV5, C8UIX8, C8UIX9, C8UIZ6, C8UJ22, C8UJ23, C8UJ24, C8UJ30, C8UJ36, C8UJ38, C8UJ39, C8UJ59, C8UJ60, C8UJ91, C8UJ92, C8UJ94, C8UJ95, C8UJB0, C8UJB2, C8UJC3, C8UJD1, C8UJD2, C8UJE0, C8UJF0, C8UJG2, C8UJG5, C8UJJ7, C8UJK0, C8UJP8, C8UJP9, C8UJQ0, C8UJQ7, C8UJU0, C8UJV8, C8UJW8, C8UJY5, C8UK13, C8UK52, C8UK57, C8UK62, C8UK87, C8UK98, C8UKA0, C8UKB8, C8UKC1, C8UKC8, C8UKE4, C8UKJ5, C8UKJ6, C8UKM6, C8UKN2, C8UKN3, C8UKN5, C8UKN6, C8UKN7, C8UKN8, C8UKN9, C8UKP0, C8UKP7, C8UKS6, C8UKU0, C8UKZ7, C8UL12, C8UL15, C8UL17, C8UL27, C8UL45, C8UL54, C8UL79, C8UL83, C8UL88, C8ULA3, C8ULA4, C8ULA9, C8ULB0, C8ULB6, C8ULD4, C8ULD7, C8ULE0, C8ULE4, C8ULF3, C8ULF6, C8ULH1, C8ULH2, C8ULI4, C8ULL2, C8ULQ3, C8ULR3, C8ULU5, C8ULU6, C8ULV4, C8ULY4, C8ULY5, C8ULY7, C8UM07, C8UM08, C8UM14, C8UM15, C8UM16, C8UM20, C8UM22, C8UM23, C8UM29, C8UM76, C8UM77, C8UM80, C8UM93, C8UM97, C8UMC8, C8UMC9, C8UMG1, C8UML5, C8UMM6, C8UMP3, C8UMS2, C8UMV2, C8UMZ0, C8UMZ5, C8UN08, C8UN38, C8UN80, C8UN83, C8UN98, C8UNB0, C8UNE8, C8UNF0, C8UNF3, C8UNG7, C8UNH7, C8UNI0, C8UNQ6, C8UNR3, C8UNR5, C8UNV9, C8UNW0, C8UNW6, C8UNW7, C8UNW8, C8UNX2, C8UNX3, C8UNX4, C8UNY7, C8UNZ9, C8UP00, C8UP67, C8UP68, C8UP76, C8UP86, C8UP90, C8UP91, C8UPB6, C8UPE1, C8UPE2, C8UPE3, C8UPL1, C8UPL6, C8UPN5, C8UPS4, C8UPS5, C8UPT7, C8UPV9, C8UQ00, C8UQ01, C8UQC1, C8UQF6, C8UQG1, C8UQU5, C8UR41, C8UR44, C8UR96, C8URC0, C8URF9, C8URI1, C8URJ3, C8URK6, C8URK7 |
| **O127:H6 (strain E2348/69 / EPEC)** | B7UFD1, B7UFE6, B7UFF6, B7UFG3, B7UFG5, B7UFJ7, B7UFK0, B7UFK1, B7UFK2, B7UFL4, B7UFL8, B7UFM5, B7UFR7, B7UFT1, B7UFW4, B7UFX6, B7UFY8, B7UGA5, B7UGC2, B7UGD7, B7UGM4, B7UGN8, B7UGU3, B7UGU5, B7UGU7, B7UGV7, B7UGW0, B7UGX8, B7UGY1, B7UGY7, B7UGZ9, B7UH06, B7UH07, B7UH09, B7UH15, B7UH16, B7UH20, B7UH29, B7UH55, B7UH56, B7UH57, B7UH58, B7UH59, B7UH66, B7UH68, B7UH97, B7UHA6, B7UHA7, B7UHB4, B7UHB5, B7UHE9, B7UHG2, B7UHG4, B7UHH0, B7UHJ8, B7UHJ9, B7UHK1, B7UHK7, B7UHM2, B7UHN3, B7UHN6, B7UHT9, B7UHU0, B7UHU1, B7UHU9, B7UHX3, B7UHZ2, B7UHZ3, B7UI61, B7UI62, B7UI68, B7UI71, B7UI99, B7UIA4, B7UIA5, B7UIB7, B7UIC0, B7UIC2, B7UIE4, B7UIE8, B7UIG6, B7UIH5, B7UII7, B7UII8, B7UIJ1, B7UIJ2, B7UIJ8, B7UIK7, B7UIL4, B7UIL5, B7UIL7, B7UIT8, B7UIW4, B7UIX3, B7UIX4, B7UIX5, B7UIY1, B7UJ17, B7UJ57, B7UJ59, B7UJ60, B7UJ61, B7UJ63, B7UJ64, B7UJ68, B7UJ72, B7UJ73, B7UJ76, B7UJ78, B7UJ79, B7UJ84, B7UJ85, B7UJ89, B7UJ93, B7UJ96, B7UJA1, B7UJB0, B7UJB1, B7UJC3, B7UJC4, B7UJD0, B7UJE4, B7UJH1, B7UJJ6, B7UJM8, B7UJM9, B7UJN7, B7UJN9, B7UJP6, B7UJQ9, B7UJR8, B7UJS1, B7UJS2, B7UJT5, B7UJU1, B7UJU2, B7UJW2, B7UJW3, B7UJX9, B7UJZ1, B7UK02, B7UK06, B7UK11, B7UK12, B7UK18, B7UK19, B7UK20, B7UK21, B7UK22, B7UK23, B7UK24, B7UK25, B7UK26, B7UK27, B7UK28, B7UK29, B7UK30, B7UK31, B7UK32, B7UK33, B7UK34, B7UK35, B7UK36, B7UK37, B7UK38, B7UK39, B7UK40, B7UK41, B7UK42, B7UK43, B7UK44, B7UK45, B7UK49, B7UK50, B7UK51, B7UK52, B7UK63, B7UK89, B7UKA4, B7UKB2, B7UKB4, B7UKC4, B7UKC5, B7UKC6, B7UKF4, B7UKG1, B7UKH3, B7UKH8, B7UKK3, B7UKM4, B7UKM5, B7UKN1, B7UKR5, B7UKT2, B7UKT5, B7UKW1, B7UL01, B7UL05, B7UL06, B7UL14, B7UL15, B7UL31, B7UL32, B7UL37, B7UL47, B7UL60, B7UL93, B7UL94, B7UL99, B7ULA0, B7ULB6, B7ULB7, B7ULB8, B7ULB9, B7ULC0, B7ULC8, B7ULE9, B7ULF0, B7ULG2, B7ULJ0, B7ULJ1, B7ULJ2, B7ULJ5, B7ULN3, B7ULN8, B7ULP4, B7ULR6, B7ULX6, B7ULZ1, B7ULZ4, B7ULZ5, B7UM05, B7UM16, B7UM24, B7UM48, B7UM50, B7UM71, B7UM73, B7UM74, B7UM85, B7UMA6, B7UME4, B7UMH0, B7UMH1, B7UMH3, B7UMH4, B7UMI8, B7UMJ0, B7UMJ7, B7UMK5, B7UMK6, B7UML4, B7UMM4, B7UMN3, B7UMN5, B7UMN8, B7UMU7, B7UMV1, B7UMV6, B7UMX1, B7UMX2, B7UMX7, B7UMX8, B7UMY4, B7UMZ7, B7UN00, B7UN03, B7UN07, B7UN16, B7UN19, B7UN27, B7UN28, B7UN66, B7UN67, B7UND3, B7UND9, B7UNG3, B7UNG5, B7UNI6, B7UNK4, B7UNQ5, B7UNQ6, B7UNU5, B7UNZ3, B7UP60, B7UPA0, B7UPA2, B7UPA5, B7UPB9, B7UPD0, B7UPD3, B7UPD5, B7UPD6, B7UPD8, B7UPD9, B7UPE0, B7UPE1, B7UPE2, B7UPE3, B7UPF0, B7UPI3, B7UPJ6, B7UPK8, B7UPM7, B7UPP7, B7UPP8, B7UPR1, B7UPS0, B7UPS1, B7UPS9, B7UPV1, B7UPX7, B7UPY1, B7UPY2, B7UQ35, B7UQ42, B7UQ44, B7UQ82, B7UQ83, B7UQ91, B7UQ92, B7UQ98, B7UQ99, B7UQA0, B7UQA4, B7UQC4, B7UQD6, B7UQD7, B7UQG6, B7UQG7, B7UQH6, B7UQI0, B7UQI1, B7UQI2, B7UQI8, B7UQI9, B7UQK9, B7UQL1, B7UQL2, B7UQN9, B7UQQ3, B7UQQ7, B7UQR5, B7UQS6, B7UQU1, B7UQW9, B7UQX7, B7UQZ9, B7UR02, B7UR21, B7UR73, B7UR75, B7UR84, B7UR96, B7URA5, B7URA6, B7URC6, B7URE7, B7URE8, B7URE9, B7URG2, B7URI7, B7URL5, B7URM9, B7URP3, B7URW9, B7URX8, B7URZ3, B7US02, B7US21, B7US48, B7US96, B7US97, B7US99, B7USA0, B7USA1, B7USA2, B7USD7, B7USG7, B7USH9, B7USI3, B7USI4, B7USJ9, B7USK8, B7USL2, B7USM1, B7USN5, B7USP3, B7USQ3, B7USR0, B7USS2, B7UST6, B7USV5, B7UT49, B7UT56, B7UT61, B7UT62, B7UTG9, B7UTH5, B7UTI3, B7UTI5, B7UTI9, B7UTJ0, P0A3B2, P0A4L6 |
| **O145:H28 str. RM12581** | A0A023YRI6, A0A023YRM4, A0A023YRP0, A0A023YRS2, A0A023YRT5, A0A023YRT8, A0A023YRU8, A0A023YRW4, A0A023YRW5, A0A023YRW7, A0A023YS03, A0A023YS05, A0A023YS11, A0A023YS12, A0A023YS16, A0A023YS21, A0A023YS34, A0A023YS49, A0A023YS70, A0A023YS77, A0A023YS85, A0A023YS92, A0A023YS97, A0A023YSA1, A0A023YSA2, A0A023YSB7, A0A023YSC3, A0A023YSD9, A0A023YSE2, A0A023YSE5, A0A023YSF5, A0A023YSG8, A0A023YSI6, A0A023YSK7, A0A023YSL8, A0A023YSM6, A0A023YSN4, A0A023YSN6, A0A023YSP6, A0A023YSR8, A0A023YST7, A0A023YSU7, A0A023YSV2, A0A023YSV5, A0A023YSW3, A0A023YSW8, A0A023YSW9, A0A023YSY5, A0A023YT00, A0A023YT07, A0A023YT09, A0A023YT43, A0A023YT49, A0A023YT74, A0A023YT84, A0A023YTB5, A0A023YTF1, A0A023YTF4, A0A023YTH5, A0A023YTK6, A0A023YTU2, A0A023YTU7, A0A023YTV7, A0A023YTY0, A0A023YU17, A0A023YU19, A0A023YU34, A0A023YU38, A0A023YU57, A0A023YU60, A0A023YU92, A0A023YUA1, A0A023YUA3, A0A023YUA8, A0A023YUC9, A0A023YUD1, A0A023YUE4, A0A023YUE9, A0A023YUF1, A0A023YUH0, A0A023YUH9, A0A023YUI5, A0A023YUI7, A0A023YUJ2, A0A023YUK5, A0A023YUK6, A0A023YUM6, A0A023YUQ2, A0A023YUY2, A0A023YV47, A0A023YV55, A0A023YV62, A0A023YV82, A0A023YV86, A0A023YV99, A0A023YVC0, A0A023YVF1, A0A023YVH0, A0A023YVJ1, A0A023YVL7, A0A023YVL8, A0A023YVQ1, A0A023YVS0, A0A023YVU0, A0A023YVV5, A0A023YW16, A0A023YW19, A0A023YW23, A0A023YW37, A0A023YW78, A0A023YW94, A0A023YWB0, A0A023YWB5, A0A023YWC5, A0A023YWE0, A0A023YWE7, A0A023YWK3, A0A023YWL5, A0A023YWM3, A0A023YWN2, A0A023YWN6, A0A023YWN7, A0A023YWS6, A0A023YWS8, A0A023YWU9, A0A023YWV5, A0A023YWX6, A0A023YX25, A0A023YX44, A0A023YX48, A0A023YX61, A0A023YX63, A0A023YXB1, A0A023YXB4, A0A023YXC1, A0A023YXC2, A0A023YXK6, A0A023YXL1, A0A023YXL2, A0A023YXL6, A0A023YXM3, A0A023YXP3, A0A023YXW5, A0A023YXW9, A0A023YXX6, A0A023YXY1, A0A023YY12, A0A023YY35, A0A023YY36, A0A023YY40, A0A023YY43, A0A023YY48, A0A023YY57, A0A023YY75, A0A023YY77, A0A023YY87, A0A023YYA2, A0A023YYB6, A0A023YYH0, A0A023YYI0, A0A023YYL3, A0A023YYR9, A0A023YYS7, A0A023YYW1, A0A023YYX4, A0A023YYY4, A0A023YZ33, A0A023YZD4, A0A023YZE6, A0A023YZF1, A0A023YZG3, A0A023YZL3, A0A023YZP0, A0A023YZS4, A0A023YZT5, A0A023YZX8, A0A023YZY3, A0A023YZY8, A0A023YZY9, A0A023Z003, A0A023Z031, A0A023Z033, A0A023Z039, A0A023Z048, A0A023Z053, A0A023Z084, A0A023Z0B8, A0A023Z0G0, A0A023Z0G6, A0A023Z0I4, A0A023Z0J2, A0A023Z0K1, A0A023Z0N1, A0A023Z0S6, A0A023Z0S7, A0A023Z0Z3, A0A023Z129, A0A023Z140, A0A023Z149, A0A023Z150, A0A023Z157, A0A023Z167, A0A023Z172, A0A023Z192, A0A023Z1A4, A0A023Z1B0, A0A023Z1B4, A0A023Z1D0, A0A023Z1D9, A0A023Z1F5, A0A023Z1G3, A0A023Z1H0, A0A023Z1H2, A0A023Z1H8, A0A023Z1I4, A0A023Z1I6, A0A023Z1J4, A0A023Z1K1, A0A023Z1L3, A0A023Z1N6, A0A023Z1P2, A0A023Z1T1, A0A023Z1T2, A0A023Z1U9, A0A023Z1V6, A0A023Z1W0, A0A023Z1W9, A0A023Z1X5, A0A023Z1Y3, A0A023Z1Z5, A0A023Z214, A0A023Z245, A0A023Z256, A0A023Z262, A0A023Z273, A0A023Z284, A0A023Z291, A0A023Z2C0, A0A023Z2E3, A0A023Z2E9, A0A023Z2G5, A0A023Z2H3, A0A023Z2I2, A0A023Z2K2, A0A023Z2K3, A0A023Z2M5, A0A023Z2N4, A0A023Z2Q4, A0A023Z2R0, A0A023Z2R9, A0A023Z2U5, A0A023Z2Y1, A0A023Z2Y7, A0A023Z2Z0, A0A023Z310, A0A023Z314, A0A023Z328, A0A023Z329, A0A023Z336, A0A023Z342, A0A023Z345, A0A023Z367, A0A023Z374, A0A023Z377, A0A023Z3C5, A0A023Z3D0, A0A023Z3D6, A0A023Z3D8, A0A023Z3E1, A0A023Z3E3, A0A023Z3E8, A0A023Z3E9, A0A023Z3F3, A0A023Z3F8, A0A023Z3G4, A0A023Z3G5, A0A023Z3G6, A0A023Z3G9, A0A023Z3H1, A0A023Z3H4, A0A023Z3H8, A0A023Z3I0, A0A023Z3I4, A0A023Z3I5, A0A023Z3I8, A0A023Z3J1, A0A023Z3J2, A0A023Z3J6, A0A023Z3L8, A0A023Z3M5, A0A023Z3N5, A0A023Z3N6, A0A023Z3N8, A0A023Z3P4, A0A023Z3Q1, A0A023Z3Q7, A0A023Z3R2, A0A023Z3R3, A0A023Z3R4, A0A023Z3R6, A0A023Z3R7, A0A023Z3S8, A0A023Z3T3, A0A023Z3T6, A0A023Z3U0, A0A023Z3U1, A0A023Z3V3, A0A023Z3V7, A0A023Z3V8, A0A023Z3W4, A0A023Z3W8, A0A023Z3X3, A0A023Z3X6, A0A023Z3Y4, A0A023Z401, A0A023Z410, A0A023Z412, A0A023Z439, A0A023Z482, A0A023Z495, A0A023Z4A1, A0A023Z4A2, A0A023Z4A3, A0A023Z4B4, A0A023Z4B9, A0A023Z4C0, A0A023Z4C1, A0A023Z4C2, A0A023Z4C4, A0A023Z4C6, A0A023Z4D1, A0A023Z4D7, A0A023Z4E4, A0A023Z4E8, A0A023Z4G0, A0A023Z4G1, A0A023Z4H7, A0A023Z4I4, A0A023Z4J8, A0A023Z4K7, A0A023Z4L4, A0A023Z4L6, A0A023Z4N5, A0A023Z4N6, A0A023Z4Q1, A0A023Z4Q4, A0A023Z4R8, A0A023Z4V8, A0A023Z4V9, A0A023Z4W2, A0A023Z4Y2, A0A023Z4Z0, A0A023Z502, A0A023Z506, A0A023Z511, A0A023Z513, A0A023Z520, A0A023Z529, A0A023Z532, A0A023Z563, A0A023Z570, A0A023Z584, A0A023Z593, A0A023Z5A2, A0A023Z5B3, A0A023Z5C6, A0A023Z5D1, A0A023Z5D2, A0A023Z5E8, A0A023Z5F7, A0A023Z5G8, A0A023Z5G9, A0A023Z5H4, A0A023Z5P1, A0A023Z5P9, A0A023Z5S2, A0A023Z5S6, A0A023Z5T0, A0A023Z5U0, A0A023Z5U6, A0A023Z5V5, A0A023Z5V7, A0A023Z5V8, A0A023Z5Z3, A0A023Z600, A0A023Z635, A0A023Z640, A0A023Z650, A0A023Z651, A0A023Z657, A0A023Z681, A0A023Z688, A0A023Z689, A0A023Z692, A0A023Z693, A0A023Z6A7, A0A023Z6B7, A0A023Z6M3, A0A023Z6P6, A0A023Z6R1, A0A023Z6R3, A0A023Z6S9, A0A023Z6T1, A0A023Z6T6, A0A023Z6V6, A0A023Z6W4, A0A023Z6X5, A0A023Z6Y3, A0A023Z6Y5, A0A023Z6Y7, A0A023Z6Z6, A0A023Z713, A0A023Z716, A0A023Z723, A0A023Z725, A0A023Z738, A0A023Z742, A0A023Z746, A0A023Z7C8, A0A023Z7G4, A0A023Z7K5, A0A023Z7L2, A0A023Z7M0, A0A023Z7N5, A0A023Z7N7, A0A023Z7R1, A0A023Z7R6, A0A023Z7V3, A0A023Z7X5, A0A023Z817, A0A023Z876, A0A023Z897, A0A023Z8B1 |
| **strain UTI89 / UPEC** | P0C203, Q1R1P6, Q1R1U0, Q1R1X7, Q1R201, Q1R223, Q1R238, Q1R250, Q1R270, Q1R2A9, Q1R2D3, Q1R2F8, Q1R2G2, Q1R2T6, Q1R2Y1, Q1R2Z6, Q1R312, Q1R327, Q1R357, Q1R358, Q1R360, Q1R380, Q1R381, Q1R387, Q1R388, Q1R389, Q1R393, Q1R395, Q1R396, Q1R3A2, Q1R3D0, Q1R3F6, Q1R3G4, Q1R3G5, Q1R3H4, Q1R3I5, Q1R3I6, Q1R3L2, Q1R3N9, Q1R3Q1, Q1R3R6, Q1R3U2, Q1R3Y2, Q1R431, Q1R442, Q1R450, Q1R455, Q1R475, Q1R477, Q1R4B1, Q1R4B8, Q1R4F5, Q1R4F9, Q1R4I3, Q1R4J1, Q1R4J2, Q1R4K2, Q1R4L0, Q1R4L3, Q1R4M7, Q1R4M8, Q1R4N2, Q1R4N3, Q1R4S8, Q1R4T9, Q1R4U0, Q1R4U2, Q1R4U9, Q1R4V5, Q1R4V6, Q1R4V8, Q1R4Y9, Q1R503, Q1R504, Q1R528, Q1R536, Q1R537, Q1R538, Q1R539, Q1R541, Q1R556, Q1R557, Q1R560, Q1R562, Q1R564, Q1R597, Q1R5B7, Q1R5C8, Q1R5D8, Q1R5D9, Q1R5F6, Q1R5F7, Q1R5H3, Q1R5H4, Q1R5H8, Q1R5L3, Q1R5L4, Q1R5L5, Q1R5M5, Q1R5M7, Q1R5N7, Q1R5Q3, Q1R5T0, Q1R5U1, Q1R5U2, Q1R5U3, Q1R5U4, Q1R5U5, Q1R5U7, Q1R5U9, Q1R5V0, Q1R5V1, Q1R5V3, Q1R5V4, Q1R5W1, Q1R5Y2, Q1R601, Q1R602, Q1R604, Q1R606, Q1R607, Q1R609, Q1R610, Q1R612, Q1R613, Q1R615, Q1R616, Q1R617, Q1R619, Q1R620, Q1R621, Q1R622, Q1R624, Q1R626, Q1R627, Q1R629, Q1R630, Q1R631, Q1R632, Q1R633, Q1R635, Q1R636, Q1R637, Q1R638, Q1R644, Q1R645, Q1R650, Q1R657, Q1R668, Q1R677, Q1R693, Q1R6A9, Q1R6B0, Q1R6D2, Q1R6D3, Q1R6D9, Q1R6F0, Q1R6F2, Q1R6F4, Q1R6F7, Q1R6F9, Q1R6G3, Q1R6G8, Q1R6H0, Q1R6H2, Q1R6H3, Q1R6H4, Q1R6H8, Q1R6M0, Q1R6Q5, Q1R6R2, Q1R6R4, Q1R6R5, Q1R6S5, Q1R6V5, Q1R743, Q1R781, Q1R782, Q1R7A1, Q1R7D0, Q1R7D9, Q1R7E1, Q1R7J5, Q1R7J9, Q1R7N4, Q1R7Q1, Q1R7Q7, Q1R7Q9, Q1R7R1, Q1R7T7, Q1R7U0, Q1R7U6, Q1R7U8, Q1R7W4, Q1R7Y8, Q1R804, Q1R805, Q1R810, Q1R811, Q1R821, Q1R863, Q1R8A4, Q1R8A6, Q1R8B5, Q1R8B6, Q1R8B7, Q1R8B8, Q1R8B9, Q1R8D1, Q1R8F1, Q1R8F6, Q1R8F7, Q1R8G3, Q1R8G6, Q1R8G7, Q1R8H5, Q1R8I8, Q1R8J7, Q1R8K0, Q1R8L9, Q1R8M2, Q1R8M7, Q1R8M9, Q1R8N8, Q1R8Q3, Q1R8U9, Q1R8W9, Q1R906, Q1R957, Q1R982, Q1R994, Q1R9A7, Q1R9E5, Q1R9G0, Q1R9K6, Q1R9L4, Q1R9L8, Q1R9N4, Q1R9N5, Q1R9N6, Q1R9N9, Q1R9S4, Q1R9S6, Q1R9T3, Q1R9U4, Q1R9V8, Q1RA48, Q1RA49, Q1RA54, Q1RA61, Q1RA83, Q1RAG2, Q1RAG3, Q1RAK0, Q1RAM3, Q1RAN8, Q1RAQ0, Q1RAQ8, Q1RAR8, Q1RAS6, Q1RAU2, Q1RAV2, Q1RAV6, Q1RAW7, Q1RAY4, Q1RAY6, Q1RAZ0, Q1RB05, Q1RB36, Q1RB76, Q1RB77, Q1RB78, Q1RB79, Q1RB81, Q1RB82, Q1RB86, Q1RBB4, Q1RBD4, Q1RBE3, Q1RBF8, Q1RBG8, Q1RBP8, Q1RBS6, Q1RBX6, Q1RBZ5, Q1RC01, Q1RC21, Q1RC22, Q1RC23, Q1RC47, Q1RC68, Q1RC69, Q1RC77, Q1RC89, Q1RC99, Q1RCA1, Q1RCG0, Q1RCI9, Q1RCJ0, Q1RCK6, Q1RCM6, Q1RCM7, Q1RCM8, Q1RCN4, Q1RCN5, Q1RCP6, Q1RCP7, Q1RCU4, Q1RD11, Q1RD18, Q1RD20, Q1RD28, Q1RD37, Q1RD40, Q1RD51, Q1RD68, Q1RD71, Q1RD73, Q1RDB4, Q1RDF9, Q1RDK7, Q1RDM9, Q1RDN0, Q1RDR5, Q1RDR6, Q1RDS4, Q1RDS7, Q1RDT6, Q1RDU1, Q1RDU4, Q1RDU7, Q1RE19, Q1RE21, Q1RE27, Q1RE28, Q1RE43, Q1RE44, Q1RE61, Q1RE66, Q1RE70, Q1RE96, Q1REA4, Q1REB6, Q1REC6, Q1RED0, Q1RED3, Q1REF1, Q1REF9, Q1REG5, Q1REH1, Q1REK9, Q1REN7, Q1RER6, Q1RER9, Q1RET7, Q1REX4, Q1RF08, Q1RF33, Q1RF38, Q1RF52, Q1RF61, Q1RF86, Q1RF87, Q1RF90, Q1RFA0, Q1RFB7, Q1RFC4, Q1RFC7, Q1RFD5, Q1RFD6, Q1RFH8, Q1RFK7, Q1RFK9, Q1RFL5, Q1RFQ0, Q1RFS8, Q1RFT5, Q1RFT6, Q1RFT8, Q1RFX6, Q1RFX7, Q1RFY9, Q1RFZ4, Q1RFZ7, Q1RG01, Q1RG05, Q1RG06, Q1RG17, Q1RG19, Q1RG21, Q1RG28, Q1RG37, Q1RG42, Q1RG43, Q1RG46, Q1RG47, Q1RG64, Q1RG73, Q1RG83, Q1RG98, Q1RGA1, Q1RGC5, Q1RGC7, Q1RGD0, Q1RGE0, Q1RGE1, Q1RGE6, Q1RGH6, Q1RGH9, Q1RGI6 |
| **O157:H7 (strain EC4115 / EHEC)** | ACI34452, ACI34454, ACI34484, ACI34495, ACI34532, ACI34546, ACI34568, ACI34590, ACI34611, ACI34621, ACI34675, ACI34676, ACI34684, ACI34692, ACI34695, ACI34717, ACI34733, ACI34734, ACI34743, ACI34752, ACI34762, ACI34771, ACI34775, ACI34806, ACI34828, ACI34829, ACI34834, ACI34840, ACI34859, ACI34864, ACI34873, ACI34917, ACI34931, ACI34958, ACI34965, ACI34975, ACI34995, ACI35001, ACI35007, ACI35008, ACI35009, ACI35040, ACI35058, ACI35065, ACI35079, ACI35083, ACI35095, ACI35112, ACI35118, ACI35133, ACI35140, ACI35170, ACI35188, ACI35192, ACI35197, ACI35203, ACI35217, ACI35227, ACI35251, ACI35269, ACI35282, ACI35286, ACI35295, ACI35315, ACI35323, ACI35356, ACI35360, ACI35375, ACI35388, ACI35393, ACI35405, ACI35408, ACI35414, ACI35429, ACI35442, ACI35447, ACI35462, ACI35468, ACI35501, ACI35506, ACI35529, ACI35544, ACI35555, ACI35595, ACI35607, ACI35621, ACI35624, ACI35647, ACI35653, ACI35660, ACI35676, ACI35692, ACI35720, ACI35742, ACI35753, ACI35755, ACI35807, ACI35828, ACI35839, ACI35843, ACI35848, ACI35854, ACI35859, ACI35865, ACI35876, ACI35903, ACI35904, ACI35922, ACI35942, ACI35949, ACI35961, ACI35962, ACI35994, ACI35997, ACI36031, ACI36077, ACI36079, ACI36080, ACI36109, ACI36115, ACI36123, ACI36125, ACI36136, ACI36148, ACI36168, ACI36181, ACI36182, ACI36203, ACI36211, ACI36212, ACI36216, ACI36240, ACI36258, ACI36269, ACI36305, ACI36320, ACI36339, ACI36341, ACI36344, ACI36354, ACI36392, ACI36399, ACI36422, ACI36429, ACI36436, ACI36449, ACI36473, ACI36534, ACI36541, ACI36543, ACI36552, ACI36556, ACI36559, ACI36562, ACI36572, ACI36599, ACI36609, ACI36620, ACI36622, ACI36671, ACI36677, ACI36684, ACI36689, ACI36716, ACI36718, ACI36738, ACI36761, ACI36774, ACI36781, ACI36782, ACI36802, ACI36811, ACI36812, ACI36826, ACI36835, ACI36838, ACI36845, ACI36865, ACI36869, ACI36879, ACI36914, ACI36920, ACI36925, ACI36931, ACI36932, ACI36949, ACI36951, ACI36981, ACI36998, ACI36999, ACI37017, ACI37107, ACI37117, ACI37121, ACI37136, ACI37143, ACI37173, ACI37185, ACI37196, ACI37200, ACI37201, ACI37237, ACI37253, ACI37270, ACI37310, ACI37314, ACI37343, ACI37346, ACI37364, ACI37370, ACI37376, ACI37380, ACI37382, ACI37384, ACI37406, ACI37418, ACI37453, ACI37462, ACI37471, ACI37473, ACI37484, ACI37486, ACI37488, ACI37495, ACI37499, ACI37550, ACI37560, ACI37564, ACI37566, ACI37585, ACI37598, ACI37616, ACI37617, ACI37624, ACI37625, ACI37653, ACI37655, ACI37656, ACI37658, ACI37671, ACI37696, ACI37700, ACI37701, ACI37723, ACI37751, ACI37753, ACI37755, ACI37757, ACI37770, ACI37771, ACI37776, ACI37777, ACI37784, ACI37789, ACI37815, ACI37826, ACI37839, ACI37876, ACI37883, ACI37894, ACI37906, ACI37933, ACI37948, ACI37980, ACI37992, ACI38001, ACI38012, ACI38015, ACI38025, ACI38048, ACI38050, ACI38051, ACI38056, ACI38066, ACI38089, ACI38095, ACI38106, ACI38111, ACI38121, ACI38125, ACI38139, ACI38162, ACI38165, ACI38168, ACI38188, ACI38202, ACI38207, ACI38219, ACI38224, ACI38234, ACI38237, ACI38279, ACI38285, ACI38289, ACI38297, ACI38300, ACI38303, ACI38320, ACI38323, ACI38339, ACI38343, ACI38344, ACI38352, ACI38372, ACI38405, ACI38441, ACI38446, ACI38453, ACI38497, ACI38511, ACI38524, ACI38525, ACI38537, ACI38539, ACI38542, ACI38545, ACI38550, ACI38560, ACI38648, ACI38650, ACI38680, ACI38718, ACI38737, ACI38740, ACI38744, ACI38753, ACI38783, ACI38835, ACI38838, ACI38840, ACI38846, ACI38861, ACI38865, ACI38873, ACI38879, ACI38891, ACI38898, ACI38922, ACI38927, ACI38932, ACI38945, ACI38986, ACI39004, ACI39005, ACI39036, ACI39042, ACI39043, ACI39064, ACI39076, ACI39082, ACI39083, ACI39087, ACI39101, ACI39107, ACI39120, ACI39129, ACI39130, ACI39134, ACI39147, ACI39168, ACI39182, ACI39198, ACI39214, ACI39223, ACI39235, ACI39262, ACI39266, ACI39271, ACI39276, ACI39303, ACI39328, ACI39338, ACI39360, ACI39382, ACI39388, ACI39417, ACI39418, ACI39423, ACI39430, ACI39440, ACI39447, ACI39448, ACI39480, ACI39484, ACI39491, ACI39502, ACI39520, ACI39551, ACI39601, ACI39612, ACI39613, ACI39620, ACI39621, ACI39649, ACI39651, ACI39652, ACI39695, ACI39724, ACI39727, ACI39745, ACI39751, ACI39765, ACI39780, ACI39782, ACI39787, ACI39790, ACI39804, ACI39805, ACI39806, ACI39815, ACI39825, ACI39834, ACI39873 |
| **O78:H11 (strain H10407 / ETEC)** | D0Z6Q1, D0Z6R2, E3PAG1, E3PAG9, E3PAH9, E3PAI7, E3PAK0, E3PAU2, E3PAV6, E3PAW2, E3PAX3, E3PAX5, E3PAZ3, E3PAZ6, E3PB03, E3PB20, E3PB67, E3PB68, E3PBA6, E3PBB3, E3PBB6, E3PBC7, E3PBG1, E3PBG2, E3PBK0, E3PBN7, E3PBP6, E3PBQ1, E3PBQ2, E3PBU2, E3PBU8, E3PBU9, E3PBV1, E3PBV2, E3PBV3, E3PBV4, E3PBV5, E3PBV6, E3PBW4, E3PBY7, E3PC00, E3PC34, E3PC69, E3PC77, E3PCA5, E3PCB1, E3PCB2, E3PCB9, E3PCC2, E3PCC5, E3PCE6, E3PCE9, E3PCF0, E3PCG8, E3PCH7, E3PCJ3, E3PCJ4, E3PCJ7, E3PCJ8, E3PCK1, E3PCQ8, E3PCS0, E3PCT0, E3PCT7, E3PCT9, E3PCX2, E3PCX5, E3PCX6, E3PCX7, E3PD07, E3PD16, E3PD17, E3PD25, E3PD51, E3PD74, E3PD89, E3PDB3, E3PDC1, E3PDC2, E3PDC4, E3PDD5, E3PDD6, E3PDE0, E3PDE4, E3PDE7, E3PDF2, E3PDG1, E3PDG2, E3PDL2, E3PDL4, E3PDM0, E3PDN8, E3PDQ3, E3PDQ7, E3PDR3, E3PDV7, E3PDX1, E3PE11, E3PE26, E3PE32, E3PE33, E3PE35, E3PE39, E3PE40, E3PE41, E3PE47, E3PE48, E3PE68, E3PE70, E3PE71, E3PE99, E3PEB3, E3PEB7, E3PEC6, E3PEM6, E3PEP5, E3PEU1, E3PEU3, E3PEV4, E3PF04, E3PF23, E3PF74, E3PFA2, E3PFF7, E3PFH3, E3PFI5, E3PFM2, E3PFM3, E3PFN1, E3PFN3, E3PFP0, E3PFQ2, E3PFR1, E3PFR4, E3PFR5, E3PFT9, E3PFU6, E3PFV5, E3PFW0, E3PFW4, E3PFZ0, E3PG48, E3PG51, E3PG53, E3PG55, E3PG58, E3PG76, E3PG79, E3PG91, E3PGA3, E3PGF8, E3PGI9, E3PGJ7, E3PGN2, E3PGQ0, E3PGR1, E3PGV6, E3PGV7, E3PGV9, E3PGW5, E3PGW6, E3PGX1, E3PGY0, E3PGZ0, E3PGZ1, E3PGZ2, E3PGZ3, E3PGZ4, E3PH01, E3PH03, E3PH47, E3PH60, E3PH61, E3PH74, E3PHA3, E3PHE2, E3PHE7, E3PHF6, E3PHL9, E3PHP0, E3PHQ0, E3PHQ2, E3PHQ8, E3PHR3, E3PHR4, E3PHR5, E3PHR6, E3PHR7, E3PHT9, E3PHU0, E3PHU2, E3PHU8, E3PHW3, E3PHX3, E3PI07, E3PI24, E3PI27, E3PI29, E3PI39, E3PI50, E3PI59, E3PI85, E3PI89, E3PI94, E3PIB0, E3PIG6, E3PIG7, E3PIG8, E3PIH6, E3PIL7, E3PIL8, E3PIP7, E3PIQ7, E3PIQ8, E3PIR4, E3PIT2, E3PIT5, E3PIT8, E3PIU2, E3PIV1, E3PIV4, E3PIX2, E3PIX3, E3PIY6, E3PJ11, E3PJ12, E3PJ62, E3PJE1, E3PJE2, E3PJH9, E3PJK8, E3PJP8, E3PJQ0, E3PJQ3, E3PJR7, E3PJS7, E3PJT0, E3PJX1, E3PJY0, E3PJY1, E3PJY2, E3PJY8, E3PK25, E3PK74, E3PK76, E3PK77, E3PK78, E3PK80, E3PK81, E3PK85, E3PK89, E3PK90, E3PK93, E3PK95, E3PK96, E3PKD6, E3PKE3, E3PKE5, E3PKJ0, E3PKJ1, E3PKJ7, E3PKJ8, E3PKJ9, E3PKK3, E3PKK4, E3PKL9, E3PKN2, E3PKN3, E3PKP3, E3PKP9, E3PKQ0, E3PKS3, E3PKS4, E3PKT9, E3PKV1, E3PKW2, E3PKW6, E3PKX1, E3PKX2, E3PKX8, E3PKX9, E3PKY0, E3PKY1, E3PKY2, E3PKY3, E3PKY4, E3PKY5, E3PKY6, E3PKY7, E3PKY8, E3PKY9, E3PKZ0, E3PKZ1, E3PKZ2, E3PKZ3, E3PKZ4, E3PKZ5, E3PKZ6, E3PKZ7, E3PKZ8, E3PKZ9, E3PL00, E3PL01, E3PL02, E3PL03, E3PL04, E3PL05, E3PL25, E3PL27, E3PL36, E3PL46, E3PL50, E3PL51, E3PL78, E3PLA3, E3PLA4, E3PLA5, E3PLE7, E3PLE8, E3PLE9, E3PLF0, E3PLG0, E3PLJ1, E3PLK7, E3PLL4, E3PLL6, E3PLM5, E3PLM6, E3PLM7, E3PLP6, E3PLQ2, E3PLQ6, E3PLQ7, E3PLR4, E3PLS0, E3PLU2, E3PLY3, E3PLY4, E3PLZ6, E3PM13, E3PM19, E3PM53, E3PM54, E3PM69, E3PM70, E3PM78, E3PM90, E3PMC8, E3PMD0, E3PMD1, E3PME4, E3PME5, E3PME8, E3PME9, E3PMF6, E3PMK4, E3PML4, E3PMM9, E3PMN0, E3PMN8, E3PMQ7, E3PMV5, E3PMV6, E3PMX0, E3PMZ7, E3PMZ8, E3PMZ9, E3PN05, E3PN11, E3PN13, E3PN14, E3PN33, E3PN34, E3PN53, E3PN84, E3PN94, E3PN98, E3PN99, E3PNA0, E3PNA1, E3PNA2, E3PNA3, E3PNE0, E3PNH1, E3PNI8, E3PNJ2, E3PNJ3, E3PNK8, E3PNL7, E3PNM1, E3PNN0, E3PNQ1, E3PNQ2, E3PNQ5, E3PNQ6, E3PNS2, E3PNS4, E3PNT1, E3PNT9, E3PNU0, E3PNU8, E3PNV8, E3PNX0, E3PNX3, E3PP14, E3PP26, E3PPE3, E3PPG8 |
| **O26:H11 (strain 11368 / EHEC)** | C8TFS5, C8TFT8, C8TFT9, C8TFV4, C8TFX7, C8TFZ1, C8TFZ3, C8TFZ9, C8TG04, C8TG05, C8TG06, C8TG07, C8TG08, C8TG09, C8TG29, C8TG30, C8TG32, C8TG38, C8TG53, C8TG65, C8TG68, C8TGE6, C8TGE7, C8TGF1, C8TGF4, C8TGI3, C8TGI8, C8TGI9, C8TGJ6, C8TGJ9, C8TGK3, C8TGM5, C8TGM8, C8TGM9, C8TGQ2, C8TGQ3, C8TGQ4, C8TGR2, C8TGU1, C8TGU2, C8TGV2, C8TGV7, C8TGW7, C8TGW8, C8TH75, C8TH84, C8TH85, C8TH86, C8TH92, C8THD0, C8THE6, C8THF5, C8THH0, C8THH1, C8THH4, C8THH5, C8THH8, C8THI7, C8THJ5, C8THJ6, C8THJ8, C8THK9, C8THL0, C8THL4, C8THL8, C8THM1, C8THM6, C8THN5, C8THN6, C8THS2, C8THV3, C8THV4, C8THW0, C8THZ2, C8TI12, C8TI18, C8TI20, C8TI72, C8TI74, C8TI75, C8TI76, C8TI78, C8TI79, C8TI83, C8TI87, C8TI88, C8TI91, C8TI93, C8TI94, C8TIA3, C8TIA9, C8TIB0, C8TID1, C8TID2, C8TIE7, C8TIG0, C8TIG8, C8TII0, C8TII4, C8TII9, C8TIJ0, C8TIJ6, C8TIJ7, C8TIJ8, C8TIJ9, C8TIK0, C8TIK1, C8TIK2, C8TIK3, C8TIK4, C8TIK5, C8TIK6, C8TIK7, C8TIK8, C8TIK9, C8TIL0, C8TIL1, C8TIL2, C8TIL3, C8TIL4, C8TIL5, C8TIL6, C8TIL7, C8TIL8, C8TIL9, C8TIM0, C8TIM1, C8TIM2, C8TIM3, C8TIM7, C8TIM8, C8TIM9, C8TIN0, C8TIP1, C8TIS2, C8TIW2, C8TIZ5, C8TIZ6, C8TJ04, C8TJ06, C8TJ13, C8TJ29, C8TJ38, C8TJ41, C8TJ42, C8TJ67, C8TJ74, C8TJ83, C8TJ88, C8TJB2, C8TJJ0, C8TJJ7, C8TJJ9, C8TJK9, C8TJL0, C8TJL1, C8TJN3, C8TJN9, C8TJP3, C8TJP4, C8TJQ2, C8TJQ3, C8TJR8, C8TJR9, C8TJS4, C8TJT6, C8TJX6, C8TJY1, C8TJY2, C8TJZ6, C8TJZ7, C8TK48, C8TK64, C8TK71, C8TK74, C8TKA2, C8TKA3, C8TKA4, C8TKB1, C8TKE6, C8TKG4, C8TKH3, C8TKJ0, C8TKL9, C8TKQ8, C8TKR3, C8TKR9, C8TKS5, C8TKY2, C8TKY4, C8TKZ5, C8TL02, C8TL34, C8TL37, C8TL49, C8TL59, C8TL67, C8TL68, C8TL76, C8TL87, C8TL89, C8TLA5, C8TLA6, C8TLA8, C8TLA9, C8TLD8, C8TLD9, C8TLH6, C8TLH7, C8TLH9, C8TLI5, C8TLJ1, C8TLJ2, C8TLJ3, C8TLL9, C8TLP4, C8TLQ9, C8TLR2, C8TLR5, C8TLS5, C8TLT5, C8TLU5, C8TLX1, C8TLX5, C8TLY0, C8TLZ5, C8TLZ6, C8TM01, C8TM02, C8TM08, C8TM26, C8TM29, C8TM32, C8TM36, C8TM46, C8TM49, C8TM64, C8TM65, C8TM78, C8TMB0, C8TME3, C8TME4, C8TMG7, C8TMH5, C8TMH6, C8TMH7, C8TMH8, C8TMH9, C8TML3, C8TML9, C8TMM0, C8TMM2, C8TMM3, C8TMM4, C8TMM5, C8TMM6, C8TMM7, C8TMN5, C8TMR2, C8TMS6, C8TMT9, C8TMU9, C8TMY1, C8TMY2, C8TMZ0, C8TN13, C8TNC0, C8TND6, C8TNG9, C8TNJ8, C8TNL4, C8TNP0, C8TNT0, C8TNX9, C8TNY5, C8TNY6, C8TNY8, C8TNZ2, C8TNZ3, C8TNZ4, C8TP00, C8TP01, C8TP20, C8TP22, C8TP23, C8TP45, C8TP46, C8TP49, C8TP66, C8TP70, C8TP78, C8TPA3, C8TPC8, C8TPF4, C8TPF6, C8TPF9, C8TPH3, C8TPI4, C8TPI7, C8TPL3, C8TPS9, C8TPT0, C8TQ28, C8TQ77, C8TQ87, C8TQ89, C8TQ90, C8TQC5, C8TQC8, C8TQE4, C8TQF6, C8TQG6, C8TQG8, C8TQL5, C8TQL6, C8TQM2, C8TQM3, C8TQM4, C8TQM8, C8TQM9, C8TQP3, C8TQQ5, C8TQQ6, C8TQT5, C8TQW6, C8TQW7, C8TQX5, C8TQY6, C8TQZ0, C8TQZ1, C8TR17, C8TR42, C8TR43, C8TR44, C8TR51, C8TR68, C8TRE0, C8TRE6, C8TRG5, C8TRK4, C8TRL6, C8TRN2, C8TRU1, C8TRU6, C8TRU7, C8TRV1, C8TSA4, C8TSB3, C8TSC8, C8TSC9, C8TSD8, C8TSF7, C8TSI3, C8TSN0, C8TSN1, C8TSN3, C8TSN4, C8TSN5, C8TSN6, C8TSS2, C8TSU5, C8TSW4, C8TSW8, C8TSW9, C8TSY4, C8TSZ2, C8TSZ6, C8TT42, C8TT43, C8TT58, C8TT71, C8TT92, C8TT99, C8TTA7, C8TTB7, C8TTC5, C8TTD8, C8TTJ8, C8TTL7, C8TTP6, C8TTP7, C8TTS5, C8TTU4, C8TTV3, C8TTV8, C8TTV9, C8TU40, C8TU54, C8TU64, C8TU71, C8TU73, C8TUA5, C8TUA8, C8TUA9, C8TUB0, C8TUD9, C8TUE3, C8TUF0, C8TUL4, C8TUQ2, C8TUR6, C8TUV1, C8TUW3, C8TUX5, C8TV65, C8TVA6, C8TVC4, C8TVG8, C8TVH0, C8TVH1, C8TVJ5, C8TVK2, C8TVK4, C8TVK6, C8TVK9, C8TVM7, C8TVN0, C8TVP2, C8TVQ4, C8TVR1, C8TVR2, C8TVR4, C8TVS0, C8TVS1, C8TVS6, C8TVT5, C8TVU6, C8TVU7, C8TVU8, C8TVU9, C8TVV0, C8TVV7, C8TVV9, C8TVZ4, C8TW29, C8TW36, C8TW37, C8TWC0, C8TWC1, C8TWG6, C8TWH1 |
| **O139:H28 (strain E24377A / ETEC)** | A7ZGL5, A7ZGM9, A7ZGN8, A7ZGU5, A7ZGW5, A7ZGX8, A7ZH36, A7ZHB2, A7ZHB5, A7ZHE4, A7ZHE9, A7ZHF0, A7ZHF7, A7ZHG1, A7ZHG4, A7ZHI5, A7ZHI8, A7ZHI9, A7ZHK7, A7ZHL6, A7ZHN4, A7ZHN5, A7ZHN8, A7ZHN9, A7ZHP3, A7ZHQ2, A7ZHQ9, A7ZHR0, A7ZHR2, A7ZHS3, A7ZHS4, A7ZHS8, A7ZHT2, A7ZHT6, A7ZHU1, A7ZHV1, A7ZHV2, A7ZHZ4, A7ZHZ5, A7ZI01, A7ZI30, A7ZI61, A7ZI67, A7ZI70, A7ZIF7, A7ZIF8, A7ZIG7, A7ZIG9, A7ZIH7, A7ZIJ4, A7ZIK3, A7ZIK6, A7ZIK7, A7ZIN4, A7ZIP1, A7ZIQ0, A7ZIQ5, A7ZIT5, A7ZIX2, A7ZJ11, A7ZJ31, A7ZJ43, A7ZJ63, A7ZJ95, A7ZJD5, A7ZJE0, A7ZJE6, A7ZJH5, A7ZJI8, A7ZJK5, A7ZJK8, A7ZJL2, A7ZJM4, A7ZJN6, A7ZJP6, A7ZJS3, A7ZJS7, A7ZJT3, A7ZJU8, A7ZJV3, A7ZJV4, A7ZJW2, A7ZK00, A7ZK03, A7ZK06, A7ZK11, A7ZK21, A7ZK25, A7ZK52, A7ZK53, A7ZK66, A7ZK91, A7ZK92, A7ZKB5, A7ZKE8, A7ZKI9, A7ZKJ0, A7ZKK7, A7ZKL8, A7ZKM1, A7ZKQ0, A7ZKR6, A7ZKS3, A7ZKS5, A7ZKX6, A7ZKX7, A7ZKY4, A7ZKY5, A7ZKY6, A7ZKZ0, A7ZKZ1, A7ZL05, A7ZL17, A7ZL18, A7ZL58, A7ZL85, A7ZL86, A7ZL96, A7ZLA9, A7ZLB5, A7ZLB6, A7ZLE7, A7ZLH2, A7ZLH3, A7ZLH4, A7ZLK9, A7ZLN4, A7ZLT2, A7ZLT3, A7ZLU5, A7ZLX1, A7ZLX5, A7ZM19, A7ZM88, A7ZM99, A7ZMB5, A7ZMB7, A7ZMC7, A7ZME7, A7ZMH7, A7ZMI1, A7ZMI2, A7ZMI4, A7ZMI5, A7ZMI6, A7ZMM5, A7ZMQ7, A7ZMS5, A7ZMS9, A7ZMT1, A7ZMU5, A7ZMV4, A7ZMV8, A7ZMW7, A7ZMY2, A7ZMZ0, A7ZN02, A7ZN11, A7ZN25, A7ZN42, A7ZN64, A7ZNI3, A7ZNJ1, A7ZNJ6, A7ZNJ7, A7ZNT3, A7ZNU6, A7ZNV7, A7ZNW9, A7ZNX1, A7ZP04, A7ZP07, A7ZP08, A7ZP09, A7ZP23, A7ZP27, A7ZP33, A7ZP73, A7ZP87, A7ZPC1, A7ZPD3, A7ZPE6, A7ZPK7, A7ZPM2, A7ZPR8, A7ZPS0, A7ZPU3, A7ZPV1, A7ZPV4, A7ZPV7, A7ZPX6, A7ZPX9, A7ZPZ0, A7ZQ02, A7ZQ10, A7ZQ11, A7ZQ13, A7ZQ20, A7ZQ21, A7ZQ26, A7ZQ36, A7ZQ46, A7ZQ47, A7ZQ48, A7ZQ49, A7ZQ50, A7ZQ58, A7ZQ60, A7ZQA8, A7ZQC4, A7ZQC5, A7ZQE1, A7ZQH1, A7ZQH3, A7ZQI7, A7ZQI9, A7ZQJ5, A7ZQJ9, A7ZQK0, A7ZQK1, A7ZQK2, A7ZQK3, A7ZQK4, A7ZQM5, A7ZQM6, A7ZQM8, A7ZQN4, A7ZQQ1, A7ZQS5, A7ZQZ9, A7ZR00, A7ZR09, A7ZR41, A7ZR42, A7ZR52, A7ZR58, A7ZR68, A7ZR69, A7ZR98, A7ZRA4, A7ZRB9, A7ZRD1, A7ZRT7, A7ZRU7, A7ZRU8, A7ZRU9, A7ZRV7, A7ZS03, A7ZS57, A7ZS61, A7ZS62, A7ZS63, A7ZS65, A7ZS67, A7ZS71, A7ZS75, A7ZS76, A7ZS79, A7ZS81, A7ZS83, A7ZS94, A7ZSA0, A7ZSA1, A7ZSC4, A7ZSC5, A7ZSE0, A7ZSF5, A7ZSG7, A7ZSH1, A7ZSH6, A7ZSH7, A7ZSI3, A7ZSI4, A7ZSI5, A7ZSI6, A7ZSI7, A7ZSI8, A7ZSI9, A7ZSJ0, A7ZSJ1, A7ZSJ2, A7ZSJ3, A7ZSJ4, A7ZSJ5, A7ZSJ6, A7ZSJ7, A7ZSJ8, A7ZSJ9, A7ZSK0, A7ZSK1, A7ZSK2, A7ZSK3, A7ZSK4, A7ZSK5, A7ZSK6, A7ZSK7, A7ZSK8, A7ZSK9, A7ZSL0, A7ZSL4, A7ZSL5, A7ZSL6, A7ZSL7, A7ZSM8, A7ZSQ9, A7ZSS6, A7ZST5, A7ZST8, A7ZSU9, A7ZSV1, A7ZSV2, A7ZSX5, A7ZSY2, A7ZSY7, A7ZSY8, A7ZSZ9, A7ZT00, A7ZT17, A7ZT18, A7ZT22, A7ZT35, A7ZT81, A7ZT84, A7ZT85, A7ZTA2, A7ZTA3, A7ZTA4, A7ZTA5, A7ZTA6, A7ZTB4, A7ZTD8, A7ZTD9, A7ZTG0, A7ZTI6, A7ZTI7, A7ZTI8, A7ZTJ4, A7ZTK1, A7ZTK3, A7ZTK4, A7ZTQ9, A7ZTR0, A7ZTR2, A7ZTS9, A7ZTT2, A7ZTU4, A7ZTV2, A7ZTW0, A7ZTY2, A7ZTY4, A7ZU21, A7ZU25, A7ZU29, A7ZU40, A7ZU42, A7ZU61, A7ZU65, A7ZU76, A7ZU92, A7ZUF0, A7ZUF1, A7ZUI5, A7ZUJ2, A7ZUJ3, A7ZUJ6, A7ZUJ7, A7ZUJ8, A7ZUK0, A7ZUK1, A7ZUK2, A7ZUL0, A7ZUN6, A7ZUQ7, A7ZUS0, A7ZUS9, A7ZUW6, A7ZUW7, A7ZUX5, A7ZUZ9, A7ZV28, A7ZV32, A7ZV33, A7ZV35, A7ZV40, A7ZV41, A7ZV42, A7ZV49, A7ZV51, A7ZV71, A7ZV73, A7ZV74, A7ZVA2, A7ZVA3, A7ZVA7, A7ZVC5, A7ZVC9, A7ZVD7, A7ZVE6, A7ZVG6, A7ZVJ0, A7ZVK2, A7ZVK4, A7ZVN0, A7ZVR1, A7ZVR5, A7ZVT3, A7ZVU5 |
| **O145:H25 str. 07-3858** | A0A070CDB8, A0A070CDQ3, A0A070CDR3, A0A070CDS4, A0A070CDT9, A0A070CDU6, A0A070CDV1, A0A070CE10, A0A070CEB8, A0A070CEE1, A0A070CEH9, A0A070CEJ8, A0A070CEK8, A0A070CER1, A0A070CET9, A0A070CEU0, A0A070CEW8, A0A070CEX4, A0A070CF05, A0A070CF20, A0A070CF47, A0A070CF69, A0A070CFA0, A0A070CFJ4, A0A070CFQ9, A0A070CFW7, A0A070CFX3, A0A070CFY3, A0A070CG34, A0A070CG41, A0A070CG70, A0A070CG74, A0A070CG83, A0A070CG91, A0A070CGF2, A0A070CGM4, A0A070CGP5, A0A070CGS8, A0A070CH90, A0A070CH98, A0A070CHD6, A0A070CHE8, A0A070CHF8, A0A070CHG2, A0A070CHG4, A0A070CHH2, A0A070CHH4, A0A070CHI8, A0A070CHJ3, A0A070CHL7, A0A070CHM3, A0A070CHN1, A0A070CHP0, A0A070CHP9, A0A070CHR8, A0A070CHU8, A0A070CHV1, A0A070CI35, A0A070CI61, A0A070CIH8, A0A070CIK1, A0A070CIL8, A0A070CIV9, A0A070CIW5, A0A070CIW8, A0A070CJ56, A0A070CJB0, A0A070CJB6, A0A070CJL6, A0A070CJP2, A0A070CJS8, A0A070CL90, A0A070CL96, A0A070CLE7, A0A070CLH7, A0A070CLN0, A0A070CLS7, A0A070CLU6, A0A070CLV6, A0A070CM53, A0A070CMG1, A0A070CMI5, A0A070CMK6, A0A070CMS1, A0A070CMY9, A0A070CN24, A0A070CN91, A0A070CND7, A0A070CNE6, A0A070CNF7, A0A070CNG1, A0A070CNG2, A0A070CPB1, A0A070CPC3, A0A070CPC7, A0A070CPD6, A0A070CPD8, A0A070CPE6, A0A070CPN1, A0A070CPT1, A0A070CPZ2, A0A070CQB0, A0A070CQK9, A0A070CQM3, A0A070CQM7, A0A070CQQ7, A0A070CQS5, A0A070CQS9, A0A070CQW3, A0A070CRF1, A0A070CRF6, A0A070CRJ7, A0A070CRR9, A0A070CWB4, A0A070CWM4, A0A070CWP1, A0A070CWT9, A0A070CWV0, A0A070CXK9, A0A070CXP0, A0A070D036, A0A070D0R6, A0A070D0U2, A0A070D0U9, A0A070D0Y9, A0A070D0Z5, A0A070D100, A0A070D148, A0A070D183, A0A070D1J5, A0A070D1W2, A0A070D1Y2, A0A070D2A4, A0A070D2C0, A0A070D2G1, A0A070D2X0, A0A070D2X8, A0A070D333, A0A070D336, A0A070D339, A0A070D344, A0A070D369, A0A070D373, A0A070D381, A0A070D3B1, A0A070D3H7, A0A070D3L7, A0A070D3R8, A0A070D3S4, A0A070D3T3, A0A070D3U4, A0A070D3V7, A0A070D3W0, A0A070D417, A0A070D451, A0A070D4B3, A0A070D4I0, A0A070D4P9, A0A070D4U2, A0A070D4U8, A0A070D4W5, A0A070D4X8, A0A070D4Z5, A0A070D509, A0A070D516, A0A070D519, A0A070D523, A0A070D525, A0A070D545, A0A070D573, A0A070D578, A0A070D580, A0A070D587, A0A070D596, A0A070D598, A0A070D5B2, A0A070D5B7, A0A070D5B8, A0A070D5D6, A0A070D5G7, A0A070D5H1, A0A070D5J7, A0A070D5K3, A0A070D5K8, A0A070D5L7, A0A070D5M0, A0A070D5Q5, A0A070D5S0, A0A070D5W2, A0A070D5Y4, A0A070D649, A0A070D656, A0A070D667, A0A070D694, A0A070D6C5, A0A070D6C7, A0A070D6E9, A0A070D6F2, A0A070D6N4, A0A070D6N8, A0A070D6Q5, A0A070D6S3, A0A070D6T2, A0A070D6V4, A0A070D6Y3, A0A070D6Y5, A0A070D6Y7, A0A070D6Z1, A0A070D6Z7, A0A070D703, A0A070D714, A0A070D718, A0A070D721, A0A070D723, A0A070D728, A0A070D734, A0A070D740, A0A070D741, A0A070D752, A0A070D764, A0A070D782, A0A070D785, A0A070D788, A0A070D796, A0A070D7A6, A0A070D7A7, A0A070D7A9, A0A070D7B3, A0A070D7B7, A0A070D7C2, A0A070D7C6, A0A070D7D6, A0A070D7H7, A0A070D7I6, A0A070D7K0, A0A070D7K7, A0A070D7P4, A0A070D7P9, A0A070D7S3, A0A070D7T4, A0A070D7U2, A0A070D7V9, A0A070D7X2, A0A070D7Z9, A0A070D812, A0A070D823, A0A070D853, A0A070D855, A0A070D888, A0A070D891, A0A070D8A7, A0A070D8C2, A0A070D8G7, A0A070D8H2, A0A070D8H7, A0A070D8J4, A0A070D8K2, A0A070D8K6, A0A070D8K9, A0A070D8L1, A0A070D8L3, A0A070D8L4, A0A070D8M2, A0A070D8S7, A0A070D8T2, A0A070D8U1, A0A070D8U3, A0A070D8U5, A0A070D8U7, A0A070D8U9, A0A070D8V0, A0A070D8V1, A0A070D8V3, A0A070D8V5, A0A070D8V7, A0A070D8V9, A0A070D8W0, A0A070D8W7, A0A070D8W8, A0A070D8X3, A0A070D908, A0A070D915, A0A070D923, A0A070D926, A0A070D945, A0A070D955, A0A070D965, A0A070D979, A0A070D990, A0A070D992, A0A070D996, A0A070D9A2, A0A070D9A5, A0A070D9C3, A0A070D9E3, A0A070D9E5, A0A070D9E9, A0A070D9F1, A0A070D9H1, A0A070D9I2, A0A070D9I7, A0A070D9K5, A0A070D9K8, A0A070D9Q7, A0A070D9R8, A0A070D9S2, A0A070D9T7, A0A070D9V9, A0A070D9X4, A0A070DA00, A0A070DA12, A0A070DA18, A0A070DA32, A0A070DA48, A0A070DA59, A0A070DA69, A0A070DA80, A0A070DAA4, A0A070DAH2, A0A070DAJ8, A0A070DAP2, A0A070DAP8, A0A070DAR1, A0A070DAR2, A0A070DAS2, A0A070DAS8, A0A070DAW3, A0A070DB19, A0A070DBC0, A0A070DBE0, A0A070DBG5, A0A070DBL0, A0A070DBM9, A0A070DBN7, A0A070DCA7, A0A070DD00, A0A070DD46, A0A070DD59, A0A070DDJ3, A0A070DDP2, A0A070DDR4, A0A070DDT1, A0A070DDU6, A0A070DDX9, A0A070DE29, A0A070DE35, A0A070DE68, A0A070DEA9, A0A070DEF9, A0A070DEG4, A0A070DEU8, A0A070DEY8, A0A070DF26, A0A070DF57, A0A070DF80, A0A070DF93, A0A070DFD9, A0A070DFE7, A0A070DFF3, A0A070DFG0, A0A070DFG6, A0A070DFH3, A0A070DFH8, A0A070DFM5, A0A070DFQ6, A0A070DFR7, A0A070DFT6, A0A070DG62, A0A070DG72, A0A070DG92, A0A070DGC0, A0A070DGE1, A0A070DGI8, A0A070DGT6, A0A070DGU5, A0A070DGY0, A0A070DHB0, A0A070DLY0, A0A070DN59, A0A070DP80, A0A070DPA2, A0A070DPG6, A0A070DQ62, A0A070DQB6, A0A070DQC0, A0A070DQD2, A0A070DQK2, A0A070DQK8, A0A070DQY6, A0A070DR57, A0A070DRB4, A0A070DRB9, A0A070DRI9, A0A070DRM5, A0A070DRZ4, A0A070DS31, A0A070DS87, A0A070DSA6, A0A070DSQ3, A0A070DSR8, A0A070DXZ4, A0A070DY22, A0A070DYI3, A0A070DZ36, A0A070DZC8, A0A070DZF0, A0A070DZI8, A0A070DZY9 |
| **O55:H7 (strain CB9615 / EPEC)** | D3QJ91, D3QJ93, D3QJD0, D3QJD7, D3QJH3, D3QJH4, D3QJI4, D3QJI6, D3QJJ4, D3QJK7, D3QJL6, D3QJL9, D3QJQ0, D3QJR5, D3QJS6, D3QJT9, D3QJU1, D3QJX2, D3QJX5, D3QJX6, D3QJX7, D3QJZ0, D3QJZ4, D3QK00, D3QK29, D3QK42, D3QK55, D3QK65, D3QK96, D3QKA3, D3QKA4, D3QKB2, D3QKD5, D3QKF9, D3QKG6, D3QKH4, D3QKI2, D3QKI7, D3QKL6, D3QKN6, D3QKV2, D3QKW6, D3QKZ9, D3QL11, D3QL23, D3QL51, D3QL77, D3QL81, D3QL82, D3QL84, D3QL88, D3QL89, D3QL90, D3QL96, D3QL97, D3QLB7, D3QLB9, D3QLC0, D3QLE3, D3QLE4, D3QLE7, D3QLG2, D3QLG6, D3QLH5, D3QLM3, D3QLM4, D3QLN1, D3QLR6, D3QLT4, D3QLU3, D3QLZ4, D3QM09, D3QM53, D3QM79, D3QM87, D3QM98, D3QM99, D3QMA1, D3QMC6, D3QMD9, D3QME1, D3QME2, D3QMG3, D3QMI3, D3QMI6, D3QMI7, D3QMJ3, D3QML8, D3QMP6, D3QMU6, D3QMV1, D3QMV7, D3QMZ6, D3QMZ8, D3QN01, D3QN23, D3QN26, D3QN38, D3QN50, D3QNA7, D3QNA8, D3QNB1, D3QNB7, D3QNB8, D3QNC3, D3QND7, D3QNF1, D3QNG3, D3QNJ5, D3QNL0, D3QNL3, D3QNL5, D3QNM5, D3QNN6, D3QNP5, D3QNS3, D3QNS7, D3QNT5, D3QNV3, D3QNW4, D3QNW5, D3QNW6, D3QNW7, D3QNW8, D3QNX5, D3QNX7, D3QP34, D3QP79, D3QP80, D3QP85, D3QP86, D3QP92, D3QPA8, D3QPB1, D3QPB4, D3QPB8, D3QPC7, D3QPD0, D3QPE4, D3QPE5, D3QPI3, D3QPI4, D3QPK3, D3QPN0, D3QPP3, D3QPP4, D3QPQ9, D3QPT2, D3QPU0, D3QPU2, D3QPU8, D3QPV3, D3QPV4, D3QPV5, D3QPV6, D3QPV7, D3QPV9, D3QPY1, D3QPY2, D3QPY4, D3QQ20, D3QQ40, D3QQ79, D3QQ81, D3QQ84, D3QQ98, D3QQA9, D3QQB2, D3QQB9, D3QQC4, D3QQD9, D3QQE9, D3QQL4, D3QQL5, D3QQM3, D3QQR6, D3QQR8, D3QQW3, D3QQW4, D3QQX6, D3QQX7, D3QQY3, D3QQY4, D3QQY5, D3QQY9, D3QR03, D3QR15, D3QR16, D3QR31, D3QR32, D3QR50, D3QR51, D3QRH8, D3QRN6, D3QRN8, D3QRP7, D3QRQ8, D3QRR7, D3QRR8, D3QRX8, D3QS02, D3QS11, D3QS12, D3QS13, D3QS19, D3QS56, D3QS77, D3QSA2, D3QSA3, D3QSA4, D3QSB2, D3QSC5, D3QSH9, D3QSN4, D3QSN6, D3QSN7, D3QSN8, D3QSP0, D3QSP1, D3QSP5, D3QSP9, D3QSQ0, D3QSQ3, D3QSQ5, D3QSQ6, D3QSR5, D3QSS1, D3QSS2, D3QSU4, D3QSU5, D3QSW0, D3QSX3, D3QSY3, D3QSY9, D3QT17, D3QT30, D3QT31, D3QT43, D3QT62, D3QT68, D3QTC2, D3QTC7, D3QTC8, D3QTD3, D3QTD4, D3QTD5, D3QTD6, D3QTD7, D3QTD8, D3QTD9, D3QTE0, D3QTE1, D3QTE2, D3QTE3, D3QTE4, D3QTE5, D3QTE6, D3QTE7, D3QTE8, D3QTE9, D3QTF0, D3QTF1, D3QTF2, D3QTF3, D3QTF4, D3QTF5, D3QTF6, D3QTF7, D3QTF8, D3QTF9, D3QTG0, D3QTG5, D3QTG6, D3QTG7, D3QTG8, D3QTH9, D3QTK1, D3QTL6, D3QTM4, D3QTM6, D3QTN9, D3QTP0, D3QTP1, D3QTR8, D3QTY5, D3QTZ4, D3QU09, D3QU10, D3QU18, D3QU52, D3QU57, D3QU58, D3QU68, D3QU69, D3QUA2, D3QUA3, D3QUB5, D3QUC7, D3QUE4, D3QUH6, D3QUK3, D3QUQ5, D3QUQ7, D3QUQ8, D3QUR0, D3QUR1, D3QUR2, D3QUR3, D3QUU9, D3QUW5, D3QUW7, D3QUW8, D3QUY6, D3QUY7, D3QUY8, D3QUY9, D3QUZ0, D3QUZ8, D3QV15, D3QV16, D3QV34, D3QV60, D3QV61, D3QV62, D3QV68, D3QV74, D3QV76, D3QV77, D3QV99, D3QVA7, D3QVB0, D3QVD8, D3QVE4, D3QVE5, D3QVF2, D3QVF6, D3QVF8, D3QVI0, D3QVI3, D3QVI4, D3QVK3, D3QVL2, D3QVQ2, D3QVR9, D3QVS3, D3QVS4, D3QVT9, D3QVU8, D3QVV2, D3QVW1, D3QVX5, D3QVY3, D3QVZ3, D3QW01, D3QW29, D3QW59, D3QW70, D3QW95, D3QW96, D3QW98, D3QWA1, D3QWB7, D3QWC9, D3QWD7, D3QWD8, D3QWE5, D3QWE6, D3QWE9, D3QWF0, D3QWF3, D3QWG2, D3QWG9, D3QWH0, D3QWH2, D3QWI3, D3QWI4, D3QWI8, D3QWJ2, D3QWJ5, D3QWK0, D3QWK9, D3QWL0, D3QWN4, D3QWQ5, D3QWQ7, D3QWQ8, D3QWR4, D3QWS3, D3QWT8, D3QWW2, D3QX39, D3QX51, D3QX61, D3QX73, D3QX76, D3QXA8, D3QXB1, D3QXB7, D3QXC8, D3QXD0, D3QXE8, D3QXF2, D3QXF9, D3QXH8, D3QXR6, D3QXU6, D3QXZ0, D3QXZ9, D3QY04, D3QY05, D3QY83, D3QYD5, D3QYD6, D3QYH2, D3QYH9, D3QYI0, D3QYI2, D3QYI3, D3QYI4, D3QYI5, D3QYI6, D3QYI7, D3QYJ5, D3QYM0, D3QYQ5 |
| **O104:H4 (strain 2011C-3493)** | A0A0E0XS57, A0A0E0XSB3, A0A0E0XSF0, A0A0E0XSH3, A0A0E0XSI3, A0A0E0XSK3, A0A0E0XSS6, A0A0E0XSY1, A0A0E0XT14, A0A0E0XT25, A0A0E0XTA6, A0A0E0XTB3, A0A0E0XTB8, A0A0E0XTC3, A0A0E0XTC7, A0A0E0XTD2, A0A0E0XTD7, A0A0E0XTE1, A0A0E0XTF9, A0A0E0XTL8, A0A0E0XTM3, A0A0E0XTN5, A0A0E0XTR1, A0A0E0XTR5, A0A0E0XTS0, A0A0E0XTS4, A0A0E0XTS8, A0A0E0XTT6, A0A0E0XTU1, A0A0E0XTV3, A0A0E0XTX6, A0A0E0XTY6, A0A0E0XTZ2, A0A0E0XU09, A0A0E0XU12, A0A0E0XU23, A0A0E0XU38, A0A0E0XU44, A0A0E0XU46, A0A0E0XU66, A0A0E0XUB9, A0A0E0XUI6, A0A0E0XUM7, A0A0E0XUP1, A0A0E0XUP9, A0A0E0XUQ0, A0A0E0XUQ3, A0A0E0XUT2, A0A0E0XUU0, A0A0E0XUW0, A0A0E0XUX1, A0A0E0XUX6, A0A0E0XUY0, A0A0E0XUY6, A0A0E0XUZ1, A0A0E0XUZ4, A0A0E0XUZ5, A0A0E0XUZ9, A0A0E0XV01, A0A0E0XV15, A0A0E0XV22, A0A0E0XV29, A0A0E0XV33, A0A0E0XV38, A0A0E0XV40, A0A0E0XV41, A0A0E0XV46, A0A0E0XV56, A0A0E0XV61, A0A0E0XV63, A0A0E0XV68, A0A0E0XV75, A0A0E0XV81, A0A0E0XV95, A0A0E0XVA2, A0A0E0XVC5, A0A0E0XVD6, A0A0E0XVE0, A0A0E0XVE1, A0A0E0XVE4, A0A0E0XVE8, A0A0E0XVF4, A0A0E0XVG6, A0A0E0XVI3, A0A0E0XVI6, A0A0E0XVJ1, A0A0E0XVJ4, A0A0E0XVL7, A0A0E0XVN3, A0A0E0XVQ5, A0A0E0XVQ7, A0A0E0XVR2, A0A0E0XVU4, A0A0E0XVV5, A0A0E0XVW0, A0A0E0XVW1, A0A0E0XVX0, A0A0E0XVZ3, A0A0E0XW29, A0A0E0XW34, A0A0E0XW38, A0A0E0XW56, A0A0E0XW84, A0A0E0XW91, A0A0E0XWC3, A0A0E0XWD1, A0A0E0XWD6, A0A0E0XWE6, A0A0E0XWE7, A0A0E0XWF5, A0A0E0XWH3, A0A0E0XWH8, A0A0E0XWI2, A0A0E0XWI4, A0A0E0XWN1, A0A0E0XWN6, A0A0E0XWP0, A0A0E0XWQ2, A0A0E0XWQ4, A0A0E0XWR3, A0A0E0XWT4, A0A0E0XWU4, A0A0E0XWU7, A0A0E0XWV6, A0A0E0XWV7, A0A0E0XWW2, A0A0E0XWW3, A0A0E0XWW8, A0A0E0XWW9, A0A0E0XWX3, A0A0E0XWX8, A0A0E0XWZ0, A0A0E0XWZ3, A0A0E0XX01, A0A0E0XX02, A0A0E0XX26, A0A0E0XX40, A0A0E0XX65, A0A0E0XX80, A0A0E0XX81, A0A0E0XX90, A0A0E0XX96, A0A0E0XX99, A0A0E0XXA0, A0A0E0XXA6, A0A0E0XXB6, A0A0E0XXE0, A0A0E0XXI9, A0A0E0XXJ0, A0A0E0XXK9, A0A0E0XXM6, A0A0E0XXN7, A0A0E0XXQ9, A0A0E0XXU4, A0A0E0XXX8, A0A0E0XXZ3, A0A0E0XY03, A0A0E0XY39, A0A0E0XY48, A0A0E0XY53, A0A0E0XY82, A0A0E0XY93, A0A0E0XYB1, A0A0E0XYB7, A0A0E0XYC0, A0A0E0XYC5, A0A0E0XYC7, A0A0E0XYD0, A0A0E0XYE5, A0A0E0XYE7, A0A0E0XYF1, A0A0E0XYF3, A0A0E0XYF9, A0A0E0XYK2, A0A0E0XYM3, A0A0E0XYN4, A0A0E0XYP3, A0A0E0XYP6, A0A0E0XYP7, A0A0E0XYP9, A0A0E0XYQ2, A0A0E0XYQ7, A0A0E0XYV9, A0A0E0XYX1, A0A0E0XYX8, A0A0E0XZ10, A0A0E0XZ16, A0A0E0XZ35, A0A0E0XZ60, A0A0E0XZ81, A0A0E0XZA1, A0A0E0XZA5, A0A0E0XZB4, A0A0E0XZD9, A0A0E0XZE7, A0A0E0XZJ2, A0A0E0XZJ5, A0A0E0XZM1, A0A0E0XZM5, A0A0E0XZM7, A0A0E0XZN7, A0A0E0XZQ1, A0A0E0XZR9, A0A0E0XZS2, A0A0E0XZT8, A0A0E0XZU2, A0A0E0XZX6, A0A0E0XZY3, A0A0E0Y001, A0A0E0Y027, A0A0E0Y029, A0A0E0Y035, A0A0E0Y041, A0A0E0Y043, A0A0E0Y044, A0A0E0Y054, A0A0E0Y068, A0A0E0Y079, A0A0E0Y081, A0A0E0Y0A9, A0A0E0Y0B2, A0A0E0Y0E1, A0A0E0Y0E3, A0A0E0Y0E9, A0A0E0Y0G1, A0A0E0Y0G4, A0A0E0Y0L9, A0A0E0Y0P0, A0A0E0Y0P4, A0A0E0Y0R0, A0A0E0Y0R4, A0A0E0Y0S8, A0A0E0Y0W9, A0A0E0Y0X0, A0A0E0Y0Z0, A0A0E0Y145, A0A0E0Y166, A0A0E0Y183, A0A0E0Y190, A0A0E0Y191, A0A0E0Y199, A0A0E0Y1F6, A0A0E0Y1G9, A0A0E0Y1H1, A0A0E0Y1H6, A0A0E0Y1I0, A0A0E0Y1K8, A0A0E0Y1K9, A0A0E0Y1L3, A0A0E0Y1L7, A0A0E0Y1M0, A0A0E0Y1N2, A0A0E0Y1R0, A0A0E0Y1S5, A0A0E0Y1S8, A0A0E0Y1U8, A0A0E0Y1W1, A0A0E0Y1Y7, A0A0E0Y1Y8, A0A0E0Y219, A0A0E0Y224, A0A0E0Y229, A0A0E0Y254, A0A0E0Y2C5, A0A0E0Y2L3, A0A0E0Y2M4, A0A0E0Y2N0, A0A0E0Y2N9, A0A0E0Y2P8, A0A0E0Y2S7, A0A0E0Y2S8, A0A0E0Y2S9, A0A0E0Y2U0, A0A0E0Y2V3, A0A0E0Y2Z1, A0A0E0Y318, A0A0E0Y326, A0A0E0Y328, A0A0E0Y330, A0A0E0Y348, A0A0E0Y350, A0A0E0Y355, A0A0E0Y357, A0A0E0Y359, A0A0E0Y366, A0A0E0Y367, A0A0E0Y377, A0A0E0Y378, A0A0E0Y3A6, A0A0E0Y3B1, A0A0E0Y3G4, A0A0E0Y3K6, A0A0E0Y3L7, A0A0E0Y3M5, A0A0E0Y3N4, A0A0E0Y3N5, A0A0E0Y3P0, A0A0E0Y3Q3, A0A0E0Y3Q9, A0A0E0Y3T2, A0A0E0Y3U3, A0A0E0Y3V2, A0A0E0Y3V7, A0A0E0Y3V8, A0A0E0Y3W0, A0A0E0Y3W9, A0A0E0Y3X9, A0A0E0Y3Y0, A0A0E0Y3Y1, A0A0E0Y3Y7, A0A0E0Y3Z0, A0A0E0Y3Z7, A0A0E0Y410, A0A0E0Y417, A0A0E0Y445, A0A0E0Y448, A0A0E0Y467, A0A0E0Y469, A0A0E0Y474, A0A0E0Y484, A0A0E0Y4B9, A0A0E0Y4C7, A0A0E0Y4D8, A0A0E0Y4G5, A0A0E0Y4I5, A0A0E0Y4I7, A0A0E0Y4K4, A0A0E0Y4M0, A0A0E0Y4N9, A0A0E0Y4R7, A0A0E0Y4T8, A0A0E0Y4U8, A0A0E0Y4W0, A0A0E0Y4W3, A0A0E0Y4X5, A0A0E0Y4Y6, A0A0E0Y4Z3, A0A0E0Y4Z6, A0A0E0Y506, A0A0E0Y516, A0A0E0Y527, A0A0E0Y542, A0A0E0Y543, A0A0E0Y548, A0A0E0Y569, A0A0E0Y580, A0A0E0Y597, A0A0E0Y5B0, A0A0E0Y5E1, A0A0E0Y5E8, A0A0E0Y5F0, A0A0E0Y5F5, A0A0E0Y5F9, A0A0E0Y5H9, A0A0E0Y5K0, A0A0E0Y5N1, A0A0E0Y5N7, A0A0E0Y5P0, A0A0E0Y5Q8, A0A0E0Y5S4, A0A0E0Y5T0, A0A0E0Y5U1, A0A0E0Y5U6, A0A0E0Y5W0, A0A0E0Y5W9, A0A0E0Y5X1, A0A0E0Y5X9, A0A0E0Y5Y3, A0A0E0Y5Z3, A0A0E0Y5Z8, A0A0E0Y601, A0A0E0Y605, A0A0E0Y609, A0A0E0Y650, A0A0E0Y651, A0A0E0Y655, A0A0E0Y697, A0A0E0Y6C1, A0A0E0Y6D2, A0A0E0Y6E5, A0A0E0Y6F2, A0A0E0Y6G1, A0A0E0Y6G7, A0A0E0Y6K1, A0A0E0Y6K5, A0A0E0Y6N2, A0A0E0Y6R5, A0A0E0Y6S5, A0A0E0Y6S9, A0A0E0Y6U6, A0A0E0Y6V4, A0A0E0Y6W8, A0A0E0Y6Y4, A0A0E0Y713, A0A0E0Y717, A0A0E0Y746, A0A0E0Y7A2, A0A0E0Y7B7, A0A0E0Y7E3, A0A0E0Y7G6, A0A0E0Y7J3, A0A0E0Y7K6, A0A0E0Y7L1, A0A0E0Y7L7, A0A0E0Y7L9, A0A0E0Y7P0, A0A0E0Y7P7, A0A0E0Y7R6, A0A0E0Y7T9, A0A0E0Y7U3, A0A0E0Y7U4, A0A0E0Y7W3, A0A0E0Y7Y0, A0A0E0Y801, A0A0E0Y810, A0A0E0Y834, A0A0E0Y854, A0A0E0Y8A8, A0A0E0Y8B4, A0A0E0Y8D4, A0A0E0Y8H6, A0A0E0Y8J2, A0A0E0Y8J7, A0A0E0Y8N9, A0A0E0Y8Q9, A0A0E0Y8T4, A0A0E0Y915, A0A0E0Y945, A0A0E0Y951, A0A0E0Y998, A0A0E0Y9Q0, A0A0E0Y9X9, A0A0E0YAG4 |
| **O103:H2 (strain 12009 / EHEC)** | C8TWJ5, C8TWM9, C8TWZ2, C8TX01, C8TX02, C8TX03, C8TX19, C8TX32, C8TX58, C8TX68, C8TXA9, C8TXF9, C8TXG1, C8TXG2, C8TXG3, C8TXG5, C8TXG6, C8TXH0, C8TXH4, C8TXH5, C8TXH8, C8TXI0, C8TXI1, C8TXJ0, C8TXJ6, C8TXJ7, C8TXL9, C8TXM0, C8TXN5, C8TXP7, C8TXQ8, C8TXR2, C8TXR7, C8TXR8, C8TXS4, C8TXS5, C8TXS6, C8TXS7, C8TXS8, C8TXS9, C8TXT0, C8TXT1, C8TXT2, C8TXT3, C8TXT4, C8TXT5, C8TXT6, C8TXT7, C8TXT8, C8TXT9, C8TXU0, C8TXU1, C8TXU2, C8TXU3, C8TXU4, C8TXU5, C8TXU6, C8TXU7, C8TXU8, C8TXU9, C8TXV0, C8TXV1, C8TXV5, C8TXV6, C8TXV7, C8TXV8, C8TXW9, C8TY00, C8TY16, C8TY23, C8TY25, C8TY35, C8TY36, C8TY53, C8TY60, C8TY66, C8TY70, C8TY71, C8TY79, C8TY80, C8TY95, C8TY96, C8TYA8, C8TYB9, C8TYG0, C8TYG1, C8TYG5, C8TYG6, C8TYH7, C8TYJ3, C8TYK0, C8TYK3, C8TYM1, C8TYM3, C8TYN4, C8TYP1, C8TYS3, C8TYS6, C8TYT8, C8TYU8, C8TYV6, C8TYV7, C8TYW5, C8TYX2, C8TYX4, C8TYZ0, C8TYZ1, C8TYZ3, C8TYZ4, C8TZ24, C8TZ25, C8TZ45, C8TZ46, C8TZ48, C8TZ60, C8TZ74, C8TZ80, C8TZ81, C8TZ82, C8TZA8, C8TZC6, C8TZC7, C8TZH6, C8TZH7, C8TZK0, C8TZK8, C8TZK9, C8TZL0, C8TZL1, C8TZL2, C8TZQ6, C8TZR2, C8TZR3, C8TZR5, C8TZR6, C8TZR7, C8TZR8, C8TZR9, C8TZS0, C8TZS7, C8TZU8, C8TZW2, C8TZX5, C8TZY5, C8U015, C8U024, C8U025, C8U033, C8U056, C8U0C2, C8U0C6, C8U0C7, C8U0C9, C8U0D3, C8U0D4, C8U0D5, C8U0E1, C8U0E2, C8U0G2, C8U0G3, C8U0G4, C8U0J0, C8U0J1, C8U0J4, C8U0K9, C8U0L3, C8U0M1, C8U0N1, C8U0N3, C8U0N4, C8U0N8, C8U0P7, C8U0S9, C8U0X0, C8U0Z1, C8U147, C8U163, C8U175, C8U191, C8U192, C8U196, C8U199, C8U1C4, C8U1C9, C8U1D0, C8U1D7, C8U1E0, C8U1E4, C8U1G6, C8U1G9, C8U1I8, C8U1J7, C8U1L3, C8U1L4, C8U1L7, C8U1L8, C8U1M1, C8U1N0, C8U1N7, C8U1N8, C8U1P0, C8U1Q1, C8U1Q2, C8U1Q6, C8U1R0, C8U1R2, C8U1R7, C8U1S6, C8U1S7, C8U1W6, C8U1W7, C8U1X3, C8U1Y8, C8U208, C8U214, C8U216, C8U256, C8U289, C8U290, C8U298, C8U2A0, C8U2A7, C8U2C3, C8U2D2, C8U2D5, C8U2D6, C8U2G0, C8U2G7, C8U2H6, C8U2I1, C8U2K7, C8U2N4, C8U2U4, C8U2U5, C8U2V2, C8U2Y6, C8U304, C8U314, C8U331, C8U358, C8U383, C8U390, C8U3G7, C8U3H5, C8U3I8, C8U3K2, C8U3M1, C8U3U4, C8U3Y9, C8U3Z4, C8U400, C8U451, C8U467, C8U470, C8U472, C8U482, C8U4A0, C8U4B0, C8U4D6, C8U4E0, C8U4E5, C8U4G0, C8U4G1, C8U4G6, C8U4G7, C8U4H3, C8U4J1, C8U4J4, C8U4J6, C8U4K0, C8U4L0, C8U4L3, C8U4Y9, C8U555, C8U564, C8U569, C8U570, C8U5B2, C8U5B3, C8U5C6, C8U5F1, C8U5F2, C8U5H2, C8U5K1, C8U5N9, C8U5P1, C8U5P4, C8U5Q8, C8U5R9, C8U5S2, C8U5U7, C8U5Z0, C8U657, C8U672, C8U682, C8U689, C8U691, C8U6C2, C8U6C5, C8U6C6, C8U6C7, C8U6D9, C8U6E3, C8U6F0, C8U6I3, C8U6J7, C8U6M9, C8U6P1, C8U6Q4, C8U6R9, C8U6S1, C8U6W5, C8U6W6, C8U6X2, C8U6X3, C8U6X4, C8U6X8, C8U6X9, C8U6Y0, C8U6Z4, C8U706, C8U707, C8U734, C8U735, C8U736, C8U785, C8U786, C8U794, C8U7A5, C8U7A9, C8U7B0, C8U7D6, C8U7G0, C8U7G1, C8U7G2, C8U7L2, C8U7P3, C8U7T3, C8U7V1, C8U7Z5, C8U7Z7, C8U7Z8, C8U819, C8U826, C8U828, C8U830, C8U833, C8U851, C8U854, C8U891, C8U897, C8U8B7, C8U8F6, C8U8F7, C8U8G6, C8U8I4, C8U8J0, C8U8N2, C8U8V0, C8U8W2, C8U8X4, C8U936, C8U937, C8U939, C8U945, C8U946, C8U952, C8U961, C8U972, C8U973, C8U974, C8U975, C8U976, C8U984, C8U986, C8U9B0, C8U9C3, C8U9C4, C8U9D9, C8U9G1, C8U9H5, C8U9H7, C8U9I3, C8U9I8, C8U9I9, C8U9J0, C8U9J1, C8U9J2, C8U9J3, C8U9K6, C8U9M1, C8U9M2, C8U9N1, C8U9Q1, C8U9S7, C8U9X8, C8U9X9, C8U9Y1, C8U9Y2, C8U9Y3, C8U9Y4, C8UA19, C8UA41, C8UA61, C8UA65, C8UA66, C8UA81, C8UA90, C8UA94, C8UAA2, C8UAB4, C8UAB5, C8UAB7, C8UAC3, C8UAD8, C8UAG7, C8UAL6, C8UAP4, C8UAP5, C8UAP6, C8UAQ5, C8UAT3, C8UAV1, C8UAV2, C8UB11, C8UB23 |
