# Supplementary material for: Bioinformatics comparisons of RNA-binding proteins of pathogenic and non-pathogenic Escherichia coli strains reveal novel virulence factors
Source: BMC Genomics. 2017 Aug 24;18:658. doi: 10.1186/s12864-017-4045-3 (PMC5571608; doi:10.1186/s12864-017-4045-3)
Supplement: Supplementary file 2 — Clusters of RNA-binding proteins obtained from 19 E. coli proteomes. The clusters of RBPs with more than one member in the GWS of 19 E. coli strains have been listed in this table. The RBPs were clustered based on BLASTP searches at E-value, percentage identity and percentage query coverage cut-offs of 10−5, 30 and 70, respectively. (DOC 376 kb) [file 12864_2017_4045_MOESM2_ESM.doc]

Additional File 1: Clusters of RNA-binding proteins obtained from 19 *E. coli* proteomes.

The clusters of RBPs with more than one member in the GWS of 19 *E. coli* strains have been listed in this table. The RBPs were clustered based on BLASTP searches at E-value, percentage identity and percentage query coverage cut-offs of 10^-5^, 30 and 70 respectively.

| **Cluster name** | **Number of members** | **UniProt IDs of members** |
| --- | --- | --- |
| Cluster 1 | 19 | A0A023YRI6, A0A025CL99, D3QVF6, A0A070D5J7, A0A0E0Y474, ACT69969, ACT30547, ACI36677, Q8FL80, Q1RGC7, AID77128, C8UGT9, C8U1E0, C8TGJ9, P33595, E3PCC2, A0A0E2TV05, A7ZHG1, B7UIC0 |
| Cluster 2 | 16 | A0A023YRM4, Q8FL61, D3QVI4, AID77155, ACT69996, ACI38050, A0A025CRA5, B7UIE8, P10408, ACT30518, A7ZHI9, A0A0E0Y7G6, A0A070DDX9, C8TGM9, A0A0E2U812, E3PCF0 |
| Cluster 3 | 19 | A0A023YRP0, Q1RGH9, P0A7U8, P0A7U7, E3PBB3, D3QVA7, C8UFZ5, C8U196, C8TGF1, B7UI68, AID77080, ACT69919, ACT30591, ACI38560, A7ZHB2, A0A0E2TQS8, A0A0E0Y4B9, A0A070D7K0, A0A025CK82 |
| Cluster 4 | 19 | A0A023YRS2, A0A025CLI6, D3QWE9, ACT70043, ACI39168, P37025, E3PCJ7, C8UH15, C8U1L7, C8THH4, A7ZHN8, A0A0E0Y548, A0A070DDT1, A0A0E2U1U7, B7UIJ1, ACT30471, AID77212, Q1RG43, A0A0H2V4S8 |
| Cluster 5 | 1459 | A0A023YRT5, A0A025CR75, ACT30550, A7ZHF7, C8UGT6, C8U1D7, C8TGJ6, A0A0E2TQL3, A0A0E0Y3Y0, D3QVF2, ACT69965, ACI38545, B7UIB7, Q8FL82, AID77125, P31548, E3PCB9, Q1RGD0, A0A0E2U8N0, A0A0E0Y378, A0A025C7U4, A0A023YUA8, P31134, E3PI85, ACT29761, D3QNS3, B7UMU7, ACT70812, ACI39613, A0A070D373, C8UL79, C8U4D6, C8TLX1, AID77879, Q1RE70, A0A0H2V5R1, A7ZJS3, Q8FB37, E3PC00, AID81216, A0A0E2TZ68, A0A025CA57, A0A023Z693, Q1R3Q1, P68187, D3QK42, C8UKU0, C8TZW2, C8TMS6, B7UPJ6, ACT74798, ACT30985, ACI38753, A7ZUQ7, A0A0E0Y6W8, A0A070D9K8, A0A070DY22, P77481, E3PL78, C8U7D6, C8TR17, ACT29326, A0A0E2TS45, A0A0E0XZM1, B7URC6, A7ZLE7, D3QS77, ACT71672, ACI37755, A0A025CV30, A0A023YWS8, Q8FHR3, AID78468, Q1RC47, C8UPB6, P14175, E3PH47, D3QPN0, C8UEN5, C8U9B0, C8TFS5, ACT28108, A7ZQA8, A0A0E2TGH9, A0A0E0XWW2, A0A070CHU8, B7UH97, Q1R821, AID79717, A0A0H2VBD7, A0A025CYV8, A0A023Z1B4, B7UGC2, ACT73385, ACI37270, A0A0E2TUW3, AID78219, P69875, P69874, E3PKD6, D3QQB9, C8U5Z0, B7UQ35, ACT71327, ACI39804, A0A0E0Y1S8, A0A025BQP1, A0A023YWK3, Q1RD28, P10346, E3PI39, ACT29834, Q1REB6, D3QNM5, C8UL27, C8U482, C8TLS5, B7UM05, AID77833, ACT70765, ACI37777, A7ZJM4, A0A0H2V661, A0A0E2U8R9, A0A0E0Y348, A0A070D587, A0A025C8U7, A0A023YUE4, C8UDZ7, P16676, E3PF23, A0A070CF05, A0A025D189, A0A023Z1F5, C8U7V1, C8TVC4, A7ZPM2, A0A0E0XZJ2, Q8FFB3, Q1R8U9, AID79528, ACT73136, ACI37136, D3QM09, ACT28324, A7ZKR6, A0A0E2TQQ6, C8UNQ6, C8TQ28, ACT29496, A0A070DRB4, Q1RBX6, B7URI7, AID78532, A0A0H2V778, A0A025C842, A0A023YXY1, A7ZLN4, A0A0E0XZS2, A0A070D0Y9, P77795, C8UPN5, C8U8B7, C8TRG5, ACT29230, A0A0E2TH25, E3PLU2, D3QSY9, ACT71746, ACI36931, D3QJD0, ACT70300, ACI37751, A0A025CAT2, A0A023YSR8, B7UL01, Q1R5H8, Q8FCQ2, AID80584, P37009, C8U4E5, P0AAF7, P0AAF6, E3PI94, D3QNT5, C8UL88, C8TLY0, B7UMV6, AID77888, ACT70824, ACT29752, ACI38289, A7ZJT3, A0A0E2TR79, A0A0E0Y1H1, A0A070DBM9, A0A025CAD9, A0A023YUE9, C8UGM9, C8TXQ8, C8TII0, A0A0E2TXC6, D3QSY3, ACT73976, ACI39382, A7ZSG7, A0A070D788, Q1RE61, ACT74127, ACI38343, A0A025BLR5, A0A023Z3S8, A0A070D996, A0A025CPD0, A0A023Z439, P10907, E3PLQ2, C8UHT0, C8TJN9, A0A0E2TML7, A0A0E0XUQ3, D3QU52, A7ZSY2, B7UK02, AID80410, A0A0H2VBN0, P45769, E3PKW2, ACT27544, A0A0E2TI70, A0A0E0XX01, B7UJJ6, A0A0E0Y3B1, Q1R657, Q47538, A0A025CCG7, A0A023YTB5, ACT70307, ACI36543, C8U256, D3QJD7, A0A070D4U2, E3PEU1, ACT30295, C8UIF6, C8TIW2, ACT27367, Q1RFH8, C8TY66, Q1RDS4, Q8FKF5, AID77461, C8TT92, C8UBW5, C8ULF6, C8U4L3, C8TM49, A0A0E2TSR7, A0A0E0Y199, Q8FJ95, AID77950, A0A070DBE0, P0AAI1, E3PIV4, D3QPD0, ACT70894, ACT29684, ACI37700, A7ZK25, B7UN19, Q1RER6, B7UKT5, A0A025CHH7, A0A023YU92, A0A0E0XXM6, E3PAU2, C8UC88, C8U3K2, C8TTJ8, A7ZN42, A0A0E2U4S2, P37774, ACT28776, A0A070CPC3, A0A025C275, A0A023YYY4, AID77699, A0A0H2V794, D3QWT8, ACT72328, ACI36552, B7UST6, AID78999, A0A0H2V9G0, Q1RAM3, E3PDF2, P30750, Q1RFY9, P63355, C8UH66, C8U1R7, C8THM6, B7UJA1, AID77265, ACT70093, ACT30419, ACI38162, A7ZHU1, A0A0E0Y3V2, A0A070D6Z1, A0A025CRK8, A0A023YS77, A0A0E2TJY2, P0AAG3, E3PGR1, D3QLU3, C8UJW8, C8U314, C8TKH3, ACT70577, ACI34611, A7ZJ43, A0A0E2TT81, A0A0E0Y3X9, A0A025CGK6, A0A023YT84, A0A070D5K3, D3QWK0, D3QUU9, A0A025CQD8, A0A023YY75, Q1RB36, ACT72135, ACI36811, P76909, E3PNE0, ACT28935, AID78796, A0A0H2V938, Q1R2T6, Q8FDZ8, A0A070D148, C8UBL0, C8TSS2, A0A0E0XYK2, D3QLN1, ACT70516, ACI38323, C8UA19, A7ZMM5, A0A0E2U616, D3QQ20, ACT71067, ACI37784, A0A025CGR3, A0A023YTF1, P15031, C8TPA3, AID81649, ACT30741, A7ZVG6, A0A0E0Y5W9, A0A025BU49, A0A023Z2R0, P23878, E3PGJ7, C8UJQ7, C8U2V2, C8TKB1, ACT30074, A7ZIX2, A0A0E2TQJ4, A0A0E0Y5Z3, A0A070DCA7, Q1REX4, B7UKN1, AID77644, A0A0H2V546, E3PIP7, Q1R223, ACT75179, ACI34454, B7USD7, C8U672, ACT71791, ACI36716, C8UPS4, A0A0E2TQN1, A0A070D2G1, D3QT30, D3QJR5, ACT72783, ACI36865, A0A025C810, A0A023YX48, AID79271, A0A0H2V908, P77622, E3PLY3, C8U8F6, B7UFE6, P75957, E3PJT0, C8UNI0, C8TPI7, A7ZKM1, A7ZNU6, C8URC0, C8UB11, C8TW29, A0A023Z7X5, A7ZLT2, A0A0E2TG12, A0A070DN59, A0A025BX20, C8TU54, A0A0E0XYD0, P33360, A0A025CKL1, A0A023Z0J2, B7UPD3, C8UD61, ACT29192, E3PCS0, A0A0E2U9J6, A0A070CWV0, ACT28589, C8TY79, A0A0E0XZN7, Q1R5F7, P0A9R8, P0A9R7, E3PM53, D3QU68, C8UHU3, C8TJQ2, B7UL14, AID80597, ACT74144, ACT27354, ACI37876, A7ZSZ9, A0A0E2U4F7, A0A0E0XUP1, A0A070D7H7, A0A025CV80, A0A023Z4V9, Q1R9U4, B7UJT5, Q1R6D9, P63387, P63386, E3PKP3, D3QSR5, C8UGF3, C8TXJ0, C8TIA3, AID80336, ACT73905, ACT27617, ACI36781, A7ZS94, A0A0E2TTU5, A0A0E0XVC5, A0A070D8M2, A0A025CGG8, A0A023Z3E9, C8UL12, C8TLQ9, A0A0E0Y366, Q8FIM7, Q1RD37, C8U5S2, AID78210, ACT29506, A0A0E2TLF1, A0A0E0Y0A9, AID77818, ACT70749, ACI37617, A0A0E2TT48, A0A025C8W7, A0A023YU34, B7ULZ1, P0A9U1, E3PI24, C8U467, A7ZJK5, A0A070D3T3, D3QKB2, ACT74889, ACI38320, Q1RED3, D3QNL0, A7ZSA0, ACT29849, A0A0H2V5F3, A0A025C7N9, A0A023YVF1, D3QQB2, A0A023YS21, A0A025CKK0, D3QWF3, C8UH19, C8THH8, B7UIJ8, AID77216, ACT30467, ACI37343, A0A0E2TK21, A0A070D741, P07821, E3PCK1, A7ZHP3, A0A0E0Y543, ACT70047, C8U1M1, Q1RG37, A0A0H2V482, D3QRX8, B7UIT8, ACT73746, ACI34676, A0A0H2VB19, Q1R6V5, AID80172, B7UL60, Q1R597, Q8FCJ1, AID80646, A0A025CQG2, A0A023Z4G1, D3QUE4, ACT74220, ACI39805, Q1RA83, AID79145, A0A0H2VAW4, D3QQW4, Q1RCP6, B7UQ83, AID78348, A0A0H2V6V0, A0A025BK85, A0A023YVQ1, AID78305, A0A0H2V715, Q1RCU4, C8U033, C8TMZ0, A7ZUX5, A0A0E0Y8T4, A0A025C3H4, A0A023Z6T6, P16677, C8ULV4, E3PD25, ACT30911, A0A0E2TJ96, A0A070CEB8, ACT28982, Q8FAV1, AID81309, Q1R3F6, B7UPS9, A0A025CR84, A0A023YXK6, Q8FH28, Q1RB86, B7US48, AID78754, C8UBC8, C8U9S7, C8TSI3, E3PN94, A7ZMH7, A0A0E2U5Y1, A0A0E0Y079, A0A070D9E5, D3QUK3, ACT72087, ACI35653, P06611, B7UN00, C8U4J4, Q8FJB1, Q1RDU4, AID77932, P60752, E3PIT5, D3QPB1, C8ULD7, C8TM29, ACT70874, ACT29703, ACI37314, A7ZK03, A0A0E2TVV9, A0A0E0Y2U0, A0A070DBG5, A0A025CIA3, A0A023YUK5, C8UDB0, D3QJX2, ACT72842, ACI35753, A0A070CGF2, B7UFJ7, A7ZP04, A0A070CNG2, A0A0E2TTR7, C8U6C2, C8TM01, A0A025CEP9, A0A023YZY3, C8TUA5, A0A0E0XYF9, C8ULA9, C8U4G6, A0A0E0Y350, Q1R9N9, P33916, E3PCX2, ACT28539, AID79319, A0A0H2V901, A0A025CHD1, A0A023YU57, ACT29731, A0A0E2U8K8, A0A070D333, E3PIQ7, P23886, ACT70847, ACI37658, ACT71261, ACI34829, D3QP85, A7ZJV3, AID77908, A0A0H2V5T0, B7UMX7, D3QJ93, ACT70263, ACI38453, Q1RFK7, A0A025CVB4, A0A023Z513, C8UIB8, C8U216, C8TI20, A7ZI70, A0A0E0Y4I7, Q1RE28, E3PM69, B7UGY7, AID79628, A0A0H2V9R7, ACT28203, A0A0E0XVL7, Q1R8I8, C8U8W2, A7ZPZ0, P77509, A0A070CER1, Q1R4L0, P0AAH1, P0AAH0, E3PNS4, D3QWB7, C8UJB2, C8TYX2, C8TL87, B7UMJ0, AID80887, ACT74484, ACT31286, ACI34675, A7ZTT2, A0A0E2U247, A0A0E0Y801, A0A070DA00, A0A025C229, A0A023Z4Z0, AID77589, A0A0H2V4Y9, A0A0E0XU66, B7UL99, ACT74255, ACI37980, AID80681, A0A0H2VEJ7, A7ZT84, D3QUW7, A0A025BX28, A0A023Z482, A0A0E2TME7, C8UI22, C8TJY1, A0A070D9H1, P37313, E3PMD0, ACT27272, Q1R557, C8TYG5, Q1RE96, B7UM24, A7ZJP6, Q8FJL0, AID77851, D3QNP5, ACI39182, ACT70784, P75796, ACT29814, A0A0E2TRB3, E3PI59, C8UL54, C8U4B0, C8TLU5, A0A0E0Y1K8, A0A025C827, A0A023YUJ2, A0A023YSA1, P36879, E3PCH7, C8UGZ6, C8U1J7, C8THF5, ACT30491, A7ZHL6, A0A0E2TUV8, A0A0E0Y569, A0A025CKI0, Q1RG64, AID77189, A0A0H2V455, D3QVL2, ACT70023, ACI37933, A0A070DDU6, B7UIH5, A0A0E2TR54, Q1RE44, P75831, B7UMX1, D3QP79, ACT70841, ACI34695, A0A025CDQ9, A0A023YU38, C8ULA3, C8TLZ5, ACT29737, A0A070DBL0, A0A0E0Y1F6, C8U4G0, C8UHW5, C8TJS4, ACT74190, ACI37462, A0A025CQE0, A0A023Z4E4, A7ZT22, A0A0E0XWD1, B7UL37, D3QUB5, A0A0E2TMQ3, E3PM78, P37624, ACT27330, A0A070D7P4, C8TYA8, AID80624, A0A0H2VBM6, Q1R5C8, P04983, D3QX51, B7UML4, A0A070DA59, A0A025BZF8, A0A023Z502, E3PNU8, Q1R4I3, C8TYU8, A7ZTW0, A0A0E0Y7L7, C8UJE0, C8TL59, ACT31261, A0A0E2TKE8, Q8FBS3, AID80911, ACT74516, ACI39042, A0A023YSB7, P0A9T8, E3PFW0, D3QKI7, C8UIS9, C8TJ88, ACT70448, ACT30161, ACI37883, A7ZIQ5, A0A0E2TIY0, A0A0E0Y2M4, A0A070D4P9, A0A025CAI2, B7UKH8, C8U2I1, Q1RF33, AID81768, Q8FCM9, Q1R5D8, B7UL32, AID80614, C8TY96, A7ZT18, ACT27337, C8UHW0, C8TJR9, A0A0E0XTA6, P33594, E3PM70, A0A070D7P9, A0A0E2TMP9, A0A025CRW7, A0A023Z4I4, D3QUA3, E3PPG8, ACT74178, ACI38344, Q1RCJ0, B7UQD6, P76027, D3QR15, AID78400, ACT71477, ACI36269, A0A0H2V700, A0A025CD73, A0A023YW78, E3PKN2, C8UNZ9, C8U706, C8TQQ5, ACT29402, A7ZL17, A0A0E2U7A3, A0A0E0Y001, A0A070DRM5, B7UPP7, AID81276, A0A0H2VD40, A0A0E0Y8B4, Q1R3I6, A0A070D7S3, P0AAG0, E3PMD1, D3QUW8, C8TYG6, ACT74256, ACT27271, ACI37253, A7ZT85, A0A0E0XSK3, A0A025BXW1, A0A023Z4A2, A0A0E2TIQ7, C8TJY2, C8UPS5, A0A0E0Y0S8, C8TRK4, ACT71792, ACI35112, C8UI23, A0A025CBI5, A0A023YXC2, A0A023YSC3, P77279, E3PFV5, A0A025C9N6, D3QKI2, C8U2H6, ACT70443, ACI35506, A7ZIQ0, A0A0E0Y2N0, A0A070D656, Q1RF38, B7UKH3, ACT30166, C8UIS4, C8TJ83, A0A0E2TIX7, A0A0H2V4Y5, AID77585, Q1RDF9, AID78093, A0A0H2V707, E3PBG1, Q1RAG3, C8TTP6, A0A0E0XXQ9, A0A023YSW8, A0A025CFS2, C8U2D5, A7ZIK6, ACT30207, A0A070DD00, A0A0E0Y330, D3QJL9, ACT70393, ACI36211, E3PFR4, P77265, C8TJ41, C8UIN2, A0A0E2TIT3, AID77544, A0A0H2V4W0, B7UJS1, Q1RF87, Q1RAG2, E3PBG2, AID79081, A0A0E0XYV9, A0A0H2V8I8, C8TTP7, AID79080, A0A0H2V8B0, A0A023YT43, P0AAG6, P0AAG5, E3PFR5, AID77545, ACT70394, ACI36125, A0A070D4I0, A0A025C9X1, C8UIN3, C8U2D6, C8TJ42, ACT30206, A7ZIK7, A0A0E0Y469, D3QKD5, A0A0E2U071, B7UJS2, Q1RF86, Q1R9A7, D3QKZ9, C8UDQ7, C8U6M9, C8TUV1, B7UFW4, AID79437, ACT72960, ACI35405, A7ZPC1, A0A0H2V9B3, A0A0E2U909, A0A0E0XXX8, A0A070CPN1, A0A025CGR7, A0A023Z0K1, P07109, E3PE11, ACT28416, A0A025C578, A0A023Z6B7, B7UPS1, AID81301, D3QKA4, ACT74881, ACI39338, Q1R3G4, A0A0H2VDA1, P16678, E3PD17, ACT30919, C8U025, A0A0E2U105, C8ULU6, C8TMY2, A7ZUW7, A0A0E0Y6R5, A0A070CFA0, Q1R6D3, P0A9V2, P0A9V1, E3PKP9, D3QSS1, C8UGF9, C8TXJ6, C8TIA9, B7UJU1, AID80342, ACT73911, ACT27611, ACI36932, A0A0E0XX65, A0A070D6S3, A0A025CHE6, A0A023Z342, A0A0E2TSW8, A0A0E2TP63, Q1RAS6, P0A9X2, P0A9X1, E3PAG1, D3QVX5, C8UBX2, C8U383, C8TT99, B7USN5, AID78892, ACT72243, ACI39107, A7ZMY2, A0A0E0XZM7, A0A070D9X4, A0A025CNH4, B7UQN9, A0A023YTU2, A0A025CFL6, A0A0E0Y3P0, P31060, E3PHE2, C8UK52, C8TKQ8, A0A070D3W0, A7ZJD5, A0A0E2TT78, A0A0H2V7F9, C8U3Y9, D3QMU6, ACT70672, ACI34775, Q1REH1, AID77783, B7ULN3, AID80589, A0A0H2VBJ6, B7UL06, C8U015, C8UEB5, C8TVP2, D3QK96, AID81291, A0A0H2VDI5, Q1R3H4, ACT74873, ACI37566, P32721, ACT30929, A0A023YUA1, A0A025C8T2, Q1REA4, P0A9U4, P0A9U3, E3PI50, B7UM16, AID77843, ACT70777, ACT29823, ACI37655, A7ZJN6, A0A0E2U8R1, A0A0E0Y1M0, A0A070D580, D3QNN6, C8TLT5, C8UL45, C8U4A0, A0A0E2TM05, AID80485, A0A0H2VBH0, A0A070D8X3, B7UK63, P63389, E3PLG0, C8UHI4, C8TXW9, C8TIP1, ACT74037, ACT27467, ACI37418, A7ZSM8, A0A0E0XTL8, A0A025CQ11, A0A023Z3U0, D3QTH9, Q1R5T0, B7UN28, A0A0E2TQZ0, AID77959, A0A0H2V5V0, Q1RDR5, D3QPE5, ACT70910, ACI38405, C8ULH2, C8TM65, C8U5B3, A7ZK53, A0A0E0Y2Z1, A0A070D2X0, A0A025CID6, A0A023YUK6, P43672, E3PIX3, ACT29668, Q1R250, A0A0H2VFI8, A7ZVT3, P0A9W3, E3PFH3, D3QNF1, C8UN98, C8TQE4, B7UR21, AID81560, ACT75148, ACT30625, ACI39147, A0A0E2TSL6, A0A0E0Y7R6, A0A070DEA9, A0A025CL22, A0A023Z897, C8U163, AID81864, A0A0E0Y0R4, AID78072, A0A0H2V648, E3PLQ6, A7ZSY7, Q1RCI9, D3QR16, E3PKN3, C8UP00, C8U707, C8TQQ6, B7UQD7, AID78401, ACT71478, ACT29401, ACI36689, A7ZL18, A0A0H2V854, A0A0E2TQ19, A0A0E0Y1R0, A0A070DS31, A0A025C7B6, A0A023YVV5, P77737, Q1R556, B7ULA0, AID80682, A0A0H2VBW7, A0A070D0R6, P77268, ACT29191, A0A0E2U6G4, D3QT31, E3PLY4, A7ZLT3, C8U8F7, D3QRR7, ACT71646, ACI39502, A0A0E0Y3A6, Q1RC69, P0AAH9, P0AAH8, E3PL50, C8UP90, C8U7A9, C8TQZ0, B7URA5, AID78447, ACT29354, A7ZLB5, A0A0E2TNB5, A0A070DQK8, A0A025CWS4, A0A023YWE0, B7UPP8, AID81277, Q1R3I5, A0A0H2VDI0, Q1RC68, P0AAH5, P0AAH4, E3PL51, D3QRR8, C8UP91, C8U7B0, C8TQZ1, B7URA6, AID78448, ACT71647, ACT29353, ACI38861, A7ZLB6, A0A0E2TNU6, A0A0E0Y035, A0A070DQB6, A0A025D013, A0A023YX44, A0A0E2TMB3, C8TY95, ACT27338, A7ZT17, A0A070D965, Q1R5D9, B7UL31, P33593, C8UHV9, C8TJR8, A0A0E0XWD6, D3QUA2, ACT74177, ACI37906, Q8FCN0, AID80613, Q8FCE2, B7ULC8, AID80707, D3QUZ8, ACT74285, ACI38372, A0A070D9K5, A0A025CL07, A0A070CQB0, C8UC74, C8U3I8, C8TTD8, A7ZN25, A0A0E2TMT4, P0AAF4, P0AAF3, E3PAK0, D3QWS3, B7USS2, AID78986, ACT72283, ACT28792, ACI36879, A0A0E0XXN7, A0A070CPD8, A0A025CUH6, A0A023YYR9, A0A0E0XY48, A0A0H2VCT3, AID79964, Q1R3G5, AID81300, A0A0H2VFV6, Q1RAN8, E3PD16, Q1R5H4, A0A0H2VD85, A0A0E0XUP9, A0A070D7I6, D3QU57, C8UHT4, C8TY70, C8TJP3, B7UL05, AID80588, ACT74132, ACI35251, A0A0E2TM88, A0A025CPD5, A0A023Z3V7, P22731, ACT27363, ACT28831, ACT75009, P77257, A0A025BRC5, A0A023YWV5, E3PM13, D3QT62, ACT71820, ACI34568, A0A070D036, C8U8I4, A0A0E2TNI5, C8TRN2, A0A0E0Y0X0, A7ZLX1, A0A025C2U7, A0A023Z6A7, B7UPR1, A0A0E2U114, E3PD07, ACT27988, Q1R677, A0A0H2VCX0, AID80391, C8UGL0, C8TIG0, ACT30792, E3PE99, Q6BEX0, D3QLE7, A0A025CL80, A0A023Z6X5, A0A0E0Y4N9, C8TP49, A7ZVA7, A0A0E2TLQ6, AID81448, A0A0H2VGC9, C8U0J4, C8UM80, Q1R327, A0A0E2TTP8, ACT73257, ACI35544, D3QN38, A0A025CFM6, A0A023Z1D9, A0A023Z4C6, ACT72809, ACI36738, C8TZK0, A7ZTB4, A0A0E0XSH3, P37388, C8UIV5, C8TMG7, C8UD80, C8TU73, A7ZNX1, E3PMF6, D3QJU1, P0AAG8, E3PCT9, C8U691, A0A0E2TS91, Q8FFU7, B7UFG5, AID79289, Q1R9S4, A0A0E0XYB1, A0A025CH59, A0A023Z048, Q1R528, A0A070CQS5, ACT28570, E3PG91, A0A023YXP3, A0A025CQ42, D3QUH6, ACT72060, ACI35188, AID78727, A0A0H2V7V5, ACT29007, A0A0E2TSG6, C8UBA2, A7ZME7, A0A0E0XYX8, A0A070D381, P77499, E3PMQ7, B7US21, C8TSF7, Q1RBB4, C8U9Q1, A0A023YYA2, A0A023YZT5, C8UDE6, C8U6D9, C8TUD9, ACT28521, A0A0E0XWP0, A0A070CNE6, A0A025CGZ7, P33931, E3PDQ3, B7UFL4, A7ZP23, ACI35315, Q1R9L8, D3QJZ0, ACT72861, AID79338, Q8FFR2, A0A0E2TS01, A0A023Z2E3, A0A025CRN7, ACT73640, ACI36845, D3QR32, A7ZR42, C8UFD6, C8TGU2, A0A0E2TR25, Q1R7A1, A0A023Z2K3, D3QR31, A0A025CU81, C8UFD5, C8UAT3, C8TGU1, ACT73639, ACI38234, A0A0E2TU59, A0A0E0XWC3, A0A070CIH8, A7ZR41, B7UHX3, Q1R5H3, P0A9S7, D3QU58, C8UHT5, C8TY71, C8TJP4, ACT74133, ACT27362, ACI36258, A7ZSY8, A0A0E0XWF5, E3PLQ7, A0A025CV72, A0A023Z3X6, A0A0E2TMM2, A0A070D990, A0A023Z6M3, A0A025C2N4, P16679, C8U024, ACT30920, C8ULU5, C8TMY1, A7ZUW6, A0A0E0Y5E1, D3QKA3, ACT74880, ACI34873, A0A0E2TJ41, A0A070CDB8, B7UPS0, A0A023YSM6, A0A025C9P4, D3QKH4, ACT70434, ACI39448, AID77363, A0A0H2V4J2, A0A0H2VAR2, AID80088, Q1R743 |
| Cluster 6 | 19 | A0A023YRT8, A0A025CN66, C8U1N0, A7ZHQ2, A0A0E2TNM9, A0A0E0Y3Z0, E3PDB3, C8UH28, C8THI7, ACT30458, B7UIK7, P15723, Q8FL13, Q1RG28, AID77225, A0A070D5M0, D3QWG2, ACT70056, ACI35442 |
| Cluster 7 | 19 | A0A023YRU8, Q1RG19, P0A6P2, P0A6P1, D3QWH0, C8THJ6, B7UIL5, AID77233, ACT70064, ACT30449, ACI38125, A7ZHR0, A0A0E2U1S6, A0A0E0Y580, A0A070D785, A0A025CRH3, C8U1N8, C8UH37, E3PDC2 |
| Cluster 8 | 19 | A0A023YRW4, Q1RGA1, P0ABH1, P0ABH0, E3PCE6, D3QVI0, C8UGW5, C8U1G6, C8TGM5, B7UIE4, AID77151, ACT69992, ACT30522, ACI35676, A7ZHI5, A0A0E2TS81, A0A0E0Y448, A0A070D5G7, A0A025CMZ0 |
| Cluster 9 | 19 | A0A023YRW5, E3PDD6, ACT30435, A0A070D764, A0A025CKS2, P10443, C8U1Q2, A7ZHS4, A0A0E0Y3L7, C8UH51, ACT70078, ACI37310, A0A0E2TJT2, C8THL0, D3QWI4, Q1RG05, Q8FL05, B7UJ85, AID77246 |
| Cluster 10 | 19 | A0A023YRW7, A0A025CL88, Q1RGE0, P60240, E3PCB2, D3QVE5, C8U1D0, C8TGI9, ACT69958, ACT30557, A0A0E2TV13, A0A0E0Y484, A0A070D5K8, A7ZHF0, C8UGS9, Q8FL92, B7UIA5, AID77116, ACI36436 |
| Cluster 11 | 18 | A0A023YS03, ACI35994, D3QVI3, ACT30519, A7ZHI8, Q1RG98, P62396, AID77154, ACT69995, A0A025CKJ2, P62395, C8UGW8, C8U1G9, C8TGM8, A0A0E2TQJ3, A0A0E0Y5F0, A0A070D782, E3PCE9 |
| Cluster 12 | 19 | A0A023YS05, A0A025CKC8, C8TGF4, A7ZHB5, C8UFZ8, P00956, C8U199, A0A0E0Y5N7, A0A070DE68, D3QVB0, ACT30588, E3PBB6, ACT69922, ACI39266, Q8FLB7, AID77082, A0A0E2TSH1, B7UI71, Q1RGH6 |
| Cluster 13 | 19 | A0A023YS11, A0A025CKN4, C8U1L3, B7UII7, AID77205, ACI37370, A0A0E2TKL6, C8UH11, C8THH0, A7ZHN4, A0A070D5B8, P0ABF2, P0ABF1, ACT70039, E3PCJ3, D3QWE5, ACT30475, A0A0E0Y3Q9, Q1RG47 |
| Cluster 14 | 38 | A0A023YS12, A0A025BW14, A0A0E2TIT9, C8UI56, C8U1W6, C8THV3, A0A0E0Y3G4, A7ZHZ4, AID77377, A0A0H2V4J1, Q1RFT6, B7UJC3, A0A023YSV5, A0A025C1K3, A0A070DFR7, C8UHQ1, C8TJL0, A0A025CRQ7, A0A023Z3R4, Q1R5L4, P46850, E3PLM6, ACT27396, A0A0E2TN69, A7ZSV1, ACT74099, ACI36599, D3QTP0, A0A0E0XWI4, AID80547, A0A0H2VE71, B7UKC5, ACI36320, ACT70156, D3QWQ7, P75675, E3PDL4, ACT30386 |
| Cluster 15 | 135 | A0A023YS16, D3QWF0, ACI35388, A0A025CKN9, Q1RG42, A0A0H2V477, ACT70044, A0A0E2TKL1, C8UH16, C8THH5, A0A070D5B2, ACT30470, A0A0E0Y3Q3, E3PCJ8, C8U1L8, A7ZHN9, P37024, AID77213, B7UIJ2, AID81681, A0A023YU60, A0A025CE05, P25888, E3PI27, ACT29846, A0A0E2TRE0, A0A0E0Y5B0, A0A070D3H7, C8U470, A7ZJK8, C8TLR2, C8UL15, D3QNL3, ACT70752, ACI35192, AID77821, A0A0H2V5F2, Q1RED0, B7ULZ4, D3QSN4, ACT73874, ACI39130, A0A025CGD7, A0A023Z3I5, C8UGC1, C8TI72, P0A9P7, P0A9P6, E3PK74, C8TXF9, B7UJ57, AID80298, ACT27648, A0A0E2TXL4, A0A0E0XTN5, A0A070D6Y7, A7ZS57, Q1R6H8, A0A025BZZ7, A0A023Z5Z3, Q1R4F9, P0A8J9, P0A8J8, E3PNX0, C8TYS6, B7UMN5, AID80932, ACT31241, A7ZTY2, A0A0E2U3B2, A0A0E0Y5X9, A0A070DA48, D3QX73, ACT74539, ACI36339, C8UJG2, C8TL37, A0A0H2V8C0, AID78492, Q1RC22, B7URE8, P21693, E3PLA4, ACT29301, D3QSA3, ACT71699, ACI38056, P21507, Q1R8F6, E3PGW6, D3QNB8, C8UEE2, C8U946, C8TVS1, B7UH16, AID79657, ACT73288, ACT28170, ACI36622, A7ZQ21, A0A0H2V9T0, A0A0E2TUE0, A0A0E0XXA6, A0A070CHG2, A0A025CD35, A0A023Z1H8, C8UPE2, C8U7G1, C8TR43, A7ZLH3, A0A025CU21, A0A023YWN7, A0A0E2TPN5, A0A070DXZ4, A0A0E0Y357, A0A023YX63, A0A025C5S4, D3QSH9, A0A0E2TZ47, A0A070D2A4, ACT71718, ACI39551, ACT29258, A7ZLK9, P43329, AID78511, Q1RC01, A0A0H2V7F8, C8U891, C8UPL1, A0A0E0Y326, E3PLR4, C8TRE0, B7URG2, AID81783 |
| Cluster 16 | 75 | A0A023YS34, P0AA37, E3PCB1, C8U1C9, ACT30558, A7ZHE9, A0A0E2TSB6, A0A0E0Y7K6, A0A025CKB6, Q8FL93, AID77115, C8UGS8, C8TGI8, Q1RGE1, B7UIA4, A0A070DE29, D3QVE4, ACT69957, ACI34864, Q8FIP7, AID78181, ACT71229, ACI39043, A0A025C6T2, A0A023YW94, Q1RD71, P0AA39, E3PJQ0, C8UNF0, C8U5P1, C8TPF6, ACT29537, A7ZKJ0, A0A0E2TLS2, A0A0E0Y1Y7, A0A070CEE1, B7UPA2, D3QQ81, C8TVT5, P65834, AID79671, A0A0E0XX90, C8UEF5, C8U961, A7ZQ36, A0A070CHE8, A0A025CSH2, A0A023Z1K1, D3QNV3, ACT73303, ACI35468, A0A0E2TZH1, B7UH29, Q1R8D1, P33643, E3PGY0, ACT28156, Q1R7Q1, C8UEZ8, C8UAC3, C8TG38, A7ZQN4, P0AA41, E3PHU8, D3QQC4, B7UHK7, ACT28004, A0A0E2TS16, A0A070CHI8, A0A025D1U4, A0A023Z1T1, ACT73493, ACI35848, AID79825, Q8FEF9 |
| Cluster 17 | 19 | A0A023YS49, Q1RGC5, P0A6A7, P0A6A6, E3PCC5, C8UGU3, C8U1E4, C8TGK3, AID77131, ACT30543, A0A0E2TSA1, A0A070D7A9, A0A025CMW6, B7UIC2, A0A0E0Y7J3, D3QVF8, ACT69971, ACI36136, A7ZHG4 |
| Cluster 18 | 19 | A0A023YS70, C8U1R2, C8THM1, ACT30424, A0A0E2TJY7, A0A025CRK2, C8UH61, A7ZHT6, A0A070D6Z7, A0A0E0Y3V8, D3QWJ5, ACT70088, ACI37382, E3PDE7, P16659, Q8FKZ4, Q1RFZ4, B7UJ96, AID77260 |
| Cluster 19 | 19 | A0A023YS85, Q8FK44, Q1RF08, E3PGF8, C8U2K7, AID77613, ACT30132, A7ZIT5, A0A0E2TP73, A0A0E0Y3Z7, A0A070D649, A0A025C9N9, P21888, D3QKL6, ACT70475, ACI35904, B7UKK3, C8UJK0, C8TJB2 |
| Cluster 20 | 19 | A0A023YS92, ACI34931, A0A0E2TRI9, A0A025CLF4, P36683, E3PCG8, C8UGY7, ACT30500, A7ZHK7, A0A0E0Y3T2, ACT70014, Q1RG73, A0A0H2V4N5, A0A070D5D6, C8U1I8, C8THE6, D3QVK3, AID77180, B7UIG6 |
| Cluster 21 | 19 | A0A023YS97, A0A025CLM4, D3QWI3, ACT70077, ACI39235, A0A070D578, E3PDD5, B7UJ84, ACT30436, Q8FL06, AID77245, Q1RG06, P10442, C8UH50, C8U1Q1, C8THK9, A7ZHS3, A0A0E0Y4Z6, A0A0E2TJZ4 |
| Cluster 22 | 19 | A0A023YSA2, A0A025CLM9, D3QWI8, ACT70082, ACI38537, P52097, A0A070D573, C8UH55, C8U1Q6, C8THL4, A7ZHS8, A0A0E0Y4Z3, B7UJ89, E3PDE0, Q1RG01, ACT30431, Q8FL00, AID77250, A0A0E2TNL3 |
| Cluster 23 | 19 | A0A023YSD9, P06992, E3PCA5, C8UGS3, C8U1C4, C8TGI3, ACT30563, A0A0E2TSC0, A0A070DE35, A0A025CMU1, D3QVD8, ACT69951, ACI36216, A7ZHE4, A0A0E0Y7L9, Q8FL96, Q1RGE6, B7UI99, AID77110 |
| Cluster 24 | 19 | A0A023YSE2, Q8FKL1, Q2EEQ2, Q1RFQ0, E3PEM6, D3QXR6, C8UI89, C8U1Y8, C8THZ2, B7UJE4, AID77397, ACT70218, ACT30361, ACI35922, A7ZI30, A0A0E2U427, A0A0E0Y4T8, A0A025BY06, A0A070D598 |
| Cluster 25 | 19 | A0A023YSE5, Q1RG21, P0A7V1, P0A7V0, E3PDC1, D3QWG9, B7UIL4, AID77232, ACT70063, ACT30450, ACI38188, A0A025CKQ7, C8UH36, C8U1N7, C8THJ5, A7ZHQ9, A0A0E2TJU8, A0A0E0Y3N4, A0A070DDR4 |
| Cluster 26 | 19 | A0A023YSF5, A0A025BVH1, Q1RFS8, P0A7B6, P0A7B5, E3PDM0, D3QWR4, C8UI63, C8U1X3, C8THW0, B7UJD0, AID77384, ACT70163, ACT30377, ACI37364, A7ZI01, A0A0E2TMM9, A0A0E0Y6Y4, A0A070D525 |
| Cluster 27 | 70 | A0A023YSG8, E3PDE4, A0A025CKN8, D3QWJ2, C8THL8, A7ZHT2, A0A0E2TKI2, A0A0E0Y3W0, B7UJ93, AID77254, A0A0H2V496, A0A070DDP2, C8U1R0, P40711, ACT30427, Q1RFZ7, C8UH59, ACT70085, ACI36812, A0A0E2TPZ7, Q1RCM7, P0A7I1, P0A7I0, E3PKJ8, D3QQY4, C8UNW7, C8U6X3, C8TQM3, AID78364, ACT71442, ACT29436, ACI37237, A7ZKY5, A0A0E0Y0G4, A0A025C7E3, A0A023YW16, B7UQ99, A0A070DR57, P07012, P66023, E3PIG7, C8UF97, C8UAP5, C8TGQ3, B7UHU0, ACT27914, AID79928, A0A0E2TQ87, A0A0E0XYB7, A0A070CH98, A0A025CCP0, A0A023Z2E9, A0A023YSK7, A0A025BXY8, A0A0E0Y506, A7ZHZ5, A0A070DDJ3, C8U1W7, A0A0E2TIX6, Q1RFT5, AID77378, A0A0H2V574, C8UI57, C8THV4, B7UJC4, P28369, ACT30385, D3QWQ8, ACT70157, ACI37121 |
| Cluster 28 | 19 | A0A023YSI6, Q1RFX6, A0A0H2V657, ACT30409, A0A025BVW8, P03007, D3QWL0, ACT70103, ACI39727, C8UH76, C8U1S7, C8THN6, A7ZHV2, A0A0E2U3Y9, A0A0E0Y3U3, A0A070D6Y5, E3PDG2, AID77275, B7UJB1 |
| Cluster 29 | 39 | A0A023YSL8, A0A025CRE6, D3QWE6, ACT70040, ACI38012, P27305, E3PCJ4, C8U1L4, A0A0E2TNP0, A0A070D7A6, ACT30474, C8UH12, C8THH1, A0A0E0Y597, A7ZHN5, Q1RG46, B7UII8, Q8FL25, AID77206, Q1R8W9, P04805, E3PF04, C8UDX9, C8U7T3, C8TVA6, ACT28341, A7ZPK7, A0A0E0XWE6, Q8FFC9, B7UGA5, AID79511, A0A0E2TUY1, A0A070CF20, A0A025D1A1, A0A023Z0G6, D3QLZ4, ACT73121, ACI34975, AID77209 |
| Cluster 30 | 20 | A0A023YSN4, P0A7F9, E3PFM2, D3QJH3, C8TIZ5, ACT70347, ACT30256, ACI39129, A7ZIF7, A0A025CFV8, C8U289, A0A0E2TI99, A0A0E0Y6E5, A0A070D6F2, C8UII6, B7UJM8, Q8FKC5, AID77495, Q1RFD6, AID81726 |
| Cluster 31 | 19 | A0A023YSN6, P0AAR3, E3PFU6, D3QKG6, C8UIR5, C8U2G7, C8TJ74, B7UKG1, ACT30175, A7ZIP1, A0A0E2U0A6, A0A0E0Y4C7, A0A070D667, A0A025C9V2, Q1RF52, AID77575, A0A0H2V708, ACT70425, ACI38279 |
| Cluster 32 | 19 | A0A023YSP6, Q1RG17, P0A806, P0A805, E3PDC4, D3QWH2, C8UH39, C8U1P0, C8THJ8, B7UIL7, AID77235, ACT70066, ACT30447, ACI36869, A7ZHR2, A0A0E2TNM1, A0A0E0Y3Y1, A0A070D721, A0A025CKL9 |
| Cluster 33 | 19 | A0A023YST7, Q1RFX7, P0A7Y5, P0A7Y4, E3PDG1, D3QWK9, C8UH75, C8THN5, ACT70102, ACT30410, ACI35007, A7ZHV1, A0A0E2TLU8, A0A070D5H1, A0A025BYC4, C8U1S6, A0A0E0Y746, AID77274, B7UJB0 |
| Cluster 34 | 20 | A0A023YSU7, A0A025BTD6, B7UJH1, P77736, C8U208, ACT30339, A7ZI61, A0A0E2TQN2, A0A0E0Y6N2, A0A070D6T2, Q1RFL5, C8UIB0, E3PEP5, C8TI12, AID77428, A0A0H2V5B2, D3QXU6, ACT70253, ACI39440, A0A023YT49 |
| Cluster 35 | 19 | A0A023YSV2, P0A850, E3PFQ2, D3QJK7, C8UIM0, C8U2C3, B7UJQ9, ACT70381, ACT30221, ACI35949, A0A0E0Y4G5, A0A070D4W5, A0A025C9S5, A7ZIJ4, Q8FKA7, C8TJ29, AID77531, A0A0E2TIE8, Q1RFA0 |
| Cluster 36 | 19 | A0A023YSW3, A0A025CCC5, B7UJP6, P77718, E3PFP0, Q8FKB7, Q1RFB7, AID77517, C8UIK4, C8U2A7, A0A0E0Y2V3, ACT30238, A7ZIH7, A0A0E2U041, A0A070D6E9, C8TJ13, D3QJJ4, ACT70368, ACI35854 |
| Cluster 37 | 38 | A0A023YSW9, A0A025CCD2, AID77508, A0A0H2V4V3, A0A0E2TI97, Q1RFC7, C8UIJ5, C8TJ04, P25539, E3PFN1, C8U298, B7UJN7, A0A0E0Y4I5, A0A070D4Z5, A7ZIG7, ACT30247, D3QJI4, ACT70358, ACI34546, A0A023Z140, Q1R8H5, D3QN50, E3PGA3, A0A0E0XZ60, A7ZQ02, Q8FF24, B7UGZ9, AID79640, ACT73269, ACI39004, A0A025CD79, P68398, C8UEC5, C8TVQ4, ACT28189, A0A0E2TV23, A0A070DYI3, C8U8X4 |
| Cluster 38 | 19 | A0A023YSY5, ACI35962, D3QKF9, Q1RF61, P69441, E3PFT9, C8UIQ8, C8U2G0, C8TJ67, ACT70418, ACT30182, A7ZIN4, A0A0E2TM70, A0A0E0Y445, A0A070D694, A0A025CC87, Q8FK84, B7UKF4, AID77568 |
| Cluster 39 | 19 | A0A023YT00, Q1RFC4, P0A781, P0A780, E3PFN3, D3QJI6, C8UIJ7, C8U2A0, C8TJ06, B7UJN9, AID77510, ACT70360, ACT30245, ACI35692, A7ZIG9, A0A0E2TM19, A0A0E0Y367, A0A070DD46, A0A025C9Z9 |
| Cluster 40 | 20 | A0A023YT07, Q1RFD5, P0A848, P0A847, D3QJH4, C8UII7, C8U290, B7UJM9, AID77496, ACT70348, ACT30255, ACI38297, A7ZIF8, A0A0E2TM09, A0A0E0Y377, A0A025CA06, E3PFM3, A0A070DD59, C8TIZ6, AID81727 |
| Cluster 41 | 159 | A0A023YT09, A0A025CLU9, C8UJP9, C8U2U4, C8TKA3, D3QLM4, C8UJQ0, C8U2U5, C8TKA4, AID77637, ACT70509, ACI34717, A0A0E2TR33, A0A0E0Y3W9, A0A070D4B3, A0A025CID0, B7UKM4, P77091, E3PGI9, C8UJP8, C8TKA2, B7UKM5, ACT30083, A0A0E2U6V9, A0A070D5W2, D3QLM3, ACT70508, A0A0E0Y601, ACT28034, Q1R7T7, C8UEX0, C8U9J3, C8TG09, AID79797, ACT73463, A0A0E0XWN1, A0A070CQM3, D3QPV9, A0A0E2TRG9, B7UTI3, C8TWH1, P37305, B7ULB8, AID80697, ACT27255, A0A0E2TIP7, Q1R538, E3PME5, C8UI43, C8TZL0, C8TMH7, ACT74275, ACI38168, A7ZTA4, A0A0E0XSI3, A0A070DG72, D3QUY8, C8TWC0, A0A070CRJ7, C8TWC1, C8UQU5, A0A0E0Y834, A0A023Z4B9, C8URF9, ACT70951, B7UQG7, A0A0E2TKE0, A0A023YV47, ACT71106, ACI39214, A0A023Z2Q4, ACI34965, A0A0E0Y0W9, A0A025C8E6, A0A023Z0I4, A0A0E0XZE7, Q1RE19, A0A0E2TMP1, A0A070CWB4, A0A025C4R3, A0A023YYL3, D3QUQ5, A0A0E0XYP9, A0A0E2TI47, Q1R1U0, ACT75224, ACI34495, Q1RGI6, E3PBA6, P33236, C8UFZ0, C8U191, C8TGE6, ACT69913, D3QV99, ACI35876, P0ACG4, C8UFZ1, C8U192, C8TGE7, AID77074, ACT30597, A0A0E2TSI1, A0A070D5Q5, A0A025CL45, D3QRH8, C8UQ00, C8U8N2, C8TQT5, A0A070D2X8, A0A025BKY3, B7UI61, E3PLS0, A0A025C5S0, C8U3U4, C8TMB0, B7UQG6, P0ACG6, D3QX39, C8UQ01, C8U736, C8TRV1, C8U735, C8TPL3, ACT71284, ACT29121, C8U5U7, P77494, C8UPL6, C8U897, C8TRE6, A0A0E2TZ44, A0A0E0XZU2, D3QTR8, C8TR68, A0A070DP80, B7UI62, A0A0E0Y0E3, D3QSC5, A0A0E0Y7P7, C8ULL2, A0A070D4U8, A0A0E2TZI5, A0A025BK68, C8UCI9, C8U4Y9, C8TT42, A0A070CMK6, C8UML5, C8TT43, A0A0E2U3F1, A0A0E0XZX6, Q1RCG0, A0A070DZI8, D0Z6Q1, C8UR44, ACT71502, E3PPE3, A7ZGU5 |
| Cluster 42 | 19 | A0A023YT74, A0A025CGL5, P07813, E3PGQ0, C8UJV8, C8U304, C8TKG4, B7UKT2, A0A0E0Y3Y7, Q8FJY9, AID77696, A0A0E2TTB5, A0A070D3S4, D3QLT4, ACT70569, ACT30018, ACI35040, A7ZJ31, Q1RER9 |
| Cluster 43 | 132 | A0A023YTF4, Q1RET7, P0A973, P0A972, E3PGN2, D3QLR6, C8UJU0, C8U2Y6, C8TKE6, B7UKR5, AID77678, ACT70551, ACT30036, ACI38025, A7ZJ11, A0A0E2TPM2, A0A0E0Y5W0, A0A070D451, A0A025CI91, Q1RAW7, P0A9Y7, P0A9Y6, D3QVT9, C8UBS2, C8UA81, C8TSY4, B7USJ9, AID78860, ACT72207, ACT28869, ACI35961, A7ZMU5, A0A0E2TP32, A0A0E0Y1L7, A0A070D3L7, A0A025CNE2, A0A023YYW1, E3PNK8, D3QMJ3, P0A987, P0A986, C8TRU1, A0A0E2TNV9, A0A070DEF9, Q1R539, P0A9Y0, P0A9X9, D3QUY7, C8UI42, C8TZL1, C8TMH8, B7ULB7, AID80696, ACT74274, ACT27256, ACI39198, A7ZTA3, A0A0E2TMD1, A0A0E0XU46, A0A070D7T4, A0A025CIG6, A0A023Z4A1, Q1RDM9, P0A979, P0A978, E3PJ12, D3QPI4, C8UMC9, C8U5F2, C8TPT0, B7UN67, AID77990, ACT71008, ACT29627, ACI37107, A7ZK92, A0A0E2TMG9, A0A0E0Y145, A0A070CE10, A0A025C8H4, A0A023YUY2, A0A0H2V9S9, A0A070D5Y4, P36995, C8TRU6, D3QMI7, A7ZM19, A0A0E2TK78, Q1RE43, D3QP80, P0A969, P0A968, C8ULA4, C8U4G1, C8TLZ6, B7UMX2, AID77903, ACT70842, ACT29736, ACI39064, A7ZJU8, A0A0E2U8L3, A0A0E0Y355, A0A070D336, A0A025C7W1, A0A023YUD1, E3PIB0, Q1RDN0, P0A983, P0A982, E3PJ11, D3QPI3, C8UMC8, C8U5F1, C8TPS9, B7UN66, AID77989, ACT71006, ACT29628, ACI39621, A7ZK91, A0A0E2U4L7, A0A0E0Y2L3, A0A070CF47, A0A025C971, A0A023YV55, P0A977, P0A976, C8TRU7, A0A0E2TNW4, D3QMI6, A0A070DEG4 |
| Cluster 44 | 19 | A0A023YTH5, A0A025CAM1, D3QJL6, ACT70390, ACI36951, E3PFR1, C8U2D2, A0A0E2TID4, A0A070D6C5, B7UJR8, A7ZIK3, Q1RF90, AID77540, A0A0E0Y2S7, P46890, ACT30210, A0A0H2V4U9, C8UIM9, C8TJ38 |
| Cluster 45 | 19 | A0A023YTK6, Q8FJW4, Q1REN7, B7UKW1, AID77725, A0A025CFN5, D3QML8, ACT70593, ACI37757, P00962, E3PH74, C8UJY5, C8U331, C8TKJ0, ACT29992, A7ZJ63, A0A0E2TPC6, A0A0E0Y5S4, A0A070D417 |
| Cluster 46 | 18 | A0A023YTU7, A0A025CFL3, D3QMV1, C8U3Z4, ACT70677, ACI37484, A7ZJE0, B7ULN8, P09833, E3PHE7, Q8FJR4, AID77788, C8UK57, C8TKR3, A0A0E2TT73, Q1REG5, A0A070D3V7, A0A0E0Y3N5 |
| Cluster 47 | 18 | A0A023YTV7, A0A025BZ96, C8UK62, C8U400, C8TKR9, A7ZJE6, A0A0E2TW93, A0A0E0Y5K0, P75764, E3PHF6, A0A070D5B7, AID77794, A0A0H2V5D4, B7ULP4, D3QMV7, ACT70683, ACI38202, Q1REF9 |
| Cluster 48 | 17 | A0A023YTY0, A0A025CFQ5, D3QMP6, C8U358, A7ZJ95, A0A070D5L7, A0A0E0Y3K6, C8UK13, C8TKL9, E3PHA3, ACT29962, A0A0E2TWD6, P50465, Q8FJU5, AID77750, Q1REK9, B7ULJ5 |
| Cluster 49 | 19 | A0A023YU17, AID77803, Q1REF1, P0A8F8, E3PI07, D3QNJ5, C8UKZ7, C8U451, C8TLP4, B7ULX6, ACT70734, ACT29864, ACI37185, A7ZJI8, A0A0E2U8U1, A0A0E0Y229, A0A070D3U4, A0A025C8Y7, Q8FJP8 |
| Cluster 50 | 19 | A0A023YU19, A0A025C7Z2, A0A070DBN7, B7UMV1, Q1RE66, Q8FJE7, AID77883, ACT29757, C8UL83, C8U4E0, C8TLX5, A7ZJS7, A0A0E0Y1H6, P75817, E3PI89, D3QNS7, ACT70816, ACI38048, A0A0E2TR84 |
| Cluster 51 | 19 | A0A023YUA3, A0A025CHJ0, P75864, E3PIX2, D3QPE4, ACT70909, ACI37826, ACT29669, A7ZK52, A0A0E0Y183, A0A070DBC0, A0A0E2TS24, C8ULH1, C8TM64, Q8FJ88, B7UN27, AID77958, C8U5B2, Q1RDR6 |
| Cluster 52 | 19 | A0A023YUC9, ACI37550, D3QNL5, ACT29844, A0A025C8W0, Q8FJN1, Q1REC6, B7ULZ5, AID77823, A0A070D596, ACT70755, P27296, C8U472, A0A0E0Y359, C8TLR5, A0A0E2TT41, A7ZJL2, E3PI29, C8UL17 |
| Cluster 53 | 19 | A0A023YUF1, ACT29730, A0A025CI83, C8U4G7, A0A0E2TSW1, ACT70848, ACI39724, P29018, C8ULB0, C8TM02, A0A070D545, A0A0E0Y4Y6, D3QP86, A7ZJV4, AID77909, A0A0H2V7U6, E3PIQ8, B7UMX8, Q1RE27 |
| Cluster 54 | 19 | A0A023YUH0, Q1RDU7, P0AG68, P0AG67, E3PIT2, D3QPA8, C8ULD4, C8U4J1, C8TM26, B7UMZ7, AID77929, ACT70871, ACT29706, ACI36305, A7ZK00, A0A0E2TR24, A0A0E0Y328, A0A070D523, A0A025CN98 |
| Cluster 55 | 19 | A0A023YUH9, D3QPB8, ACT70881, ACI35058, A0A025CNA8, P36566, E3PIU2, C8ULE4, C8U4K0, C8TM36, ACT29696, A7ZK11, A0A0E2TR15, A0A0E0Y318, A0A0H2V5U7, B7UN07, AID77938, A0A070D516, Q1RDT6 |
| Cluster 56 | 38 | A0A023YUI5, Q1RDS7, P0A8M1, P0A8M0, E3PIV1, C8ULF3, C8U4L0, C8TM46, B7UN16, AID77947, ACT29687, A7ZK21, A0A0E2TS39, A0A0E0Y4U8, A0A025CHK7, A0A070D509, D3QPC7, ACT70891, ACI35859, A0A025CUE0, Q8FGQ9, Q1RAR8, B7USP3, AID78900, C8UBY0, C8U390, C8TTA7, A7ZMZ0, A0A0E0XZD9, A0A070D3R8, A0A023YY43, D3QVY3, ACT72251, ACI35807, E3PAG9, A0A0E2TQI4, P21889, ACT28823 |
| Cluster 57 | 19 | A0A023YUI7, A0A025CHH4, Q1RE21, P0A8L2, P0A8L1, E3PIR4, D3QP92, C8ULB6, C8U4H3, C8TM08, B7UMY4, AID77915, ACT70854, ACT29724, ACI35429, A7ZJW2, A0A0E2TSV6, A0A0E0Y1S5, A0A070D4X8 |
| Cluster 58 | 37 | A0A023YUM6, Q1RDK7, P0AFQ6, P0AFQ5, E3PJH9, C8U5H2, B7UNZ3, AID78007, ACT29606, A7ZKB5, A0A070CM53, A0A025C8I9, D3QPK3, C8UMM6, C8TNC0, A0A0E2TMQ7, A0A0E0Y190, ACT71027, A0A023YY40, C8UBQ7, C8TSW9, B7USI4, ACT28884, D3QVS4, P0AEB8, P0AEB7, E3PNJ3, AID78846, ACT72192, ACI38865, A7ZMT1, A0A0E2TPL0, A0A0E0XYE7, A0A070D344, A0A025CPC7, Q1RAY4, C8UA66 |
| Cluster 59 | 12 | A0A023YUQ2, A0A0E0Y2N9, A0A025CK32, P15038, E3PIY6, C8ULI4, C8TM78, ACT29654, A0A0E2TVK2, C8U5C6, A7ZK66, AID77971 |
| Cluster 60 | 10 | A0A023YV62, C8UMS2, C8TNG9, ACI35269, A0A0E2TLB8, A0A070D855, A0A025C2S6, C8TX32, A0A0E0Y254, ACT71120 |
| Cluster 61 | 38 | A0A023YV82, A0A025C9E1, C8UNE8, C8TPF4, A0A0E0Y417, Q1RD73, A0A0E2TQ76, A0A070CFJ4, P21513, A7ZKI9, E3PJP8, B7UPA0, ACT29538, C8U5N9, AID78180, A0A0H2V8D9, D3QQ79, ACT71227, ACI39790, A0A023Z3J1, Q1R693, P0A9J1, P0A9J0, E3PKT9, D3QSW0, C8UGJ7, C8TXN5, C8TIE7, B7UJX9, AID80379, ACT73950, ACT27570, ACI35447, A7ZSE0, A0A0E2TTR6, A0A0E0XX26, A0A070DF93, A0A025CGM6 |
| Cluster 62 | 19 | A0A023YV86, Q1RDU1, P0AAZ8, P0AAZ7, E3PIT8, D3QPB4, C8ULE0, C8U4J6, C8TM32, B7UN03, AID77934, ACT70877, ACT29700, ACI38237, A7ZK06, A0A0E2U8I3, A0A0E0Y4W0, A0A070D519, A0A025CJX7 |
| Cluster 63 | 5 | A0A023YV99, C8UMV2, C8TNJ8, A0A0E0Y0R0, A0A025BTH2 |
| Cluster 64 | 27 | A0A023YVC0, C8TNL4, ACT71162, ACI38840, E3PD74, A0A0E0XS57, C8TPC8, C8TV65, C8TWJ5, A7ZVJ0, A0A070DSR8, E3PBK0, AID81631, A0A023Z6W4, A7ZRB9, E3PJ62, A0A0E0XVV5, A0A0E0Y2C5, AID78108, A0A0H2V678, C8TX19, C8TTS5, A0A0H2VAM5, A0A0E2TJT0, A0A023Z367, AID80052, C8U0P7 |
| Cluster 65 | 17 | A0A023YVH0, Q1RD68, P0A7N4, E3PJQ3, D3QQ84, C8UNF3, C8U5P4, C8TPF9, B7UPA5, AID78184, ACT71232, ACT29534, ACI36826, A0A0E2TMJ5, A0A0E0Y410, A0A070CMG1, A0A025CCU1 |
| Cluster 66 | 19 | A0A023YVJ1, P0ACE7, E3PJR7, C8UNG7, C8U5Q8, C8TPH3, B7UPB9, ACT71247, ACT29520, ACI37380, A7ZKK7, A0A0E2U3K8, A0A0E0Y219, A0A070CEH9, A0A025C6Z7, D3QQ98, Q1RD51, AID78197, A0A0H2V8F0 |
| Cluster 67 | 19 | A0A023YVL7, A0A025C6S1, Q1RDB4, P0A8D7, P0A8D6, E3PJK8, D3QQ40, B7UP60, AID78144, ACT71190, ACT29576, ACI35083, A7ZKE8, A0A0E2U3S4, A0A070CG74, C8UMZ0, C8U5K1, C8TNP0, A0A0E0Y224 |
| Cluster 68 | 19 | A0A023YVL8, A0A025C5C7, P25745, E3PKE3, C8UNR3, C8U6R9, C8TQG6, ACT29489, A7ZKS3, A0A0E0Y1Y8, A0A070DZ36, D3QQR6, ACT71354, ACI36212, B7UQ42, Q1RD20, AID78244, A0A0E2TRA1, Q8CXZ8 |
| Cluster 69 | 19 | A0A023YVS0, P0A6X1, E3PKJ7, D3QQY3, ACT71441, ACI39303, A0A025CD96, ACT29437, C8U6X2, C8TQM2, A7ZKY4, A0A0E2U774, A0A0E0Y3M5, A0A070DZC8, C8UNW6, Q8FI03, Q1RCM8, AID78363, B7UQ98 |
| Cluster 70 | 19 | A0A023YVU0, A0A025C771, D3QR03, ACT71463, ACI35095, Q1RCK6, P0A440, E3PKL9, C8UNY7, C8U6Z4, B7UQC4, AID78388, ACT29416, A0A0E2TTZ9, A0A0E0Y0E9, A0A070DZF0, P37051, A7ZL05, C8TQP3 |
| Cluster 71 | 52 | A0A023YW19, D3QQY9, A7ZKZ0, A7ZKZ1, ACT29431, A0A0H2V755, C8U6Y0, A0A0E0Y029, Q6BF87, B7UQA4, ACT71448, ACT29430, E3PKK4, Q6BF86, C8UNX4, C8UNX2, C8U6X8, C8TQM8, ACT71447, A0A0E0Y0G1, C8U6X9, A0A0E0Y1K9, C8UNX3, C8TQM9, E3PKK3, AID80679, A0A070DG62, AID80678, A0A0H2VBW3, ACI36609, A0A0E2U0I1, A0A070D812, D3QUW5, Q1R560, A7ZT81, A0A0E2TIL8, A0A025C371, A0A023Z570, E3PMC8, Q1R564, Q1R562, C8TYG1, C8UI19, C8TYG0, C8TJX6, B7UL93, ACT74253, ACT27274, A0A0E0XW56, B7UL94, Q6BF25, ACT27276 |
| Cluster 72 | 7 | A0A023YW23, A0A025BL00, D3QQW3, Q1RCP7, AID78347, B7UQ82, A0A0H2V6X6 |
| Cluster 73 | 19 | A0A023YW37, A0A025C9V0, Q1RCN4, P0A7D2, P0A7D1, E3PKJ1, D3QQX7, C8UNW0, C8U6W6, C8TQL6, B7UQ92, AID78357, ACT71435, ACT29443, ACI37406, A7ZKX7, A0A0E2TRF4, A0A0E0Y1U8, A0A070DQK2 |
| Cluster 74 | 19 | A0A023YWB0, P0ABU2, E3PKJ0, D3QQX6, C8UNV9, C8U6W5, C8TQL5, ACT71434, ACI37671, A7ZKX6, A0A0E2TTS2, A0A0E0Y044, A0A070DRZ4, A0A025C796, Q1RCN5, B7UQ91, AID78356, A0A0H2V6V8, ACT29444 |
| Cluster 75 | 19 | A0A023YWB5, Q1RCA1, D3QRN6, ACI39787, P0AFR5, P0AFR4, E3PL25, C8UP67, C8U785, B7UR73, AID78419, ACT71616, ACT29382, A7ZL85, A0A0E2TPG1, A0A0E0XZR9, A0A025CV93, A0A070DPG6, C8TQW6 |
| Cluster 76 | 19 | A0A023YWC5, A0A025CV01, C8UPE3, C8TR44, ACT29300, D3QSA4, Q8FHP6, AID78493, A0A070DPA2, B7URE9, Q1RC21, A0A0E2U5K9, P76055, C8U7G2, ACT71700, ACI37471, A7ZLH4, A0A0E0XZY3, E3PLA5 |
| Cluster 77 | 18 | A0A023YWE7, A0A025CCV8, P30958, E3PJS7, D3QQA9, ACT71258, ACI38224, A0A070CMI5, C8TPI4, A0A0E2TMG8, A0A0E0Y3V7, Q1RD40, B7UPD0, ACT29509, C8UNH7, A7ZKL8, C8U5R9, A0A0H2V7A9 |
| Cluster 78 | 75 | A0A023YWL5, A0A025CWV2, P37765, E3PL27, ACT29380, Q8FHV4, Q1RC99, B7UR75, AID78421, D3QRN8, C8UP68, C8U786, C8TQW7, ACT71618, ACI35555, A7ZL86, A0A0E2U5H3, A0A0E0Y1I0, A0A070DQY6, Q8FB47, Q1R3R6, B7UPI3, AID81202, A0A025C485, A0A023Z5V5, C8TZU8, D3QK29, ACT74783, ACI39223, A7ZUN6, A0A0E0Y915, A0A0E2THP6, A0A070DAW3, P0AA44, P0AA43, E3PCX5, D3QJX5, C8UDB3, C8U6C5, C8TUA8, B7UFK0, AID79322, ACT72845, ACT28536, ACI39388, A7ZP07, A0A0E2TS15, A0A0E0XY82, A0A070CPZ2, A0A025CH14, A0A023Z039, Q1R9N6, A0A023YWM3, A0A025C734, P75966, E3PKE5, C8UNR5, C8U6S1, C8TQG8, A7ZKS5, A0A0E2TPU5, A0A070DRB9, D3QQR8, ACT71356, A0A0E0Y0L9, ACI35118, Q8FIB6, Q1RD18, ACT29487, B7UQ44, C8UKS6, C8TMR2, P32684, E3PBY7, ACT31000 |
| Cluster 79 | 15 | A0A023YWN2, A0A025BRV8, C8UPV9, D3QT68, C8U8J0, ACT71826, ACI38680, A0A0E0Y2S8, P76145, E3PM19, ACT29162, A0A0E2TJU4, A0A070D2C0, A7ZLX5, Q1RBP8 |
| Cluster 80 | 19 | A0A023YWN6, A0A025CU73, P30850, C8U7A5, A7ZLA9, A0A0E0Y041, ACT71638, ACI34834, C8UP86, A0A070DQC0, E3PL46, D3QRQ8, C8TQY6, A0A0E2TNT8, Q8FHT6, AID78439, ACT29358, B7UR96, Q1RC77 |
| Cluster 81 | 19 | A0A023YWS6, A0A025C816, A0A0E0Y1L3, P0ACC1, C8UNW8, C8TQM4, A0A0E2TQV6, A0A070DRI9, C8U6X4, A7ZKY6, Q1RCM6, AID78365, A0A0H2V965, B7UQA0, D3QQY5, ACT71443, ACI34958, E3PKJ9, ACT29435 |
| Cluster 82 | 19 | A0A023YWU9, Q1RC23, P64424, P64423, E3PLA3, D3QSA2, C8UPE1, C8TR42, B7URE7, AID78491, ACT71696, ACT29303, ACI38524, A7ZLH2, A0A0E2TS63, A0A0E0XZJ5, A0A070DQ62, A0A025CZW1, C8U7G0 |
| Cluster 83 | 19 | A0A023YWX6, A0A025BRD7, E3PLZ6, P31826, D3QT43, ACT71803, ACI38106, C8UPT7, C8TRL6, C8U8G6, ACT29179, A7ZLU5, A0A0E0Y0Z0, Q1RBS6, AID78577, A0A0H2V7C3, A0A070D1Y2, B7URM9, A0A0E2TGW0 |
| Cluster 84 | 19 | A0A023YX25, A0A0E0Y1G9, A0A025CU82, A7ZL96, A0A070DQD2, E3PL36, C8UP76, C8U794, C8TQX5, ACT29370, P25516, D3QRP7, ACT71627, ACI35595, A0A0E2TNT0, B7UR84, AID78429, A0A0H2V9B9, Q1RC89 |
| Cluster 85 | 18 | A0A023YXB1, A0A025CQ07, Q1RBG8, D3QTY5, C8U8V0, ACT72006, ACI36422, A7ZM88, A0A0E2TP36, A0A0E0Y081, P77223, E3PMK4, C8UQC1, C8TSA4, ACT29060, B7URW9, Q8FH96, AID78671 |
| Cluster 86 | 6 | A0A023YXB4, D3QSB2, C8TT58, P38394, C8TR51, ACT29294 |
| Cluster 87 | 19 | A0A023YXC1, D3QTZ4, P0AGJ9, E3PML4, C8UB58, C8U9K6, C8TSB3, ACT72015, ACI37789, A7ZM99, A0A0E2TNM4, A0A025CQ19, Q1RBF8, Q8FH88, B7URX8, AID78681, A0A0E0Y068, A0A070D992, ACT29050 |
| Cluster 88 | 19 | A0A023YXL1, Q1RB81, P67036, D3QUQ8, B7US97, AID78758, ACT72092, ACI36473, A0A0E0Y027, A0A025CR89, A0A0E2U5Y5, P08312, E3PN99, C8U9X9, C8TSN1, ACT28977, A7ZMI2, A0A070D9E9, C8UBH0 |
| Cluster 89 | 19 | A0A023YXL2, Q1RBE3, P66681, D3QU09, C8UB73, C8U9M1, C8TSC8, AID78696, ACT72030, ACT29035, ACI35217, A7ZMB5, A0A0E2U5T1, A0A0E0Y054, A0A025CQ38, B7URZ3, P30014, E3PMM9, A0A070D0U2 |
| Cluster 90 | 19 | A0A023YXL6, A0A025CQ85, Q1RB76, P0A8M4, P0A8M3, E3PNA3, B7USA2, AID78762, ACT28972, A0A070D9F1, D3QUR3, ACT72097, ACI39262, A7ZMI6, A0A0E0XYC0, C8UBH5, C8U9Y4, C8TSN6, A0A0E2TQ45 |
| Cluster 91 | 19 | A0A023YXM3, Q1RBD4, P0A9H8, P0A9H7, E3PMN8, C8UB83, C8TSD8, B7US02, AID78704, ACT29026, A0A070D0U9, A0A025CR36, C8U9N1, A7ZMC7, A0A0E2TPX9, A0A0E0Y043, ACT72041, ACI34828, D3QU18 |
| Cluster 92 | 19 | A0A023YXW5, A0A025CNC7, E3PNJ2, D3QVS3, C8UBQ6, C8TSW8, AID78845, ACT72191, ACT28885, ACI35607, A7ZMS9, A0A0E0Y1N2, A0A070D9Q7, C8UA65, A0A0H2V842, Q1RAY6, A0A0E2TP14, P76257, B7USI3 |
| Cluster 93 | 18 | A0A023YXW9, A0A025CQ78, ACT72091, ACI38744, C8TSN0, A7ZMI1, A0A0E2TQ39, A0A0E0XYC5, P07395, ACT28978, A0A070D0Z5, D3QUQ7, C8UBG9, C8U9X8, E3PN98, P59664, B7US96, Q1RB82 |
| Cluster 94 | 19 | A0A023YXX6, Q1RB77, E3PNA2, D3QUR2, AID78761, ACI38539, A7ZMI5, A0A0E2TSK0, A0A0E0XZT8, A0A070D100, A0A025CST1, P0A708, P0A707, C8UBH4, C8U9Y3, C8TSN5, B7USA1, ACT72096, ACT28973 |
| Cluster 95 | 19 | A0A023YY12, A0A025CND1, P64483, D3QVQ2, B7USG7, ACT72170, ACI35140, Q1RB05, E3PNH1, C8UA41, ACT28905, A0A070D183, C8UBN3, C8TSU5, A0A0E0XZM5, AID78829, A0A0H2V975, A0A0E2TNZ7, A7ZMQ7 |
| Cluster 96 | 19 | A0A023YY35, A0A025CPC1, P09155, E3PNI8, A0A0E2TPK6, D3QVR9, C8UA61, ACT72187, ACI35375, A7ZMS5, A0A0E0XYF1, A0A070D339, ACT28889, C8UBQ2, Q1RAZ0, B7USH9, A0A0H2V985, AID78841, C8TSW4 |
| Cluster 97 | 17 | A0A023YY36, Q1RB78, P0A7Q1, E3PNA1, D3QUR1, C8UBH3, C8U9Y2, C8TSN4, AID78760, ACT72095, ACT28974, ACI36620, A0A0E2TPD7, A0A0E0XYP3, A0A070D3B1, A0A025CW47, B7USA0 |
| Cluster 98 | 5 | A0A023YY48, C8U734, A0A025BJX1, A7ZKQ0, A7ZL58 |
| Cluster 99 | 19 | A0A023YY57, Q8FGQ0, AID78966, A0A025CUF3, A0A0E0XZ81, P11875, E3PAH9, D3QVZ3, ACT28813, A0A070CRR9, C8UC53, C8U3G7, C8TTB7, B7USQ3, ACT72261, ACI34752, A0A0E2TQI9, Q1RAQ8, A7ZN02 |
| Cluster 100 | 19 | A0A023YY77, A0A025CNH3, A7ZMV8, A0A0E0XY03, E3PNM1, C8UA94, ACT28856, ACT72220, ACI38542, B7USL2, A0A070D369, P76273, Q8FGS9, AID78871, D3QVV2, A0A0E2TSW4, C8TSZ6, Q1RAV2, C8UBT5 |
| Cluster 101 | 19 | A0A023YY87, A0A025CPG6, Q1RAU2, P0AEK1, P0AEK0, D3QVW1, C8UBU4, C8UAA2, C8TT71, AID78880, A7ZMW7, A0A0E0XXZ3, A0A070D9V9, ACT28847, B7USM1, E3PNN0, A0A0E2TPP1, ACT72229, ACI37017 |
| Cluster 102 | 15 | A0A023YYB6, A0A025CVY0, ACT72031, ACI37653, D3QU10, ACT29034, P30015, E3PMN0, C8U9M2, A0A0E2TNN3, A0A0E0XYN4, C8UB74, A0A070D9A5, A7ZMB7, C8TSC9 |
| Cluster 103 | 19 | A0A023YYH0, P07364, A0A025CPL0, C8UC61, C8TTC5, A0A0E2TNS7, A0A0E0Y191, D3QW01, ACT72269, ACI39083, ACT28805, AID78973, A0A0H2V812, E3PAI7, A0A070CXP0, C8U3H5, Q1RAQ0, A7ZN11, B7USR0 |
| Cluster 104 | 19 | A0A023YYI0, Q1RB79, P0A7L4, P0A7L3, E3PNA0, D3QUR0, C8UBH2, C8U9Y1, C8TSN3, B7US99, AID78759, ACT72094, ACT28975, ACI35828, A7ZMI4, A0A0E2TNS8, A0A0E0Y1W1, A0A070D1J5, A0A025CQA7 |
| Cluster 105 | 55 | A0A023YYS7, P52612, E3PBC7, C8U3M1, C8TTL7, ACT72352, ACT28756, ACI38838, A0A0E2TML4, A0A0E0XXU4, C8UCB3, Q1RAK0, D3QWW2, A7ZN64, A0A070CXK9, AID79028, A0A0H2V887, B7USV5, C8THS2, C8UHC0, C8UFL5, C8TNT0, D3QW29, B7UMA6, ACT74404, ACI35286, A0A025BZR7, A0A023Z4Q4, A0A070CLH7, C8TWM9, A0A0E2TK34, ACT73572, ACI35009, A0A025CVR8, A0A023Z245, A0A0E2U259, Q1R4K2, P0ABB5, P0ABB4, E3PNT1, D3QWC9, C8UJC3, C8TYW5, C8TL76, B7UMJ7, AID80894, ACT74499, ACT31279, ACI39447, A7ZTU4, A0A0E0Y9X9, A0A070D888, A0A025BZM8, A0A023Z5D1, A0A070CIL8 |
| Cluster 106 | 37 | A0A023YYX4, D3QVU8, ACT72216, ACI37948, A0A070D1W2, A0A025CUA3, E3PNL7, C8UBT1, C8UA90, C8TSZ2, ACT28860, A0A0E2TQG0, A0A0E0XZQ1, Q8FGT3, Q1RAV6, B7USK8, AID78868, P45577, A7ZMV4, A0A023Z4J8, A0A025CJK2, C8TZ60, D3QYM0, C8TWG6, Q1R1P6, C8URI1, A0A070DLY0, A0A0E2TKG4, E3PP26, C8UB23, A0A0E0Y8Q9, ACT75236, ACI34452, A7ZGM9, E3PN53, A0A0E0Y6S9, B7UTI5 |
| Cluster 107 | 22 | A0A023YZ33, P60664, E3PBQ2, C8U570, B7UT62, ACT72568, ACI36718, A0A025CBM9, Q8FG48, Q1RA48, AID79176, A0A0E0XXJ0, D3QY05, A0A070CPD6, A0A0E2U9S9, C8TTV9, A7ZNJ7, ACT28692, C8UCX5, A0A070CPC7, A0A025CBP3, A0A023YZG3 |
| Cluster 108 | 19 | A0A023YZD4, A0A025CAU8, ACT72554, ACI37346, C8UCW0, D3QXZ0, C8TTU4, A0A0H2VAY0, B7UT49, AID79164, A0A0E0XYP6, ACT28704, C8U555, P04995, Q1RA61, A0A0E2TU92, E3PBN7, A7ZNI3, A0A070CQQ7 |
| Cluster 109 | 19 | A0A023YZE6, Q1RA54, P60758, P60757, E3PBP6, D3QXZ9, C8UCW9, C8U564, C8TTV3, B7UT56, AID79170, A7ZNJ1, A0A0E2TSK6, A0A0E0Y0P4, A0A070CRF6, A0A025CAR8, ACT72562, ACI35755, ACT28698 |
| Cluster 110 | 18 | A0A023YZF1, B7UT61, A0A025CAS5, ACT28693, ACT72567, ACI39745, C8U569, A7ZNJ6, A0A070CRF1, E3PBQ1, Q8FG49, Q1RA49, AID79175, C8TTV8, A0A0E2TSK1, D3QY04, A0A0E0Y0P0, C8UCX4 |
| Cluster 111 | 19 | A0A023YZL3, A0A025CFH6, P25889, D3QJT9, C8U689, ACT72807, ACT28572, ACI35170, A0A0E2TWY6, C8UD78, C8TU71, A0A0E0Y0E1, E3PCT7, Q1R9S6, A0A0H2V8T8, AID79287, A7ZNW9, B7UFG3, A0A070CQS9 |
| Cluster 112 | 38 | A0A023YZP0, A0A025CB42, P00959, D3QJQ0, ACT72702, ACI36354, E3PCQ8, C8UD48, C8U657, C8TU40, A7ZNT3, A0A0E0XYE5, A0A070CQW3, B7UFD1, A0A0E2TX09, Q8FFX8, Q1R9V8, AID79258, ACT28604, A0A023Z336, C8UG32, C8TX68, C8TH92, A7ZRV7, A0A0E2UAD4, A0A0E0XXI9, A0A070D8C2, A0A025CG52, Q1R6Q5, D3QS19, B7UIY1, AID80218, ACT73786, ACI37992, A0A0H2VDQ7, P42589, E3PJY8, ACT27737 |
| Cluster 113 | 19 | A0A023YZS4, D3QJX6, B7UFK1, ACT72846, ACI37143, A0A025CKG5, C8UDB4, C8U6C6, C8TUA9, P33919, E3PCX6, ACT28535, A0A0E2U9B9, Q1R9N5, AID79323, A0A0H2V8W2, A0A070CWP1, A7ZP08, A0A0E0XWQ4 |
| Cluster 114 | 19 | A0A023YZX8, D3QKW6, ACI37117, A0A025CKA1, P0A8V0, E3PDX1, C8UDM3, C8U6J7, C8TUR6, ACT28453, A0A070CNG1, A0A0E2TTJ1, ACT72925, A7ZP87, A0A0E0XWH8, B7UFT1, Q8FFK8, Q1R9E5, AID79404 |
| Cluster 115 | 19 | A0A023YZY8, P68919, E3PCX7, D3QJX7, C8UDB5, C8U6C7, C8TUB0, ACT28534, A7ZP09, A0A0E2TTR1, A0A0E0XYF3, A0A025CEP4, ACT72847, ACI37585, A0A070CNF7, Q1R9N4, Q8FFS1, B7UFK2, AID79324 |
| Cluster 116 | 7 | A0A023YZY9, C8TKS5, C8UCV6, Q1R906, Q1RD11, A0A0H2V6N3, C8UR96 |
| Cluster 117 | 19 | A0A023Z003, A0A025CGY9, P33941, E3PDR3, ACT28510, A7ZP33, AID79348, A0A0H2V8X9, A0A070CND7, C8UDF7, C8U6F0, A0A0E2TRZ1, D3QK00, ACT72871, ACI38095, C8TUF0, A0A0E0XYC7, B7UFM5, Q1R9K6 |
| Cluster 118 | 38 | A0A023Z031, Q1R9T3, P33371, E3PCT0, ACT28579, A0A025CKK2, A0A0E2U9I6, Q8FFV5, B7UFF6, AID79280, C8U682, C8UD71, C8TU64, A0A0E0XWU4, D3QJS6, ACT72794, ACI39751, A7ZNV7, A0A070CWT9, A0A023Z3R3, Q1R668, P0ABT6, P0ABT5, E3PKV1, D3QSX3, C8UGL8, C8TXP7, C8TIG8, B7UJZ1, AID80399, ACT73964, ACT27556, ACI35079, A7ZSF5, A0A0E2TUD1, A0A0E0XV75, A0A070D6Y3, A0A025CMI2 |
| Cluster 119 | 38 | A0A023Z033, A0A025CEI8, D3QKV2, ACT72911, ACI37839, B7UFR7, A0A070CPT1, Q8FFM1, AID79390, C8U6I3, C8TUQ2, A7ZP73, A0A0E0XY93, Q1R9G0, P77398, E3PDV7, ACT28467, A0A0E2TWM0, A0A023Z4A3, A0A025C6H8, A0A070D8T2, C8TXR7, B7UK11, A0A0E2TLS5, A0A0E0XV56, D3QTC7, ACT73985, ACI35997, A7ZSH6, P23882, E3PKX1, ACT27535, C8UGN7, C8TII9, Q1R645, Q8FD13, AID80419, A7ZGN8 |
| Cluster 120 | 18 | A0A023Z053, Q1R9L4, P0AAL4, P0AAL3, E3PDQ7, D3QJZ4, C8UDF0, C8U6E3, C8TUE3, B7UFL8, AID79342, ACT28517, A7ZP27, A0A0E2TT24, A0A070CWM4, A0A025CEJ8, ACT72865, ACI36182 |
| Cluster 121 | 19 | A0A023Z084, P65844, D3QL11, AID79448, ACT72972, ACI36109, A0A0E0XY39, A0A025CED3, Q1R994, B7UFX6, A7ZPD3, E3PEU3, ACT28404, C8U6P1, A0A0E2TWG5, A0A070CN24, P07649, C8UDR9, C8TUW3 |
| Cluster 122 | 8 | A0A023Z0B8, C8U7P3, A0A025C9N5, ACT73069, ACI39765, A0A0E0Y2S9, E3PG48, B7UGU3 |
| Cluster 123 | 19 | A0A023Z0G0, A0A025CE85, P39199, E3PEV4, A0A0E2TRP0, A7ZPE6, C8U6Q4, A0A070CN91, AID79460, C8UDT1, C8TUX5, ACT28392, A0A0E0XWR3, ACT72984, ACI39271, B7UFY8, Q1R982, A0A0H2V945, D3QL23 |
| Cluster 124 | 19 | A0A023Z0N1, A0A025C550, D3QM53, ACT73180, ACI36399, A0A0E2TV90, C8UE41, C8U7Z5, C8TVG8, A7ZPR8, A0A0E0XXK9, A0A070CMY9, B7UGM4, Q1R8Q3, P76562, E3PFW4, ACT28280, AID79570, A0A0H2V9K2 |
| Cluster 125 | 5 | A0A023Z0S6, C8U7L2, A0A025CBA9, A0A0E0Y2P8, A0A0E2TH56 |
| Cluster 126 | 18 | A0A023Z0S7, A0A025CFR7, Q8FF64, AID79593, Q1R8M7, A0A0E2TV63, A0A0E0XZA5, P04994, E3PG53, ACT28243, A0A070CFX3, C8U828, C8TVK4, C8UE77, D3QMZ6, ACT73216, ACI39417, B7UGU7 |
| Cluster 127 | 19 | A0A023Z0Z3, A0A025CD95, Q1R8M2, Q8FF59, AID79598, P0A6P5, E3PG55, D3QMZ8, C8UE79, C8U830, C8TVK6, ACT73218, ACT28241, ACI38511, A7ZPV4, A0A0E2TUM7, A0A0E0XX99, A0A070CET9, B7UGV7 |
| Cluster 128 | 19 | A0A023Z129, D3QM79, A0A070CFY3, A0A025CDC9, E3PFZ0, C8UE68, C8U819, C8TVJ5, ACT28254, A7ZPU3, A0A0E2TYC2, A0A0E0XZB4, P08179, Q1R8N8, AID79584, A0A0H2V9D8, ACT73206, ACI34621, B7UGN8 |
| Cluster 129 | 19 | A0A023Z149, P60786, P60785, E3PGV9, D3QNB1, C8UED5, C8U939, C8TVR4, AID79650, ACT73281, ACT28178, ACI37488, A7ZQ13, A0A0E2TV18, A0A0E0XVZ3, A0A070CI61, A0A025CD70, Q1R8G3, B7UH09 |
| Cluster 130 | 19 | A0A023Z150, Q1R8L9, P60907, P60906, E3PG58, D3QN01, C8UE82, C8U833, C8TVK9, AID79601, ACT73221, ACT28238, ACI36920, A7ZPV7, A0A025CDB5, B7UGW0, A0A0E0XZA1, A0A0E2TV58, A0A070CFW7 |
| Cluster 131 | 19 | A0A023Z157, D3QNW6, ACI35323, Q1R8B7, P0A7X7, P0A7X6, E3PGZ2, C8UEG8, C8U974, C8TVU8, B7UH57, AID79683, ACT73315, ACT28144, A7ZQ48, A0A0E2TI33, A0A0E0XVW1, A0A070CG91, A0A025CV16 |
| Cluster 132 | 19 | A0A023Z167, P52119, E3PH01, D3QNX5, C8UEH7, C8U984, C8TVV7, ACT28135, A7ZQ58, A0A0E2TZE4, A0A0E0XZ10, A0A070CG83, A0A025CYD3, Q8FEY5, B7UH66, AID79692, ACT73324, ACI35742, Q1R8A6 |
| Cluster 133 | 38 | A0A023Z172, Q1R8K0, P0AE02, P0AE01, E3PG76, C8U851, B7UGX8, AID79619, ACT28218, A0A0E0XVN3, A0A070CGM4, A0A025CFN7, D3QN23, ACT73243, ACI36429, A7ZPX6, A0A0E2TTR4, C8UEA0, C8TVM7, A0A023Z7R6, D3QNG3, A0A025CKA7, ACT30613, ACT75160, ACI36203, C8UNB0, C8TQF6, A7ZVU5, A0A0E2TV86, A0A070D5S0, B7URL5, C8U175, A0A0E0Y4D8, P37005, A0A0H2VEC9, AID81571, Q1R238, E3PFI5 |
| Cluster 134 | 19 | A0A023Z192, A0A025CE19, Q1R8M9, P64294, D3QM87, C8UE75, C8U826, C8TVK2, AID79591, ACT73214, ACI36449, A0A0E2TTT4, A0A0E0XVR2, A0A070CGP5, P04079, E3PG51, B7UGU5, ACT28245, A7ZPV1 |
| Cluster 135 | 19 | A0A023Z1A4, Q1R8G6, P0A7Y1, P0A7Y0, E3PGV7, D3QNA8, C8UED3, C8U937, C8TVR2, B7UH07, AID79648, ACT73278, ACT28180, ACI39620, A7ZQ11, A0A0E2TTJ5, A0A0E0XXB6, A0A070CPE6, A0A025CFK6 |
| Cluster 136 | 19 | A0A023Z1B0, P0A7T4, P0A7T3, E3PGZ3, D3QNW7, C8UEG9, C8U975, C8TVU9, B7UH58, AID79684, ACT73316, ACT28143, ACI34590, A7ZQ49, A0A0E2TLD8, A0A0E0XX02, A0A070CI35, A0A025CSH4, Q1R8B6 |
| Cluster 137 | 17 | A0A023Z1D0, A0A025CYU7, D3QPP4, ACT73399, ACI37624, A0A070CJS8, B7UHB5, C8UEP9, C8U9C4, C8TFT9, A7ZQC5, A0A0E2TZJ1, A0A0E0XVQ5, P00957, E3PH61, Q1R804, ACT28094 |
| Cluster 138 | 19 | A0A023Z1G3, P06616, E3PGV6, ACT28181, A0A025CD45, Q8FF17, Q1R8G7, C8UED2, C8U936, C8TVR1, B7UH06, AID79647, A7ZQ10, A0A0E2TUE7, A0A0E0XVJ4, A0A070CHH2, D3QNA7, ACT73277, ACI38879 |
| Cluster 139 | 19 | A0A023Z1H0, Q1R8B8, P0A874, P0A873, E3PGZ1, D3QNW5, C8U973, C8TVU7, AID79682, ACT73314, ACT28145, ACI39695, A7ZQ47, A0A0E2TZF9, A0A0E0XZ16, A0A070CPB1, A0A025CYC3, B7UH56, C8UEG7 |
| Cluster 140 | 19 | A0A023Z1H2, A0A025CW39, A0A070CHN1, D3QPU2, ACT73446, ACI37753, C8U9H7, A0A0E0XWW9, Q57261, ACT28045, A7ZQI9, A0A0E2TZM8, Q8FEJ7, AID79788, Q1R7U6, E3PHQ2, C8TFZ3, C8UEV3, B7UHG4 |
| Cluster 141 | 19 | A0A023Z1I4, P0AGJ5, E3PGX1, D3QNC3, C8UEE6, C8U952, C8TVS6, ACT73293, ACT28165, ACI36123, A0A0E2TUD4, A0A070CHF8, A0A025CD30, A7ZQ26, A0A0E0XZ35, B7UH20, Q1R8F1, AID79662, A0A0H2VB45 |
| Cluster 142 | 12 | A0A023Z1I6, Q46899, E3PHR5, A0A0E0XWN6, C8UEW6, C8TG06, A0A0E2TLP0, A7ZQK1, D3QPV5, ACT73459, ACI38737, A0A070CHP0 |
| Cluster 143 | 18 | A0A023Z1J4, A0A025CYT7, D3QPQ9, ACI36559, Q1R7Y8, A0A0H2VBG4, ACT28079, A0A0E0XWT4, C8U9D9, A7ZQE1, A0A070CHR8, ACT73413, A0A0E2TZK2, C8UER4, C8TFV4, E3PHL9, P30131, AID79752 |
| Cluster 144 | 28 | A0A023Z1L3, Q1R7Q9, P0AE72, D3QPY2, C8UEZ1, C8UAB5, C8TG30, B7UHJ9, ACT73486, ACT28012, ACI39652, A7ZQM6, A0A0E2TRE4, A0A0E0XVG6, A0A070CHM3, A0A025CX01, E3PHU0, D3QLE3, ACI39082, ACT75005, P08365, C8UM76, C8U0J0, C8TP45, A7ZVA2, A0A0E0Y6C1, A0A025CM69, A0A023Z6Z6 |
| Cluster 145 | 19 | A0A023Z1N6, Q1R8B9, P0A7K7, P0A7K6, E3PGZ0, D3QNW4, C8UEG6, C8U972, C8TVU6, B7UH55, AID79681, ACT73313, ACT28146, ACI37770, A7ZQ46, A0A0E2THM6, A0A0E0XX80, A0A070CHD6, A0A025CSI1 |
| Cluster 146 | 4 | A0A023Z1P2, A0A025CW57, E3PHR6, P76632 |
| Cluster 147 | 19 | A0A023Z1T2, A0A025CYR9, P23909, ACT28057, D3QPT2, ACT73436, ACI38015, C8UET7, C8U9G1, C8TFX7, A0A0E2TI91, A0A0E0XV68, A0A070CJP2, E3PHP0, Q8FEL3, AID79774, Q1R7W4, B7UHE9, A7ZQH3 |
| Cluster 148 | 19 | A0A023Z1U9, P54901, D3QN26, C8UEA3, C8TVN0, ACT73246, ACI38945, A0A025CDZ1, E3PG79, ACT28215, B7UGY1, A0A070CMS1, AID79622, C8U854, A7ZPX9, A0A0E2TY84, A0A0E0XW29, Q1R8J7, A0A0H2V9I2 |
| Cluster 149 | 4 | A0A023Z1V6, Q46897, A0A025CYQ4, E3PHR3 |
| Cluster 150 | 11 | A0A023Z1W0, A0A025CW29, P38036, A0A0E2TUU8, A0A070CIV9, A7ZQK4, C8UEW9, C8U9J2, A0A0E0XYP7, ACT73462, ACI37723 |
| Cluster 151 | 19 | A0A023Z1W9, Q1R805, P69914, P69913, E3PH60, D3QPP3, C8UEP8, C8U9C3, C8TFT8, B7UHB4, AID79731, ACT73398, ACT28095, ACI38783, A7ZQC4, A0A0E2TI66, A0A0E0XYX1, A0A070CHV1, A0A025CW77 |
| Cluster 152 | 19 | A0A023Z1X5, A0A025CBZ0, P21893, D3QQL5, ACT73603, ACI38898, E3PIG8, Q1R7D9, A0A070CIK1, ACT27913, A0A0E2U7K7, C8UAP6, B7UHU1, AID79929, A0A0H2VCR3, C8UF98, C8TGQ4, A7ZR00, A0A0E0XV63 |
| Cluster 153 | 29 | A0A023Z1Y3, A0A025CW13, P0AE70, E3PHT9, D3QPY1, ACT73485, ACT28013, ACI39484, A0A0E2TUT1, A0A070CQK9, Q1R7R1, B7UHJ8, C8UEZ0, C8UAB4, C8TG29, A7ZQM5, A0A0E0XYM3, D3QLE4, C8UM77, C8U0J1, C8TP46, ACT75006, ACI38648, A7ZVA3, A0A0E0Y8A8, A0A025CLB0, A0A023Z7V3, P33647, A0A025CV31 |
| Cluster 154 | 19 | A0A023Z1Z5, P31825, E3PGW5, ACT28171, A0A025CDW3, A7ZQ20, A0A0E0XVI3, C8UEE1, C8U945, C8TVS0, A0A070CG70, Q8FF14, Q1R8F7, B7UH15, AID79656, A0A0E2TY29, ACT73287, ACI38891, D3QNB7 |
| Cluster 155 | 19 | A0A023Z214, P0A7A5, E3PHQ0, D3QPU0, C8UEV1, C8U9H5, C8TFZ1, B7UHG2, ACT73444, ACT28047, ACI38111, A7ZQI7, A0A0E2TLM2, A0A0E0XWQ2, A0A070CHP9, A0A025CW69, Q8FEJ8, Q1R7U8, AID79786 |
| Cluster 156 | 19 | A0A023Z256, Q1R8B5, P0AGD8, P0AGD7, E3PGZ4, D3QNW8, C8U976, B7UH59, AID79685, ACT73317, ACT28142, ACI36835, A7ZQ50, A0A0E2THP4, A0A0E0XVF4, A0A070CG41, A0A025CTF9, C8UEH0, C8TVV0 |
| Cluster 157 | 19 | A0A023Z262, Q1R8A4, P0A833, P0A832, E3PH03, D3QNX7, C8UEH9, C8U986, C8TVV9, B7UH68, AID79694, ACT73326, ACT28133, ACI38650, A7ZQ60, A0A0E2TLA3, A0A0E0XWZ3, A0A025CSI4, A0A070CG34 |
| Cluster 158 | 19 | A0A023Z273, P0ADR6, E3PHW3, C8UAD8, C8TG53, ACT73508, ACT27983, ACI37173, A7ZQQ1, A0A0E0XV01, A0A025D1S3, Q8FEE5, B7UHM2, AID79842, C8UF13, A0A0E2TS00, Q1R7N4, A0A070CHH4, D3QQD9 |
| Cluster 159 | 19 | A0A023Z284, A0A025D1Q8, P04993, E3PHX3, A7ZQS5, A0A0E0XUZ4, ACT27973, A0A070CHG4, C8TG68, AID79876, A0A0H2VCM0, C8UAG7, A0A0E2TRZ0, Q1R7J5, C8UF27, D3QQE9, ACT73518, ACI36562, B7UHN6 |
| Cluster 160 | 19 | A0A023Z291, P67603, E3PIH6, D3QQM3, ACT73611, ACI37656, A0A025CBX1, C8UAQ5, A0A0E2TR53, C8UFA6, C8TGR2, ACT27905, A7ZR09, A0A0E0XWH3, A0A070CJB0, Q8FE69, Q1R7D0, AID79937, B7UHU9 |
| Cluster 161 | 4 | A0A023Z2C0, A0A025CW81, Q1R811, B7UHA6 |
| Cluster 162 | 19 | A0A023Z2G5, P0AGL8, P0AGL7, D3QR51, AID79982, ACT73659, ACT27862, ACI35227, A7ZR69, A0A025CRS7, Q1R781, E3PIL8, C8UFG2, C8UAV2, C8TGW8, A0A0E2U7E0, A0A0E0XUI6, A0A070CJ56, B7UHZ3 |
| Cluster 163 | 19 | A0A023Z2H3, A0A025CYQ8, P23845, A0A070CIW8, Q8FEJ1, Q1R7U0, C8TFZ9, AID79794, D3QPU8, ACT73452, ACI38986, A0A0E0XYQ7, C8UEV9, E3PHQ8, ACT28039, A7ZQJ5, C8U9I3, B7UHH0, A0A0E2THW0 |
| Cluster 164 | 4 | A0A023Z2I2, A0A025CX35, Q46901, E3PHR7 |
| Cluster 165 | 18 | A0A023Z2K2, A0A025CYL4, B7UHK1, P55135, E3PHU2, A7ZQM8, Q8FEG6, AID79820, D3QPY4, ACT73488, ACT28010, ACI38497, A0A0E2U7U6, C8UAB7, C8TG32, A0A0E0XV15, Q1R7Q7, A0A070CHJ3 |
| Cluster 166 | 19 | A0A023Z2M5, A0A025CSR2, P25736, E3PIL7, C8UFG1, C8UAV1, C8TGW7, A7ZR68, A0A0E2TQ49, A0A0E0XW38, A0A070CH90, ACT27863, Q1R782, D3QR50, ACT73658, ACI35282, AID79981, A0A0H2VCU8, B7UHZ2 |
| Cluster 167 | 15 | A0A023Z2N4, A0A025CT22, A0A0E2TJS7, A0A0E0Y516, A0A0E0Y810, A0A0E0Y8H6, A7ZVE6, A7ZH36, Q1R201, A7ZGL5, A7ZRA4, Q47688, D0Z6R2, A0A0H2VC93, A0A0H2VDW4 |
| Cluster 168 | 19 | A0A023Z2R9, Q1R6R5, P68680, P68679, E3PJY0, D3QS11, C8UG24, C8TX01, C8TH84, B7UIX3, AID80211, ACT73777, ACT27745, ACI35001, A7ZRU7, A0A0E2TXU8, A0A0E0XVI6, A0A070DSA6, A0A025CU40 |
| Cluster 169 | 56 | A0A023Z2U5, Q1R7E1, P0A8N4, P0A8N3, E3PIG6, D3QQL4, AID79927, ACT27915, A0A025CBW4, C8UF96, C8UAP4, A7ZQZ9, A0A0E2TR61, A0A0E0XWI2, A0A070CJB6, C8TGQ2, ACT73602, ACI36981, B7UHT9, P0A8N5, E3PD51, D3QL51, C8U056, C8TN13, ACT74913, ACI39480, A0A0E0Y6S5, A0A025C2R6, A0A023Z6V6, A7ZUZ9, ACT30888, Q8FAT5, AID81330, Q1R3D0, A0A0E2TMQ6, A0A070CLE7, B7UPV1, A0A023Z7L2, Q1R3A2, P0A8N8, P0A8N7, E3PE26, D3QL77, C8UM29, C8U0C2, C8TNX9, B7UPX7, AID81375, ACT74940, ACT30861, ACI34762, A7ZV28, A0A0E2TKS9, A0A0E0Y542, A0A070CEX4, A0A025CL60 |
| Cluster 170 | 5 | A0A023Z2Y1, Q46865, E3PJE2, ACT27786, A0A025CT82 |
| Cluster 171 | 18 | A0A023Z2Y7, D3QS13, C8UG26, C8TX03, C8TH86, B7UIX5, ACT73780, ACI38285, A7ZRU9, A0A0E2TT91, A0A0E0XVQ7, A0A070DZY9, A0A025CVN1, Q1R6R2, E3PJY2, ACT27743, P00579, A0A0H2VB35 |
| Cluster 172 | 2 | A0A023Z2Z0, A0A025CM84 |
| Cluster 173 | 19 | A0A023Z310, Q8FD87, Q1R6H4, AID80300, A0A025CGH3, D3QSN6, C8TXG1, ACT73876, ACI36556, A7ZS61, A0A0E2TT00, A0A0E0XXA0, E3PK76, P05055, ACT27646, A0A070D6N4, C8UGC3, C8TI74, B7UJ59 |
| Cluster 174 | 19 | A0A023Z314, Q1R6G8, P0AFF7, P0AFF6, E3PK81, D3QSP1, C8TXG6, B7UJ64, AID80305, ACT27641, A7ZS67, A0A0E2TSZ4, A0A0E0XX96, A0A070D6N8, A0A025CGH9, C8UGC8, C8TI79, ACT73881, ACI37560 |
| Cluster 175 | 18 | A0A023Z328, Q1R6F0, P0AG48, E3PK96, D3QSQ6, C8UGE4, C8TXI1, C8TI94, B7UJ79, AID80327, ACT73896, ACT27626, ACI38352, A7ZS83, A0A0E2TSY2, A0A0E0XX81, A0A070D6Q5, A0A025CHC7 |
| Cluster 176 | 10 | A0A023Z329, C8TX58, A0A025CIL3, A0A0E0Y166, C8UMP3, C8TND6, ACT71083, ACI36774, A0A070DEU8, A0A0E2TXY9 |
| Cluster 177 | 19 | A0A023Z345, Q1R6H3, P0ADZ5, P0ADZ4, E3PK77, D3QSN7, C8UGC4, C8TXG2, C8TI75, B7UJ60, AID80301, ACT27645, A7ZS62, A0A0E2UA46, A0A0E0XU44, A0A070DF26, A0A025CMA0, ACT73877, ACI34692 |
| Cluster 178 | 54 | A0A023Z374, P0AGL3, P0AGL2, E3PK25, C8UG73, C8TXA9, C8THD0, B7UJ17, AID80250, ACT73823, ACT27696, ACI35903, A0A0E2UA97, A0A0E0XVE1, A0A070DEY8, A0A025CGC3, D3QS56, Q1R6M0, A7ZS03, P0AF94, P0AF93, E3PEB3, C8UM93, C8U0K9, C8TP66, B7UQQ3, AID81462, ACT75025, ACT30777, A0A0E2TPH3, A0A0E0Y4M0, A0A070CLN0, A0A025CL90, A0A023Z713, D3QLG2, ACI35865, Q1R312, A7ZVC5, P39332, C8UM97, C8U0L3, A7ZVC9, A0A0E2TKQ8, A0A0E0Y650, E3PEB7, D3QLG6, ACT75030, ACI36168, A0A070CEK8, B7UQQ7, C8TP70, ACT30774, A0A023Z716, A0A025CS59 |
| Cluster 179 | 19 | A0A023Z377, A0A025CGL6, Q1R6D2, C8UGG0, C8TIB0, AID80343, ACT73912, ACT27610, ACI36079, A7ZSA1, A0A0H2VDV8, A0A0E2UA15, A0A0E0XU09, A0A070DF57, B7UJU2, D3QSS2, P24255, E3PKQ0, C8TXJ7 |
| Cluster 180 | 19 | A0A023Z3C5, P0A705, E3PK80, D3QSP0, C8TXG5, C8TI78, ACT73880, ACT27642, ACI39780, A7ZS65, A0A0E0XVE8, A0A070D8K2, A0A025CHA6, A0A0E2TTW4, C8UGC7, Q1R6H0, P59587, B7UJ63, AID80304 |
| Cluster 181 | 19 | A0A023Z3D0, Q1R6G3, P0AGA0, P0AG99, E3PK85, D3QSP5, C8TXH0, B7UJ68, AID80317, ACT73885, ACI39601, A0A0E0XVE4, A0A070D8K6, A0A025CGE6, C8UGD3, C8TI83, ACT27637, A7ZS71, A0A0E2TTW1 |
| Cluster 182 | 19 | A0A023Z3D6, Q1R6F7, P0AGK5, P0AGK4, E3PK90, D3QSQ0, C8UGD8, C8TXH5, C8TI88, B7UJ73, AID80321, ACT73890, ACT27632, ACI36534, A7ZS76, A0A0E2TTV7, A0A0E0XVE0, A0A070D8K9, A0A025CGF1 |
| Cluster 183 | 19 | A0A023Z3D8, Q1R637, P0A7Z5, P0A7Z4, E3PKX9, D3QTD4, C8UGP5, C8TXS5, C8TIJ7, B7UK19, AID80427, ACT73993, ACT27527, ACI35621, A7ZSI4, A0A0E2TZR9, A0A0E0XUZ9, A0A070D714, A0A025C4R9 |
| Cluster 184 | 19 | A0A023Z3E1, Q1R6F2, P0A7L9, P0A7L8, E3PK95, D3QSQ5, C8UGE3, C8TXI0, C8TI93, B7UJ78, AID80326, ACT73895, ACT27627, ACI36344, A7ZS81, A0A0E2TTV2, A0A0E0XVD6, A0A070D8L4, A0A025CGF5 |
| Cluster 185 | 19 | A0A023Z3E3, Q1R631, P0AGA3, P0AGA2, E3PKY4, D3QTD9, C8UHE8, C8TXT0, C8TIK2, B7UK24, AID80432, ACT73998, ACT27522, ACI39815, A7ZSI9, A0A0E2TZR4, A0A0E0XUZ5, A0A070D718, A0A025C4R5 |
| Cluster 186 | 19 | A0A023Z3E8, Q1R624, P0AG56, P0AG55, E3PKY9, D3QTE4, C8UHF3, C8TXT5, C8TIK7, B7UK29, AID80437, ACT74003, ACT27517, ACI37495, A7ZSJ4, A0A0E2TZQ9, A0A0E0XUZ1, A0A070D723, A0A025C4R0 |
| Cluster 187 | 19 | A0A023Z3F3, Q1R617, P0ADY4, P0ADY3, E3PKZ4, D3QTE9, C8UHF8, C8TXU0, C8TIL2, B7UK34, AID80442, ACT74008, ACT27512, ACI36392, A7ZSJ9, A0A0E2TZQ4, A0A0E0XUY6, A0A070D728, A0A025C4Q1 |
| Cluster 188 | 19 | A0A023Z3F8, Q1R610, P61176, P61175, E3PKZ9, D3QTF4, C8UHG3, C8TXU5, C8TIL7, B7UK39, AID80447, ACT74013, ACT27507, ACI39087, A7ZSK4, A0A0E2TZQ0, A0A0E0XUY0, A0A070D734, A0A025C4N4 |
| Cluster 189 | 19 | A0A023Z3G4, Q1R602, P60439, P60438, E3PL04, D3QTF9, C8UHG8, C8TXV0, C8TIM2, B7UK44, AID80452, ACT74018, ACT27502, ACI37815, A7ZSK9, A0A0E2TZP6, A0A0E0XUX6, A0A070D740, A0A025C4M8 |
| Cluster 190 | 5 | A0A023Z3G5, Q46864, E3PJE1, ACT27787, A0A025CU95 |
| Cluster 191 | 19 | A0A023Z3G6, Q1R636, P0A7V9, P0A7V8, E3PKY0, D3QTD5, C8UGP6, C8TXS6, C8TIJ8, B7UK20, AID80428, ACT73994, ACT27526, ACI35133, A7ZSI5, A0A0E0XTE1, A0A070DFD9, A0A025C3X9, A0A0E2TIG2 |
| Cluster 192 | 19 | A0A023Z3G9, Q1R630, P66071, D3QTE0, C8UHE9, C8TXT1, C8TIK3, B7UK25, AID80433, ACT73999, ACT27521, ACI34917, A7ZSJ0, A0A0E2TIE5, A0A0E0XTD7, A0A070DFE7, A0A025C3X5, P02413, E3PKY5 |
| Cluster 193 | 54 | A0A023Z3H1, Q1R5Y2, P0CE47, E3PLE7, D3QTG5, C8UHH2, C8TXV5, C8TIM7, B7UK49, AID80471, ACT74023, ACT27481, ACI35462, A7ZSL4, A0A0E0XUX1, A0A070D752, Q1R5U4, P0CE48, P0A6N2, E3PBU8, D3QYH9, C8UKN2, C8TZR2, C8TML9, B7UPD5, AID81156, ACT74739, ACT31037, ACI39782, A0A0E0Y717, A0A070DAP2, A0A023Z689, A7ZUJ2, A0A0E2TFI2, A0A025BUG5, A0A070D7V9, C8TME4, C8UIX8, B7ULE9, C8TZH7, A7ZTD8, P14081, E3PMV5, A0A0E2TIY4, D3QV15, Q1R504, ACT74305, ACI38303, AID80728, A0A0H2VDK3, ACT27217, A0A025CIM9, A0A023Z4N6, A0A0E0XU12 |
| Cluster 194 | 19 | A0A023Z3H4, Q1R622, P0A7W8, P0A7W7, E3PKZ0, D3QTE5, C8UHF4, C8TXT6, C8TIK8, B7UK30, AID80438, ACT74004, ACT27516, ACI38835, A7ZSJ5, A0A0E2TID9, A0A0E0XTD2, A0A070DFF3, A0A025C3X2 |
| Cluster 195 | 19 | A0A023Z3H8, Q1R6A9, P0AA11, P0AA10, E3PKS4, D3QSU5, C8UGI2, C8TXM0, C8TID2, B7UJW3, AID80364, ACT73935, ACT27586, ACI34734, A7ZSC5, A0A0E2TTS8, A0A0E0XX40, A0A070DF80, A0A025CGK4 |
| Cluster 196 | 19 | A0A023Z3I0, Q1R616, P0AG64, P0AG63, E3PKZ5, D3QTF0, C8UHF9, C8TXU1, C8TIL3, B7UK35, AID80443, ACT74009, ACT27511, ACI35942, A7ZSK0, A0A0E2TID6, A0A0E0XTC7, A0A070DFG0, A0A025C3W2 |
| Cluster 197 | 19 | A0A023Z3I4, Q1R609, P0A7U4, P0A7U3, E3PL00, D3QTF5, C8UHG4, C8TXU6, C8TIL8, B7UK40, AID80448, ACT74014, ACT27506, ACI35197, A7ZSK5, A0A0E2TID2, A0A0E0XTC3, A0A070DFG6, A0A025C3U5 |
| Cluster 198 | 19 | A0A023Z3I8, Q1R601, P0A7R6, P0A7R5, E3PL05, D3QTG0, C8UHG9, C8TXV1, C8TIM3, B7UK45, AID80453, ACT74019, ACT27501, ACI38219, A7ZSL0, A0A0E2TIC7, A0A0E0XTB8, A0A070DFH3, A0A025C3T6 |
| Cluster 199 | 19 | A0A023Z3J2, Q1R5U3, P0A6M9, P0A6M8, E3PLE8, D3QTG6, C8UHH3, C8TXV6, C8TIM8, B7UK50, AID80472, ACT74024, ACT27480, ACI35843, A7ZSL5, A0A0E2TQQ5, A0A0E0XTB3, A0A070DFH8, A0A025CRK0 |
| Cluster 200 | 19 | A0A023Z3J6, Q1R6F9, P0C0R9, P0C0R7, E3PK89, D3QSP9, C8TXH4, C8TI87, B7UJ72, AID80320, ACT73889, ACT27633, ACI34806, A7ZS75, A0A0E2TXJ9, A0A0E0XTM3, A0A070D703, A0A025CIY6, C8UGD7 |
| Cluster 201 | 19 | A0A023Z3L8, A0A025CT49, D3QS02, C8TWZ2, A0A070DS87, A7ZRT7, A0A0E0XTY6, A0A0E2TU74, P06961, E3PJX1, ACT27754, ACT73768, ACI35408, C8UG15, C8TH75, B7UIW4, Q1R6S5, Q8CXX6, AID80202 |
| Cluster 202 | 19 | A0A023Z3M5, P0ABS5, E3PJY1, D3QS12, C8TX02, ACT73778, ACT27744, ACI35393, A7ZRU8, A0A0E2TU63, A0A0E0XTX6, A0A025CT37, Q8FDG5, Q1R6R4, B7UIX4, AID80212, C8UG25, C8TH85, A0A070DSQ3 |
| Cluster 203 | 19 | A0A023Z3N5, P00954, ACT27435, A0A025CP33, Q1R5Q3, P67588, B7UK89, AID80510, ACT74059, ACI36148, C8UHL5, C8TY00, C8TIS2, A0A0E2TQT9, A0A0E0XUQ0, A0A070DFM5, D3QTK1, E3PLJ1, A7ZSQ9 |
| Cluster 204 | 19 | A0A023Z3N6, Q1R6B0, P0A7X4, P0A7X3, E3PKS3, D3QSU4, C8UGI1, C8TXL9, C8TID1, B7UJW2, AID80363, ACT73934, ACT27587, ACI35529, A7ZSC4, A0A0E2TXG4, A0A0E0XVA2, A0A070D6V4, A0A025CJ38 |
| Cluster 205 | 19 | A0A023Z3N8, Q1R638, P0AG45, P0AG44, E3PKX8, D3QTD3, C8UGP4, C8TXS4, C8TIJ6, B7UK18, AID80426, ACT73992, ACT27528, ACI38740, A7ZSI3, A0A0E2THZ6, A0A0E0XTT6, A0A070D8U1, A0A025C439 |
| Cluster 206 | 18 | A0A023Z3P4, Q1R632, P0A7Q6, E3PKY3, D3QTD8, C8UGP9, C8TXS9, C8TIK1, B7UK23, AID80431, ACT73997, ACT27523, ACI39036, A7ZSI8, A0A0E2THZ3, A0A0E0XTS8, A0A070D8U5, A0A025C436 |
| Cluster 207 | 19 | A0A023Z3Q1, Q1R626, P0C019, P0C018, E3PKY8, D3QTE3, C8UHF2, C8TXT4, C8TIK6, B7UK28, AID80436, ACT74002, ACT27518, ACI36761, A7ZSJ3, A0A0E2THY4, A0A0E0XTS4, A0A070D8V0, A0A025C431 |
| Cluster 208 | 19 | A0A023Z3Q7, P60624, E3PKZ3, D3QTE8, C8UHF7, C8TXT9, C8TIL1, ACT74007, ACT27513, ACI36541, A7ZSJ8, A0A0E2THY1, A0A0E0XTS0, A0A070D8V3, A0A025C419, Q8FD03, Q1R619, B7UK33, AID80441 |
| Cluster 209 | 19 | A0A023Z3R2, Q1R612, P0A7V4, P0A7V3, D3QTF3, C8UHG2, C8TXU4, C8TIL6, B7UK38, AID80446, ACT74012, ACT27508, ACI39328, A7ZSK3, A0A0E2THX7, A0A0E0XTR5, A0A070D8V7, A0A025C401, E3PKZ8 |
| Cluster 210 | 19 | A0A023Z3R6, A0A025CV28, D3QTM4, ACT74083, ACI37376, Q1R5M7, C8TY23, A7ZST5, AID80534, A0A0H2VBU9, A0A0E0XUM7, A0A070D923, P46837, ACT27410, A0A0E2TM45, E3PLL4, C8UHN8, C8TJJ7, B7UKB2 |
| Cluster 211 | 19 | A0A023Z3R7, Q1R604, P60724, P60723, E3PL03, D3QTF8, C8UHG7, C8TXU9, C8TIM1, B7UK43, AID80451, ACT74017, ACT27503, ACI37384, A7ZSK8, A0A0E2THX4, A0A0E0XTR1, A0A070D8W0, A0A025C3Z1 |
| Cluster 212 | 19 | A0A023Z3T3, ACT73980, ACI35356, A0A025CWS8, Q1R650, C8UGN3, C8TXR2, C8TII4, A7ZSH1, A0A0E0XV61, D3QTC2, A0A0E2TLS9, A0A070D8S7, P45748, E3PKW6, ACT27540, B7UK06, Q8FD17, AID80414 |
| Cluster 213 | 19 | A0A023Z3T6, A0A025CP73, E3PLM7, P38035, ACT27395, C8UHQ2, C8TJL1, D3QTP1, ACT74100, ACI38446, A7ZSV2, A0A0E0XTF9, A0A070D945, A0A0E2U4B8, C8TY36, B7UKC6, AID80548, A0A0H2VBK7, Q1R5L3 |
| Cluster 214 | 19 | A0A023Z3U1, C8TXR8, A7ZSH7, A0A0E2TI14, A0A0E0XWZ0, A0A025CA01, P36929, E3PKX2, B7UK12, ACT27534, A0A070D796, D3QTC8, ACT73986, ACI35065, C8UGN8, C8TIJ0, Q1R644, Q8FD12, AID80420 |
| Cluster 215 | 19 | A0A023Z3V3, Q1R633, P0A7T0, P0A7S9, E3PKY2, D3QTD7, C8UGP8, C8TXS8, C8TIK0, B7UK22, AID80430, ACT73996, ACT27524, ACI37486, A7ZSI7, A0A0E2TI09, A0A0E0XWX8, A0A070D7A7, A0A025C9Z4 |
| Cluster 216 | 19 | A0A023Z3V8, Q1R627, P0A7W2, P0A7W1, E3PKY7, D3QTE2, C8UHF1, C8TXT3, C8TIK5, B7UK27, AID80435, ACT74001, ACI36998, A7ZSJ2, A0A0E2TI06, A0A0E0XWX3, A0A070D7B3, A0A025C9Z0, ACT27519 |
| Cluster 217 | 19 | A0A023Z3W4, Q1R620, P62400, P62399, E3PKZ2, D3QTE7, C8UHF6, C8TXT8, C8TIL0, B7UK32, AID80440, ACT74006, ACT27514, ACI35008, A7ZSJ7, A0A0E2TI04, A0A0E0XWW8, A0A070D7B7, A0A025C9Y7 |
| Cluster 218 | 19 | A0A023Z3W8, Q1R613, P0ADY8, P0ADY7, E3PKZ7, D3QTF2, C8UHG1, C8TXU3, C8TIL5, B7UK37, AID80445, ACT74011, ACT27509, ACI39834, A7ZSK2, A0A0E2THZ9, A0A0E0XWW3, A0A070D7C2, A0A025C9X6 |
| Cluster 219 | 19 | A0A023Z3X3, Q1R606, P0ADZ1, P0ADZ0, E3PL02, D3QTF7, C8UHG6, C8TXU8, C8TIM0, B7UK42, AID80450, ACT74016, ACT27504, ACI39612, A7ZSK7, A0A0E2THZ5, A0A0E0XWV7, A0A070D7C6, A0A025C9V8 |
| Cluster 220 | 19 | A0A023Z3Y4, Q1R5U1, P0A7S4, P0A7S3, E3PLF0, D3QTG8, C8UHH5, C8TXV8, C8TIN0, B7UK52, AID80474, ACT74026, ACT27478, ACI34684, A7ZSL7, A0A0E2U435, A0A0E0XWU7, A0A070D7D6, A0A025CQ06 |
| Cluster 221 | 19 | A0A023Z401, A0A025CIX5, Q1R6H2, P60341, P60340, E3PK78, D3QSN8, C8TXG3, B7UJ61, AID80302, ACT27644, A0A0E2TUI5, A0A070D8H7, ACT73878, ACI38300, C8UGC5, C8TI76, A7ZS63, A0A0E0XV95 |
| Cluster 222 | 19 | A0A023Z410, Q8FD82, E3PK93, D3QSQ3, AID80324, ACT73893, ACI38165, A0A025CMB8, C8TXH8, B7UJ76, ACT27629, A7ZS79, A0A0E2TUH7, A0A0E0XV81, A0A070D8J4, P42641, C8UGE1, C8TI91, Q1R6F4 |
| Cluster 223 | 19 | A0A023Z412, A0A025CV40, D3QTN9, ACT74098, ACI38089, AID80546, Q1R5L5, C8UHQ0, C8TJK9, Q8FCS8, P46849, E3PLM5, ACT27397, C8TY35, A7ZSU9, A0A0E2TQW7, B7UKC4, A0A0E0XUT2, A0A070D9E3 |
| Cluster 224 | 13 | A0A023Z495, P46855, C8UHS4, C8TJN3, ACT27375, A0A070D9A2, C8TY60, A0A0E2TM78, A0A0E0XSY1, E3PLP6, A7ZSX5, AID80578, A0A0H2VE97 |
| Cluster 225 | 19 | A0A023Z4B4, Q1R635, P0A7S0, P0A7R9, E3PKY1, D3QTD6, C8UGP7, C8TXS7, C8TIJ9, B7UK21, AID80429, ACT73995, ACT27525, ACI36914, A7ZSI6, A0A0E2TLR6, A0A0E0XV46, A0A070D8U3, A0A025C6H1 |
| Cluster 226 | 19 | A0A023Z4C0, Q1R629, P0AG52, P0AG51, E3PKY6, D3QTE1, C8UHF0, C8TXT2, C8TIK4, B7UK26, AID80434, ACT74000, ACT27520, ACI36240, A7ZSJ1, A0A0E2TLR1, A0A0E0XV41, A0A070D8U7, A0A025C6G6 |
| Cluster 227 | 19 | A0A023Z4C1, A0A025CPE6, P0A821, E3PMV6, C8TZH6, ACT74306, ACT27216, ACI36031, A7ZTD9, A0A0E2TM95, A0A0E0XSF0, D3QV16, C8UIX9, C8TME3, Q8FCC1, Q1R503, A0A070DG92, AID80729, B7ULF0 |
| Cluster 228 | 19 | A0A023Z4C2, A0A025CRT7, E3PM54, C8UHU4, C8TJQ3, ACT74145, ACI37201, D3QU69, C8TY80, P10121, ACT27353, B7UL15, A0A070D979, A0A0E2TM99, A7ZT00, A0A0E0XWE7, Q1R5F6, AID80598, A0A0H2VBP4 |
| Cluster 229 | 19 | A0A023Z4C4, Q1R621, P0AG60, P0AG59, E3PKZ1, D3QTE6, C8UHF5, C8TXT7, C8TIK9, B7UK31, AID80439, ACT74005, ACT27515, ACI39418, A7ZSJ6, A0A0E2TLQ8, A0A0E0XV38, A0A070D8V1, A0A025C6G1 |
| Cluster 230 | 19 | A0A023Z4D1, Q1R615, P0A7M7, P0A7M6, E3PKZ6, D3QTF1, C8UHG0, C8TXU2, C8TIL4, B7UK36, AID80444, ACT74010, ACT27510, ACI37564, A7ZSK1, A0A0E2TLQ4, A0A0E0XV33, A0A070D8V5, A0A025C6F1 |
| Cluster 231 | 19 | A0A023Z4D7, Q1R607, P60423, P60422, E3PL01, D3QTF6, C8UHG5, C8TXU7, C8TIL9, B7UK41, AID80449, ACT74015, ACT27505, ACI34733, A7ZSK6, A0A0E2TLQ0, A0A0E0XV29, A0A070D8V9, A0A025C6D5 |
| Cluster 232 | 19 | A0A023Z4E8, P02359, Q1R5U2, P66606, E3PLE9, D3QTG7, C8UHH4, C8TXV7, C8TIM9, B7UK51, AID80473, ACT74025, ACT27479, ACI36782, A7ZSL6, A0A0E2TN06, A0A0E0XV22, A0A070D8W7, A0A025CP05 |
| Cluster 233 | 19 | A0A023Z4G0, A0A025CPI4, Q1R4V8, E3PMZ7, D3QV60, C8UJ22, C8TLJ3, B7ULJ0, ACT74351, ACI37701, A7ZTI6, A0A070DGC0, P05523, ACT27170, A0A0E2TJJ8, Q8FC87, AID80769, C8TZ82, A0A0E0XSB3 |
| Cluster 234 | 19 | A0A023Z4H7, Q1R4V6, P0A7P0, P0A7N9, E3PMZ8, D3QV61, C8UJ23, C8TZ81, C8TLJ2, B7ULJ1, AID80770, ACT74352, ACT27169, ACI39806, A7ZTI7, A0A0E2TN98, A0A0E0XU38, A0A070D9S2, A0A025CL50 |
| Cluster 235 | 19 | A0A023Z4K7, Q1R541, P0A9V6, P0A9V5, D3QUY6, C8UI41, C8TZL2, C8TMH9, B7ULB6, AID80695, ACT74273, ACT27257, ACI38927, A7ZTA2, A0A0E2TJ14, A0A0E0XT14, A0A070D9I2, A0A025CKZ8, E3PME4 |
| Cluster 236 | 19 | A0A023Z4L4, A0A025CIU7, P0AGJ2, E3PN13, D3QV76, C8UJ38, C8TLH7, ACT74367, ACT27153, ACI37696, A0A0E2TNB0, Q1R4U0, AID80785, A7ZTK3, A0A0H2VEU4, A0A070D9T7, C8TZ46, A0A0E0XU23, B7UM73 |
| Cluster 237 | 19 | A0A023Z4L6, A0A025CQ45, Q1R5N7, P0ACG9, P0ACG8, E3PLK7, D3QTL6, B7UKA4, AID80526, ACT74075, ACT27418, ACI36572, A0A0E0XT25, C8UHN1, C8TY16, C8TJJ0, A7ZSS6, A0A0E2TN57, A0A070D926 |
| Cluster 238 | 2 | A0A023Z4N5, A0A025CP91 |
| Cluster 239 | 19 | A0A023Z4Q1, A0A025CIP2, Q1R4Y9, P0AGJ8, P0AGJ7, E3PMX0, B7ULG2, AID80743, ACT27197, A0A0E2TJB1, D3QV34, C8UIZ6, C8TZA8, C8TLL9, ACT74325, ACI38932, A7ZTG0, A0A0E0XTZ2, A0A070D7X2 |
| Cluster 240 | 19 | A0A023Z4R8, Q1R536, P67025, E3PME9, D3QUZ0, C8UI45, C8TZK8, C8TMH5, B7ULC0, AID80699, ACT74277, ACT27251, ACI35203, A7ZTA6, A0A0E2U0G0, A0A0E0XW34, A0A070D9I7, A0A025CIK6, P00960 |
| Cluster 241 | 19 | A0A023Z4V8, Q1R4U2, P0A801, P0A800, E3PN11, D3QV74, C8UJ36, C8TZ48, C8TLH9, B7UM71, AID80783, ACT74365, ACT27155, ACI38873, A7ZTK1, A0A0E2TJF0, A0A0E0XTV3, A0A070D7Z9, A0A025CIQ9 |
| Cluster 242 | 19 | A0A023Z4W2, ACT31308, Q1R4N2, P0A7Y8, E3PNQ2, D3QW96, C8UJ92, C8TYZ3, C8TLA8, B7UMH1, AID80868, A7ZTR0, A0A0E2TJR8, A0A0E0Y655, A0A070D8H2, A0A025C5R7, ACT74468, ACI35360, Q8FBV5 |
| Cluster 243 | 19 | A0A023Z4Y2, P66679, D3QV68, C8TZ74, AID80777, ACT74359, ACI38139, A7ZTJ4, A0A025CJI9, E3PN05, C8UJ30, C8TLI5, ACT27162, A0A0E2U1B4, A0A0E0XSS6, A0A070D9R8, Q1R4U9, B7UM50, P0CG19 |
| Cluster 244 | 18 | A0A023Z506, Q1R4J2, P0A6U6, P0A6U5, E3PNT9, C8UJD1, C8TYV7, C8TL68, B7UMK5, AID80902, ACT74507, ACT31271, ACI38207, A0A0E2TP38, A0A0E0Y605, A0A070D8L3, A0A025C207, D3QWD7 |
| Cluster 245 | 11 | A0A023Z511, P62523, P62522, E3PNV8, D3QX61, C8UJF0, C8TYT8, B7UMM4, ACT74527, A0A070DA69, C8TL49 |
| Cluster 246 | 5 | A0A023Z520, D3QW59, ACT74432, ACI36949, A0A025BZP5 |
| Cluster 247 | 19 | A0A023Z529, Q1R4F5, P0AG31, P0AG30, E3PNX3, D3QX76, C8UJG5, C8TYS3, C8TL34, B7UMN8, AID80934, ACT74541, ACI39491, A7ZTY4, A0A0E2TPZ3, A0A0E0Y651, A0A070DA80, A0A025C639, ACT31238 |
| Cluster 248 | 19 | A0A023Z532, C8UHX7, C8TYB9, C8TJT6, AID80634, ACT27318, A0A0E2TR31, A0A0E0XW91, A0A025CVD5, Q1R5B7, A0A0H2VBS2, A7ZT35, E3PM90, B7UL47, A0A070DFT6, D3QUC7, ACT74203, ACI34840, P37634 |
| Cluster 249 | 19 | A0A023Z563, Q1R4N3, P0A7P6, P0A7P5, E3PNQ1, D3QW95, C8UJ91, C8TYZ4, C8TLA9, B7UMH0, AID80867, ACT74467, ACT31309, ACI35647, A7ZTQ9, A0A0E2TJK2, A0A0E0Y7U3, A0A070DA12, A0A025C264 |
| Cluster 250 | 27 | A0A023Z584, P0ABI5, P0ABI4, E3PAV6, D3QXA8, C8UJJ7, C8TYP1, C8TL02, B7UND3, AID80966, ACT74573, ACT31205, ACI37776, A7ZU21, A0A0E2TPW0, A0A0E0Y9Q0, A0A070DAA4, A0A025BZW5, Q1R4B8, A0A023Z5G9, A0A025C020, D3QXB1, ACI35295, A0A0E0Y5U6, A7ZU25, A0A0E2U391, A0A070D8G7 |
| Cluster 251 | 19 | A0A023Z593, Q1R537, P67030, D3QUY9, B7ULB9, AID80698, ACT74276, ACI38550, A0A025CJC1, C8UI44, C8TZK9, C8TMH6, A0A0E2TIJ9, A7ZTA5, A0A0E0XUB9, P00961, ACT27252, A0A070D823, E3PME8 |
| Cluster 252 | 19 | A0A023Z5A2, P0A887, E3PAX3, D3QXC8, C8UK98, C8TYM3, C8TKY4, B7UNG3, ACT74592, ACI39276, A7ZU40, A0A0E2TL53, A0A0E0Y7P0, A0A070DGU5, A0A025BSS6, Q8FBJ0, Q1R477, AID81006, ACT31172 |
| Cluster 253 | 13 | A0A023Z5B3, Q1R4M7, P0AD90, P0AD89, E3PNQ6, D3QWA1, C8UJ95, C8TYZ0, C8TLA5, B7UMH4, ACT74473, ACT31304, A0A0E0Y7T9 |
| Cluster 254 | 18 | A0A023Z5C6, P0AEG4, D3QXE8, C8UKB8, C8TYK3, C8TK74, ACT74612, ACI39649, A7ZU61, A0A0E2U5F2, A0A0E0Y7L1, A0A070D8L1, A0A025C7U9, Q1R455, P0A4L6, P0A4L5, AID81027, E3PAZ3 |
| Cluster 255 | 18 | A0A023Z5D2, P00582, ACT31148, A7ZU65, A0A070D8W8, A0A025C486, E3PAZ6, ACT74615, ACI39134, C8UKC1, A0A0E0Y5Q8, C8TYK0, D3QXF2, C8TK71, AID81031, A0A0E2TN73, B7UNI6, Q1R450 |
| Cluster 256 | 19 | A0A023Z5E8, Q1R4V5, P0A7M3, P0A7M2, E3PMZ9, D3QV62, C8UJ24, C8TZ80, C8TLJ1, B7ULJ2, AID80771, ACT74353, ACT27168, ACI36115, A7ZTI8, A0A0E2TJX2, A0A0E0XVW0, A0A070D891, A0A025CIN0 |
| Cluster 257 | 19 | A0A023Z5F7, Q1R431, P0A6M5, P0A6M4, E3PB20, D3QY83, C8UKE4, C8TYH7, C8TK48, B7UNK4, AID81052, ACT74642, ACT31123, ACI34995, A7ZU92, A0A0E2TP17, A0A0E0Y5X1, A0A070DAH2, A0A025C1P0 |
| Cluster 258 | 19 | A0A023Z5G8, A0A025CPL8, D3QV77, ACT74368, ACI36077, A0A0E2TJY5, A7ZTK4, AID80786, A0A0H2VC41, Q1R4T9, A0A0E0XVU4, C8TZ45, A0A070D8A7, P24230, C8UJ39, C8TLH6, ACT27152, E3PN14, B7UM74 |
| Cluster 259 | 19 | A0A023Z5H4, Q1R4B1, D3QXB7, ACI38922, A0A0H2VFB5, ACT31198, P15043, C8TKZ5, AID80972, ACT74581, A0A0E0Y5U1, A0A070DGT6, A0A025C609, C8UK87, C8TYN4, A0A0E2U386, E3PAW2, B7UND9, A7ZU29 |
| Cluster 260 | 19 | A0A023Z5P1, Q1R442, P32132, P0A3B2, P0A3B1, D3QXF9, C8UKC8, C8TYJ3, C8TK64, AID81038, ACT74625, ACT31139, ACI36999, A7ZU76, A0A0E2TP27, A0A0E0Y7B7, A0A070DGY0, A0A025C1U8, E3PB03 |
| Cluster 261 | 9 | A0A023Z5P9, P03061, E3PN84, D3QW70, B7UME4, A0A0H2VCG6, C8UJ59, C8TZ25, C8TLD9 |
| Cluster 262 | 19 | A0A023Z5S2, Q1R5V3, P0A8V3, P0A8V2, E3PBV5, D3QYI6, C8UKN9, C8TZR9, C8TMM6, B7UPE2, AID81163, ACT74746, ACT31030, ACI34743, A7ZUK1, A0A0E0Y7A2, A0A070DHB0, A0A025BN04, A0A0E2THH6 |
| Cluster 263 | 19 | A0A023Z5S6, A0A025C0E9, P25522, E3PNQ5, A0A0E2TK69, D3QW98, ACT74470, ACI34771, C8TYZ1, ACT31305, A0A0E0Y6D2, B7UMH3, A0A070DGI8, Q8FBV3, Q1R4M8, AID80870, A7ZTR2, C8TLA6, C8UJ94 |
| Cluster 264 | 19 | A0A023Z5T0, D3QYH2, A0A025C3V7, P23003, E3PBU2, C8TZQ6, ACT31042, A7ZUI5, A0A0E2TNT5, A0A0E0Y5P0, A0A070DAR2, ACT74733, ACI36341, Q1R3U2, AID81150, B7UNU5, Q8CXW3, C8UKM6, C8TML3 |
| Cluster 265 | 19 | A0A023Z5U0, Q1R5U7, P0A7J8, P0A7J7, E3PBV1, D3QYI2, C8UKN5, C8TZR5, C8TMM2, B7UPD8, AID81159, ACT74742, ACT31034, ACI38718, A7ZUJ6, A0A0E2TZB8, A0A0E0Y951, A0A070DAS2, A0A025BMB7 |
| Cluster 266 | 19 | A0A023Z5U6, P0A8T7, E3PBV6, D3QYI7, C8TZS0, C8TMM7, ACT74747, ACT31029, ACI38001, A7ZUK2, A0A0E2TZB3, A0A0E0Y945, A0A070DAS8, A0A025BMB0, Q1R5V4, AID81164, C8UKP0, B7UPE3, Q8FB83 |
| Cluster 267 | 19 | A0A023Z5V8, P0A6U4, P0A6U3, E3PNU0, C8TYV6, AID80903, A7ZTV2, A0A0E2TL22, A0A0E0Y7Y0, A0A025C5L1, A0A070DA18, B7UMK6, Q1R4J1, C8UJD2, C8TL67, D3QWD8, ACT74508, ACT31270, ACI39120 |
| Cluster 268 | 19 | A0A023Z600, P0C203, P0C076, P0A7M9, E3PB68, D3QYD6, C8UKJ6, C8TZC6, C8TJZ6, B7UNQ6, AID81103, ACT74699, ACT31071, ACI38441, A7ZUF1, A0A0E2TMX0, A0A0E0Y998, A0A070DAJ8, A0A025C1F3 |
| Cluster 269 | 19 | A0A023Z635, Q1R5U5, P0AG97, P0AG96, E3PBU9, D3QYI0, C8UKN3, C8TZR3, AID81157, ACT74740, ACT31036, ACI34859, A7ZUJ3, A0A0E2THK4, A0A0E0Y5F9, A0A070D908, A0A025BMF7, C8TMM0, B7UPD6 |
| Cluster 270 | 19 | A0A023Z640, Q1R5V1, P0A7K3, P0A7K2, E3PBV4, D3QYI5, C8UKN8, C8TZR8, C8TMM5, B7UPE1, AID81162, ACT74745, ACT31031, ACI36671, A7ZUK0, A0A0E2THK1, A0A0E0Y5F5, A0A070D915, A0A025BMF2 |
| Cluster 271 | 19 | A0A023Z650, P0AFX4, E3PBW4, D3QYJ5, C8UKP7, C8TZS7, C8TMN5, ACT74754, ACT31021, ACI35720, A7ZUL0, A0A0E2THJ6, A0A0E0Y5E8, A0A070DAR1, A0A025BME2, Q8FB76, AID81171, Q1R5W1, B7UPF0 |
| Cluster 272 | 19 | A0A023Z651, A0A025C3L4, A0A0E2TN38, A0A070CL90, E3PC69, C8ULQ3, ACI36838, A7ZUS0, P32695, D3QK55, C8TZX5, C8TMT9, ACT30969, A0A0E0Y6V4, ACT74829, Q8FB30, Q1R3N9, B7UPK8, AID81227 |
| Cluster 273 | 19 | A0A023Z657, A0A025C282, A0A0E2TN96, A0A070D955, C8UKJ5, C8TJZ7, A0A0E0Y7E3, E3PB67, D3QYD5, C8TZC7, A7ZUF0, ACT74698, ACI37616, B7UNQ5, P17888, ACT31072, A0A0H2VFH8, AID81102, Q1R3Y2 |
| Cluster 274 | 19 | A0A023Z681, B7UNG5, A0A070D8U9, A0A025BR00, Q8FBI8, Q1R475, AID81008, P0A6A0, D3QXD0, C8UKA0, C8TYM1, C8TKY2, ACT74594, ACT31170, ACI38121, A7ZU42, A0A0E2TM34, A0A0E0Y609, E3PAX5 |
| Cluster 275 | 19 | A0A023Z688, P0A698, E3PC77, C8ULR3, C8TZY5, C8TMU9, A7ZUS9, A0A025C3K7, Q1R3L2, D3QK65, B7UPM7, ACT74839, ACI37598, Q8FB02, AID81253, A0A070CL96, ACT30959, A0A0E0Y6U6, A0A0E2TJQ1 |
| Cluster 276 | 19 | A0A023Z692, Q1R5V0, P0A7J4, P0A7J3, E3PBV3, D3QYI4, C8UKN7, C8TZR7, C8TMM4, B7UPE0, AID81161, ACT74744, ACT31032, ACI39101, A7ZUJ8, A0A0E2TL93, A0A0E0Y713, A0A070DAP8, A0A025BTE4 |
| Cluster 277 | 19 | A0A023Z6P6, E3PE41, D3QL90, C8UM14, C8U0D5, ACT74953, ACT30848, ACI35414, A7ZV42, A0A0E2TKU4, A0A0E0Y527, A0A025CL44, Q1R387, B7UQI2, AID81388, A0A0H2VF40, A0A070CFQ9, P25519, C8TNZ4 |
| Cluster 278 | 19 | A0A023Z6R1, Q1R5U9, P0A7L1, P0A7L0, E3PBV2, D3QYI3, C8UKN6, C8TZR6, C8TMM3, AID81160, ACT74743, ACT31033, ACI35660, A7ZUJ7, A0A0E2THV2, A0A0E0Y5N1, A0A070D9C3, A0A025BPT9, B7UPD9 |
| Cluster 279 | 19 | A0A023Z6R3, A0A025CM22, E3PE32, D3QL81, C8UM23, C8U0C6, C8TNY5, ACT74944, ACT30857, ACI38051, A7ZV32, A0A0E2TPB8, A0A0E0Y6G1, A0A070CDT9, P39286, Q8FAL3, B7UPY1, AID81379, Q1R396 |
| Cluster 280 | 19 | A0A023Z6S9, C8TP00, A7ZV49, A0A0E0Y6F2, A0A070CDS4, A0A025CM37, P21499, C8U0E1, ACT30842, A0A0E2TPD0, D3QL96, ACT74959, ACI37473, B7UQI8, E3PE47, Q1R381, A0A0H2VG66, AID81394, C8UM08 |
| Cluster 281 | 19 | A0A023Z6T1, Q1R357, P0A7R2, P0A7R1, E3PE71, D3QLC0, C8ULY4, C8U0G4, C8TP23, B7UQL2, AID81418, ACT74983, ACT30818, ACI39651, A7ZV74, A0A0E2TKM3, A0A0E0Y4R7, A0A070CDR3, A0A025CL61 |
| Cluster 282 | 19 | A0A023Z6Y3, A0A025CNN4, A7ZV35, D3QL84, ACT74947, ACI36925, C8UM20, C8U0C9, C8TNY8, A0A0E0Y8J7, A0A0E2TKJ6, P31806, E3PE35, ACT30854, A0A070CDV1, B7UQH6, Q1R393, A0A0H2VDW3, AID81382 |
| Cluster 283 | 13 | A0A023Z6Y5, A0A025BM83, Q1R863, C8TVZ4, Q1R2A9, E3PDN8, A0A0H2V9Z3, A0A0E2TM18, E3PC34, A0A070DB19, C8U2N4, D3QMI3, A0A0E0XY53 |
| Cluster 284 | 19 | A0A023Z6Y7, Q1R388, P0A6X4, P0A6X3, E3PE40, D3QL89, C8UM15, C8U0D4, C8TNZ3, B7UQI1, AID81387, ACT74952, ACT30849, ACI39520, A7ZV41, A0A0E2TKJ9, A0A0E0Y8J2, A0A070CDU6, A0A025CNN8 |
| Cluster 285 | 19 | A0A023Z723, A0A025CLD7, D3QLH5, C8TP78, ACT75039, ACI39005, C8U0M1, A0A0E2U2U3, A0A070CEJ8, Q1R2Z6, B7UQR5, Q8FAD1, AID81474, ACT30765, C8UMZ5, A0A0E0Y854, P07118, E3PEC6, A7ZVD7 |
| Cluster 286 | 19 | A0A023Z725, Q1R358, P0A7T8, P0A7T7, E3PE70, D3QLB9, C8ULY5, C8U0G3, C8TP22, B7UQL1, AID81417, ACT74982, ACT30819, ACI39360, A7ZV73, A0A0E2U2P1, A0A0E0Y697, A0A070CLS7, A0A025CNQ9 |
| Cluster 287 | 5 | A0A023Z738, A0A025CS79, D3QM99, ACT75065, ACI37625 |
| Cluster 288 | 5 | A0A023Z742, A0A025CWL7, ACT75067, ACI38525, D3QMA1 |
| Cluster 289 | 19 | A0A023Z746, D3QL88, A0A0E2U2K9, A0A025CRZ9, P16384, E3PE39, C8U0D3, ACT30850, A7ZV40, A0A0E0Y6K5, A0A070CLV6, Q8CXV3, AID81386, C8UM16, C8TNZ2, B7UQI0, Q1R389, ACT74951, ACI39423 |
| Cluster 290 | 5 | A0A023Z7C8, A0A025CLF1, D3QM98, ACT75064, ACI39430 |
| Cluster 291 | 4 | A0A023Z7G4, A0A025BLA1, A0A0H2V632, AID78060 |
| Cluster 292 | 19 | A0A023Z7K5, E3PFA2, D3QMG3, C8UN80, C8U0X0, B7UQZ9, AID81541, ACT75128, ACI36181, A0A0E2TL94, A0A0E0Y7U4, A0A025CWC5, A0A070D7U2, C8TQC5, A7ZVR1, Q1R2D3, A0A0H2VGJ2, P28632, ACT30645 |
| Cluster 293 | 19 | A0A023Z7M0, A0A025CL63, P0A785, P0A784, E3PE33, C8UM22, C8U0C7, C8TNY6, B7UPY2, ACT30856, A7ZV33, A0A0E2TLJ2, A0A0E0Y4X5, A0A070CEW8, Q1R395, AID81380, D3QL82, ACT74945, ACI37453 |
| Cluster 294 | 19 | A0A023Z7N5, D3QND7, Q1R270, E3PFF7, A0A0E2TSM9, A0A025CK28, C8U147, P0A7I5, P0A7I4, B7UR02, ACT75131, ACT30642, ACI37894, A0A0E0Y467, AID81544, A7ZVR5, C8TQC8, C8UN83, A0A070D7K7 |
| Cluster 295 | 19 | A0A023Z7N7, Q1R380, P63178, P63177, D3QL97, C8U0E2, C8TP01, B7UQI9, AID81395, ACT74960, ACT30841, ACI38066, A7ZV51, A0A0E2TLK9, A0A0E0Y4W3, A0A070CLU6, A0A025CL76, E3PE48, C8UM07 |
| Cluster 296 | 19 | A0A023Z7R1, Q1R360, P0A4D0, E3PE68, D3QLB7, C8ULY7, C8U0G2, C8TP20, B7UQK9, AID81415, ACT74980, ACI37200, A7ZV71, A0A0E2TPE5, A0A0E0Y8D4, A0A070CDQ3, A0A025CL89, P02358, ACT30821 |
| Cluster 297 | 15 | A0A023Z817, D3QMC6, B7UQW9, A0A025CZ30, C8UN38, C8U0S9, A0A0E2TL57, A0A0E0Y4K4, A0A070D6C7, P39380, E3PF74, AID81507, ACT30693, A7ZVN0, C8TQ77 |
| Cluster 298 | 6 | A0A023Z876, C8TW36, A0A0E2TFK4, A0A025C334, A0A0E0YAG4, B7UTH5 |
| Cluster 299 | 13 | A0A023Z8B1, C8TW37, A0A0E2TXL1, A0A025BZG8, A7ZGW5, A0A0E0Y8N9, B7UTJ0, A0A0E0XW84, C8UE43, C8U7Z7, C8TVH0, A0A0E2TUR5, A0A070CGS8 |
| Cluster 300 | 4 | A0A025BLS8, A0A0E2TH37, A0A0E0Y6G7, D3QYQ5 |
| Cluster 301 | 3 | A0A025BMX8, A0A0E2TZ09, ACI39873 |
| Cluster 302 | 2 | A0A025BNL6, A0A0E2TKW9 |
| Cluster 303 | 2 | A0A025CGX4, A0A023YX61 |
| Cluster 304 | 3 | A0A025D203, Q46898, E3PHR4 |
| Cluster 305 | 8 | A0A070CEU0, C8UE44, C8U7Z8, C8TVH1, A7ZPS0, A0A0E2TTW0, A0A0E0XXE0, B7UTI9 |
| Cluster 306 | 8 | A0A070CF69, A0A0E2TL97, D3QXH8, A0A0E2TXN3, ACT29908, C8TUL4, D3QP34, B7ULR6 |
| Cluster 307 | 10 | A0A070CHL7, C8U9J1, A0A0E2TZP3, D3QPV7, ACT73461, ACI34532, A7ZQK3, A0A0E0XWV6, C8TG08, C8UEW8 |
| Cluster 308 | 10 | A0A070CIW5, A0A0E0XYQ2, A7ZQJ9, D3QPV3, ACT73457, ACI38339, A0A0E2THW6, C8UEW4, C8U9I8, C8TG04 |
| Cluster 309 | 10 | A0A070CJL6, C8U9J0, A7ZQK2, A0A0E0XV40, A0A0E2TIC3, D3QPV6, ACT73460, ACI36802, C8UEW7, C8TG07 |
| Cluster 310 | 10 | A0A070CQM7, A7ZQK0, D3QPV4, ACT73458, ACI35624, C8U9I9, C8TG05, A0A0E2THY9, A0A0E0XVJ1, C8UEW5 |
| Cluster 311 | 19 | A0A070DA32, P11989, C8UJB0, A7ZTS9, C8TL89, A0A0E2TP15, C8TYX4, Q1R4L3, A0A0H2VE51, ACT31288, AID80885, E3PNS2, B7UMI8, A0A0H2VA53, B7UHN3, Q1R7J9, AID79873, C8UF24, C8TG65 |
| Cluster 312 | 17 | A0A070DFQ6, C8UHP0, C8TJJ9, A0A0E2TQV9, A0A0E0XUU0, D3QTM6, C8TY25, ACT74085, ACI36080, A7ZST8, P33650, ACT27408, E3PLL6, Q8FCT7, AID80536, Q1R5M5, B7UKB4 |
| Cluster 313 | 4 | A0A070DGE1, A7ZVK2, A7ZR98, A0A0E0XUW0 |
| Cluster 314 | 5 | A0A0E0XTU1, C8UJ60, C8TZ24, C8TLD8, A5A627 |
| Cluster 315 | 2 | A0A0E0Y5H9, A7ZJH5 |
| Cluster 316 | 11 | A0A0E0Y5T0, Q8FA88, D3QMD9, ACT75105, ACI35501, Q1R2G2, C8TQ87, P39394, ACT30679, B7UQX7, AID81517 |
| Cluster 317 | 6 | A0A0E0Y5Y3, A0A0H2VDS2, C8TQ89, D3QME1, ACT75107, ACI37196 |
| Cluster 318 | 9 | A0A0E0Y6K1, C8UIB6, C8U214, C8TI18, A7ZI67, D3QJ91, ACI38846, Q1RFK9, ACT70260 |
| Cluster 319 | 6 | A0A0E0Y7W3, A0A0H2VE89, C8TQ90, ACT75108, ACI37771, D3QME2 |
| Cluster 320 | 2 | A0A0E2TJF1, C8URK6 |
| Cluster 321 | 3 | A0A0H2VBI1, AID80572, C8TY53 |
| Cluster 322 | 4 | A0A0H2VC51, AID80797, Q1R4S8, B7UM85 |
| Cluster 323 | 3 | A7ZR52, C8UFE6, C8TGV2 |
| Cluster 324 | 3 | A7ZR58, C8UFF1, C8TGV7 |
| Cluster 325 | 2 | ACI34484, ACT75217 |
| Cluster 326 | 3 | ACI35839, ACT71778, D3QT17 |
| Cluster 327 | 2 | ACI36684, ACT72512 |
| Cluster 328 | 7 | ACI37499, D3QWQ5, ACT70154, E3PDL2, ACT30388, Q47157, Q1RFT8 |
| Cluster 329 | 4 | ACI39076, C8UMG1, C8U0Z1, ACT73051 |
| Cluster 330 | 3 | ACI39825, ACT70130, D3QWN4 |
| Cluster 331 | 3 | ACT30675, Q1R2F8, P08956 |
| Cluster 332 | 2 | ACT72766, D3QKN6 |
| Cluster 333 | 2 | AID77171, Q1RG83 |
| Cluster 334 | 2 | B7UHA7, Q1R810 |
| Cluster 335 | 2 | B7UTG9, Q1R1X7 |
| Cluster 336 | 2 | C8UQG1, C8UQF6 |
| Cluster 337 | 1 | A0A023Z5V7 |
| Cluster 338 | 1 | A0A025BW30 |
| Cluster 339 | 1 | A0A070D853 |
| Cluster 340 | 1 | A0A0E0XVX0 |
| Cluster 341 | 1 | A0A0E0Y0B2 |
| Cluster 342 | 1 | A0A0E0Y5Z8 |
| Cluster 343 | 1 | A0A0E2THV7 |
| Cluster 344 | 1 | A0A0E2U1P2 |
| Cluster 345 | 1 | A0A0E2U2V5 |
| Cluster 346 | 1 | A0A0H2VDP7 |
| Cluster 347 | 1 | A0A0H2VFD1 |
| Cluster 348 | 1 | A7ZGX8 |
| Cluster 349 | 1 | A7ZQH1 |
| Cluster 350 | 1 | A7ZRD1 |
| Cluster 351 | 1 | A7ZVK4 |
| Cluster 352 | 1 | ACT29787 |
| Cluster 353 | 1 | ACT29923 |
| Cluster 354 | 1 | ACT75094 |
| Cluster 355 | 1 | AID81842 |
| Cluster 356 | 1 | B7UGD7 |
| Cluster 357 | 1 | B7UM48 |
| Cluster 358 | 1 | B7UMN3 |
| Cluster 359 | 1 | B7UQS6 |
| Cluster 360 | 1 | B7UQU1 |
| Cluster 361 | 1 | B7URP3 |
| Cluster 362 | 1 | C1P607 |
| Cluster 363 | 1 | C1P608 |
| Cluster 364 | 1 | C1P615 |
| Cluster 365 | 1 | C1P616 |
| Cluster 366 | 1 | C1P617 |
| Cluster 367 | 1 | C8U0N1 |
| Cluster 368 | 1 | C8U0N3 |
| Cluster 369 | 1 | C8U0N4 |
| Cluster 370 | 1 | C8U0N8 |
| Cluster 371 | 1 | C8UAL6 |
| Cluster 372 | 1 | C8UN08 |
| Cluster 373 | 1 | C8UR41 |
| Cluster 374 | 1 | C8URJ3 |
| Cluster 375 | 1 | C8URK7 |
| Cluster 376 | 1 | E3PD89 |
| Cluster 377 | 1 | E3PN33 |
| Cluster 378 | 1 | E3PN34 |
| Cluster 379 | 1 | E3PP14 |
| Cluster 380 | 1 | P52126 |
| Cluster 381 | 1 | P52129 |
| Cluster 382 | 1 | Q1R2Y1 |
| Cluster 383 | 1 | Q1R957 |
| Cluster 384 | 1 | Q1RBZ5 |
