# Supplementary material for: Bioinformatics comparisons of RNA-binding proteins of pathogenic and non-pathogenic Escherichia coli strains reveal novel virulence factors
Source: BMC Genomics. 2017 Aug 24;18:658. doi: 10.1186/s12864-017-4045-3 (PMC5571608; doi:10.1186/s12864-017-4045-3)
Supplement: Supplementary file 3 — RNA-binding proteins in all complete E. coli proteomes. All the RBPs obtained in the GWS of 166 E. coli strains have been listed here. The RefSeq IDs of the proteins are listed along with the total number of strains in which the protein is present mentioned in brackets. (DOC 185 kb) [file 12864_2017_4045_MOESM3_ESM.doc]

Additional File 1: RNA-binding proteins in all complete *E. coli* proteomes.

All the RBPs obtained in the GWS of 166 *E. coli* strains have been listed here. The RefSeq IDs of the proteins are listed along with the total number of strains in which the protein is present mentioned in brackets.

WP_000014594.1(160), WP_000062611.1(160), WP_000063125.1(160), WP_000065253.1(160), WP_000066490.1(160), WP_000091955.1(160), WP_000123197.1(160), WP_000124850.1(160), WP_000130100.1(160), WP_000135058.1(160), WP_000135199.1(160), WP_000256450.1(160), WP_000290727.1(160), WP_000617544.1(160), WP_000644741.1(160), WP_000710769.1(160), WP_000829818.1(160), WP_000831330.1(160), WP_000847559.1(160), WP_000868187.1(160), WP_000906486.1(160), WP_001051883.1(160), WP_001054420.1(160), WP_001085926.1(160), WP_001096200.1(160), WP_001138117.1(160), WP_001140433.1(160), WP_001181004.1(160), WP_001207201.1(160), WP_000135224.1(159), WP_000224099.1(159), WP_000271401.1(159), WP_000301864.1(159), WP_000358960.1(159), WP_000447529.1(159), WP_000588474.1(159), WP_000613955.1(159), WP_000622418.1(159), WP_000801125.1(159), WP_000940595.1(159), WP_000941212.1(159), WP_001051798.1(159), WP_001124225.1(159), WP_001128858.1(159), WP_001144069.1(159), WP_001216368.1(159), WP_001364348.1(159), WP_000028878.1(158), WP_000034825.1(158), WP_000087763.1(158), WP_000140327.1(158), WP_000145975.1(158), WP_000410785.1(158), WP_000529945.1(158), WP_000579833.1(158), WP_001196062.1(158), WP_001311244.1(158), WP_000043335.1(157), WP_000090775.1(157), WP_000455798.1(157), WP_000570668.1(157), WP_000947159.1(157), WP_001062678.1(157), WP_001068343.1(157), WP_001118930.1(157), WP_001162094.1(157), WP_001219652.1(157), WP_001274021.1(156), WP_001275702.1(156), WP_001700733.1(156), WP_000031783.1(155), WP_000031784.1(155), WP_000091945.1(155), WP_000438245.1(155), WP_000667319.1(155), WP_001295575.1(155), WP_000162574.1(154), WP_000719990.1(154), WP_000047539.1(153), WP_000263098.1(153), WP_000866436.1(153), WP_001293282.1(153), WP_000804726.1(152), WP_000424395.1(151), WP_000460035.1(151), WP_000818114.1(151), WP_000886683.1(151), WP_000940121.1(151), WP_001234850.1(151), WP_000059466.1(150), WP_000124700.1(150), WP_001054527.1(150), WP_001285288.1(150), WP_001118861.1(149), WP_000932839.1(148), WP_000027205.1(147), WP_000387388.1(146), WP_000042533.1(145), WP_000239730.1(145), WP_000004771.1(144), WP_000617723.1(143), WP_001029684.1(143), WP_001295493.1(143), WP_000179165.1(142), WP_000350058.1(141), WP_000807125.1(141), WP_001168544.1(141), WP_000246815.1(140), WP_000560983.1(140), WP_001031057.1(140), WP_001295188.1(139), WP_000254708.1(137), WP_000961458.1(137), WP_001216676.1(137), WP_000202996.1(135), WP_000152933.1(134), WP_000264777.1(134), WP_001110573.1(134), WP_001220233.1(134), WP_000089698.1(133), WP_001140652.1(133), WP_000573407.1(132), WP_001096684.1(132), WP_000809168.1(131), WP_001107167.1(130), WP_000944256.1(129), WP_000246882.1(128), WP_000790168.1(128), WP_000117881.1(127), WP_000047499.1(126), WP_000940019.1(126), WP_001045520.1(126), WP_000581937.1(124), WP_001295400.1(124), WP_000082101.1(123), WP_000729185.1(123), WP_000917883.1(123), WP_000994905.1(123), WP_000857405.1(122), WP_001126780.1(122), WP_001295553.1(121), WP_000934302.1(120), WP_001295556.1(120), WP_000133044.1(119), WP_000499788.1(119), WP_000003071.1(118), WP_000046749.1(118), WP_000725337.1(118), WP_001138043.1(118), WP_000531594.1(117), WP_000918827.1(117), WP_000131782.1(114), WP_000660483.1(114), WP_000653944.1(112), WP_001198386.1(111), WP_000150637.1(110), WP_000631384.1(110), WP_000219193.1(109), WP_000249410.1(109), WP_000551270.1(108), WP_000035581.1(107), WP_000505866.1(107), WP_000846343.1(107), WP_001320773.1(106), WP_000187530.1(105), WP_001117838.1(105), WP_001222509.1(105), WP_001238917.1(105), WP_001196486.1(104), WP_001117011.1(103), WP_000494183.1(102), WP_000525176.1(102), WP_001187819.1(100), WP_000186631.1(99), WP_000809051.1(99), WP_000634798.1(98), WP_000932347.1(98), WP_000985494.1(98), WP_001266503.1(98), WP_000888560.1(96), WP_000997403.1(96), WP_001297409.1(96), WP_000175457.1(95), WP_000357740.1(95), WP_000593994.1(95), WP_001065381.1(95), WP_001297048.1(94), WP_000956455.1(93), WP_001295074.1(93), WP_000140647.1(92), WP_001297484.1(92), WP_000175940.1(91), WP_000213294.1(91), WP_000254738.1(91), WP_000286500.1(91), WP_001070177.1(91), WP_001307570.1(91), WP_000138282.1(90), WP_000695655.1(90), WP_001144202.1(90), WP_001280345.1(90), WP_001287154.1(88), WP_001298300.1(88), WP_000206275.1(87), WP_000880182.1(86), WP_000007101.1(85), WP_000187022.1(85), WP_000808990.1(85), WP_001282281.1(84), WP_000569080.1(83), WP_001135732.1(83), WP_001223208.1(83), WP_000165552.1(82), WP_001307511.1(82), WP_000023635.1(79), WP_000890001.1(79), WP_001090506.1(79), WP_000018588.1(78), WP_001248691.1(78), WP_001300687.1(78), WP_000020749.1(77), WP_000250006.1(77), WP_000650107.1(77), WP_000956465.1(77), WP_001294757.1(77), WP_000950196.1(76), WP_000018596.1(75), WP_000460360.1(75), WP_000813254.1(75), WP_000060506.1(74), WP_000956071.1(74), WP_001247093.1(74), WP_001291217.1(74), WP_000230281.1(73), WP_000415584.1(73), WP_000416895.1(73), WP_000569430.1(73), WP_001151855.1(73), WP_001182957.1(73), WP_001256201.1(73), WP_001294700.1(73), WP_000066495.1(72), WP_000220396.1(72), WP_000569083.1(72), WP_000912345.1(72), WP_000982994.1(72), WP_001295363.1(72), WP_000079100.1(70), WP_000484984.1(70), WP_000555849.1(70), WP_001033695.1(70), WP_001299679.1(70), WP_001300753.1(70), WP_001340191.1(70), WP_000020737.1(69), WP_000954225.1(69), WP_000956458.1(69), WP_001033694.1(69), WP_000708487.1(68), WP_001114533.1(68), WP_001295251.1(68), WP_000138043.1(67), WP_000744778.1(67), WP_000891692.1(67), WP_000021036.1(66), WP_000078349.1(66), WP_000107012.1(66), WP_000128847.1(66), WP_000387770.1(66), WP_000673561.1(66), WP_000996005.1(66), WP_001075514.1(66), WP_001286597.1(66), WP_000087756.1(65), WP_000450594.1(65), WP_001264861.1(65), WP_001291772.1(65), WP_001300558.1(65), WP_000004473.1(64), WP_000123737.1(64), WP_000138270.1(64), WP_000611405.1(64), WP_001158931.1(64), WP_001301412.1(64), WP_001025322.1(63), WP_001258678.1(63), WP_000066484.1(62), WP_000235257.1(62), WP_000568943.1(62), WP_001282346.1(62), WP_001286857.1(62), WP_001295554.1(62), WP_000053099.1(61), WP_000132715.1(61), WP_000204337.1(61), WP_000829360.1(61), WP_000860273.1(61), WP_001157540.1(61), WP_000672380.1(60), WP_000937912.1(60), WP_000450588.1(59), WP_000578061.1(59), WP_000936377.1(59), WP_001090361.1(59), WP_001293612.1(59), WP_000096869.1(57), WP_000149156.1(57), WP_000245524.1(57), WP_000422182.1(57), WP_000456467.1(57), WP_001086539.1(57), WP_001272928.1(57), WP_001301279.1(57), WP_000907792.1(56), WP_001114543.1(56), WP_001301269.1(56), WP_000041970.1(55), WP_000437371.1(55), WP_000950183.1(55), WP_001105504.1(55), WP_001238914.1(55), WP_001291788.1(55), WP_001307844.1(55), WP_001339108.1(55), WP_000046812.1(54), WP_000173631.1(54), WP_000948863.1(54), WP_000996107.1(54), WP_001285536.1(54), WP_001293613.1(54), WP_001300574.1(54), WP_000141634.1(53), WP_000239579.1(53), WP_001158929.1(53), WP_001295202.1(53), WP_001300582.1(53), WP_000047184.1(52), WP_000194927.1(52), WP_000255039.1(52), WP_000668662.1(52), WP_000775955.1(52), WP_000950182.1(52), WP_001335950.1(52), WP_000041964.1(51), WP_000126265.1(51), WP_000281400.1(51), WP_000569315.1(51), WP_000939375.1(51), WP_001150457.1(51), WP_000193547.1(50), WP_000416392.1(50), WP_000555857.1(50), WP_000593938.1(50), WP_000678419.1(50), WP_000809057.1(50), WP_001136229.1(50), WP_000165543.1(49), WP_000635537.1(49), WP_001028612.1(49), WP_001193409.1(49), WP_001282283.1(49), WP_001283590.1(49), WP_001297709.1(49), WP_001340834.1(49), WP_000110945.1(48), WP_000176549.1(48), WP_000188144.1(48), WP_000431370.1(48), WP_000494186.1(48), WP_000594006.1(48), WP_000628576.1(48), WP_000673575.1(48), WP_000813200.1(48), WP_001107704.1(48), WP_001113989.1(48), WP_001272991.1(48), WP_000099534.1(47), WP_000138051.1(47), WP_000431381.1(47), WP_000493470.1(47), WP_001040654.1(47), WP_001043598.1(47), WP_001054909.1(47), WP_001115094.1(47), WP_001194860.1(47), WP_001263489.1(47), WP_001334996.1(47), WP_001352051.1(47), WP_000582468.1(46), WP_000752800.1(46), WP_000912385.1(46), WP_001223132.1(46), WP_001310858.1(46), WP_001350533.1(46), WP_000064450.1(45), WP_000827360.1(45), WP_000916281.1(45), WP_000938610.1(45), WP_000996007.1(45), WP_001028614.1(45), WP_001393574.1(45), WP_000175943.1(44), WP_001202177.1(44), WP_000057073.1(43), WP_000192349.1(43), WP_000219203.1(43), WP_000239577.1(43), WP_000460362.1(43), WP_000980727.1(43), WP_001146473.1(43), WP_001260717.1(43), WP_001283586.1(43), WP_001296798.1(43), WP_000076332.1(42), WP_000139543.1(42), WP_000416891.1(42), WP_000437376.1(42), WP_001050401.1(42), WP_001138045.1(42), WP_001149682.1(42), WP_001235649.1(42), WP_001291216.1(42), WP_000029466.1(41), WP_000110948.1(41), WP_000136402.1(41), WP_000155570.1(41), WP_000433152.1(41), WP_001238234.1(41), WP_001312198.1(41), WP_001138624.1(40), WP_001287134.1(40), WP_001301544.1(40), WP_000041976.1(39), WP_000140634.1(39), WP_000205805.1(39), WP_001025326.1(39), WP_001028626.1(39), WP_001070179.1(39), WP_001157406.1(39), WP_001291206.1(39), WP_000076316.1(38), WP_000132601.1(38), WP_000460357.1(38), WP_000505872.1(38), WP_001135738.1(38), WP_001157551.1(38), WP_001232948.1(38), WP_001272994.1(38), WP_001352260.1(38), WP_000133040.1(37), WP_000729186.1(37), WP_000808991.1(37), WP_000950198.1(37), WP_001046950.1(37), WP_001117834.1(37), WP_001182956.1(37), WP_001196477.1(37), WP_000057067.1(36), WP_000187545.1(36), WP_000255034.1(36), WP_000551259.1(36), WP_000673572.1(36), WP_000846342.1(36), WP_000904011.1(36), WP_001075531.1(36), WP_001295775.1(36), WP_001297427.1(36), WP_001303529.1(36), WP_000003806.1(35), WP_000021034.1(35), WP_000132446.1(35), WP_000150638.1(35), WP_001110630.1(35), WP_001264872.1(35), WP_001298740.1(35), WP_000029474.1(34), WP_000083664.1(34), WP_000128841.1(34), WP_000186633.1(34), WP_001182954.1(34), WP_001247089.1(34), WP_001260712.1(34), WP_000450589.1(33), WP_000635545.1(33), WP_001157535.1(33), WP_001282361.1(33), WP_001283598.1(33), WP_001295427.1(33), WP_000246884.1(32), WP_000653952.1(32), WP_000725344.1(32), WP_001296131.1(32), WP_001298330.1(32), WP_001338704.1(32), WP_000046754.1(31), WP_000081418.1(31), WP_000416407.1(31), WP_000437380.1(31), WP_000932342.1(31), WP_001146479.1(31), WP_001258662.1(31), WP_001136236.1(30), WP_000357763.1(29), WP_000450524.1(29), WP_000695662.1(29), WP_000934300.1(29), WP_000939359.1(29), WP_000956460.1(29), WP_001157896.1(29), WP_000004421.1(28), WP_000110954.1(28), WP_000138052.1(28), WP_000437375.1(28), WP_000602123.1(28), WP_000947905.1(28), WP_000981388.1(28), WP_001222508.1(28), WP_001247096.1(28), WP_000003068.1(27), WP_000173310.1(27), WP_000531601.1(27), WP_000635528.1(27), WP_001117001.1(27), WP_001136232.1(27), WP_001256174.1(27), WP_001273000.1(27), WP_001295704.1(27), WP_001296116.1(27), WP_001307383.1(27), WP_000156927.1(26), WP_000569423.1(26), WP_000622316.1(26), WP_000980706.1(26), WP_001272898.1(26), WP_001280359.1(26), WP_001312861.1(26), WP_000485012.1(25), WP_000744779.1(25), WP_001144199.1(25), WP_001146503.1(25), WP_001157890.1(25), WP_001202188.1(25), WP_001302184.1(25), WP_000046817.1(24), WP_000103863.1(24), WP_000126270.1(24), WP_000611411.1(24), WP_000668685.1(24), WP_001043561.1(24), WP_001150440.1(24), WP_001286823.1(24), WP_001295972.1(24), WP_001296291.1(24), WP_000165553.1(23), WP_000173200.1(23), WP_000439434.1(23), WP_000447335.1(23), WP_000573412.1(23), WP_000857399.1(23), WP_000889999.1(23), WP_000890008.1(23), WP_000907790.1(23), WP_000940018.1(23), WP_001282347.1(23), WP_001339629.1(23), WP_024179276.1(23), WP_000117262.1(22), WP_000173666.1(22), WP_000204335.1(22), WP_001065363.1(22), WP_001157412.1(22), WP_001198388.1(22), WP_001283585.1(22), WP_001296460.1(22), WP_000021040.1(21), WP_000047176.1(21), WP_000078344.1(21), WP_000126267.1(21), WP_000936370.1(21), WP_000948872.1(21), WP_000948882.1(21), WP_000956881.1(21), WP_000985490.1(21), WP_001260694.1(21), WP_000078916.1(20), WP_000126264.1(20), WP_000132630.1(20), WP_000387762.1(20), WP_000525208.1(20), WP_000672359.1(20), WP_001135731.1(20), WP_001151854.1(20), WP_001157407.1(20), WP_001193391.1(20), WP_001218658.1(20), WP_001315241.1(20), WP_001325907.1(20), WP_001719048.1(20), WP_000029464.1(19), WP_000053089.1(19), WP_000057022.1(19), WP_000057086.1(19), WP_000078907.1(19), WP_000079112.1(19), WP_000173679.1(19), WP_000416385.1(19), WP_000912352.1(19), WP_000936385.1(19), WP_000940874.1(19), WP_000950178.1(19), WP_000956456.1(19), WP_000983716.1(19), WP_001187810.1(19), WP_001193397.1(19), WP_001294702.1(19), WP_001294774.1(19), WP_000016205.1(18), WP_000029329.1(18), WP_000057977.1(18), WP_000079111.1(18), WP_000132912.1(18), WP_000139321.1(18), WP_000187001.1(18), WP_000230273.1(18), WP_000249989.1(18), WP_000255032.1(18), WP_000264790.1(18), WP_000431376.1(18), WP_000578103.1(18), WP_000586449.1(18), WP_000593936.1(18), WP_000668687.1(18), WP_000956067.1(18), WP_000997384.1(18), WP_001022015.1(18), WP_001090357.1(18), WP_001090514.1(18), WP_001107179.1(18), WP_001146509.1(18), WP_001149732.1(18), WP_001235611.1(18), WP_001258675.1(18), WP_001266506.1(18), WP_001282280.1(18), WP_001282368.1(18), WP_001296226.1(18), WP_001296259.1(18), WP_001305312.1(18), WP_001309484.1(18), WP_001341949.1(18), WP_001442069.1(18), WP_000007091.1(17), WP_000029460.1(17), WP_000047157.1(17), WP_000053080.1(17), WP_000053085.1(17), WP_000057985.1(17), WP_000076322.1(17), WP_000078338.1(17), WP_000096849.1(17), WP_000110940.1(17), WP_000117888.1(17), WP_000125640.1(17), WP_000126271.1(17), WP_000139528.1(17), WP_000152940.1(17), WP_000188152.1(17), WP_000194884.1(17), WP_000206261.1(17), WP_000254701.1(17), WP_000350057.1(17), WP_000420115.1(17), WP_000420117.1(17), WP_000422200.1(17), WP_000431368.1(17), WP_000431388.1(17), WP_000456450.1(17), WP_000493440.1(17), WP_000502513.1(17), WP_000521559.1(17), WP_000555842.1(17), WP_000560981.1(17), WP_000568920.1(17), WP_000569347.1(17), WP_000578064.1(17), WP_000578079.1(17), WP_000631387.1(17), WP_000634830.1(17), WP_000660487.1(17), WP_000668686.1(17), WP_000672320.1(17), WP_000740046.1(17), WP_000744765.1(17), WP_000827406.1(17), WP_000860311.1(17), WP_000891663.1(17), WP_000907823.1(17), WP_000917890.1(17), WP_000918851.1(17), WP_000935259.1(17), WP_000936388.1(17), WP_000950188.1(17), WP_000983009.1(17), WP_000985509.1(17), WP_000996111.1(17), WP_001029685.1(17), WP_001040681.1(17), WP_001086497.1(17), WP_001090370.1(17), WP_001096687.1(17), WP_001097132.1(17), WP_001114009.1(17), WP_001149713.1(17), WP_001193415.1(17), WP_001202204.1(17), WP_001216675.1(17), WP_001248677.1(17), WP_001258676.1(17), WP_001260690.1(17), WP_001266492.1(17), WP_001280339.1(17), WP_001293592.1(17), WP_001294708.1(17), WP_001294788.1(17), WP_001295781.1(17), WP_001295896.1(17), WP_001295930.1(17), WP_001295968.1(17), WP_001296006.1(17), WP_001296445.1(17), WP_001296481.1(17), WP_001296625.1(17), WP_001303793.1(17), WP_001304373.1(17), WP_001313744.1(17), WP_001314420.1(17), WP_001321003.1(17), WP_001350700.1(17), WP_001531894.1(17), WP_023142319.1(17), WP_000004770.1(16), WP_000125646.1(16), WP_000173201.1(16), WP_000176523.1(16), WP_000186998.1(16), WP_000202990.1(16), WP_000204320.1(16), WP_000206260.1(16), WP_000220422.1(16), WP_000230277.1(16), WP_000254745.1(16), WP_000254750.1(16), WP_000387752.1(16), WP_000387753.1(16), WP_000593914.1(16), WP_000708500.1(16), WP_000775946.1(16), WP_000813195.1(16), WP_000891683.1(16), WP_000911329.1(16), WP_000918810.1(16), WP_000937933.1(16), WP_000939399.1(16), WP_000956074.1(16), WP_000982997.1(16), WP_000996092.1(16), WP_001075518.1(16), WP_001090386.1(16), WP_001135715.1(16), WP_001238258.1(16), WP_001295619.1(16), WP_001296139.1(16), WP_001297366.1(16), WP_001297549.1(16), WP_001300912.1(16), WP_001301437.1(16), WP_001301572.1(16), WP_001353768.1(16), WP_001531210.1(16), WP_000057107.1(15), WP_000078339.1(15), WP_000188180.1(15), WP_000205813.1(15), WP_000206445.1(15), WP_000250007.1(15), WP_000456454.1(15), WP_000476153.1(15), WP_000578056.1(15), WP_000590258.1(15), WP_000611428.1(15), WP_000622545.1(15), WP_000660485.1(15), WP_000888559.1(15), WP_000916291.1(15), WP_000950181.1(15), WP_000994906.1(15), WP_000995994.1(15), WP_001069668.1(15), WP_001090493.1(15), WP_001114026.1(15), WP_001126777.1(15), WP_001144190.1(15), WP_001168560.1(15), WP_001182418.1(15), WP_001187785.1(15), WP_001192027.1(15), WP_001218657.1(15), WP_001235579.1(15), WP_001238241.1(15), WP_001248695.1(15), WP_001263493.1(15), WP_001272924.1(15), WP_001296497.1(15), WP_001296993.1(15), WP_001350562.1(15), WP_001362982.1(15), WP_001485179.1(15), WP_001531905.1(15), WP_000018429.1(14), WP_000079107.1(14), WP_000132643.1(14), WP_000176578.1(14), WP_000213308.1(14), WP_000233452.1(14), WP_000422188.1(14), WP_000456571.1(14), WP_000528869.1(14), WP_000569419.1(14), WP_000654452.1(14), WP_000813212.1(14), WP_000813215.1(14), WP_000887491.1(14), WP_000907827.1(14), WP_000932344.1(14), WP_000983710.1(14), WP_000996091.1(14), WP_000997411.1(14), WP_001031055.1(14), WP_001043577.1(14), WP_001193413.1(14), WP_001223181.1(14), WP_001238231.1(14), WP_001295786.1(14), WP_001298774.1(14), WP_001301504.1(14), WP_001318538.1(14), WP_001326256.1(14), WP_000004432.1(13), WP_000016207.1(13), WP_000068009.1(13), WP_000085058.1(13), WP_000099483.1(13), WP_000099521.1(13), WP_000099530.1(13), WP_000107031.1(13), WP_000123746.1(13), WP_000126269.1(13), WP_000128874.1(13), WP_000132718.1(13), WP_000153099.1(13), WP_000173684.1(13), WP_000187543.1(13), WP_000210557.1(13), WP_000235245.1(13), WP_000357755.1(13), WP_000550703.1(13), WP_000578076.1(13), WP_000586468.1(13), WP_000587626.1(13), WP_000617726.1(13), WP_000631389.1(13), WP_000634789.1(13), WP_000678405.1(13), WP_000775931.1(13), WP_000775933.1(13), WP_000790169.1(13), WP_000829367.1(13), WP_000866321.1(13), WP_000916271.1(13), WP_000916310.1(13), WP_000936361.1(13), WP_000937876.1(13), WP_000938582.1(13), WP_000956868.1(13), WP_000972087.1(13), WP_000980740.1(13), WP_001013636.1(13), WP_001034044.1(13), WP_001040659.1(13), WP_001057134.1(13), WP_001065365.1(13), WP_001187827.1(13), WP_001223200.1(13), WP_001232966.1(13), WP_001235630.1(13), WP_001261286.1(13), WP_001294798.1(13), WP_001295535.1(13), WP_001296537.1(13), WP_001300922.1(13), WP_001301344.1(13), WP_001301726.1(13), WP_001306448.1(13), WP_001315912.1(13), WP_001338705.1(13), WP_001339550.1(13), WP_032140777.1(13), WP_000004454.1(12), WP_000004459.1(12), WP_000007095.1(12), WP_000020744.1(12), WP_000023640.1(12), WP_000029463.1(12), WP_000046824.1(12), WP_000047170.1(12), WP_000053083.1(12), WP_000057080.1(12), WP_000057976.1(12), WP_000076340.1(12), WP_000078833.1(12), WP_000078920.1(12), WP_000079092.1(12), WP_000096891.1(12), WP_000099535.1(12), WP_000103571.1(12), WP_000110950.1(12), WP_000117895.1(12), WP_000123789.1(12), WP_000132599.1(12), WP_000132712.1(12), WP_000140631.1(12), WP_000147776.1(12), WP_000149132.1(12), WP_000150612.1(12), WP_000188174.1(12), WP_000194871.1(12), WP_000204340.1(12), WP_000281463.1(12), WP_000286494.1(12), WP_000456466.1(12), WP_000499793.1(12), WP_000525162.1(12), WP_000525186.1(12), WP_000555853.1(12), WP_000569336.1(12), WP_000586497.1(12), WP_000611407.1(12), WP_000668690.1(12), WP_000695640.1(12), WP_000708473.1(12), WP_000775988.1(12), WP_000809182.1(12), WP_000813185.1(12), WP_000813190.1(12), WP_000827117.1(12), WP_000829294.1(12), WP_000829314.1(12), WP_000860282.1(12), WP_000880185.1(12), WP_000889984.1(12), WP_000891671.1(12), WP_000912351.1(12), WP_000916267.1(12), WP_000937887.1(12), WP_000938639.1(12), WP_000940880.1(12), WP_000948865.1(12), WP_000983028.1(12), WP_001033699.1(12), WP_001041505.1(12), WP_001065380.1(12), WP_001075526.1(12), WP_001090366.1(12), WP_001090487.1(12), WP_001107648.1(12), WP_001114546.1(12), WP_001136210.1(12), WP_001140649.1(12), WP_001144201.1(12), WP_001149719.1(12), WP_001149763.1(12), WP_001150441.1(12), WP_001150472.1(12), WP_001193352.1(12), WP_001223210.1(12), WP_001232889.1(12), WP_001248690.1(12), WP_001256214.1(12), WP_001260716.1(12), WP_001260720.1(12), WP_001263495.1(12), WP_001263500.1(12), WP_001273005.1(12), WP_001279407.1(12), WP_001282345.1(12), WP_001287136.1(12), WP_001291774.1(12), WP_001295766.1(12), WP_001298466.1(12), WP_001298974.1(12), WP_001300557.1(12), WP_001301615.1(12), WP_001301620.1(12), WP_001301674.1(12), WP_001301854.1(12), WP_001301861.1(12), WP_001301955.1(12), WP_001302029.1(12), WP_001302216.1(12), WP_001302468.1(12), WP_001303279.1(12), WP_001303804.1(12), WP_001303983.1(12), WP_001312208.1(12), WP_001314234.1(12), WP_001327954.1(12), WP_001331962.1(12), WP_001332154.1(12), WP_001350310.1(12), WP_001351125.1(12), WP_001412115.1(12), WP_023438539.1(12), WP_000047198.1(11), WP_000047209.1(11), WP_000078319.1(11), WP_000099491.1(11), WP_000103579.1(11), WP_000103864.1(11), WP_000128839.1(11), WP_000152768.1(11), WP_000156933.1(11), WP_000176515.1(11), WP_000187008.1(11), WP_000194888.1(11), WP_000204321.1(11), WP_000220436.1(11), WP_000230274.1(11), WP_000235242.1(11), WP_000250046.1(11), WP_000422239.1(11), WP_000447331.1(11), WP_000499800.1(11), WP_000502504.1(11), WP_000531578.1(11), WP_000568928.1(11), WP_000568934.1(11), WP_000569345.1(11), WP_000569405.1(11), WP_000582432.1(11), WP_000611410.1(11), WP_000634747.1(11), WP_000635525.1(11), WP_000672426.1(11), WP_000678408.1(11), WP_000695661.1(11), WP_000708479.1(11), WP_000744766.1(11), WP_000752798.1(11), WP_000775938.1(11), WP_000827370.1(11), WP_000860259.1(11), WP_000860295.1(11), WP_000887477.1(11), WP_000902687.1(11), WP_000916269.1(11), WP_000916325.1(11), WP_000937882.1(11), WP_000948883.1(11), WP_000956043.1(11), WP_000956879.1(11), WP_000967408.1(11), WP_000980739.1(11), WP_000994894.1(11), WP_001025351.1(11), WP_001040684.1(11), WP_001043587.1(11), WP_001045527.1(11), WP_001086511.1(11), WP_001086557.1(11), WP_001097119.1(11), WP_001105503.1(11), WP_001110564.1(11), WP_001114008.1(11), WP_001150456.1(11), WP_001150487.1(11), WP_001198385.1(11), WP_001223177.1(11), WP_001238202.1(11), WP_001261287.1(11), WP_001264869.1(11), WP_001291771.1(11), WP_001294674.1(11), WP_001296749.1(11), WP_001301497.1(11), WP_001302008.1(11), WP_001350069.1(11), WP_001350159.1(11), WP_001350819.1(11), WP_001350873.1(11), WP_001363705.1(11), WP_032140800.1(11), WP_000003075.1(10), WP_000021019.1(10), WP_000021035.1(10), WP_000029325.1(10), WP_000046790.1(10), WP_000046816.1(10), WP_000057094.1(10), WP_000059467.1(10), WP_000076303.1(10), WP_000082099.1(10), WP_000089719.1(10), WP_000096843.1(10), WP_000107027.1(10), WP_000107036.1(10), WP_000123782.1(10), WP_000131775.1(10), WP_000139567.1(10), WP_000149183.1(10), WP_000153089.1(10), WP_000173308.1(10), WP_000173697.1(10), WP_000176537.1(10), WP_000176582.1(10), WP_000186648.1(10), WP_000188147.1(10), WP_000193529.1(10), WP_000194914.1(10), WP_000194940.1(10), WP_000220402.1(10), WP_000220411.1(10), WP_000239733.1(10), WP_000286501.1(10), WP_000361402.1(10), WP_000387779.1(10), WP_000387782.1(10), WP_000416382.1(10), WP_000422211.1(10), WP_000485019.1(10), WP_000493462.1(10), WP_000493513.1(10), WP_000494181.1(10), WP_000521577.1(10), WP_000569434.1(10), WP_000582452.1(10), WP_000586451.1(10), WP_000593900.1(10), WP_000593922.1(10), WP_000622314.1(10), WP_000635546.1(10), WP_000668700.1(10), WP_000672328.1(10), WP_000708496.1(10), WP_000734304.1(10), WP_000819018.1(10), WP_000827413.1(10), WP_000829372.1(10), WP_000882826.1(10), WP_000890002.1(10), WP_000907770.1(10), WP_000936350.1(10), WP_000937457.1(10), WP_000938577.1(10), WP_000948855.1(10), WP_000956871.1(10), WP_000956885.1(10), WP_000957426.1(10), WP_000961820.1(10), WP_000983019.1(10), WP_000985501.1(10), WP_001013637.1(10), WP_001025306.1(10), WP_001028610.1(10), WP_001034046.1(10), WP_001043606.1(10), WP_001044768.1(10), WP_001069760.1(10), WP_001084082.1(10), WP_001089388.1(10), WP_001105471.1(10), WP_001105473.1(10), WP_001117844.1(10), WP_001138626.1(10), WP_001157413.1(10), WP_001157550.1(10), WP_001189113.1(10), WP_001194917.1(10), WP_001218659.1(10), WP_001223147.1(10), WP_001223199.1(10), WP_001232881.1(10), WP_001235600.1(10), WP_001238194.1(10), WP_001248685.1(10), WP_001256184.1(10), WP_001261278.1(10), WP_001264864.1(10), WP_001273001.1(10), WP_001280341.1(10), WP_001283251.1(10), WP_001285523.1(10), WP_001286577.1(10), WP_001286813.1(10), WP_001286832.1(10), WP_001286879.1(10), WP_001294772.1(10), WP_001296192.1(10), WP_001296342.1(10), WP_001297198.1(10), WP_001299207.1(10), WP_001301008.1(10), WP_001301498.1(10), WP_001301795.1(10), WP_001302304.1(10), WP_001303805.1(10), WP_001304008.1(10), WP_001304009.1(10), WP_001304205.1(10), WP_001304269.1(10), WP_001305013.1(10), WP_001307251.1(10), WP_001313630.1(10), WP_001327262.1(10), WP_001331495.1(10), WP_001350597.1(10), WP_001351158.1(10), WP_001362899.1(10), WP_001531895.1(10), WP_010904951.1(10), WP_024179275.1(10), WP_042007029.1(10), WP_000004944.1(9), WP_000029342.1(9), WP_000029471.1(9), WP_000046810.1(9), WP_000047185.1(9), WP_000081417.1(9), WP_000082110.1(9), WP_000131757.1(9), WP_000132612.1(9), WP_000132905.1(9), WP_000139522.1(9), WP_000139551.1(9), WP_000139561.1(9), WP_000139585.1(9), WP_000173665.1(9), WP_000188194.1(9), WP_000193517.1(9), WP_000194943.1(9), WP_000202563.1(9), WP_000204338.1(9), WP_000210558.1(9), WP_000213297.1(9), WP_000255051.1(9), WP_000357744.1(9), WP_000416403.1(9), WP_000484965.1(9), WP_000485006.1(9), WP_000493466.1(9), WP_000493471.1(9), WP_000499751.1(9), WP_000505877.1(9), WP_000568970.1(9), WP_000569344.1(9), WP_000582441.1(9), WP_000582465.1(9), WP_000586445.1(9), WP_000628537.1(9), WP_000628583.1(9), WP_000633911.1(9), WP_000635543.1(9), WP_000635550.1(9), WP_000671689.1(9), WP_000813220.1(9), WP_000827411.1(9), WP_000827437.1(9), WP_000880195.1(9), WP_000911330.1(9), WP_000937974.1(9), WP_000940869.1(9), WP_000944245.1(9), WP_000961821.1(9), WP_000997387.1(9), WP_001013642.1(9), WP_001025318.1(9), WP_001028465.1(9), WP_001039799.1(9), WP_001040644.1(9), WP_001061411.1(9), WP_001075536.1(9), WP_001146512.1(9), WP_001149743.1(9), WP_001151866.1(9), WP_001157886.1(9), WP_001189111.1(9), WP_001202189.1(9), WP_001223167.1(9), WP_001285557.1(9), WP_001295090.1(9), WP_001297428.1(9), WP_001300932.1(9), WP_001300978.1(9), WP_001301026.1(9), WP_001301369.1(9), WP_001302074.1(9), WP_001303086.1(9), WP_001309606.1(9), WP_001320043.1(9), WP_001341929.1(9), WP_001346040.1(9), WP_001351030.1(9), WP_001353941.1(9), WP_001387312.1(9), WP_001411676.1(9), WP_001433934.1(9), WP_001442133.1(9), WP_012767712.1(9), WP_012767721.1(9), WP_012767773.1(9), WP_012767774.1(9), WP_032160286.1(9), WP_038432595.1(9), WP_038432600.1(9), WP_038432623.1(9), WP_038432669.1(9), WP_038432672.1(9), WP_038432674.1(9), WP_038432678.1(9), WP_000007116.1(8), WP_000007119.1(8), WP_000046785.1(8), WP_000047491.1(8), WP_000126268.1(8), WP_000139341.1(8), WP_000173324.1(8), WP_000186627.1(8), WP_000193113.1(8), WP_000193538.1(8), WP_000205834.1(8), WP_000213277.1(8), WP_000239581.1(8), WP_000450532.1(8), WP_000568924.1(8), WP_000568946.1(8), WP_000582429.1(8), WP_000586465.1(8), WP_000590243.1(8), WP_000602103.1(8), WP_000602112.1(8), WP_000602135.1(8), WP_000660476.1(8), WP_000840472.1(8), WP_000880199.1(8), WP_000891685.1(8), WP_000909596.1(8), WP_000917888.1(8), WP_000939395.1(8), WP_000940120.1(8), WP_000950177.1(8), WP_000980709.1(8), WP_000981378.1(8), WP_000996018.1(8), WP_001028618.1(8), WP_001040664.1(8), WP_001045530.1(8), WP_001069743.1(8), WP_001090508.1(8), WP_001092355.1(8), WP_001105478.1(8), WP_001107685.1(8), WP_001114006.1(8), WP_001114025.1(8), WP_001235634.1(8), WP_001258685.1(8), WP_001260707.1(8), WP_001266511.1(8), WP_001279403.1(8), WP_001283581.1(8), WP_001294697.1(8), WP_001297411.1(8), WP_001298568.1(8), WP_001302822.1(8), WP_001304765.1(8), WP_001314100.1(8), WP_001327408.1(8), WP_001350670.1(8), WP_001553935.1(8), WP_038432715.1(8), WP_000029479.1(7), WP_000057991.1(7), WP_000078346.1(7), WP_000079114.1(7), WP_000084170.1(7), WP_000099451.1(7), WP_000103580.1(7), WP_000131234.1(7), WP_000153091.1(7), WP_000173630.1(7), WP_000188193.1(7), WP_000205800.1(7), WP_000205840.1(7), WP_000213311.1(7), WP_000235240.1(7), WP_000387395.1(7), WP_000422217.1(7), WP_000447332.1(7), WP_000521562.1(7), WP_000569361.1(7), WP_000631388.1(7), WP_000654453.1(7), WP_000708507.1(7), WP_000710783.1(7), WP_000775238.1(7), WP_000813189.1(7), WP_000891665.1(7), WP_000917878.1(7), WP_000938499.1(7), WP_000939358.1(7), WP_000939394.1(7), WP_000981380.1(7), WP_001040630.1(7), WP_001061445.1(7), WP_001069724.1(7), WP_001070187.1(7), WP_001070190.1(7), WP_001084096.1(7), WP_001086517.1(7), WP_001086540.1(7), WP_001086546.1(7), WP_001090348.1(7), WP_001097116.1(7), WP_001126784.1(7), WP_001149734.1(7), WP_001193403.1(7), WP_001196496.1(7), WP_001223166.1(7), WP_001232912.1(7), WP_001232946.1(7), WP_001285553.1(7), WP_001287151.1(7), WP_001287164.1(7), WP_001293623.1(7), WP_001299561.1(7), WP_001304492.1(7), WP_001305041.1(7), WP_001305183.1(7), WP_001328413.1(7), WP_001348769.1(7), WP_001387129.1(7), WP_001428124.1(7), WP_000004425.1(6), WP_000047196.1(6), WP_000053086.1(6), WP_000057992.1(6), WP_000076277.1(6), WP_000076344.1(6), WP_000083666.1(6), WP_000085051.1(6), WP_000085060.1(6), WP_000096851.1(6), WP_000096881.1(6), WP_000099498.1(6), WP_000107024.1(6), WP_000124703.1(6), WP_000132546.1(6), WP_000138034.1(6), WP_000149094.1(6), WP_000176513.1(6), WP_000176573.1(6), WP_000184924.1(6), WP_000194892.1(6), WP_000204318.1(6), WP_000205822.1(6), WP_000205825.1(6), WP_000206432.1(6), WP_000219196.1(6), WP_000230938.1(6), WP_000240084.1(6), WP_000255020.1(6), WP_000281399.1(6), WP_000281446.1(6), WP_000281455.1(6), WP_000357764.1(6), WP_000416366.1(6), WP_000416368.1(6), WP_000431541.1(6), WP_000493455.1(6), WP_000493487.1(6), WP_000502506.1(6), WP_000525169.1(6), WP_000568929.1(6), WP_000569381.1(6), WP_000569424.1(6), WP_000582430.1(6), WP_000586456.1(6), WP_000590251.1(6), WP_000611419.1(6), WP_000633913.1(6), WP_000653934.1(6), WP_000678402.1(6), WP_000678417.1(6), WP_000678420.1(6), WP_000678431.1(6), WP_000678443.1(6), WP_000744773.1(6), WP_000775939.1(6), WP_000790159.1(6), WP_000829302.1(6), WP_000835430.1(6), WP_000839224.1(6), WP_000846350.1(6), WP_000872525.1(6), WP_000891675.1(6), WP_000907798.1(6), WP_000907807.1(6), WP_000907826.1(6), WP_000909595.1(6), WP_000911324.1(6), WP_000916321.1(6), WP_000934299.1(6), WP_000937931.1(6), WP_000937949.1(6), WP_000938570.1(6), WP_000939392.1(6), WP_000944244.1(6), WP_000948878.1(6), WP_000950200.1(6), WP_000961457.1(6), WP_000980688.1(6), WP_000980738.1(6), WP_000995683.1(6), WP_000996090.1(6), WP_000997418.1(6), WP_001043619.1(6), WP_001065373.1(6), WP_001069772.1(6), WP_001070193.1(6), WP_001086527.1(6), WP_001086549.1(6), WP_001105470.1(6), WP_001107659.1(6), WP_001107688.1(6), WP_001110578.1(6), WP_001111200.1(6), WP_001114028.1(6), WP_001114031.1(6), WP_001114035.1(6), WP_001114542.1(6), WP_001135739.1(6), WP_001136233.1(6), WP_001149714.1(6), WP_001149756.1(6), WP_001158933.1(6), WP_001194894.1(6), WP_001202164.1(6), WP_001202198.1(6), WP_001202230.1(6), WP_001232925.1(6), WP_001232949.1(6), WP_001238238.1(6), WP_001252647.1(6), WP_001266507.1(6), WP_001280357.1(6), WP_001285527.1(6), WP_001286556.1(6), WP_001294047.1(6), WP_001294687.1(6), WP_001294736.1(6), WP_001299190.1(6), WP_001300883.1(6), WP_001302075.1(6), WP_001302911.1(6), WP_001304311.1(6), WP_001304614.1(6), WP_001304780.1(6), WP_001304787.1(6), WP_001304988.1(6), WP_001305196.1(6), WP_001309661.1(6), WP_001314343.1(6), WP_001315369.1(6), WP_001319024.1(6), WP_001323996.1(6), WP_001332444.1(6), WP_001341191.1(6), WP_001346123.1(6), WP_001353282.1(6), WP_001386881.1(6), WP_001411916.1(6), WP_001443120.1(6), WP_001443176.1(6), WP_001529383.1(6), WP_001555859.1(6), WP_001704100.1(6), WP_001704191.1(6), WP_011076177.1(6), WP_032153941.1(6), WP_038432560.1(6), WP_046377545.1(6), WP_000007102.1(5), WP_000021017.1(5), WP_000021860.1(5), WP_000027201.1(5), WP_000042538.1(5), WP_000057099.1(5), WP_000057978.1(5), WP_000081419.1(5), WP_000085069.1(5), WP_000089718.1(5), WP_000096824.1(5), WP_000096875.1(5), WP_000096894.1(5), WP_000123720.1(5), WP_000123740.1(5), WP_000123745.1(5), WP_000123770.1(5), WP_000125643.1(5), WP_000131789.1(5), WP_000132903.1(5), WP_000149085.1(5), WP_000149107.1(5), WP_000149110.1(5), WP_000153100.1(5), WP_000163444.1(5), WP_000163448.1(5), WP_000187009.1(5), WP_000187020.1(5), WP_000188129.1(5), WP_000193523.1(5), WP_000194928.1(5), WP_000202564.1(5), WP_000204342.1(5), WP_000204344.1(5), WP_000205793.1(5), WP_000205794.1(5), WP_000206223.1(5), WP_000206272.1(5), WP_000206443.1(5), WP_000220399.1(5), WP_000237741.1(5), WP_000245530.1(5), WP_000250014.1(5), WP_000250021.1(5), WP_000255016.1(5), WP_000255019.1(5), WP_000255042.1(5), WP_000256575.1(5), WP_000281434.1(5), WP_000281442.1(5), WP_000286496.1(5), WP_000340183.1(5), WP_000387756.1(5), WP_000422224.1(5), WP_000422236.1(5), WP_000431373.1(5), WP_000438157.1(5), WP_000450520.1(5), WP_000484983.1(5), WP_000493461.1(5), WP_000493473.1(5), WP_000505729.1(5), WP_000528862.1(5), WP_000551266.1(5), WP_000568949.1(5), WP_000568956.1(5), WP_000569326.1(5), WP_000569367.1(5), WP_000569401.1(5), WP_000586488.1(5), WP_000593907.1(5), WP_000627732.1(5), WP_000635534.1(5), WP_000668665.1(5), WP_000668682.1(5), WP_000672322.1(5), WP_000678439.1(5), WP_000695653.1(5), WP_000708501.1(5), WP_000739048.1(5), WP_000739054.1(5), WP_000809170.1(5), WP_000813201.1(5), WP_000813259.1(5), WP_000819019.1(5), WP_000827382.1(5), WP_000827409.1(5), WP_000829332.1(5), WP_000839225.1(5), WP_000840464.1(5), WP_000860245.1(5), WP_000860266.1(5), WP_000860296.1(5), WP_000860301.1(5), WP_000880161.1(5), WP_000880175.1(5), WP_000880176.1(5), WP_000880193.1(5), WP_000891660.1(5), WP_000891676.1(5), WP_000916280.1(5), WP_000932346.1(5), WP_000932351.1(5), WP_000937920.1(5), WP_000938651.1(5), WP_000938658.1(5), WP_000938660.1(5), WP_000939350.1(5), WP_000939364.1(5), WP_000940868.1(5), WP_000940870.1(5), WP_000950184.1(5), WP_000956088.1(5), WP_000980724.1(5), WP_000983013.1(5), WP_000983022.1(5), WP_000994888.1(5), WP_000996031.1(5), WP_000997423.1(5), WP_001025308.1(5), WP_001025327.1(5), WP_001031058.1(5), WP_001039800.1(5), WP_001043601.1(5), WP_001043637.1(5), WP_001057125.1(5), WP_001065361.1(5), WP_001084084.1(5), WP_001086548.1(5), WP_001105505.1(5), WP_001107169.1(5), WP_001114019.1(5), WP_001117840.1(5), WP_001136211.1(5), WP_001140641.1(5), WP_001140680.1(5), WP_001144192.1(5), WP_001150468.1(5), WP_001157422.1(5), WP_001182410.1(5), WP_001189118.1(5), WP_001189123.1(5), WP_001193412.1(5), WP_001194882.1(5), WP_001202197.1(5), WP_001235592.1(5), WP_001235599.1(5), WP_001238226.1(5), WP_001238237.1(5), WP_001256179.1(5), WP_001258667.1(5), WP_001261288.1(5), WP_001264866.1(5), WP_001264868.1(5), WP_001279404.1(5), WP_001282282.1(5), WP_001282344.1(5), WP_001285559.1(5), WP_001286555.1(5), WP_001286581.1(5), WP_001286865.1(5), WP_001295087.1(5), WP_001304460.1(5), WP_001306215.1(5), WP_001313971.1(5), WP_001315707.1(5), WP_001324365.1(5), WP_001324480.1(5), WP_001342536.1(5), WP_001349941.1(5), WP_001349953.1(5), WP_001349994.1(5), WP_001356308.1(5), WP_001386827.1(5), WP_001386872.1(5), WP_001400571.1(5), WP_001405519.1(5), WP_001443105.1(5), WP_001443233.1(5), WP_001529442.1(5), WP_001531929.1(5), WP_001555790.1(5), WP_001564525.1(5), WP_012779395.1(5), WP_014640573.1(5), WP_024190736.1(5), WP_038432506.1(5), WP_038432512.1(5), WP_038432516.1(5), WP_038432518.1(5), WP_060667209.1(5), WP_000004448.1(4), WP_000007108.1(4), WP_000018421.1(4), WP_000025034.1(4), WP_000029335.1(4), WP_000029444.1(4), WP_000041957.1(4), WP_000046743.1(4), WP_000046800.1(4), WP_000046821.1(4), WP_000047179.1(4), WP_000047490.1(4), WP_000047502.1(4), WP_000053069.1(4), WP_000053100.1(4), WP_000057023.1(4), WP_000057096.1(4), WP_000057979.1(4), WP_000064446.1(4), WP_000066482.1(4), WP_000068006.1(4), WP_000068007.1(4), WP_000078322.1(4), WP_000078332.1(4), WP_000078340.1(4), WP_000078348.1(4), WP_000078908.1(4), WP_000079096.1(4), WP_000079110.1(4), WP_000082096.1(4), WP_000085068.1(4), WP_000089694.1(4), WP_000089714.1(4), WP_000099448.1(4), WP_000099493.1(4), WP_000103572.1(4), WP_000103574.1(4), WP_000107026.1(4), WP_000123718.1(4), WP_000123735.1(4), WP_000123738.1(4), WP_000123790.1(4), WP_000128864.1(4), WP_000128875.1(4), WP_000138832.1(4), WP_000139310.1(4), WP_000139525.1(4), WP_000139587.1(4), WP_000140645.1(4), WP_000147775.1(4), WP_000149086.1(4), WP_000149174.1(4), WP_000153108.1(4), WP_000173322.1(4), WP_000173674.1(4), WP_000173682.1(4), WP_000176526.1(4), WP_000181296.1(4), WP_000188188.1(4), WP_000194867.1(4), WP_000194883.1(4), WP_000204324.1(4), WP_000205806.1(4), WP_000206240.1(4), WP_000206281.1(4), WP_000206435.1(4), WP_000216180.1(4), WP_000220414.1(4), WP_000233439.1(4), WP_000237584.1(4), WP_000250015.1(4), WP_000250055.1(4), WP_000254747.1(4), WP_000256574.1(4), WP_000264781.1(4), WP_000271619.1(4), WP_000286503.1(4), WP_000357745.1(4), WP_000373318.1(4), WP_000376543.1(4), WP_000383109.1(4), WP_000383117.1(4), WP_000416381.1(4), WP_000416900.1(4), WP_000419018.1(4), WP_000422197.1(4), WP_000422218.1(4), WP_000433160.1(4), WP_000447323.1(4), WP_000447338.1(4), WP_000456448.1(4), WP_000456568.1(4), WP_000504857.1(4), WP_000551273.1(4), WP_000555862.1(4), WP_000569357.1(4), WP_000569400.1(4), WP_000573411.1(4), WP_000582418.1(4), WP_000582482.1(4), WP_000586440.1(4), WP_000590239.1(4), WP_000593954.1(4), WP_000593991.1(4), WP_000602116.1(4), WP_000621743.1(4), WP_000628542.1(4), WP_000628585.1(4), WP_000634808.1(4), WP_000653953.1(4), WP_000654456.1(4), WP_000660484.1(4), WP_000668703.1(4), WP_000672367.1(4), WP_000673573.1(4), WP_000678409.1(4), WP_000678480.1(4), WP_000686652.1(4), WP_000695641.1(4), WP_000708466.1(4), WP_000733252.1(4), WP_000734312.1(4), WP_000739056.1(4), WP_000768220.1(4), WP_000775932.1(4), WP_000775958.1(4), WP_000809172.1(4), WP_000813202.1(4), WP_000819002.1(4), WP_000819015.1(4), WP_000819026.1(4), WP_000827356.1(4), WP_000827407.1(4), WP_000829323.1(4), WP_000829368.1(4), WP_000844431.1(4), WP_000857409.1(4), WP_000860316.1(4), WP_000880178.1(4), WP_000886680.1(4), WP_000887482.1(4), WP_000903990.1(4), WP_000903999.1(4), WP_000907801.1(4), WP_000913116.1(4), WP_000916272.1(4), WP_000916324.1(4), WP_000932863.1(4), WP_000936355.1(4), WP_000937875.1(4), WP_000937930.1(4), WP_000938584.1(4), WP_000938596.1(4), WP_000940013.1(4), WP_000944243.1(4), WP_000944252.1(4), WP_000947148.1(4), WP_000948908.1(4), WP_000948914.1(4), WP_000956041.1(4), WP_000956045.1(4), WP_000956470.1(4), WP_000956884.1(4), WP_000961444.1(4), WP_000972085.1(4), WP_000980687.1(4), WP_000980735.1(4), WP_000983701.1(4), WP_000983706.1(4), WP_000985483.1(4), WP_000996006.1(4), WP_000996099.1(4), WP_001005919.1(4), WP_001017126.1(4), WP_001022014.1(4), WP_001025342.1(4), WP_001028621.1(4), WP_001033690.1(4), WP_001040175.1(4), WP_001040635.1(4), WP_001040647.1(4), WP_001040673.1(4), WP_001043638.1(4), WP_001045504.1(4), WP_001050399.1(4), WP_001050400.1(4), WP_001061444.1(4), WP_001069680.1(4), WP_001070168.1(4), WP_001072669.1(4), WP_001075516.1(4), WP_001084093.1(4), WP_001086520.1(4), WP_001086531.1(4), WP_001089398.1(4), WP_001090340.1(4), WP_001090484.1(4), WP_001090499.1(4), WP_001105466.1(4), WP_001105483.1(4), WP_001114033.1(4), WP_001117012.1(4), WP_001126787.1(4), WP_001136214.1(4), WP_001136234.1(4), WP_001138623.1(4), WP_001144206.1(4), WP_001144208.1(4), WP_001146501.1(4), WP_001149701.1(4), WP_001150436.1(4), WP_001150449.1(4), WP_001151867.1(4), WP_001193432.1(4), WP_001194868.1(4), WP_001194875.1(4), WP_001194884.1(4), WP_001194889.1(4), WP_001196487.1(4), WP_001198398.1(4), WP_001202214.1(4), WP_001202226.1(4), WP_001218666.1(4), WP_001223137.1(4), WP_001223151.1(4), WP_001223154.1(4), WP_001223165.1(4), WP_001223186.1(4), WP_001223213.1(4), WP_001232936.1(4), WP_001232938.1(4), WP_001235605.1(4), WP_001235609.1(4), WP_001235637.1(4), WP_001238207.1(4), WP_001238232.1(4), WP_001248693.1(4), WP_001248697.1(4), WP_001256180.1(4), WP_001256207.1(4), WP_001258269.1(4), WP_001258668.1(4), WP_001258683.1(4), WP_001272887.1(4), WP_001272906.1(4), WP_001272980.1(4), WP_001273008.1(4), WP_001285293.1(4), WP_001285531.1(4), WP_001285538.1(4), WP_001286585.1(4), WP_001286856.1(4), WP_001286887.1(4), WP_001291215.1(4), WP_001291786.1(4), WP_001293604.1(4), WP_001293624.1(4), WP_001294681.1(4), WP_001297565.1(4), WP_001300388.1(4), WP_001306170.1(4), WP_001306372.1(4), WP_001311372.1(4), WP_001315223.1(4), WP_001323790.1(4), WP_001327263.1(4), WP_001330751.1(4), WP_001331920.1(4), WP_001332064.1(4), WP_001338236.1(4), WP_001345234.1(4), WP_001349913.1(4), WP_001350064.1(4), WP_001356203.1(4), WP_001356261.1(4), WP_001362465.1(4), WP_001376672.1(4), WP_001386588.1(4), WP_001387013.1(4), WP_001392130.1(4), WP_001398823.1(4), WP_001400452.1(4), WP_001400473.1(4), WP_001400514.1(4), WP_001400694.1(4), WP_001401248.1(4), WP_001514524.1(4), WP_001545683.1(4), WP_001545994.1(4), WP_001546092.1(4), WP_001573877.1(4), WP_001578024.1(4), WP_001612582.1(4), WP_001613510.1(4), WP_022581749.1(4), WP_022581764.1(4), WP_022646037.1(4), WP_024177878.1(4), WP_024179124.1(4), WP_024199857.1(4), WP_024221588.1(4), WP_024221621.1(4), WP_024221633.1(4), WP_024221715.1(4), WP_024221749.1(4), WP_024221797.1(4), WP_032143004.1(4), WP_032143359.1(4), WP_032161953.1(4), WP_038432468.1(4), WP_038432470.1(4), WP_048967001.1(4), WP_000002088.1(3), WP_000003805.1(3), WP_000004438.1(3), WP_000007109.1(3), WP_000021031.1(3), WP_000027208.1(3), WP_000029443.1(3), WP_000040320.1(3), WP_000041980.1(3), WP_000046786.1(3), WP_000046838.1(3), WP_000047508.1(3), WP_000047538.1(3), WP_000053122.1(3), WP_000057068.1(3), WP_000057972.1(3), WP_000058403.1(3), WP_000066486.1(3), WP_000078831.1(3), WP_000079108.1(3), WP_000099507.1(3), WP_000107033.1(3), WP_000110931.1(3), WP_000123739.1(3), WP_000138037.1(3), WP_000139323.1(3), WP_000139330.1(3), WP_000139359.1(3), WP_000140646.1(3), WP_000149120.1(3), WP_000162569.1(3), WP_000164228.1(3), WP_000173690.1(3), WP_000175949.1(3), WP_000176556.1(3), WP_000176567.1(3), WP_000186638.1(3), WP_000187046.1(3), WP_000188140.1(3), WP_000188173.1(3), WP_000193508.1(3), WP_000193522.1(3), WP_000202566.1(3), WP_000202987.1(3), WP_000206431.1(3), WP_000206450.1(3), WP_000220417.1(3), WP_000230289.1(3), WP_000233443.1(3), WP_000245528.1(3), WP_000256573.1(3), WP_000271620.1(3), WP_000281395.1(3), WP_000415585.1(3), WP_000416420.1(3), WP_000422175.1(3), WP_000462760.1(3), WP_000484979.1(3), WP_000484982.1(3), WP_000484985.1(3), WP_000485008.1(3), WP_000493464.1(3), WP_000493495.1(3), WP_000493503.1(3), WP_000517697.1(3), WP_000525173.1(3), WP_000525182.1(3), WP_000568921.1(3), WP_000569327.1(3), WP_000569356.1(3), WP_000569358.1(3), WP_000569440.1(3), WP_000578083.1(3), WP_000582434.1(3), WP_000582470.1(3), WP_000582495.1(3), WP_000586495.1(3), WP_000593887.1(3), WP_000593906.1(3), WP_000593960.1(3), WP_000594008.1(3), WP_000602134.1(3), WP_000611429.1(3), WP_000622315.1(3), WP_000627728.1(3), WP_000628552.1(3), WP_000628569.1(3), WP_000672342.1(3), WP_000672417.1(3), WP_000678436.1(3), WP_000678446.1(3), WP_000708472.1(3), WP_000708482.1(3), WP_000708490.1(3), WP_000719979.1(3), WP_000725347.1(3), WP_000741723.1(3), WP_000752797.1(3), WP_000809047.1(3), WP_000818112.1(3), WP_000827383.1(3), WP_000827396.1(3), WP_000829398.1(3), WP_000857401.1(3), WP_000860253.1(3), WP_000877024.1(3), WP_000891696.1(3), WP_000902693.1(3), WP_000904003.1(3), WP_000911313.1(3), WP_000912355.1(3), WP_000913119.1(3), WP_000917867.1(3), WP_000935258.1(3), WP_000936285.1(3), WP_000936365.1(3), WP_000938609.1(3), WP_000939339.1(3), WP_000940887.1(3), WP_000944258.1(3), WP_000948894.1(3), WP_000956467.1(3), WP_000956878.1(3), WP_000980701.1(3), WP_000980764.1(3), WP_000983714.1(3), WP_000996002.1(3), WP_001025336.1(3), WP_001040656.1(3), WP_001040683.1(3), WP_001061380.1(3), WP_001084104.1(3), WP_001089390.1(3), WP_001096688.1(3), WP_001105482.1(3), WP_001110563.1(3), WP_001114037.1(3), WP_001114529.1(3), WP_001117010.1(3), WP_001135714.1(3), WP_001136198.1(3), WP_001146482.1(3), WP_001149740.1(3), WP_001150467.1(3), WP_001151863.1(3), WP_001157377.1(3), WP_001189126.1(3), WP_001194902.1(3), WP_001198381.1(3), WP_001202175.1(3), WP_001202192.1(3), WP_001216677.1(3), WP_001223149.1(3), WP_001223164.1(3), WP_001223206.1(3), WP_001232915.1(3), WP_001234854.1(3), WP_001235585.1(3), WP_001235608.1(3), WP_001235622.1(3), WP_001238248.1(3), WP_001286547.1(3), WP_001286566.1(3), WP_001286873.1(3), WP_001286892.1(3), WP_001293607.1(3), WP_001297215.1(3), WP_001297425.1(3), WP_001298972.1(3), WP_001299221.1(3), WP_001299509.1(3), WP_001299842.1(3), WP_001300406.1(3), WP_001303307.1(3), WP_001310731.1(3), WP_001313475.1(3), WP_001317990.1(3), WP_001323973.1(3), WP_001328193.1(3), WP_001329358.1(3), WP_001329571.1(3), WP_001338165.1(3), WP_001338472.1(3), WP_001351371.1(3), WP_001351532.1(3), WP_001351758.1(3), WP_001361293.1(3), WP_001362989.1(3), WP_001376535.1(3), WP_001401476.1(3), WP_001434589.1(3), WP_001531291.1(3), WP_001531295.1(3), WP_001543447.1(3), WP_001543532.1(3), WP_001543542.1(3), WP_001543545.1(3), WP_001543583.1(3), WP_001543592.1(3), WP_001543600.1(3), WP_001543702.1(3), WP_001543724.1(3), WP_001543805.1(3), WP_001544044.1(3), WP_001544053.1(3), WP_001544120.1(3), WP_001544144.1(3), WP_001544193.1(3), WP_001544214.1(3), WP_001544736.1(3), WP_001545714.1(3), WP_001545815.1(3), WP_001546035.1(3), WP_001546063.1(3), WP_001550407.1(3), WP_001550833.1(3), WP_001551736.1(3), WP_001551739.1(3), WP_001554269.1(3), WP_001564234.1(3), WP_001577603.1(3), WP_001577744.1(3), WP_001578028.1(3), WP_001578206.1(3), WP_001579065.1(3), WP_001586717.1(3), WP_001589603.1(3), WP_001589606.1(3), WP_001590975.1(3), WP_001595743.1(3), WP_001595747.1(3), WP_001599773.1(3), WP_001599848.1(3), WP_001600159.1(3), WP_001600422.1(3), WP_001600636.1(3), WP_001600939.1(3), WP_001672356.1(3), WP_004015950.1(3), WP_012135848.1(3), WP_015364499.1(3), WP_015674614.1(3), WP_020239280.1(3), WP_020239602.1(3), WP_020239776.1(3), WP_020239916.1(3), WP_021551967.1(3), WP_021570170.1(3), WP_021570173.1(3), WP_021570214.1(3), WP_024173638.1(3), WP_024174459.1(3), WP_024181845.1(3), WP_024189843.1(3), WP_024190590.1(3), WP_024194587.1(3), WP_024195938.1(3), WP_024229461.1(3), WP_024230162.1(3), WP_024241213.1(3), WP_025210100.1(3), WP_025210116.1(3), WP_025210125.1(3), WP_025210127.1(3), WP_025210129.1(3), WP_025210130.1(3), WP_025210177.1(3), WP_025210179.1(3), WP_025210182.1(3), WP_025210185.1(3), WP_025210204.1(3), WP_025210215.1(3), WP_025210245.1(3), WP_025210249.1(3), WP_025210253.1(3), WP_025210254.1(3), WP_025210260.1(3), WP_025210306.1(3), WP_025210318.1(3), WP_025210326.1(3), WP_025210331.1(3), WP_025210346.1(3), WP_025210347.1(3), WP_025210383.1(3), WP_025210394.1(3), WP_025210403.1(3), WP_025210407.1(3), WP_025210409.1(3), WP_025210416.1(3), WP_025210457.1(3), WP_025210489.1(3), WP_025210491.1(3), WP_025210500.1(3), WP_025210536.1(3), WP_025210546.1(3), WP_025210547.1(3), WP_025210554.1(3), WP_025210565.1(3), WP_025210606.1(3), WP_025210670.1(3), WP_025210674.1(3), WP_025210688.1(3), WP_025210690.1(3), WP_025210702.1(3), WP_025210709.1(3), WP_025210766.1(3), WP_025210768.1(3), WP_025210770.1(3), WP_025210771.1(3), WP_025210784.1(3), WP_025210795.1(3), WP_025210797.1(3), WP_025210798.1(3), WP_025210799.1(3), WP_025210808.1(3), WP_025210849.1(3), WP_025210904.1(3), WP_025210924.1(3), WP_025210932.1(3), WP_025210935.1(3), WP_025210936.1(3), WP_025210940.1(3), WP_025210944.1(3), WP_025210964.1(3), WP_025210965.1(3), WP_025210969.1(3), WP_025210992.1(3), WP_025211012.1(3), WP_025269752.1(3), WP_025269842.1(3), WP_025269874.1(3), WP_025269875.1(3), WP_025269886.1(3), WP_025269905.1(3), WP_025269923.1(3), WP_025270007.1(3), WP_025270032.1(3), WP_032141179.1(3), WP_032145144.1(3), WP_038428526.1(3), WP_038428572.1(3), WP_042336790.1(3), WP_048889350.1(3), WP_000004477.1(2), WP_000018742.1(2), WP_000020503.1(2), WP_000021025.1(2), WP_000023637.1(2), WP_000028875.1(2), WP_000029324.1(2), WP_000030915.1(2), WP_000035432.1(2), WP_000041966.1(2), WP_000042535.1(2), WP_000046818.1(2), WP_000047180.1(2), WP_000047503.1(2), WP_000057024.1(2), WP_000057955.1(2), WP_000064440.1(2), WP_000066485.1(2), WP_000072434.1(2), WP_000076300.1(2), WP_000076310.1(2), WP_000076339.1(2), WP_000076343.1(2), WP_000078832.1(2), WP_000078853.1(2), WP_000079097.1(2), WP_000079099.1(2), WP_000082098.1(2), WP_000087755.1(2), WP_000090774.1(2), WP_000096866.1(2), WP_000099519.1(2), WP_000107030.1(2), WP_000126279.1(2), WP_000128514.1(2), WP_000131756.1(2), WP_000131776.1(2), WP_000132545.1(2), WP_000132614.1(2), WP_000132621.1(2), WP_000132907.1(2), WP_000138042.1(2), WP_000138281.1(2), WP_000139315.1(2), WP_000139319.1(2), WP_000139371.1(2), WP_000139569.1(2), WP_000139570.1(2), WP_000139593.1(2), WP_000149154.1(2), WP_000149158.1(2), WP_000152931.1(2), WP_000153102.1(2), WP_000155032.1(2), WP_000163446.1(2), WP_000163452.1(2), WP_000163456.1(2), WP_000163459.1(2), WP_000165542.1(2), WP_000166948.1(2), WP_000166952.1(2), WP_000173318.1(2), WP_000173663.1(2), WP_000173694.1(2), WP_000175452.1(2), WP_000176547.1(2), WP_000176551.1(2), WP_000176577.1(2), WP_000187016.1(2), WP_000187018.1(2), WP_000187019.1(2), WP_000187529.1(2), WP_000188182.1(2), WP_000193507.1(2), WP_000193528.1(2), WP_000193537.1(2), WP_000193558.1(2), WP_000193566.1(2), WP_000193569.1(2), WP_000194876.1(2), WP_000194898.1(2), WP_000204339.1(2), WP_000205784.1(2), WP_000205797.1(2), WP_000205816.1(2), WP_000206247.1(2), WP_000206271.1(2), WP_000206440.1(2), WP_000213291.1(2), WP_000213292.1(2), WP_000213318.1(2), WP_000220401.1(2), WP_000220416.1(2), WP_000230262.1(2), WP_000230282.1(2), WP_000235248.1(2), WP_000235260.1(2), WP_000239727.1(2), WP_000245522.1(2), WP_000245534.1(2), WP_000250010.1(2), WP_000255024.1(2), WP_000258199.1(2), WP_000281461.1(2), WP_000286519.1(2), WP_000340003.1(2), WP_000340008.1(2), WP_000340011.1(2), WP_000350049.1(2), WP_000376545.1(2), WP_000383107.1(2), WP_000387767.1(2), WP_000392435.1(2), WP_000410810.1(2), WP_000416390.1(2), WP_000416399.1(2), WP_000416624.1(2), WP_000417651.1(2), WP_000422166.1(2), WP_000422230.1(2), WP_000433154.1(2), WP_000433156.1(2), WP_000433876.1(2), WP_000438163.1(2), WP_000438165.1(2), WP_000456461.1(2), WP_000460032.1(2), WP_000466147.1(2), WP_000484987.1(2), WP_000485001.1(2), WP_000485005.1(2), WP_000493463.1(2), WP_000493510.1(2), WP_000499789.1(2), WP_000517687.1(2), WP_000517694.1(2), WP_000517695.1(2), WP_000517696.1(2), WP_000517886.1(2), WP_000521603.1(2), WP_000528863.1(2), WP_000528870.1(2), WP_000529946.1(2), WP_000532148.1(2), WP_000543083.1(2), WP_000551264.1(2), WP_000568931.1(2), WP_000569325.1(2), WP_000569329.1(2), WP_000569343.1(2), WP_000570666.1(2), WP_000578099.1(2), WP_000581938.1(2), WP_000581939.1(2), WP_000582454.1(2), WP_000582458.1(2), WP_000582466.1(2), WP_000582492.1(2), WP_000582832.1(2), WP_000586485.1(2), WP_000590241.1(2), WP_000590253.1(2), WP_000590263.1(2), WP_000593905.1(2), WP_000593916.1(2), WP_000593945.1(2), WP_000594001.1(2), WP_000594009.1(2), WP_000602099.1(2), WP_000611387.1(2), WP_000611436.1(2), WP_000627733.1(2), WP_000628551.1(2), WP_000628584.1(2), WP_000634793.1(2), WP_000634815.1(2), WP_000659976.1(2), WP_000662472.1(2), WP_000667324.1(2), WP_000668698.1(2), WP_000671691.1(2), WP_000672314.1(2), WP_000672382.1(2), WP_000673549.1(2), WP_000678399.1(2), WP_000678418.1(2), WP_000678440.1(2), WP_000695657.1(2), WP_000695664.1(2), WP_000708470.1(2), WP_000708495.1(2), WP_000733250.1(2), WP_000744775.1(2), WP_000801440.1(2), WP_000807561.1(2), WP_000809052.1(2), WP_000809065.1(2), WP_000813197.1(2), WP_000818118.1(2), WP_000819006.1(2), WP_000827377.1(2), WP_000827388.1(2), WP_000827481.1(2), WP_000829335.1(2), WP_000846329.1(2), WP_000846337.1(2), WP_000846351.1(2), WP_000880156.1(2), WP_000880192.1(2), WP_000880194.1(2), WP_000882661.1(2), WP_000887486.1(2), WP_000903991.1(2), WP_000907785.1(2), WP_000911317.1(2), WP_000911331.1(2), WP_000912342.1(2), WP_000912347.1(2), WP_000916296.1(2), WP_000918819.1(2), WP_000918822.1(2), WP_000918826.1(2), WP_000923072.1(2), WP_000932345.1(2), WP_000932838.1(2), WP_000932864.1(2), WP_000936367.1(2), WP_000936372.1(2), WP_000937895.1(2), WP_000937904.1(2), WP_000937909.1(2), WP_000937964.1(2), WP_000938600.1(2), WP_000938615.1(2), WP_000938620.1(2), WP_000938649.1(2), WP_000939347.1(2), WP_000939373.1(2), WP_000947155.1(2), WP_000947398.1(2), WP_000948877.1(2), WP_000950174.1(2), WP_000950187.1(2), WP_000950193.1(2), WP_000956032.1(2), WP_000956076.1(2), WP_000956877.1(2), WP_000956890.1(2), WP_000956898.1(2), WP_000961453.1(2), WP_000967410.1(2), WP_000980686.1(2), WP_000980690.1(2), WP_000980697.1(2), WP_000980700.1(2), WP_000983008.1(2), WP_000985488.1(2), WP_000994912.1(2), WP_000996010.1(2), WP_000996017.1(2), WP_000997323.1(2), WP_001013626.1(2), WP_001013627.1(2), WP_001013632.1(2), WP_001013644.1(2), WP_001040639.1(2), WP_001040646.1(2), WP_001040651.1(2), WP_001041504.1(2), WP_001043590.1(2), WP_001043592.1(2), WP_001043621.1(2), WP_001043627.1(2), WP_001045509.1(2), WP_001050402.1(2), WP_001050404.1(2), WP_001057989.1(2), WP_001061417.1(2), WP_001061418.1(2), WP_001061427.1(2), WP_001061438.1(2), WP_001062679.1(2), WP_001069682.1(2), WP_001069713.1(2), WP_001069714.1(2), WP_001069757.1(2), WP_001069762.1(2), WP_001069798.1(2), WP_001069800.1(2), WP_001072672.1(2), WP_001076062.1(2), WP_001084075.1(2), WP_001084094.1(2), WP_001084112.1(2), WP_001086519.1(2), WP_001090345.1(2), WP_001090347.1(2), WP_001090495.1(2), WP_001090525.1(2), WP_001105463.1(2), WP_001105467.1(2), WP_001105474.1(2), WP_001105493.1(2), WP_001105518.1(2), WP_001107180.1(2), WP_001107651.1(2), WP_001107652.1(2), WP_001107655.1(2), WP_001107708.1(2), WP_001114545.1(2), WP_001117006.1(2), WP_001118931.1(2), WP_001135728.1(2), WP_001136228.1(2), WP_001136249.1(2), WP_001138625.1(2), WP_001146471.1(2), WP_001150434.1(2), WP_001151858.1(2), WP_001151859.1(2), WP_001157532.1(2), WP_001157538.1(2), WP_001182953.1(2), WP_001187813.1(2), WP_001187870.1(2), WP_001193398.1(2), WP_001194873.1(2), WP_001194881.1(2), WP_001194916.1(2), WP_001196481.1(2), WP_001198378.1(2), WP_001198454.1(2), WP_001202201.1(2), WP_001202228.1(2), WP_001218654.1(2), WP_001218669.1(2), WP_001232901.1(2), WP_001232965.1(2), WP_001232967.1(2), WP_001235636.1(2), WP_001238224.1(2), WP_001247087.1(2), WP_001247091.1(2), WP_001247094.1(2), WP_001247097.1(2), WP_001248694.1(2), WP_001256219.1(2), WP_001256222.1(2), WP_001258268.1(2), WP_001258690.1(2), WP_001260706.1(2), WP_001261274.1(2), WP_001263491.1(2), WP_001264877.1(2), WP_001266512.1(2), WP_001272907.1(2), WP_001272909.1(2), WP_001280349.1(2), WP_001283249.1(2), WP_001285525.1(2), WP_001285539.1(2), WP_001286559.1(2), WP_001286575.1(2), WP_001286881.1(2), WP_001286883.1(2), WP_001289625.1(2), WP_001291768.1(2), WP_001291797.1(2), WP_001294693.1(2), WP_001294704.1(2), WP_001296826.1(2), WP_001298231.1(2), WP_001298479.1(2), WP_001300645.1(2), WP_001304268.1(2), WP_001306022.1(2), WP_001310439.1(2), WP_001315242.1(2), WP_001317926.1(2), WP_001330437.1(2), WP_001331364.1(2), WP_001333396.1(2), WP_001335321.1(2), WP_001338359.1(2), WP_001341957.1(2), WP_001347925.1(2), WP_001350580.1(2), WP_001350678.1(2), WP_001350691.1(2), WP_001350820.1(2), WP_001351689.1(2), WP_001351945.1(2), WP_001352361.1(2), WP_001353637.1(2), WP_001359006.1(2), WP_001359011.1(2), WP_001359711.1(2), WP_001359792.1(2), WP_001360328.1(2), WP_001371044.1(2), WP_001376015.1(2), WP_001378857.1(2), WP_001387489.1(2), WP_001395096.1(2), WP_001395720.1(2), WP_001395971.1(2), WP_001400536.1(2), WP_001400627.1(2), WP_001401030.1(2), WP_001407301.1(2), WP_001417730.1(2), WP_001419087.1(2), WP_001443155.1(2), WP_001443235.1(2), WP_001446633.1(2), WP_001488516.1(2), WP_001522014.1(2), WP_001533743.1(2), WP_001538498.1(2), WP_001542893.1(2), WP_001543081.1(2), WP_001543365.1(2), WP_001553854.1(2), WP_001555993.1(2), WP_001556019.1(2), WP_001556163.1(2), WP_001559503.1(2), WP_001564045.1(2), WP_001567328.1(2), WP_001571626.1(2), WP_001572020.1(2), WP_001576302.1(2), WP_001576831.1(2), WP_001576840.1(2), WP_001576845.1(2), WP_001576877.1(2), WP_001597021.1(2), WP_001701073.1(2), WP_004187427.1(2), WP_004972005.1(2), WP_011579218.1(2), WP_012304832.1(2), WP_012311895.1(2), WP_012728230.1(2), WP_013009103.1(2), WP_013009107.1(2), WP_014639874.1(2), WP_014639938.1(2), WP_014640031.1(2), WP_014640045.1(2), WP_014640050.1(2), WP_015967850.1(2), WP_016243364.1(2), WP_016243931.1(2), WP_021522464.1(2), WP_022645167.1(2), WP_022645171.1(2), WP_022645197.1(2), WP_022645198.1(2), WP_022645200.1(2), WP_022645224.1(2), WP_022645225.1(2), WP_022645229.1(2), WP_022645265.1(2), WP_022645370.1(2), WP_022645376.1(2), WP_022645395.1(2), WP_022645396.1(2), WP_022645405.1(2), WP_022645444.1(2), WP_022645479.1(2), WP_022645480.1(2), WP_022645508.1(2), WP_022645526.1(2), WP_022645527.1(2), WP_022645539.1(2), WP_022645549.1(2), WP_022645584.1(2), WP_022645616.1(2), WP_022645642.1(2), WP_022645666.1(2), WP_022645683.1(2), WP_022645686.1(2), WP_022645705.1(2), WP_022645743.1(2), WP_022645752.1(2), WP_022645763.1(2), WP_022645764.1(2), WP_022645777.1(2), WP_022645796.1(2), WP_022645806.1(2), WP_022645809.1(2), WP_022645816.1(2), WP_022645872.1(2), WP_022645877.1(2), WP_022645887.1(2), WP_022645888.1(2), WP_022645909.1(2), WP_022645963.1(2), WP_022645973.1(2), WP_022645985.1(2), WP_022645994.1(2), WP_022645998.1(2), WP_022646031.1(2), WP_022646032.1(2), WP_022646038.1(2), WP_022646039.1(2), WP_022646054.1(2), WP_022646056.1(2), WP_022646057.1(2), WP_022646077.1(2), WP_022646174.1(2), WP_022646187.1(2), WP_022646214.1(2), WP_022646215.1(2), WP_022646221.1(2), WP_022646225.1(2), WP_022646228.1(2), WP_022646249.1(2), WP_022646256.1(2), WP_022646257.1(2), WP_022646268.1(2), WP_022646277.1(2), WP_022646280.1(2), WP_022646284.1(2), WP_022646298.1(2), WP_022646352.1(2), WP_022646377.1(2), WP_022646447.1(2), WP_022646456.1(2), WP_022646466.1(2), WP_023155300.1(2), WP_023908778.1(2), WP_023908880.1(2), WP_023908991.1(2), WP_023909166.1(2), WP_023909198.1(2), WP_024173951.1(2), WP_024179510.1(2), WP_024181813.1(2), WP_024181822.1(2), WP_024185627.1(2), WP_024186193.1(2), WP_024221576.1(2), WP_024221593.1(2), WP_024221753.1(2), WP_024229265.1(2), WP_024256148.1(2), WP_024946528.1(2), WP_025210341.1(2), WP_025210357.1(2), WP_025210388.1(2), WP_025210406.1(2), WP_025210579.1(2), WP_025210741.1(2), WP_025380230.1(2), WP_025380237.1(2), WP_025380257.1(2), WP_025380266.1(2), WP_025380284.1(2), WP_025380357.1(2), WP_025380379.1(2), WP_025380414.1(2), WP_025380503.1(2), WP_025380504.1(2), WP_025380563.1(2), WP_025380564.1(2), WP_025380601.1(2), WP_025380611.1(2), WP_025380646.1(2), WP_025380670.1(2), WP_025380671.1(2), WP_025380675.1(2), WP_025380699.1(2), WP_025404314.1(2), WP_025404343.1(2), WP_025404345.1(2), WP_025404440.1(2), WP_025404492.1(2), WP_025404505.1(2), WP_029594353.1(2), WP_032140208.1(2), WP_032140236.1(2), WP_032140360.1(2), WP_032142472.1(2), WP_032144923.1(2), WP_032156602.1(2), WP_032161154.1(2), WP_032162051.1(2), WP_032235444.1(2), WP_032245820.1(2), WP_032315666.1(2), WP_032316417.1(2), WP_033871920.1(2), WP_038432513.1(2), WP_038432731.1(2), WP_039023146.1(2), WP_039023147.1(2), WP_044519297.1(2), WP_044519660.1(2), WP_044519753.1(2), WP_044519780.1(2), WP_046201558.1(2), WP_048943202.1(2), WP_048943205.1(2), WP_048943256.1(2), WP_048943289.1(2), WP_048943309.1(2), WP_048943314.1(2), WP_048943372.1(2), WP_050457944.1(2), WP_050868838.1(2), WP_051394484.1(2), WP_051529555.1(2), WP_059219429.1(2), NP_052606.1(1), NP_052625.1(1), NP_052642.1(1), NP_052660.1(1), NP_052671.1(1), NP_308043.1(1), NP_308053.1(1), NP_308056.1(1), NP_308083.1(1), NP_308089.1(1), NP_308090.1(1), NP_308097.1(1), NP_308101.1(1), NP_308103.1(1), NP_308125.1(1), NP_308149.1(1), NP_308158.1(1), NP_308174.2(1), NP_308175.2(1), NP_308178.2(1), NP_308179.1(1), NP_308182.1(1), NP_308191.1(1), NP_308198.1(1), NP_308199.1(1), NP_308201.1(1), NP_308212.1(1), NP_308213.1(1), NP_308217.1(1), NP_308220.1(1), NP_308223.1(1), NP_308228.1(1), NP_308237.1(1), NP_308238.1(1), NP_308287.1(1), NP_308289.1(1), NP_308290.1(1), NP_308296.1(1), NP_308299.1(1), NP_308394.1(1), NP_308402.1(1), NP_308403.1(1), NP_308440.2(1), NP_308447.1(1), NP_308483.1(1), NP_308484.1(1), NP_308494.1(1), NP_308496.1(1), NP_308504.1(1), NP_308517.1(1), NP_308526.1(1), NP_308529.1(1), NP_308530.1(1), NP_308554.1(1), NP_308561.1(1), NP_308570.1(1), NP_308580.1(1), NP_308585.1(1), NP_308615.1(1), NP_308647.1(1), NP_308648.1(1), NP_308654.1(1), NP_308689.1(1), NP_308707.1(1), NP_308718.1(1), NP_308737.1(1), NP_308766.1(1), NP_308815.1(1), NP_308820.1(1), NP_308826.2(1), NP_308884.1(1), NP_308899.2(1), NP_308902.1(1), NP_308904.1(1), NP_308914.1(1), NP_308924.1(1), NP_308935.1(1), NP_308962.1(1), NP_308966.1(1), NP_308974.1(1), NP_308992.1(1), NP_308993.1(1), NP_308998.1(1), NP_308999.1(1), NP_309005.1(1), NP_309021.1(1), NP_309024.1(1), NP_309027.1(1), NP_309031.1(1), NP_309040.1(1), NP_309043.1(1), NP_309059.1(1), NP_309060.1(1), NP_309107.1(1), NP_309171.1(1), NP_309172.1(1), NP_309272.1(1), NP_309283.1(1), NP_309319.1(1), NP_309332.1(1), NP_309373.1(1), NP_309374.1(1), NP_309450.1(1), NP_309489.1(1), NP_309491.1(1), NP_309494.1(1), NP_309508.2(1), NP_309519.1(1), NP_309522.2(1), NP_309547.1(1), NP_309598.1(1), NP_309632.2(1), NP_309634.2(1), NP_309724.1(1), NP_309735.1(1), NP_309736.1(1), NP_309742.1(1), NP_309743.1(1), NP_309744.1(1), NP_309761.1(1), NP_309773.1(1), NP_309774.1(1), NP_309800.1(1), NP_309866.3(1), NP_309868.1(1), NP_309876.1(1), NP_309886.1(1), NP_309894.1(1), NP_309895.1(1), NP_309924.1(1), NP_309953.1(1), NP_309954.1(1), NP_309955.1(1), NP_310042.1(1), NP_310072.1(1), NP_310100.1(1), NP_310114.1(1), NP_310115.1(1), NP_310128.1(1), NP_310147.1(1), NP_310153.1(1), NP_310225.1(1), NP_310373.1(1), NP_310388.1(1), NP_310389.1(1), NP_310416.1(1), NP_310443.1(1), NP_310447.1(1), NP_310448.1(1), NP_310450.1(1), NP_310451.3(1), NP_310452.1(1), NP_310453.1(1), NP_310489.1(1), NP_310523.1(1), NP_310540.1(1), NP_310544.1(1), NP_310545.1(1), NP_310560.1(1), NP_310568.1(1), NP_310572.1(1), NP_310581.1(1), NP_310595.1(1), NP_310603.1(1), NP_310613.1(1), NP_310621.1(1), NP_310635.1(1), NP_310682.1(1), NP_310707.1(1), NP_310781.1(1), NP_310840.1(1), NP_310847.1(1), NP_310852.2(1), NP_310853.1(1), NP_310947.1(1), NP_311021.1(1), NP_311043.1(1), NP_311053.1(1), NP_311066.2(1), NP_311068.1(1), NP_311099.1(1), NP_311102.1(1), NP_311103.1(1), NP_311104.1(1), NP_311117.1(1), NP_311127.1(1), NP_311170.1(1), NP_311183.2(1), NP_311217.1(1), NP_311229.1(1), NP_311241.2(1), NP_311305.1(1), NP_311320.1(1), NP_311363.1(1), NP_311389.1(1), NP_311396.1(1), NP_311398.1(1), NP_311400.2(1), NP_311403.1(1), NP_311425.1(1), NP_311428.2(1), NP_311440.1(1), NP_311452.1(1), NP_311459.1(1), NP_311460.1(1), NP_311462.1(1), NP_311468.1(1), NP_311469.1(1), NP_311474.1(1), NP_311484.1(1), NP_311496.1(1), NP_311497.1(1), NP_311498.3(1), NP_311499.1(1), NP_311500.1(1), NP_311507.2(1), NP_311509.1(1), NP_311567.1(1), NP_311580.1(1), NP_311581.1(1), NP_311595.1(1), NP_311616.1(1), NP_311624.1(1), NP_311626.1(1), NP_311632.1(1), NP_311637.1(1), NP_311638.1(1), NP_311639.1(1), NP_311640.1(1), NP_311641.1(1), NP_311643.1(1), NP_311669.1(1), NP_311670.1(1), NP_311672.1(1), NP_311678.1(1), NP_311693.1(1), NP_311703.1(1), NP_311757.1(1), NP_311789.1(1), NP_311790.1(1), NP_311791.1(1), NP_311799.1(1), NP_311830.1(1), NP_311848.1(1), NP_311849.2(1), NP_311943.1(1), NP_311966.1(1), NP_311975.1(1), NP_311976.1(1), NP_311977.1(1), NP_311983.1(1), NP_312020.2(1), NP_312070.2(1), NP_312072.1(1), NP_312073.1(1), NP_312074.1(1), NP_312076.1(1), NP_312077.1(1), NP_312081.1(1), NP_312085.1(1), NP_312086.1(1), NP_312089.1(1), NP_312091.1(1), NP_312092.1(1), NP_312101.1(1), NP_312107.1(1), NP_312108.1(1), NP_312130.1(1), NP_312131.1(1), NP_312146.1(1), NP_312159.1(1), NP_312171.1(1), NP_312175.1(1), NP_312180.1(1), NP_312181.1(1), NP_312186.1(1), NP_312187.1(1), NP_312188.1(1), NP_312189.1(1), NP_312190.1(1), NP_312191.1(1), NP_312192.1(1), NP_312193.1(1), NP_312194.1(1), NP_312195.1(1), NP_312196.1(1), NP_312197.1(1), NP_312198.1(1), NP_312199.1(1), NP_312200.1(1), NP_312201.1(1), NP_312202.1(1), NP_312203.1(1), NP_312204.1(1), NP_312205.1(1), NP_312206.1(1), NP_312207.1(1), NP_312208.1(1), NP_312209.1(1), NP_312210.1(1), NP_312211.1(1), NP_312212.1(1), NP_312213.1(1), NP_312217.1(1), NP_312218.1(1), NP_312219.1(1), NP_312220.1(1), NP_312230.1(1), NP_312253.1(1), NP_312269.1(1), NP_312276.1(1), NP_312290.1(1), NP_312291.1(1), NP_312292.1(1), NP_312323.2(1), NP_312328.2(1), NP_312329.1(1), NP_312339.1(1), NP_312340.1(1), NP_312373.1(1), NP_312374.1(1), NP_312386.1(1), NP_312398.1(1), NP_312414.1(1), NP_312447.1(1), NP_312448.1(1), NP_312467.1(1), NP_312468.1(1), NP_312469.1(1), NP_312470.1(1), NP_312477.1(1), NP_312494.1(1), NP_312495.1(1), NP_312511.1(1), NP_312537.1(1), NP_312538.1(1), NP_312539.1(1), NP_312545.1(1), NP_312551.1(1), NP_312553.1(1), NP_312554.1(1), NP_312595.1(1), NP_312625.1(1), NP_312640.1(1), NP_312665.1(1), NP_312666.1(1), NP_312668.1(1), NP_312671.1(1), NP_312688.1(1), NP_312709.1(1), NP_312710.1(1), NP_312718.1(1), NP_312728.1(1), NP_312740.1(1), NP_312743.1(1), NP_312773.1(1), NP_312779.1(1), NP_312792.1(1), NP_312810.1(1), NP_312813.1(1), NP_312820.1(1), NP_312837.1(1), NP_312889.1(1), NP_312890.1(1), NP_312923.1(1), NP_312930.1(1), NP_312931.1(1), NP_312933.1(1), NP_312934.1(1), NP_312935.1(1), NP_312936.1(1), NP_312937.1(1), NP_312938.1(1), NP_312945.1(1), NP_313032.1(1), NP_313045.1(1), NP_313058.1(1), NP_313067.1(1), NP_313100.1(1), NP_313106.1(1), NP_313107.1(1), NP_313115.1(1), NP_313138.1(1), NP_313163.2(1), NP_313167.2(1), NP_313168.2(1), NP_313170.1(1), NP_313174.1(1), NP_313175.1(1), NP_313176.1(1), NP_313182.2(1), NP_313183.1(1), NP_313203.1(1), NP_313205.1(1), NP_313206.1(1), NP_313229.1(1), NP_313230.1(1), NP_313233.1(1), NP_313247.1(1), NP_313252.1(1), NP_313262.1(1), NP_313287.1(1), NP_313288.1(1), NP_313290.1(1), NP_313316.2(1), NP_313334.1(1), NP_313335.1(1), NP_313360.1(1), NP_313376.1(1), NP_313388.1(1), NP_414559.1(1), NP_414564.1(1), NP_414567.1(1), NP_414593.1(1), NP_414600.1(1), NP_414601.1(1), NP_414608.1(1), NP_414611.1(1), NP_414614.1(1), NP_414636.1(1), NP_414660.1(1), NP_414669.1(1), NP_414685.4(1), NP_414686.3(1), NP_414689.4(1), NP_414690.4(1), NP_414693.1(1), NP_414702.1(1), NP_414711.1(1), NP_414712.1(1), NP_414714.1(1), NP_414725.1(1), NP_414726.1(1), NP_414730.1(1), NP_414733.1(1), NP_414736.1(1), NP_414741.1(1), NP_414750.1(1), NP_414751.1(1), NP_414768.1(1), NP_414777.1(1), NP_414796.2(1), NP_414852.1(1), NP_414900.1(1), NP_414939.1(1), NP_414940.1(1), NP_414948.1(1), NP_414950.1(1), NP_414957.1(1), NP_414970.1(1), NP_414979.1(1), NP_414982.1(1), NP_414983.1(1), NP_415007.1(1), NP_415014.1(1), NP_415023.1(1), NP_415028.1(1), NP_415059.1(1), NP_415120.1(1), NP_415156.1(1), NP_415175.1(1), NP_415185.1(1), NP_415206.1(1), NP_415242.1(1), NP_415281.1(1), NP_415286.1(1), NP_415292.2(1), NP_415300.1(1), NP_415315.4(1), NP_415318.1(1), NP_415320.1(1), NP_415330.1(1), NP_415341.1(1), NP_415350.2(1), NP_415376.4(1), NP_415380.1(1), NP_415385.1(1), NP_415400.1(1), NP_415401.1(1), NP_415406.1(1), NP_415407.1(1), NP_415413.1(1), NP_415431.1(1), NP_415434.1(1), NP_415437.1(1), NP_415441.1(1), NP_415450.1(1), NP_415453.1(1), NP_415468.1(1), NP_415469.1(1), NP_415509.1(1), NP_415510.1(1), NP_415530.1(1), NP_415563.1(1), NP_415602.1(1), NP_415604.1(1), NP_415607.1(1), NP_415621.3(1), NP_415632.1(1), NP_415635.4(1), NP_415644.1(1), NP_415651.4(1), NP_415653.4(1), NP_415721.1(1), NP_415722.1(1), NP_415728.1(1), NP_415729.1(1), NP_415730.1(1), NP_415748.1(1), NP_415763.1(1), NP_415783.6(1), NP_415785.1(1), NP_415792.1(1), NP_415802.1(1), NP_415806.1(1), NP_415807.1(1), NP_415858.1(1), NP_415859.1(1), NP_415860.1(1), NP_415931.4(1), NP_415958.1(1), NP_416000.1(1), NP_416001.1(1), NP_416013.1(1), NP_416030.1(1), NP_416036.1(1), NP_416070.1(1), NP_416075.1(1), NP_416076.1(1), NP_416080.1(1), NP_416154.1(1), NP_416169.1(1), NP_416170.1(1), NP_416197.1(1), NP_416224.1(1), NP_416228.1(1), NP_416229.1(1), NP_416231.1(1), NP_416232.3(1), NP_416233.1(1), NP_416234.1(1), NP_416270.1(1), NP_416301.1(1), NP_416318.1(1), NP_416322.1(1), NP_416323.4(1), NP_416337.1(1), NP_416349.2(1), NP_416358.1(1), NP_416372.1(1), NP_416380.1(1), NP_416390.1(1), NP_416398.1(1), NP_416413.1(1), NP_416427.1(1), NP_416451.1(1), NP_416515.1(1), NP_416523.1(1), NP_416529.1(1), NP_416617.1(1), NP_416633.1(1), NP_416645.1(1), NP_416652.4(1), NP_416654.1(1), NP_416685.1(1), NP_416688.1(1), NP_416689.1(1), NP_416690.1(1), NP_416705.2(1), NP_416715.1(1), NP_416758.1(1), NP_416771.4(1), NP_416809.1(1), NP_416821.1(1), NP_416833.4(1), NP_416899.1(1), NP_416917.1(1), NP_416969.1(1), NP_416995.1(1), NP_417002.1(1), NP_417004.1(1), NP_417006.2(1), NP_417009.1(1), NP_417027.1(1), NP_417030.2(1), NP_417042.1(1), NP_417054.2(1), NP_417061.1(1), NP_417062.1(1), NP_417064.1(1), NP_417070.2(1), NP_417071.1(1), NP_417076.1(1), NP_417085.1(1), NP_417097.1(1), NP_417098.1(1), NP_417099.4(1), NP_417100.1(1), NP_417101.1(1), NP_417108.4(1), NP_417110.1(1), NP_417116.1(1), NP_417119.1(1), NP_417163.1(1), NP_417176.1(1), NP_417177.1(1), NP_417192.1(1), NP_417213.1(1), NP_417223.1(1), NP_417225.1(1), NP_417231.1(1), NP_417236.1(1), NP_417237.2(1), NP_417238.1(1), NP_417239.1(1), NP_417240.1(1), NP_417241.1(1), NP_417262.1(1), NP_417263.1(1), NP_417265.1(1), NP_417271.1(1), NP_417286.1(1), NP_417296.1(1), NP_417366.1(1), NP_417367.1(1), NP_417368.1(1), NP_417376.1(1), NP_417420.1(1), NP_417421.4(1), NP_417493.1(1), NP_417494.1(1), NP_417528.1(1), NP_417537.1(1), NP_417538.1(1), NP_417539.1(1), NP_417545.1(1), NP_417583.4(1), NP_417631.2(1), NP_417633.4(1), NP_417634.1(1), NP_417635.1(1), NP_417637.1(1), NP_417638.1(1), NP_417642.1(1), NP_417646.1(1), NP_417647.1(1), NP_417650.1(1), NP_417652.1(1), NP_417653.1(1), NP_417662.1(1), NP_417668.1(1), NP_417669.1(1), NP_417697.1(1), NP_417698.1(1), NP_417713.2(1), NP_417726.1(1), NP_417737.1(1), NP_417741.1(1), NP_417746.1(1), NP_417747.1(1), NP_417753.1(1), NP_417754.1(1), NP_417755.1(1), NP_417756.1(1), NP_417757.1(1), NP_417758.1(1), NP_417759.1(1), NP_417760.1(1), NP_417761.1(1), NP_417762.1(1), NP_417763.1(1), NP_417764.1(1), NP_417765.1(1), NP_417766.1(1), NP_417767.1(1), NP_417768.1(1), NP_417769.1(1), NP_417770.1(1), NP_417771.1(1), NP_417772.1(1), NP_417773.1(1), NP_417774.1(1), NP_417775.1(1), NP_417776.1(1), NP_417777.1(1), NP_417778.1(1), NP_417779.1(1), NP_417780.1(1), NP_417798.1(1), NP_417799.1(1), NP_417800.1(1), NP_417801.1(1), NP_417811.1(1), NP_417843.1(1), NP_417859.1(1), NP_417866.4(1), NP_417879.1(1), NP_417880.1(1), NP_417907.2(1), NP_417911.4(1), NP_417912.1(1), NP_417920.1(1), NP_417921.1(1), NP_417936.1(1), NP_417937.1(1), NP_417956.1(1), NP_417997.1(1), NP_417998.1(1), NP_418011.1(1), NP_418012.1(1), NP_418016.1(1), NP_418017.1(1), NP_418024.1(1), NP_418047.1(1), NP_418048.1(1), NP_418063.1(1), NP_418092.1(1), NP_418093.1(1), NP_418094.1(1), NP_418106.1(1), NP_418108.1(1), NP_418109.1(1), NP_418128.1(1), NP_418158.1(1), NP_418159.1(1), NP_418162.1(1), NP_418163.1(1), NP_418179.1(1), NP_418181.1(1), NP_418196.1(1), NP_418197.1(1), NP_418205.1(1), NP_418215.1(1), NP_418227.1(1), NP_418230.1(1), NP_418260.1(1), NP_418279.1(1), NP_418297.1(1), NP_418300.1(1), NP_418323.1(1), NP_418370.1(1), NP_418371.1(1), NP_418400.1(1), NP_418407.1(1), NP_418408.1(1), NP_418410.1(1), NP_418411.1(1), NP_418412.1(1), NP_418413.1(1), NP_418414.1(1), NP_418415.1(1), NP_418423.1(1), NP_418446.1(1), NP_418459.1(1), NP_418473.3(1), NP_418482.1(1), NP_418511.1(1), NP_418520.1(1), NP_418530.1(1), NP_418553.1(1), NP_418579.2(1), NP_418585.4(1), NP_418586.4(1), NP_418588.1(1), NP_418592.1(1), NP_418593.1(1), NP_418594.1(1), NP_418600.4(1), NP_418601.1(1), NP_418621.5(1), NP_418623.1(1), NP_418624.1(1), NP_418645.2(1), NP_418646.1(1), NP_418664.2(1), NP_418669.1(1), NP_418679.1(1), NP_418707.1(1), NP_418751.4(1), NP_418767.4(1), NP_418770.2(1), NP_418792.1(1), NP_418808.1(1), NP_418820.1(1), NP_944503.1(1), NP_944505.1(1), NP_944516.1(1), NP_944530.1(1), NP_944534.1(1), NP_944586.1(1), NP_944589.1(1), WP_000003060.1(1), WP_000003074.1(1), WP_000003077.1(1), WP_000003086.1(1), WP_000003799.1(1), WP_000003800.1(1), WP_000003801.1(1), WP_000003808.1(1), WP_000004420.1(1), WP_000004426.1(1), WP_000004443.1(1), WP_000004453.1(1), WP_000004468.1(1), WP_000004470.1(1), WP_000004476.1(1), WP_000004479.1(1), WP_000004751.1(1), WP_000004946.1(1), WP_000007088.1(1), WP_000007090.1(1), WP_000007094.1(1), WP_000007106.1(1), WP_000007113.1(1), WP_000007121.1(1), WP_000007122.1(1), WP_000007131.1(1), WP_000007135.1(1), WP_000007138.1(1), WP_000007142.1(1), WP_000007144.1(1), WP_000007431.1(1), WP_000012516.1(1), WP_000012960.1(1), WP_000018428.1(1), WP_000018593.1(1), WP_000018594.1(1), WP_000020504.1(1), WP_000021011.1(1), WP_000021041.1(1), WP_000021044.1(1), WP_000021046.1(1), WP_000021050.1(1), WP_000023626.1(1), WP_000023629.1(1), WP_000023634.1(1), WP_000023638.1(1), WP_000023648.1(1), WP_000023754.1(1), WP_000023755.1(1), WP_000025035.1(1), WP_000027192.1(1), WP_000027213.1(1), WP_000027828.1(1), WP_000029313.1(1), WP_000029328.1(1), WP_000029332.1(1), WP_000029333.1(1), WP_000029467.1(1), WP_000029476.1(1), WP_000031812.1(1), WP_000035580.1(1), WP_000041955.1(1), WP_000041965.1(1), WP_000041979.1(1), WP_000042156.1(1), WP_000042509.1(1), WP_000042520.1(1), WP_000042528.1(1), WP_000042530.1(1), WP_000042532.1(1), WP_000042540.1(1), WP_000046731.1(1), WP_000046750.1(1), WP_000046752.1(1), WP_000046757.1(1), WP_000046761.1(1), WP_000046778.1(1), WP_000046792.1(1), WP_000046793.1(1), WP_000046794.1(1), WP_000046807.1(1), WP_000046808.1(1), WP_000046827.1(1), WP_000047155.1(1), WP_000047168.1(1), WP_000047171.1(1), WP_000047172.1(1), WP_000047191.1(1), WP_000047192.1(1), WP_000047199.1(1), WP_000047202.1(1), WP_000047211.1(1), WP_000047507.1(1), WP_000047509.1(1), WP_000047510.1(1), WP_000052512.1(1), WP_000053062.1(1), WP_000053079.1(1), WP_000053084.1(1), WP_000053091.1(1), WP_000053110.1(1), WP_000053113.1(1), WP_000053127.1(1), WP_000053130.1(1), WP_000057021.1(1), WP_000057076.1(1), WP_000057082.1(1), WP_000057087.1(1), WP_000057101.1(1), WP_000057108.1(1), WP_000057967.1(1), WP_000057970.1(1), WP_000057973.1(1), WP_000057982.1(1), WP_000057983.1(1), WP_000060048.1(1), WP_000064443.1(1), WP_000066154.1(1), WP_000066189.1(1), WP_000066483.1(1), WP_000066496.1(1), WP_000068004.1(1), WP_000068008.1(1), WP_000068433.1(1), WP_000070069.1(1), WP_000072429.1(1), WP_000076285.1(1), WP_000076289.1(1), WP_000076315.1(1), WP_000076318.1(1), WP_000076326.1(1), WP_000076337.1(1), WP_000078312.1(1), WP_000078316.1(1), WP_000078323.1(1), WP_000078330.1(1), WP_000078333.1(1), WP_000078342.1(1), WP_000078353.1(1), WP_000078921.1(1), WP_000078923.1(1), WP_000078925.1(1), WP_000079089.1(1), WP_000079091.1(1), WP_000079094.1(1), WP_000081424.1(1), WP_000082107.1(1), WP_000085038.1(1), WP_000085041.1(1), WP_000085044.1(1), WP_000085070.1(1), WP_000085071.1(1), WP_000085073.1(1), WP_000085074.1(1), WP_000085075.1(1), WP_000093092.1(1), WP_000093094.1(1), WP_000096832.1(1), WP_000096834.1(1), WP_000096838.1(1), WP_000096840.1(1), WP_000096846.1(1), WP_000096859.1(1), WP_000096865.1(1), WP_000096871.1(1), WP_000096882.1(1), WP_000096883.1(1), WP_000096893.1(1), WP_000096905.1(1), WP_000099453.1(1), WP_000099487.1(1), WP_000099497.1(1), WP_000099499.1(1), WP_000099515.1(1), WP_000099524.1(1), WP_000099527.1(1), WP_000099537.1(1), WP_000099548.1(1), WP_000099551.1(1), WP_000099559.1(1), WP_000103570.1(1), WP_000103577.1(1), WP_000103582.1(1), WP_000103866.1(1), WP_000106696.1(1), WP_000107022.1(1), WP_000107023.1(1), WP_000107258.1(1), WP_000110930.1(1), WP_000110961.1(1), WP_000110969.1(1), WP_000110972.1(1), WP_000120386.1(1), WP_000120464.1(1), WP_000123727.1(1), WP_000123748.1(1), WP_000123753.1(1), WP_000123756.1(1), WP_000123758.1(1), WP_000123759.1(1), WP_000123771.1(1), WP_000123773.1(1), WP_000123775.1(1), WP_000123794.1(1), WP_000124701.1(1), WP_000125555.1(1), WP_000125642.1(1), WP_000125647.1(1), WP_000126278.1(1), WP_000126284.1(1), WP_000127306.1(1), WP_000127307.1(1), WP_000128513.1(1), WP_000128827.1(1), WP_000128832.1(1), WP_000128835.1(1), WP_000128837.1(1), WP_000128845.1(1), WP_000128853.1(1), WP_000128855.1(1), WP_000128856.1(1), WP_000128863.1(1), WP_000128866.1(1), WP_000128877.1(1), WP_000131233.1(1), WP_000131767.1(1), WP_000131777.1(1), WP_000131783.1(1), WP_000131790.1(1), WP_000131791.1(1), WP_000131792.1(1), WP_000132443.1(1), WP_000132553.1(1), WP_000132602.1(1), WP_000132623.1(1), WP_000132625.1(1), WP_000132631.1(1), WP_000132632.1(1), WP_000132634.1(1), WP_000132904.1(1), WP_000132906.1(1), WP_000132908.1(1), WP_000132917.1(1), WP_000133033.1(1), WP_000133046.1(1), WP_000135228.1(1), WP_000138058.1(1), WP_000138059.1(1), WP_000138264.1(1), WP_000138279.1(1), WP_000138288.1(1), WP_000139309.1(1), WP_000139312.1(1), WP_000139325.1(1), WP_000139327.1(1), WP_000139329.1(1), WP_000139345.1(1), WP_000139351.1(1), WP_000139363.1(1), WP_000139366.1(1), WP_000139370.1(1), WP_000139378.1(1), WP_000139499.1(1), WP_000139520.1(1), WP_000139521.1(1), WP_000139532.1(1), WP_000139534.1(1), WP_000139542.1(1), WP_000139546.1(1), WP_000139556.1(1), WP_000139565.1(1), WP_000139568.1(1), WP_000139580.1(1), WP_000139589.1(1), WP_000139594.1(1), WP_000139603.1(1), WP_000139605.1(1), WP_000139616.1(1), WP_000139621.1(1), WP_000140635.1(1), WP_000140648.1(1), WP_000145290.1(1), WP_000145966.1(1), WP_000145977.1(1), WP_000149084.1(1), WP_000149096.1(1), WP_000149099.1(1), WP_000149100.1(1), WP_000149105.1(1), WP_000149118.1(1), WP_000149125.1(1), WP_000149130.1(1), WP_000149135.1(1), WP_000149152.1(1), WP_000149153.1(1), WP_000149160.1(1), WP_000149167.1(1), WP_000149168.1(1), WP_000149176.1(1), WP_000149178.1(1), WP_000149182.1(1), WP_000149184.1(1), WP_000149595.1(1), WP_000149665.1(1), WP_000149674.1(1), WP_000149677.1(1), WP_000150605.1(1), WP_000150642.1(1), WP_000150646.1(1), WP_000152765.1(1), WP_000152853.1(1), WP_000152927.1(1), WP_000152948.1(1), WP_000153095.1(1), WP_000153098.1(1), WP_000153103.1(1), WP_000153109.1(1), WP_000153112.1(1), WP_000157344.1(1), WP_000158029.1(1), WP_000162575.1(1), WP_000162576.1(1), WP_000163441.1(1), WP_000163465.1(1), WP_000163467.1(1), WP_000163472.1(1), WP_000164224.1(1), WP_000164226.1(1), WP_000165427.1(1), WP_000165540.1(1), WP_000165551.1(1), WP_000166947.1(1), WP_000168638.1(1), WP_000170738.1(1), WP_000173311.1(1), WP_000173314.1(1), WP_000173316.1(1), WP_000173319.1(1), WP_000173650.1(1), WP_000173667.1(1), WP_000173677.1(1), WP_000173687.1(1), WP_000173688.1(1), WP_000173693.1(1), WP_000175453.1(1), WP_000175458.1(1), WP_000175942.1(1), WP_000175947.1(1), WP_000175954.1(1), WP_000175958.1(1), WP_000176516.1(1), WP_000176517.1(1), WP_000176522.1(1), WP_000176525.1(1), WP_000176528.1(1), WP_000176529.1(1), WP_000176570.1(1), WP_000176585.1(1), WP_000178825.1(1), WP_000186640.1(1), WP_000186996.1(1), WP_000186999.1(1), WP_000187002.1(1), WP_000187003.1(1), WP_000187028.1(1), WP_000187040.1(1), WP_000187546.1(1), WP_000187548.1(1), WP_000188123.1(1), WP_000188130.1(1), WP_000188133.1(1), WP_000188136.1(1), WP_000188141.1(1), WP_000188142.1(1), WP_000188148.1(1), WP_000188185.1(1), WP_000188186.1(1), WP_000188197.1(1), WP_000193114.1(1), WP_000193511.1(1), WP_000193514.1(1), WP_000193515.1(1), WP_000193520.1(1), WP_000193545.1(1), WP_000193546.1(1), WP_000193551.1(1), WP_000193552.1(1), WP_000193556.1(1), WP_000193561.1(1), WP_000193565.1(1), WP_000193575.1(1), WP_000194877.1(1), WP_000194891.1(1), WP_000194893.1(1), WP_000194895.1(1), WP_000194913.1(1), WP_000194916.1(1), WP_000194931.1(1), WP_000194938.1(1), WP_000194939.1(1), WP_000198514.1(1), WP_000202985.1(1), WP_000203010.1(1), WP_000204051.1(1), WP_000204316.1(1), WP_000204329.1(1), WP_000204351.1(1), WP_000205781.1(1), WP_000205787.1(1), WP_000205791.1(1), WP_000205795.1(1), WP_000205810.1(1), WP_000205814.1(1), WP_000205815.1(1), WP_000205833.1(1), WP_000205835.1(1), WP_000206233.1(1), WP_000206234.1(1), WP_000206235.1(1), WP_000206237.1(1), WP_000206249.1(1), WP_000206258.1(1), WP_000206264.1(1), WP_000206270.1(1), WP_000206279.1(1), WP_000206280.1(1), WP_000206287.1(1), WP_000206407.1(1), WP_000206427.1(1), WP_000206433.1(1), WP_000206441.1(1), WP_000206444.1(1), WP_000206451.1(1), WP_000206452.1(1), WP_000206454.1(1), WP_000209995.1(1), WP_000210554.1(1), WP_000213266.1(1), WP_000213269.1(1), WP_000213278.1(1), WP_000213288.1(1), WP_000213293.1(1), WP_000213307.1(1), WP_000213309.1(1), WP_000213316.1(1), WP_000213597.1(1), WP_000216181.1(1), WP_000216182.1(1), WP_000220391.1(1), WP_000220393.1(1), WP_000220394.1(1), WP_000220407.1(1), WP_000220430.1(1), WP_000220433.1(1), WP_000224102.1(1), WP_000230261.1(1), WP_000230264.1(1), WP_000230270.1(1), WP_000230272.1(1), WP_000230275.1(1), WP_000230276.1(1), WP_000233457.1(1), WP_000235238.1(1), WP_000235243.1(1), WP_000235244.1(1), WP_000235246.1(1), WP_000235247.1(1), WP_000235252.1(1), WP_000235253.1(1), WP_000235255.1(1), WP_000235256.1(1), WP_000235261.1(1), WP_000239576.1(1), WP_000239729.1(1), WP_000239731.1(1), WP_000245512.1(1), WP_000249409.1(1), WP_000249991.1(1), WP_000249992.1(1), WP_000249993.1(1), WP_000249994.1(1), WP_000250008.1(1), WP_000250017.1(1), WP_000250020.1(1), WP_000250025.1(1), WP_000250042.1(1), WP_000250057.1(1), WP_000254699.1(1), WP_000254713.1(1), WP_000254739.1(1), WP_000254749.1(1), WP_000255013.1(1), WP_000255021.1(1), WP_000255027.1(1), WP_000255030.1(1), WP_000255046.1(1), WP_000264774.1(1), WP_000281394.1(1), WP_000281438.1(1), WP_000281439.1(1), WP_000281440.1(1), WP_000281441.1(1), WP_000281443.1(1), WP_000281444.1(1), WP_000281448.1(1), WP_000281454.1(1), WP_000281456.1(1), WP_000281457.1(1), WP_000286489.1(1), WP_000286493.1(1), WP_000286498.1(1), WP_000286507.1(1), WP_000301867.1(1), WP_000340177.1(1), WP_000340178.1(1), WP_000349544.1(1), WP_000357732.1(1), WP_000357768.1(1), WP_000359986.1(1), WP_000361403.1(1), WP_000373724.1(1), WP_000376538.1(1), WP_000376541.1(1), WP_000376544.1(1), WP_000383112.1(1), WP_000383115.1(1), WP_000383119.1(1), WP_000383121.1(1), WP_000387271.1(1), WP_000387390.1(1), WP_000387391.1(1), WP_000387749.1(1), WP_000387751.1(1), WP_000387768.1(1), WP_000387771.1(1), WP_000392434.1(1), WP_000399285.1(1), WP_000410856.1(1), WP_000410858.1(1), WP_000415583.1(1), WP_000416367.1(1), WP_000416376.1(1), WP_000416379.1(1), WP_000416388.1(1), WP_000416389.1(1), WP_000416393.1(1), WP_000416396.1(1), WP_000416400.1(1), WP_000416404.1(1), WP_000416419.1(1), WP_000416426.1(1), WP_000416885.1(1), WP_000416894.1(1), WP_000416899.1(1), WP_000419110.1(1), WP_000420113.1(1), WP_000420116.1(1), WP_000420119.1(1), WP_000422112.1(1), WP_000422181.1(1), WP_000422187.1(1), WP_000422191.1(1), WP_000422199.1(1), WP_000422219.1(1), WP_000422231.1(1), WP_000422234.1(1), WP_000422235.1(1), WP_000422240.1(1), WP_000422610.1(1), WP_000424041.1(1), WP_000431363.1(1), WP_000431372.1(1), WP_000431374.1(1), WP_000431375.1(1), WP_000431378.1(1), WP_000431385.1(1), WP_000431483.1(1), WP_000433151.1(1), WP_000433157.1(1), WP_000435028.1(1), WP_000437378.1(1), WP_000437381.1(1), WP_000438153.1(1), WP_000438168.1(1), WP_000438169.1(1), WP_000438239.1(1), WP_000438244.1(1), WP_000438249.1(1), WP_000439380.1(1), WP_000447342.1(1), WP_000447343.1(1), WP_000447532.1(1), WP_000450530.1(1), WP_000450587.1(1), WP_000450590.1(1), WP_000451769.1(1), WP_000456455.1(1), WP_000456459.1(1), WP_000456460.1(1), WP_000460026.1(1), WP_000460039.1(1), WP_000460043.1(1), WP_000460334.1(1), WP_000460361.1(1), WP_000479990.1(1), WP_000484968.1(1), WP_000485010.1(1), WP_000485015.1(1), WP_000485016.1(1), WP_000485061.1(1), WP_000493446.1(1), WP_000493450.1(1), WP_000493456.1(1), WP_000493476.1(1), WP_000493478.1(1), WP_000493482.1(1), WP_000493484.1(1), WP_000493485.1(1), WP_000493507.1(1), WP_000493508.1(1), WP_000495599.1(1), WP_000499756.1(1), WP_000499785.1(1), WP_000499802.1(1), WP_000499807.1(1), WP_000499810.1(1), WP_000502509.1(1), WP_000502511.1(1), WP_000502514.1(1), WP_000505862.1(1), WP_000505874.1(1), WP_000510232.1(1), WP_000516400.1(1), WP_000517689.1(1), WP_000517692.1(1), WP_000521550.1(1), WP_000521551.1(1), WP_000521561.1(1), WP_000521582.1(1), WP_000521584.1(1), WP_000521585.1(1), WP_000525158.1(1), WP_000531577.1(1), WP_000531602.1(1), WP_000537018.1(1), WP_000543832.1(1), WP_000546468.1(1), WP_000550448.1(1), WP_000551260.1(1), WP_000551261.1(1), WP_000551263.1(1), WP_000551268.1(1), WP_000555854.1(1), WP_000555858.1(1), WP_000555860.1(1), WP_000560978.1(1), WP_000560988.1(1), WP_000568912.1(1), WP_000568923.1(1), WP_000568933.1(1), WP_000568936.1(1), WP_000568952.1(1), WP_000568953.1(1), WP_000568955.1(1), WP_000568966.1(1), WP_000568972.1(1), WP_000569079.1(1), WP_000569087.1(1), WP_000569311.1(1), WP_000569314.1(1), WP_000569318.1(1), WP_000569341.1(1), WP_000569342.1(1), WP_000569360.1(1), WP_000569373.1(1), WP_000569385.1(1), WP_000569406.1(1), WP_000569417.1(1), WP_000569426.1(1), WP_000569428.1(1), WP_000569432.1(1), WP_000570675.1(1), WP_000573409.1(1), WP_000578039.1(1), WP_000578046.1(1), WP_000578049.1(1), WP_000578050.1(1), WP_000578052.1(1), WP_000578053.1(1), WP_000578065.1(1), WP_000578093.1(1), WP_000578098.1(1), WP_000579832.1(1), WP_000579836.1(1), WP_000580531.1(1), WP_000580534.1(1), WP_000581940.1(1), WP_000581941.1(1), WP_000582416.1(1), WP_000582417.1(1), WP_000582422.1(1), WP_000582423.1(1), WP_000582427.1(1), WP_000582436.1(1), WP_000582442.1(1), WP_000582456.1(1), WP_000582463.1(1), WP_000582479.1(1), WP_000582487.1(1), WP_000582488.1(1), WP_000582493.1(1), WP_000582497.1(1), WP_000582830.1(1), WP_000586419.1(1), WP_000586437.1(1), WP_000586442.1(1), WP_000586450.1(1), WP_000586461.1(1), WP_000586474.1(1), WP_000586493.1(1), WP_000586499.1(1), WP_000586515.1(1), WP_000588467.1(1), WP_000590233.1(1), WP_000590237.1(1), WP_000590242.1(1), WP_000590248.1(1), WP_000590249.1(1), WP_000590254.1(1), WP_000593828.1(1), WP_000593869.1(1), WP_000593889.1(1), WP_000593895.1(1), WP_000593898.1(1), WP_000593908.1(1), WP_000593910.1(1), WP_000593913.1(1), WP_000593924.1(1), WP_000593931.1(1), WP_000593949.1(1), WP_000593964.1(1), WP_000593973.1(1), WP_000593983.1(1), WP_000594002.1(1), WP_000594004.1(1), WP_000599710.1(1), WP_000602100.1(1), WP_000602101.1(1), WP_000602109.1(1), WP_000602120.1(1), WP_000602121.1(1), WP_000602124.1(1), WP_000602125.1(1), WP_000604346.1(1), WP_000611392.1(1), WP_000611403.1(1), WP_000611404.1(1), WP_000611412.1(1), WP_000611423.1(1), WP_000611426.1(1), WP_000611434.1(1), WP_000611438.1(1), WP_000613956.1(1), WP_000617718.1(1), WP_000621745.1(1), WP_000622542.1(1), WP_000622543.1(1), WP_000622549.1(1), WP_000622550.1(1), WP_000627725.1(1), WP_000627726.1(1), WP_000628536.1(1), WP_000628540.1(1), WP_000628541.1(1), WP_000628543.1(1), WP_000628553.1(1), WP_000628555.1(1), WP_000628557.1(1), WP_000628562.1(1), WP_000628566.1(1), WP_000628587.1(1), WP_000631391.1(1), WP_000634316.1(1), WP_000634794.1(1), WP_000634796.1(1), WP_000634811.1(1), WP_000634821.1(1), WP_000634825.1(1), WP_000634832.1(1), WP_000634841.1(1), WP_000635316.1(1), WP_000635526.1(1), WP_000635527.1(1), WP_000635547.1(1), WP_000635548.1(1), WP_000635557.1(1), WP_000653936.1(1), WP_000653940.1(1), WP_000653950.1(1), WP_000653957.1(1), WP_000653958.1(1), WP_000660598.1(1), WP_000667301.1(1), WP_000667320.1(1), WP_000667322.1(1), WP_000668660.1(1), WP_000668661.1(1), WP_000668668.1(1), WP_000668677.1(1), WP_000668691.1(1), WP_000668695.1(1), WP_000671670.1(1), WP_000671695.1(1), WP_000671698.1(1), WP_000671704.1(1), WP_000671705.1(1), WP_000672319.1(1), WP_000672338.1(1), WP_000672340.1(1), WP_000672341.1(1), WP_000672343.1(1), WP_000672346.1(1), WP_000672347.1(1), WP_000672356.1(1), WP_000672366.1(1), WP_000672369.1(1), WP_000672378.1(1), WP_000672421.1(1), WP_000672423.1(1), WP_000673570.1(1), WP_000673576.1(1), WP_000675892.1(1), WP_000678394.1(1), WP_000678395.1(1), WP_000678396.1(1), WP_000678401.1(1), WP_000678406.1(1), WP_000678421.1(1), WP_000678424.1(1), WP_000678437.1(1), WP_000678441.1(1), WP_000678447.1(1), WP_000678455.1(1), WP_000678477.1(1), WP_000691727.1(1), WP_000695422.1(1), WP_000695633.1(1), WP_000695649.1(1), WP_000695668.1(1), WP_000695676.1(1), WP_000708468.1(1), WP_000708484.1(1), WP_000708491.1(1), WP_000708492.1(1), WP_000708493.1(1), WP_000708504.1(1), WP_000708508.1(1), WP_000708513.1(1), WP_000708517.1(1), WP_000708522.1(1), WP_000719977.1(1), WP_000725338.1(1), WP_000725340.1(1), WP_000725354.1(1), WP_000734310.1(1), WP_000734317.1(1), WP_000739041.1(1), WP_000739052.1(1), WP_000739053.1(1), WP_000739059.1(1), WP_000739068.1(1), WP_000741721.1(1), WP_000741722.1(1), WP_000744767.1(1), WP_000744768.1(1), WP_000744782.1(1), WP_000752799.1(1), WP_000754566.1(1), WP_000766113.1(1), WP_000775234.1(1), WP_000775236.1(1), WP_000775917.1(1), WP_000775925.1(1), WP_000775926.1(1), WP_000775937.1(1), WP_000775940.1(1), WP_000775942.1(1), WP_000775943.1(1), WP_000775956.1(1), WP_000775961.1(1), WP_000775967.1(1), WP_000775974.1(1), WP_000775975.1(1), WP_000775978.1(1), WP_000775995.1(1), WP_000775999.1(1), WP_000790160.1(1), WP_000790161.1(1), WP_000790178.1(1), WP_000801123.1(1), WP_000804719.1(1), WP_000804723.1(1), WP_000807556.1(1), WP_000808996.1(1), WP_000808997.1(1), WP_000809036.1(1), WP_000809037.1(1), WP_000809043.1(1), WP_000809165.1(1), WP_000809174.1(1), WP_000813176.1(1), WP_000813188.1(1), WP_000813191.1(1), WP_000813193.1(1), WP_000813194.1(1), WP_000813198.1(1), WP_000813207.1(1), WP_000813208.1(1), WP_000813223.1(1), WP_000813224.1(1), WP_000813233.1(1), WP_000813239.1(1), WP_000813264.1(1), WP_000817647.1(1), WP_000818119.1(1), WP_000818124.1(1), WP_000827365.1(1), WP_000827376.1(1), WP_000827385.1(1), WP_000827392.1(1), WP_000827393.1(1), WP_000827395.1(1), WP_000827402.1(1), WP_000827403.1(1), WP_000827405.1(1), WP_000827410.1(1), WP_000827416.1(1), WP_000827417.1(1), WP_000827420.1(1), WP_000827438.1(1), WP_000827440.1(1), WP_000829310.1(1), WP_000829312.1(1), WP_000829329.1(1), WP_000829334.1(1), WP_000829343.1(1), WP_000829344.1(1), WP_000829349.1(1), WP_000829352.1(1), WP_000829362.1(1), WP_000829364.1(1), WP_000829370.1(1), WP_000829373.1(1), WP_000829376.1(1), WP_000835429.1(1), WP_000835433.1(1), WP_000840467.1(1), WP_000840475.1(1), WP_000845150.1(1), WP_000846334.1(1), WP_000846352.1(1), WP_000857406.1(1), WP_000857411.1(1), WP_000860254.1(1), WP_000860265.1(1), WP_000860276.1(1), WP_000860280.1(1), WP_000860281.1(1), WP_000860288.1(1), WP_000860292.1(1), WP_000860294.1(1), WP_000860298.1(1), WP_000860307.1(1), WP_000860314.1(1), WP_000873386.1(1), WP_000880132.1(1), WP_000880157.1(1), WP_000880158.1(1), WP_000880163.1(1), WP_000880167.1(1), WP_000880172.1(1), WP_000880173.1(1), WP_000880183.1(1), WP_000880184.1(1), WP_000880189.1(1), WP_000880205.1(1), WP_000882662.1(1), WP_000884071.1(1), WP_000884073.1(1), WP_000886685.1(1), WP_000887493.1(1), WP_000887678.1(1), WP_000889985.1(1), WP_000889992.1(1), WP_000890005.1(1), WP_000890012.1(1), WP_000891657.1(1), WP_000891664.1(1), WP_000891667.1(1), WP_000891670.1(1), WP_000894341.1(1), WP_000901118.1(1), WP_000902692.1(1), WP_000902696.1(1), WP_000902698.1(1), WP_000903984.1(1), WP_000903988.1(1), WP_000904007.1(1), WP_000904008.1(1), WP_000904013.1(1), WP_000904015.1(1), WP_000904019.1(1), WP_000907777.1(1), WP_000907796.1(1), WP_000907815.1(1), WP_000907822.1(1), WP_000907825.1(1), WP_000907828.1(1), WP_000907829.1(1), WP_000909594.1(1), WP_000911327.1(1), WP_000911333.1(1), WP_000912341.1(1), WP_000912354.1(1), WP_000913065.1(1), WP_000913117.1(1), WP_000916261.1(1), WP_000916263.1(1), WP_000916270.1(1), WP_000916274.1(1), WP_000916286.1(1), WP_000916287.1(1), WP_000916292.1(1), WP_000916297.1(1), WP_000916300.1(1), WP_000916309.1(1), WP_000918804.1(1), WP_000932348.1(1), WP_000932361.1(1), WP_000932553.1(1), WP_000932842.1(1), WP_000932845.1(1), WP_000934308.1(1), WP_000934310.1(1), WP_000936348.1(1), WP_000936369.1(1), WP_000936386.1(1), WP_000937456.1(1), WP_000937862.1(1), WP_000937871.1(1), WP_000937891.1(1), WP_000937893.1(1), WP_000937902.1(1), WP_000937918.1(1), WP_000937921.1(1), WP_000937937.1(1), WP_000937948.1(1), WP_000937952.1(1), WP_000937965.1(1), WP_000937966.1(1), WP_000937970.1(1), WP_000937975.1(1), WP_000937976.1(1), WP_000938497.1(1), WP_000938501.1(1), WP_000938503.1(1), WP_000938506.1(1), WP_000938561.1(1), WP_000938562.1(1), WP_000938574.1(1), WP_000938580.1(1), WP_000938586.1(1), WP_000938588.1(1), WP_000938605.1(1), WP_000938608.1(1), WP_000938621.1(1), WP_000938648.1(1), WP_000938655.1(1), WP_000938657.1(1), WP_000938662.1(1), WP_000938666.1(1), WP_000939335.1(1), WP_000939338.1(1), WP_000939349.1(1), WP_000939354.1(1), WP_000939357.1(1), WP_000939376.1(1), WP_000939379.1(1), WP_000939380.1(1), WP_000939382.1(1), WP_000939385.1(1), WP_000940006.1(1), WP_000940010.1(1), WP_000940011.1(1), WP_000940017.1(1), WP_000940020.1(1), WP_000940021.1(1), WP_000940043.1(1), WP_000940118.1(1), WP_000940597.1(1), WP_000940873.1(1), WP_000940886.1(1), WP_000944242.1(1), WP_000944249.1(1), WP_000944255.1(1), WP_000946660.1(1), WP_000948852.1(1), WP_000948856.1(1), WP_000948858.1(1), WP_000948871.1(1), WP_000948887.1(1), WP_000948892.1(1), WP_000948909.1(1), WP_000950186.1(1), WP_000950199.1(1), WP_000956036.1(1), WP_000956056.1(1), WP_000956059.1(1), WP_000956061.1(1), WP_000956062.1(1), WP_000956073.1(1), WP_000956079.1(1), WP_000956865.1(1), WP_000956869.1(1), WP_000956872.1(1), WP_000956893.1(1), WP_000956900.1(1), WP_000961441.1(1), WP_000961446.1(1), WP_000961483.1(1), WP_000967405.1(1), WP_000980699.1(1), WP_000980722.1(1), WP_000980730.1(1), WP_000980733.1(1), WP_000980737.1(1), WP_000980745.1(1), WP_000980757.1(1), WP_000980758.1(1), WP_000980762.1(1), WP_000981381.1(1), WP_000981384.1(1), WP_000981385.1(1), WP_000982987.1(1), WP_000983004.1(1), WP_000983006.1(1), WP_000983016.1(1), WP_000983017.1(1), WP_000983018.1(1), WP_000983020.1(1), WP_000983029.1(1), WP_000983030.1(1), WP_000983031.1(1), WP_000983702.1(1), WP_000983704.1(1), WP_000983712.1(1), WP_000983720.1(1), WP_000983722.1(1), WP_000983727.1(1), WP_000985482.1(1), WP_000985495.1(1), WP_000985504.1(1), WP_000985507.1(1), WP_000994896.1(1), WP_000994900.1(1), WP_000994908.1(1), WP_000994911.1(1), WP_000995986.1(1), WP_000995989.1(1), WP_000996008.1(1), WP_000996024.1(1), WP_000996025.1(1), WP_000996030.1(1), WP_000996034.1(1), WP_000996094.1(1), WP_000996097.1(1), WP_000996104.1(1), WP_000997380.1(1), WP_000997383.1(1), WP_000997389.1(1), WP_000997399.1(1), WP_000997422.1(1), WP_001013638.1(1), WP_001013640.1(1), WP_001013643.1(1), WP_001013665.1(1), WP_001025299.1(1), WP_001025302.1(1), WP_001025313.1(1), WP_001025321.1(1), WP_001025329.1(1), WP_001025330.1(1), WP_001028468.1(1), WP_001028627.1(1), WP_001028630.1(1), WP_001031062.1(1), WP_001033687.1(1), WP_001033692.1(1), WP_001033701.1(1), WP_001033703.1(1), WP_001035742.1(1), WP_001038407.1(1), WP_001039469.1(1), WP_001039472.1(1), WP_001040182.1(1), WP_001040613.1(1), WP_001040618.1(1), WP_001040619.1(1), WP_001040624.1(1), WP_001040626.1(1), WP_001040629.1(1), WP_001040638.1(1), WP_001040645.1(1), WP_001040649.1(1), WP_001040655.1(1), WP_001040668.1(1), WP_001040685.1(1), WP_001040689.1(1), WP_001040690.1(1), WP_001040691.1(1), WP_001041511.1(1), WP_001043558.1(1), WP_001043562.1(1), WP_001043573.1(1), WP_001043578.1(1), WP_001043583.1(1), WP_001043586.1(1), WP_001043594.1(1), WP_001043595.1(1), WP_001043600.1(1), WP_001043611.1(1), WP_001043613.1(1), WP_001043618.1(1), WP_001044769.1(1), WP_001044770.1(1), WP_001045128.1(1), WP_001045512.1(1), WP_001045518.1(1), WP_001046727.1(1), WP_001057129.1(1), WP_001057996.1(1), WP_001058005.1(1), WP_001061378.1(1), WP_001061379.1(1), WP_001061386.1(1), WP_001061397.1(1), WP_001061408.1(1), WP_001061410.1(1), WP_001061413.1(1), WP_001061416.1(1), WP_001061422.1(1), WP_001065359.1(1), WP_001065364.1(1), WP_001065374.1(1), WP_001065382.1(1), WP_001065385.1(1), WP_001069652.1(1), WP_001069678.1(1), WP_001069692.1(1), WP_001069708.1(1), WP_001069727.1(1), WP_001069732.1(1), WP_001069740.1(1), WP_001069744.1(1), WP_001069787.1(1), WP_001069797.1(1), WP_001070173.1(1), WP_001070181.1(1), WP_001070188.1(1), WP_001070192.1(1), WP_001070196.1(1), WP_001075512.1(1), WP_001075513.1(1), WP_001075537.1(1), WP_001076063.1(1), WP_001084079.1(1), WP_001084080.1(1), WP_001084083.1(1), WP_001084087.1(1), WP_001084108.1(1), WP_001084109.1(1), WP_001084113.1(1), WP_001086500.1(1), WP_001086509.1(1), WP_001086529.1(1), WP_001086530.1(1), WP_001086538.1(1), WP_001086542.1(1), WP_001086544.1(1), WP_001086547.1(1), WP_001090352.1(1), WP_001090354.1(1), WP_001090362.1(1), WP_001090371.1(1), WP_001090372.1(1), WP_001090379.1(1), WP_001090380.1(1), WP_001090383.1(1), WP_001090384.1(1), WP_001090387.1(1), WP_001090392.1(1), WP_001090394.1(1), WP_001090396.1(1), WP_001090398.1(1), WP_001090481.1(1), WP_001090482.1(1), WP_001090491.1(1), WP_001090500.1(1), WP_001090512.1(1), WP_001090519.1(1), WP_001090520.1(1), WP_001090521.1(1), WP_001090524.1(1), WP_001091723.1(1), WP_001095664.1(1), WP_001095665.1(1), WP_001096680.1(1), WP_001096689.1(1), WP_001097125.1(1), WP_001097954.1(1), WP_001100282.1(1), WP_001105436.1(1), WP_001105446.1(1), WP_001105492.1(1), WP_001105508.1(1), WP_001105515.1(1), WP_001107165.1(1), WP_001107166.1(1), WP_001107171.1(1), WP_001107202.1(1), WP_001107639.1(1), WP_001107661.1(1), WP_001107674.1(1), WP_001107678.1(1), WP_001107679.1(1), WP_001107681.1(1), WP_001107698.1(1), WP_001107701.1(1), WP_001107705.1(1), WP_001110571.1(1), WP_001110574.1(1), WP_001110575.1(1), WP_001110580.1(1), WP_001110581.1(1), WP_001110629.1(1), WP_001111198.1(1), WP_001111199.1(1), WP_001113979.1(1), WP_001113980.1(1), WP_001113987.1(1), WP_001113994.1(1), WP_001113998.1(1), WP_001114001.1(1), WP_001114005.1(1), WP_001114010.1(1), WP_001114446.1(1), WP_001114505.1(1), WP_001114527.1(1), WP_001114536.1(1), WP_001114537.1(1), WP_001114549.1(1), WP_001114556.1(1), WP_001116981.1(1), WP_001116988.1(1), WP_001117002.1(1), WP_001117017.1(1), WP_001117018.1(1), WP_001117020.1(1), WP_001117027.1(1), WP_001126770.1(1), WP_001126772.1(1), WP_001126774.1(1), WP_001126794.1(1), WP_001135725.1(1), WP_001135737.1(1), WP_001135743.1(1), WP_001135746.1(1), WP_001136065.1(1), WP_001136200.1(1), WP_001136206.1(1), WP_001136223.1(1), WP_001136240.1(1), WP_001136250.1(1), WP_001138614.1(1), WP_001138622.1(1), WP_001138631.1(1), WP_001138632.1(1), WP_001140647.1(1), WP_001140661.1(1), WP_001142869.1(1), WP_001144205.1(1), WP_001146476.1(1), WP_001146483.1(1), WP_001146491.1(1), WP_001146506.1(1), WP_001146513.1(1), WP_001146514.1(1), WP_001146515.1(1), WP_001146527.1(1), WP_001149683.1(1), WP_001149698.1(1), WP_001149700.1(1), WP_001149709.1(1), WP_001149710.1(1), WP_001149716.1(1), WP_001149717.1(1), WP_001149736.1(1), WP_001149762.1(1), WP_001149767.1(1), WP_001150414.1(1), WP_001150415.1(1), WP_001150429.1(1), WP_001150435.1(1), WP_001150451.1(1), WP_001150453.1(1), WP_001150459.1(1), WP_001150465.1(1), WP_001150470.1(1), WP_001150474.1(1), WP_001150476.1(1), WP_001150488.1(1), WP_001151839.1(1), WP_001151841.1(1), WP_001151843.1(1), WP_001151848.1(1), WP_001151849.1(1), WP_001151852.1(1), WP_001151856.1(1), WP_001151864.1(1), WP_001151865.1(1), WP_001151868.1(1), WP_001151869.1(1), WP_001153728.1(1), WP_001154827.1(1), WP_001157379.1(1), WP_001157381.1(1), WP_001157382.1(1), WP_001157405.1(1), WP_001157409.1(1), WP_001157546.1(1), WP_001157555.1(1), WP_001157887.1(1), WP_001177561.1(1), WP_001178466.1(1), WP_001182411.1(1), WP_001182419.1(1), WP_001182944.1(1), WP_001182945.1(1), WP_001182947.1(1), WP_001182951.1(1), WP_001182958.1(1), WP_001182959.1(1), WP_001182963.1(1), WP_001182966.1(1), WP_001187773.1(1), WP_001187774.1(1), WP_001187787.1(1), WP_001187788.1(1), WP_001187796.1(1), WP_001187802.1(1), WP_001187821.1(1), WP_001187822.1(1), WP_001187871.1(1), WP_001187873.1(1), WP_001187874.1(1), WP_001187875.1(1), WP_001189107.1(1), WP_001189109.1(1), WP_001189112.1(1), WP_001193379.1(1), WP_001193380.1(1), WP_001193381.1(1), WP_001193383.1(1), WP_001193388.1(1), WP_001193399.1(1), WP_001193408.1(1), WP_001194849.1(1), WP_001194852.1(1), WP_001194887.1(1), WP_001194888.1(1), WP_001194891.1(1), WP_001194895.1(1), WP_001194899.1(1), WP_001194905.1(1), WP_001194907.1(1), WP_001194911.1(1), WP_001196179.1(1), WP_001196479.1(1), WP_001196484.1(1), WP_001196495.1(1), WP_001196497.1(1), WP_001198371.1(1), WP_001198382.1(1), WP_001202143.1(1), WP_001202168.1(1), WP_001202172.1(1), WP_001202179.1(1), WP_001202181.1(1), WP_001202183.1(1), WP_001202195.1(1), WP_001202199.1(1), WP_001202202.1(1), WP_001202209.1(1), WP_001202222.1(1), WP_001216363.1(1), WP_001216678.1(1), WP_001218644.1(1), WP_001218650.1(1), WP_001218652.1(1), WP_001218655.1(1), WP_001218660.1(1), WP_001218664.1(1), WP_001219656.1(1), WP_001223129.1(1), WP_001223135.1(1), WP_001223138.1(1), WP_001223158.1(1), WP_001223182.1(1), WP_001223209.1(1), WP_001232878.1(1), WP_001232898.1(1), WP_001232900.1(1), WP_001232902.1(1), WP_001232914.1(1), WP_001232920.1(1), WP_001232926.1(1), WP_001232929.1(1), WP_001232930.1(1), WP_001232939.1(1), WP_001232945.1(1), WP_001232970.1(1), WP_001232971.1(1), WP_001232977.1(1), WP_001232979.1(1), WP_001232981.1(1), WP_001232984.1(1), WP_001234848.1(1), WP_001235581.1(1), WP_001235583.1(1), WP_001235590.1(1), WP_001235593.1(1), WP_001235598.1(1), WP_001235606.1(1), WP_001235610.1(1), WP_001235628.1(1), WP_001235632.1(1), WP_001235643.1(1), WP_001235647.1(1), WP_001235651.1(1), WP_001238187.1(1), WP_001238199.1(1), WP_001238218.1(1), WP_001238233.1(1), WP_001238242.1(1), WP_001238246.1(1), WP_001238251.1(1), WP_001238254.1(1), WP_001238257.1(1), WP_001238259.1(1), WP_001238260.1(1), WP_001238261.1(1), WP_001248144.1(1), WP_001248662.1(1), WP_001248668.1(1), WP_001248678.1(1), WP_001248681.1(1), WP_001248686.1(1), WP_001248701.1(1), WP_001248702.1(1), WP_001248733.1(1), WP_001252649.1(1), WP_001256161.1(1), WP_001256182.1(1), WP_001256187.1(1), WP_001256193.1(1), WP_001256199.1(1), WP_001256204.1(1), WP_001256206.1(1), WP_001256220.1(1), WP_001256227.1(1), WP_001258659.1(1), WP_001258674.1(1), WP_001258681.1(1), WP_001258697.1(1), WP_001259273.1(1), WP_001260691.1(1), WP_001260701.1(1), WP_001260702.1(1), WP_001260703.1(1), WP_001260708.1(1), WP_001260710.1(1), WP_001260711.1(1), WP_001261276.1(1), WP_001261282.1(1), WP_001263484.1(1), WP_001263488.1(1), WP_001263490.1(1), WP_001263499.1(1), WP_001263501.1(1), WP_001264862.1(1), WP_001264879.1(1), WP_001266480.1(1), WP_001266483.1(1), WP_001266485.1(1), WP_001266494.1(1), WP_001266499.1(1), WP_001266508.1(1), WP_001266510.1(1), WP_001272254.1(1), WP_001272881.1(1), WP_001272888.1(1), WP_001272891.1(1), WP_001272894.1(1), WP_001272901.1(1), WP_001272905.1(1), WP_001272914.1(1), WP_001272969.1(1), WP_001272977.1(1), WP_001272986.1(1), WP_001272987.1(1), WP_001273003.1(1), WP_001273007.1(1), WP_001273013.1(1), WP_001275698.1(1), WP_001275705.1(1), WP_001275706.1(1), WP_001279391.1(1), WP_001279393.1(1), WP_001279397.1(1), WP_001279410.1(1), WP_001279411.1(1), WP_001280355.1(1), WP_001280356.1(1), WP_001280365.1(1), WP_001282278.1(1), WP_001282300.1(1), WP_001282348.1(1), WP_001282352.1(1), WP_001282358.1(1), WP_001282363.1(1), WP_001282364.1(1), WP_001282370.1(1), WP_001282372.1(1), WP_001282376.1(1), WP_001283576.1(1), WP_001283577.1(1), WP_001283593.1(1), WP_001285520.1(1), WP_001285534.1(1), WP_001285537.1(1), WP_001285547.1(1), WP_001285549.1(1), WP_001285550.1(1), WP_001285562.1(1), WP_001285563.1(1), WP_001286534.1(1), WP_001286552.1(1), WP_001286554.1(1), WP_001286558.1(1), WP_001286567.1(1), WP_001286570.1(1), WP_001286571.1(1), WP_001286572.1(1), WP_001286660.1(1), WP_001286824.1(1), WP_001286825.1(1), WP_001286827.1(1), WP_001286835.1(1), WP_001286836.1(1), WP_001286861.1(1), WP_001286871.1(1), WP_001286874.1(1), WP_001286877.1(1), WP_001286891.1(1), WP_001287130.1(1), WP_001287162.1(1), WP_001287170.1(1), WP_001288181.1(1), WP_001291203.1(1), WP_001291758.1(1), WP_001291763.1(1), WP_001291775.1(1), WP_001291790.1(1), WP_001291798.1(1), WP_001291808.1(1), WP_001293266.1(1), WP_001293281.1(1), WP_001293287.1(1), WP_001293610.1(1), WP_001293625.1(1), WP_001294041.1(1), WP_001294676.1(1), WP_001294682.1(1), WP_001294689.1(1), WP_001294707.1(1), WP_001294742.1(1), WP_001294767.1(1), WP_001294769.1(1), WP_001294779.1(1), WP_001294782.1(1), WP_001294783.1(1), WP_001294789.1(1), WP_001294791.1(1), WP_001294800.1(1), WP_001294802.1(1), WP_001295063.1(1), WP_001295075.1(1), WP_001295086.1(1), WP_001295520.1(1), WP_001295636.1(1), WP_001295637.1(1), WP_001296743.1(1), WP_001296756.1(1), WP_001296832.1(1), WP_001296860.1(1), WP_001296906.1(1), WP_001296932.1(1), WP_001296940.1(1), WP_001296997.1(1), WP_001297053.1(1), WP_001297074.1(1), WP_001297123.1(1), WP_001297149.1(1), WP_001297267.1(1), WP_001297272.1(1), WP_001297314.1(1), WP_001297349.1(1), WP_001297363.1(1), WP_001297367.1(1), WP_001297438.1(1), WP_001297545.1(1), WP_001297603.1(1), WP_001297684.1(1), WP_001297708.1(1), WP_001297729.1(1), WP_001297753.1(1), WP_001297896.1(1), WP_001297993.1(1), WP_001298165.1(1), WP_001298170.1(1), WP_001298216.1(1), WP_001298248.1(1), WP_001298288.1(1), WP_001298416.1(1), WP_001298470.1(1), WP_001298643.1(1), WP_001298730.1(1), WP_001298883.1(1), WP_001298954.1(1), WP_001299280.1(1), WP_001301490.1(1), WP_001304984.1(1), WP_001305771.1(1), WP_001306323.1(1), WP_001306804.1(1), WP_001307069.1(1), WP_001307737.1(1), WP_001308123.1(1), WP_001308564.1(1), WP_001309796.1(1), WP_001310567.1(1), WP_001311941.1(1), WP_001313766.1(1), WP_001315163.1(1), WP_001315167.1(1), WP_001315935.1(1), WP_001317614.1(1), WP_001317647.1(1), WP_001317661.1(1), WP_001317706.1(1), WP_001317737.1(1), WP_001317742.1(1), WP_001317768.1(1), WP_001317772.1(1), WP_001317871.1(1), WP_001317879.1(1), WP_001317885.1(1), WP_001317982.1(1), WP_001318021.1(1), WP_001318069.1(1), WP_001318090.1(1), WP_001318115.1(1), WP_001318166.1(1), WP_001322100.1(1), WP_001325632.1(1), WP_001326787.1(1), WP_001327855.1(1), WP_001330946.1(1), WP_001331155.1(1), WP_001331399.1(1), WP_001333439.1(1), WP_001333520.1(1), WP_001333542.1(1), WP_001334858.1(1), WP_001334970.1(1), WP_001335079.1(1), WP_001335793.1(1), WP_001335977.1(1), WP_001335990.1(1), WP_001336527.1(1), WP_001337627.1(1), WP_001338997.1(1), WP_001339340.1(1), WP_001339533.1(1), WP_001339595.1(1), WP_001339695.1(1), WP_001339709.1(1), WP_001339919.1(1), WP_001339930.1(1), WP_001339936.1(1), WP_001339962.1(1), WP_001339963.1(1), WP_001340041.1(1), WP_001340073.1(1), WP_001340148.1(1), WP_001340169.1(1), WP_001341289.1(1), WP_001342101.1(1), WP_001342154.1(1), WP_001342204.1(1), WP_001342205.1(1), WP_001343365.1(1), WP_001344570.1(1), WP_001344650.1(1), WP_001344909.1(1), WP_001345114.1(1), WP_001345442.1(1), WP_001345715.1(1), WP_001346794.1(1), WP_001347587.1(1), WP_001350302.1(1), WP_001350855.1(1), WP_001351112.1(1), WP_001352007.1(1), WP_001353240.1(1), WP_001353796.1(1), WP_001354157.1(1), WP_001355473.1(1), WP_001355839.1(1), WP_001356006.1(1), WP_001358360.1(1), WP_001358409.1(1), WP_001361581.1(1), WP_001361937.1(1), WP_001362723.1(1), WP_001363612.1(1), WP_001364833.1(1), WP_001365384.1(1), WP_001365690.1(1), WP_001365783.1(1), WP_001365957.1(1), WP_001366038.1(1), WP_001366260.1(1), WP_001366292.1(1), WP_001366359.1(1), WP_001366375.1(1), WP_001366479.1(1), WP_001366502.1(1), WP_001366519.1(1), WP_001366531.1(1), WP_001366671.1(1), WP_001366690.1(1), WP_001366695.1(1), WP_001366697.1(1), WP_001366767.1(1), WP_001367651.1(1), WP_001367784.1(1), WP_001368657.1(1), WP_001369234.1(1), WP_001372237.1(1), WP_001372254.1(1), WP_001372367.1(1), WP_001372374.1(1), WP_001372426.1(1), WP_001372940.1(1), WP_001373031.1(1), WP_001373235.1(1), WP_001373346.1(1), WP_001374467.1(1), WP_001375219.1(1), WP_001375333.1(1), WP_001375336.1(1), WP_001375501.1(1), WP_001375682.1(1), WP_001375719.1(1), WP_001375840.1(1), WP_001375845.1(1), WP_001375912.1(1), WP_001375963.1(1), WP_001375995.1(1), WP_001376021.1(1), WP_001385246.1(1), WP_001386208.1(1), WP_001386296.1(1), WP_001389162.1(1), WP_001389363.1(1), WP_001389960.1(1), WP_001395333.1(1), WP_001395681.1(1), WP_001396359.1(1), WP_001397970.1(1), WP_001398190.1(1), WP_001399721.1(1), WP_001402653.1(1), WP_001405772.1(1), WP_001407282.1(1), WP_001409257.1(1), WP_001409358.1(1), WP_001409425.1(1), WP_001409605.1(1), WP_001419210.1(1), WP_001420572.1(1), WP_001420630.1(1), WP_001421306.1(1), WP_001422761.1(1), WP_001424813.1(1), WP_001429816.1(1), WP_001431452.1(1), WP_001435129.1(1), WP_001441071.1(1), WP_001441929.1(1), WP_001441993.1(1), WP_001442148.1(1), WP_001442350.1(1), WP_001443086.1(1), WP_001445961.1(1), WP_001446255.1(1), WP_001446663.1(1), WP_001449147.1(1), WP_001459371.1(1), WP_001459597.1(1), WP_001459941.1(1), WP_001459997.1(1), WP_001460448.1(1), WP_001460697.1(1), WP_001461810.1(1), WP_001472887.1(1), WP_001486621.1(1), WP_001488533.1(1), WP_001493612.1(1), WP_001499065.1(1), WP_001505200.1(1), WP_001511865.1(1), WP_001513847.1(1), WP_001519247.1(1), WP_001520915.1(1), WP_001522303.1(1), WP_001529577.1(1), WP_001530084.1(1), WP_001531724.1(1), WP_001532217.1(1), WP_001535049.1(1), WP_001540814.1(1), WP_001541774.1(1), WP_001542635.1(1), WP_001542637.1(1), WP_001542639.1(1), WP_001542755.1(1), WP_001544850.1(1), WP_001544870.1(1), WP_001545794.1(1), WP_001546257.1(1), WP_001551343.1(1), WP_001551359.1(1), WP_001551361.1(1), WP_001554636.1(1), WP_001554923.1(1), WP_001555843.1(1), WP_001556187.1(1), WP_001556191.1(1), WP_001556475.1(1), WP_001557238.1(1), WP_001557301.1(1), WP_001557396.1(1), WP_001558992.1(1), WP_001563647.1(1), WP_001564004.1(1), WP_001567740.1(1), WP_001571666.1(1), WP_001571697.1(1), WP_001571832.1(1), WP_001576746.1(1), WP_001587131.1(1), WP_001600138.1(1), WP_001604679.1(1), WP_001608389.1(1), WP_001609201.1(1), WP_001609223.1(1), WP_001610752.1(1), WP_001610795.1(1), WP_001612508.1(1), WP_001618837.1(1), WP_001624956.1(1), WP_001625492.1(1), WP_001626196.1(1), WP_001673120.1(1), WP_001673147.1(1), WP_001678413.1(1), WP_001679467.1(1), WP_001680032.1(1), WP_001681098.1(1), WP_001682855.1(1), WP_001684139.1(1), WP_001695792.1(1), WP_001696366.1(1), WP_001696384.1(1), WP_001696677.1(1), WP_001696757.1(1), WP_001698168.1(1), WP_001698169.1(1), WP_001698510.1(1), WP_001698511.1(1), WP_001698512.1(1), WP_001704052.1(1), WP_001704176.1(1), WP_001704196.1(1), WP_001716227.1(1), WP_001716241.1(1), WP_001716321.1(1), WP_001717135.1(1), WP_001717767.1(1), WP_001717923.1(1), WP_001741201.1(1), WP_001741269.1(1), WP_001741316.1(1), WP_001741866.1(1), WP_001742578.1(1), WP_001755492.1(1), WP_001762117.1(1), WP_001774374.1(1), WP_003159186.1(1), WP_005025120.1(1), WP_005119537.1(1), WP_005137363.1(1), WP_009008408.1(1), WP_011076353.1(1), WP_011076671.1(1), WP_011579053.1(1), WP_011579093.1(1), WP_011579120.1(1), WP_011579164.1(1), WP_011579219.1(1), WP_011579276.1(1), WP_011579297.1(1), WP_011703683.1(1), WP_012000901.1(1), WP_012000952.1(1), WP_012138442.1(1), WP_012139480.1(1), WP_012139797.1(1), WP_012139840.1(1), WP_012304825.1(1), WP_012304847.1(1), WP_012304850.1(1), WP_012304867.1(1), WP_012304913.1(1), WP_012304929.1(1), WP_012311584.1(1), WP_012311595.1(1), WP_012311652.1(1), WP_012311654.1(1), WP_012311665.1(1), WP_012311674.1(1), WP_012311694.1(1), WP_012311727.1(1), WP_012311760.1(1), WP_012311784.1(1), WP_012311807.1(1), WP_012311824.1(1), WP_012311831.1(1), WP_012311864.1(1), WP_012311878.1(1), WP_012311912.1(1), WP_012311951.1(1), WP_012311980.1(1), WP_012311981.1(1), WP_012449369.1(1), WP_012561166.1(1), WP_012578834.1(1), WP_012578919.1(1), WP_012578955.1(1), WP_012578982.1(1), WP_012578994.1(1), WP_012579005.1(1), WP_012579010.1(1), WP_012579018.1(1), WP_012599966.1(1), WP_012601776.1(1), WP_012602092.1(1), WP_012602171.1(1), WP_012602174.1(1), WP_012602225.1(1), WP_012602287.1(1), WP_012602300.1(1), WP_012602488.1(1), WP_012602538.1(1), WP_012602549.1(1), WP_012602562.1(1), WP_012602588.1(1), WP_012817789.1(1), WP_012817821.1(1), WP_012817833.1(1), WP_012881117.1(1), WP_013362818.1(1), WP_013362819.1(1), WP_014532315.1(1), WP_014639060.1(1), WP_014639064.1(1), WP_014639104.1(1), WP_014639111.1(1), WP_014639112.1(1), WP_014639201.1(1), WP_014639208.1(1), WP_014639243.1(1), WP_014639322.1(1), WP_014639370.1(1), WP_014639377.1(1), WP_014639777.1(1), WP_014640096.1(1), WP_014640167.1(1), WP_014640182.1(1), WP_014640193.1(1), WP_014640203.1(1), WP_014640215.1(1), WP_014640223.1(1), WP_014640531.1(1), WP_014640794.1(1), WP_014640958.1(1), WP_014641252.1(1), WP_014962245.1(1), WP_014962257.1(1), WP_014962275.1(1), WP_014962279.1(1), WP_015056402.1(1), WP_015364389.1(1), WP_015364396.1(1), WP_015364456.1(1), WP_015364521.1(1), WP_015364544.1(1), WP_015439376.1(1), WP_015439380.1(1), WP_015674611.1(1), WP_015674676.1(1), WP_015674703.1(1), WP_015674705.1(1), WP_015675233.1(1), WP_015675335.1(1), WP_015912460.1(1), WP_015912473.1(1), WP_015912562.1(1), WP_015912570.1(1), WP_015912575.1(1), WP_015953132.1(1), WP_015953242.1(1), WP_016232142.1(1), WP_016235501.1(1), WP_016242724.1(1), WP_016243970.1(1), WP_016809719.1(1), WP_021036420.1(1), WP_021036486.1(1), WP_021038053.1(1), WP_021038083.1(1), WP_021038112.1(1), WP_021038182.1(1), WP_021038191.1(1), WP_021038196.1(1), WP_021265674.1(1), WP_021292759.1(1), WP_021292824.1(1), WP_021292868.1(1), WP_021292898.1(1), WP_021292899.1(1), WP_021292907.1(1), WP_021292912.1(1), WP_021293012.1(1), WP_021293075.1(1), WP_021293157.1(1), WP_021293192.1(1), WP_021293388.1(1), WP_021293390.1(1), WP_021512162.1(1), WP_021512407.1(1), WP_021512586.1(1), WP_021513175.1(1), WP_021517176.1(1), WP_021518783.1(1), WP_021520508.1(1), WP_021521389.1(1), WP_021521551.1(1), WP_021521773.1(1), WP_021521860.1(1), WP_021523268.1(1), WP_021524707.1(1), WP_021524783.1(1), WP_021525746.1(1), WP_021527011.1(1), WP_021527170.1(1), WP_021527212.1(1), WP_021531991.1(1), WP_021539241.1(1), WP_021539247.1(1), WP_021539250.1(1), WP_021542178.1(1), WP_021554024.1(1), WP_021556401.1(1), WP_021556405.1(1), WP_021556431.1(1), WP_021556432.1(1), WP_021556436.1(1), WP_021556457.1(1), WP_021556481.1(1), WP_021556532.1(1), WP_021556533.1(1), WP_021556579.1(1), WP_021556582.1(1), WP_021556609.1(1), WP_021556620.1(1), WP_021556644.1(1), WP_021556645.1(1), WP_021556646.1(1), WP_021556654.1(1), WP_021556659.1(1), WP_021556660.1(1), WP_021556704.1(1), WP_021556712.1(1), WP_021556722.1(1), WP_021556726.1(1), WP_021556728.1(1), WP_021556729.1(1), WP_021556738.1(1), WP_021556742.1(1), WP_021556769.1(1), WP_021556807.1(1), WP_021556808.1(1), WP_021556855.1(1), WP_021556867.1(1), WP_021556888.1(1), WP_021557008.1(1), WP_021557069.1(1), WP_021557095.1(1), WP_021557132.1(1), WP_021557136.1(1), WP_021557137.1(1), WP_021557150.1(1), WP_021557151.1(1), WP_021557252.1(1), WP_021557278.1(1), WP_021557284.1(1), WP_021557285.1(1), WP_021557287.1(1), WP_021557317.1(1), WP_021557329.1(1), WP_021557385.1(1), WP_021557401.1(1), WP_021557414.1(1), WP_021557420.1(1), WP_021557446.1(1), WP_021557451.1(1), WP_021557461.1(1), WP_021557471.1(1), WP_021557474.1(1), WP_021557482.1(1), WP_021557493.1(1), WP_021557502.1(1), WP_021557902.1(1), WP_021562535.1(1), WP_021570606.1(1), WP_021570745.1(1), WP_021570768.1(1), WP_021570789.1(1), WP_021570792.1(1), WP_021570890.1(1), WP_021579544.1(1), WP_022645120.1(1), WP_022645397.1(1), WP_022645468.1(1), WP_022645499.1(1), WP_022645878.1(1), WP_022645890.1(1), WP_022645949.1(1), WP_022646018.1(1), WP_022646026.1(1), WP_022646036.1(1), WP_022646209.1(1), WP_022646403.1(1), WP_022646444.1(1), WP_022646474.1(1), WP_023147074.1(1), WP_023147955.1(1), WP_023148089.1(1), WP_023156108.1(1), WP_023156173.1(1), WP_023156258.1(1), WP_023156312.1(1), WP_023156409.1(1), WP_023156468.1(1), WP_023281794.1(1), WP_023281875.1(1), WP_023282139.1(1), WP_023352843.1(1), WP_023566235.1(1), WP_023566682.1(1), WP_023566715.1(1), WP_023567723.1(1), WP_023567988.1(1), WP_023568766.1(1), WP_023908944.1(1), WP_024017797.1(1), WP_024165587.1(1), WP_024165593.1(1), WP_024165618.1(1), WP_024167212.1(1), WP_024173669.1(1), WP_024174136.1(1), WP_024174137.1(1), WP_024174321.1(1), WP_024174322.1(1), WP_024174346.1(1), WP_024175799.1(1), WP_024175814.1(1), WP_024176429.1(1), WP_024177316.1(1), WP_024177860.1(1), WP_024179257.1(1), WP_024179514.1(1), WP_024181807.1(1), WP_024181841.1(1), WP_024181842.1(1), WP_024181843.1(1), WP_024182251.1(1), WP_024184904.1(1), WP_024186195.1(1), WP_024186196.1(1), WP_024186696.1(1), WP_024186772.1(1), WP_024187708.1(1), WP_024188929.1(1), WP_024190479.1(1), WP_024190521.1(1), WP_024192283.1(1), WP_024194549.1(1), WP_024195365.1(1), WP_024196852.1(1), WP_024198363.1(1), WP_024199598.1(1), WP_024199617.1(1), WP_024199697.1(1), WP_024199875.1(1), WP_024201075.1(1), WP_024209722.1(1), WP_024209734.1(1), WP_024209742.1(1), WP_024210271.1(1), WP_024210465.1(1), WP_024210466.1(1), WP_024216406.1(1), WP_024226472.1(1), WP_024226767.1(1), WP_024226883.1(1), WP_024226915.1(1), WP_024227169.1(1), WP_024227171.1(1), WP_024227172.1(1), WP_024227971.1(1), WP_024249999.1(1), WP_024250156.1(1), WP_024250173.1(1), WP_024250423.1(1), WP_024250547.1(1), WP_024250679.1(1), WP_024250682.1(1), WP_024255350.1(1), WP_024259555.1(1), WP_024261659.1(1), WP_024262824.1(1), WP_024561907.1(1), WP_024946589.1(1), WP_024946717.1(1), WP_025263429.1(1), WP_025263476.1(1), WP_025263514.1(1), WP_025297245.1(1), WP_025325175.1(1), WP_029403942.1(1), WP_029487634.1(1), WP_029702196.1(1), WP_029787345.1(1), WP_032106134.1(1), WP_032141048.1(1), WP_032141795.1(1), WP_032141994.1(1), WP_032142000.1(1), WP_032142727.1(1), WP_032142829.1(1), WP_032144724.1(1), WP_032145081.1(1), WP_032147093.1(1), WP_032148612.1(1), WP_032149529.1(1), WP_032150095.1(1), WP_032150958.1(1), WP_032150980.1(1), WP_032151511.1(1), WP_032153325.1(1), WP_032154012.1(1), WP_032159270.1(1), WP_032160888.1(1), WP_032163668.1(1), WP_032167149.1(1), WP_032178322.1(1), WP_032178639.1(1), WP_032179194.1(1), WP_032180501.1(1), WP_032181028.1(1), WP_032181121.1(1), WP_032181124.1(1), WP_032181279.1(1), WP_032186132.1(1), WP_032186826.1(1), WP_032189010.1(1), WP_032195991.1(1), WP_032205826.1(1), WP_032205833.1(1), WP_032205834.1(1), WP_032205864.1(1), WP_032205878.1(1), WP_032205887.1(1), WP_032205914.1(1), WP_032205931.1(1), WP_032205934.1(1), WP_032205943.1(1), WP_032205995.1(1), WP_032206023.1(1), WP_032206091.1(1), WP_032206120.1(1), WP_032206176.1(1), WP_032206177.1(1), WP_032206212.1(1), WP_032206214.1(1), WP_032206267.1(1), WP_032206299.1(1), WP_032206306.1(1), WP_032206322.1(1), WP_032206330.1(1), WP_032206331.1(1), WP_032206352.1(1), WP_032206385.1(1), WP_032206423.1(1), WP_032206471.1(1), WP_032206565.1(1), WP_032206567.1(1), WP_032206573.1(1), WP_032206576.1(1), WP_032206577.1(1), WP_032206584.1(1), WP_032206698.1(1), WP_032206743.1(1), WP_032206757.1(1), WP_032206758.1(1), WP_032206810.1(1), WP_032206818.1(1), WP_032206836.1(1), WP_032206890.1(1), WP_032206935.1(1), WP_032206966.1(1), WP_032206967.1(1), WP_032206979.1(1), WP_032206981.1(1), WP_032206984.1(1), WP_032207020.1(1), WP_032207022.1(1), WP_032207114.1(1), WP_032207216.1(1), WP_032207245.1(1), WP_032207255.1(1), WP_032207257.1(1), WP_032207260.1(1), WP_032207274.1(1), WP_032207279.1(1), WP_032207283.1(1), WP_032207286.1(1), WP_032207297.1(1), WP_032207329.1(1), WP_032207342.1(1), WP_032207392.1(1), WP_032207424.1(1), WP_032207516.1(1), WP_032207521.1(1), WP_032207604.1(1), WP_032207606.1(1), WP_032207608.1(1), WP_032207675.1(1), WP_032207733.1(1), WP_032207789.1(1), WP_032207816.1(1), WP_032207839.1(1), WP_032207881.1(1), WP_032207986.1(1), WP_032208006.1(1), WP_032208018.1(1), WP_032208036.1(1), WP_032208042.1(1), WP_032208066.1(1), WP_032208074.1(1), WP_032208081.1(1), WP_032208097.1(1), WP_032208163.1(1), WP_032208167.1(1), WP_032208171.1(1), WP_032208262.1(1), WP_032208299.1(1), WP_032208301.1(1), WP_032208303.1(1), WP_032208334.1(1), WP_032208367.1(1), WP_032208371.1(1), WP_032208383.1(1), WP_032208391.1(1), WP_032208394.1(1), WP_032208427.1(1), WP_032208507.1(1), WP_032208512.1(1), WP_032208514.1(1), WP_032210662.1(1), WP_032218129.1(1), WP_032218476.1(1), WP_032219279.1(1), WP_032223114.1(1), WP_032223554.1(1), WP_032223636.1(1), WP_032224149.1(1), WP_032229621.1(1), WP_032230672.1(1), WP_032230884.1(1), WP_032238527.1(1), WP_032258358.1(1), WP_032277067.1(1), WP_032285352.1(1), WP_032298182.1(1), WP_032301864.1(1), WP_032317072.1(1), WP_032317267.1(1), WP_032321515.1(1), WP_032329340.1(1), WP_032332725.1(1), WP_033544576.1(1), WP_033544595.1(1), WP_033553495.1(1), WP_033554089.1(1), WP_033554413.1(1), WP_033554618.1(1), WP_033560329.1(1), WP_033813638.1(1), WP_038427076.1(1), WP_038427153.1(1), WP_038427213.1(1), WP_038427220.1(1), WP_038427337.1(1), WP_038430503.1(1), WP_038430898.1(1), WP_038430976.1(1), WP_038431055.1(1), WP_038431158.1(1), WP_038431976.1(1), WP_038432291.1(1), WP_039023115.1(1), WP_039023140.1(1), WP_039023207.1(1), WP_039023208.1(1), WP_039025711.1(1), WP_039060284.1(1), WP_039102377.1(1), WP_039102540.1(1), WP_039102734.1(1), WP_039110121.1(1), WP_039258853.1(1), WP_039264327.1(1), WP_039264332.1(1), WP_039264372.1(1), WP_039264387.1(1), WP_039264390.1(1), WP_039264421.1(1), WP_039264434.1(1), WP_039264441.1(1), WP_039264447.1(1), WP_039264454.1(1), WP_039264545.1(1), WP_039264547.1(1), WP_039264553.1(1), WP_039264562.1(1), WP_039264563.1(1), WP_039264564.1(1), WP_039264569.1(1), WP_039267619.1(1), WP_039267665.1(1), WP_039267667.1(1), WP_039267669.1(1), WP_039267701.1(1), WP_039267708.1(1), WP_039267712.1(1), WP_039267713.1(1), WP_039267714.1(1), WP_039267756.1(1), WP_039267767.1(1), WP_039267774.1(1), WP_039267780.1(1), WP_039267783.1(1), WP_039267791.1(1), WP_039267798.1(1), WP_039267803.1(1), WP_039267808.1(1), WP_039267809.1(1), WP_039267829.1(1), WP_039267830.1(1), WP_039267872.1(1), WP_039267876.1(1), WP_039267878.1(1), WP_039267905.1(1), WP_039267908.1(1), WP_039267956.1(1), WP_039267996.1(1), WP_039268320.1(1), WP_039268322.1(1), WP_039268358.1(1), WP_039268474.1(1), WP_039268488.1(1), WP_039268535.1(1), WP_039722367.1(1), WP_039722375.1(1), WP_039722386.1(1), WP_040073344.1(1), WP_040110426.1(1), WP_040111971.1(1), WP_040116957.1(1), WP_040116959.1(1), WP_040123396.1(1), WP_040234484.1(1), WP_040234503.1(1), WP_040234586.1(1), WP_040234616.1(1), WP_040234621.1(1), WP_040234633.1(1), WP_040234675.1(1), WP_040234726.1(1), WP_040234815.1(1), WP_040234831.1(1), WP_040234897.1(1), WP_040234913.1(1), WP_040234924.1(1), WP_040234961.1(1), WP_040234964.1(1), WP_040235002.1(1), WP_040235177.1(1), WP_040235217.1(1), WP_040235293.1(1), WP_040235329.1(1), WP_041031393.1(1), WP_041031824.1(1), WP_041032008.1(1), WP_041032045.1(1), WP_041124050.1(1), WP_041124079.1(1), WP_041124095.1(1), WP_041124127.1(1), WP_041124158.1(1), WP_041124159.1(1), WP_041124162.1(1), WP_041124187.1(1), WP_041124217.1(1), WP_041329576.1(1), WP_041520829.1(1), WP_041520854.1(1), WP_041520987.1(1), WP_041521556.1(1), WP_042094944.1(1), WP_042634304.1(1), WP_042815301.1(1), WP_042815419.1(1), WP_042815603.1(1), WP_042815619.1(1), WP_042815954.1(1), WP_043952045.1(1), WP_044060750.1(1), WP_044502161.1(1), WP_044502175.1(1), WP_044502184.1(1), WP_044502193.1(1), WP_044502197.1(1), WP_044502200.1(1), WP_044502212.1(1), WP_044502218.1(1), WP_044502239.1(1), WP_044502242.1(1), WP_044502287.1(1), WP_044502315.1(1), WP_044502342.1(1), WP_044502361.1(1), WP_044502363.1(1), WP_044502367.1(1), WP_044502428.1(1), WP_046072809.1(1), WP_046072829.1(1), WP_046072841.1(1), WP_046072842.1(1), WP_046072861.1(1), WP_046072887.1(1), WP_046072891.1(1), WP_046072921.1(1), WP_046201411.1(1), WP_046201448.1(1), WP_046201471.1(1), WP_046201485.1(1), WP_046201511.1(1), WP_046201514.1(1), WP_046201552.1(1), WP_046201556.1(1), WP_046201560.1(1), WP_046201563.1(1), WP_046201578.1(1), WP_046201587.1(1), WP_046201589.1(1), WP_046201652.1(1), WP_046201654.1(1), WP_046201659.1(1), WP_046201700.1(1), WP_046201708.1(1), WP_046201751.1(1), WP_046608383.1(1), WP_046609272.1(1), WP_046613289.1(1), WP_046613291.1(1), WP_046613332.1(1), WP_046613442.1(1), WP_046880201.1(1), WP_046880203.1(1), WP_046880317.1(1), WP_046880539.1(1), WP_046880735.1(1), WP_046880977.1(1), WP_046880993.1(1), WP_046881013.1(1), WP_046881019.1(1), WP_046881028.1(1), WP_046881060.1(1), WP_046881113.1(1), WP_046881137.1(1), WP_046881142.1(1), WP_046881148.1(1), WP_046881159.1(1), WP_046881182.1(1), WP_046881283.1(1), WP_046881347.1(1), WP_046881598.1(1), WP_046881633.1(1), WP_047928495.1(1), WP_047928535.1(1), WP_047928536.1(1), WP_047928556.1(1), WP_047928618.1(1), WP_047928643.1(1), WP_047928689.1(1), WP_047928757.1(1), WP_047928792.1(1), WP_047928794.1(1), WP_047928815.1(1), WP_047928817.1(1), WP_047928878.1(1), WP_047928905.1(1), WP_047928928.1(1), WP_047928942.1(1), WP_047928950.1(1), WP_047928999.1(1), WP_047929003.1(1), WP_047938481.1(1), WP_048657307.1(1), WP_048816317.1(1), WP_048943274.1(1), WP_048943276.1(1), WP_048943318.1(1), WP_048943343.1(1), WP_048955515.1(1), WP_048959262.1(1), WP_049789690.1(1), WP_049828166.1(1), WP_050575545.1(1), WP_050576437.1(1), WP_051539320.1(1), WP_051763866.1(1), WP_051763867.1(1), WP_052292772.1(1), WP_052463146.1(1), WP_052476159.1(1), WP_052734130.1(1), WP_052906020.1(1), WP_053811553.1(1), WP_053811558.1(1), WP_053811573.1(1), WP_053811580.1(1), WP_057915969.1(1), WP_057915971.1(1), WP_058127826.1(1), WP_058127852.1(1), WP_058677881.1(1), WP_059218012.1(1), WP_059219410.1(1), WP_059219411.1(1), WP_059219430.1(1), WP_059219438.1(1), WP_059309176.1(1), WP_059309182.1(1), WP_059309184.1(1), WP_059309191.1(1), WP_059309202.1(1), WP_059309203.1(1), WP_059309242.1(1), WP_060552817.1(1), WP_060552819.1(1), WP_060565021.1(1), WP_060565034.1(1), WP_060565044.1(1), WP_060565070.1(1), WP_060565087.1(1), WP_060565110.1(1), WP_060565116.1(1), WP_060565172.1(1), WP_060565184.1(1), WP_060667429.1(1), WP_060667431.1(1), WP_060682020.1(1), WP_060682073.1(1), WP_060684344.1(1), WP_060684361.1(1), WP_060684379.1(1), WP_060684404.1(1), WP_060684409.1(1), WP_060684423.1(1), WP_060684434.1(1), WP_060684458.1(1), WP_060684491.1(1), WP_060684498.1(1), WP_060684499.1(1), WP_060684503.1(1), WP_060684508.1(1), WP_060684526.1(1), WP_060684527.1(1), WP_060703578.1(1), WP_060703587.1(1), WP_060710358.1(1), WP_060722659.1(1), WP_060773110.1(1), WP_060773130.1(1), WP_060773132.1(1), WP_060773135.1(1), WP_060773139.1(1), WP_060773141.1(1), WP_060773142.1(1), WP_060773143.1(1), WP_060773147.1(1), WP_060773162.1(1), WP_060773177.1(1), WP_060773199.1(1), WP_060773216.1(1), WP_060773254.1(1), WP_060773260.1(1), WP_060773261.1(1), WP_060773262.1(1), WP_060773269.1(1), WP_060773275.1(1), WP_060773307.1(1), WP_060773313.1(1), WP_060773315.1(1), WP_060773328.1(1), WP_060773336.1(1), WP_060773363.1(1), WP_060773388.1(1), WP_060773394.1(1), WP_060773395.1(1), WP_060773407.1(1), WP_060773408.1(1), WP_060773413.1(1), WP_060773464.1(1), WP_060773478.1(1), WP_060773514.1(1), WP_060773540.1(1), WP_060773580.1(1), WP_060773585.1(1), WP_060773600.1(1), WP_060773607.1(1), WP_060773632.1(1), WP_060773636.1(1), WP_060773671.1(1), WP_060773682.1(1), WP_060773701.1(1), WP_060773703.1(1), WP_060773709.1(1), WP_060773715.1(1), WP_060773739.1(1), WP_060773746.1(1), WP_060773793.1(1), WP_060773794.1(1), WP_060773795.1(1), WP_060773835.1(1), WP_060773840.1(1), WP_060773845.1(1), WP_060773862.1(1), WP_060773865.1(1), WP_060773872.1(1), WP_060773890.1(1), WP_060773912.1(1), WP_060773918.1(1), WP_060773948.1(1), WP_060773976.1(1), WP_060774000.1(1), WP_060774035.1(1), WP_060774050.1(1), WP_060774052.1(1), WP_060774063.1(1), WP_060774079.1(1), WP_060774081.1(1), WP_060774082.1(1), WP_060774084.1(1), WP_060774100.1(1), WP_060774103.1(1), WP_061069248.1(1), WP_061069254.1(1), WP_061069264.1(1), WP_061069274.1(1), WP_061069281.1(1), WP_061092168.1(1), WP_061132350.1(1), WP_061157875.1(1), WP_061157880.1(1), WP_061157956.1(1), WP_061158021.1(1), WP_061158022.1(1), WP_061158023.1(1), WP_061158038.1(1), WP_061158053.1(1), WP_061158054.1(1), WP_061186089.1(1), WP_061186093.1(1), WP_061856949.1(1), WP_062914682.1(1), WP_062914702.1(1), WP_062914706.1(1), WP_062914710.1(1), WP_062946162.1(1), WP_062946172.1(1), WP_062946177.1(1), WP_062946184.1(1), WP_062946189.1(1), WP_062946201.1(1), WP_063112458.1(1), WP_063112463.1(1), WP_063112483.1(1), WP_063112486.1(1), WP_063131858.1(1), WP_063131879.1(1), WP_063131921.1(1), WP_063131928.1(1), WP_063131936.1(1), YP_001165331.1(1), YP_002389867.1(1), YP_002389873.1(1), YP_002389876.1(1), YP_002389905.1(1), YP_002389910.1(1), YP_002389911.1(1), YP_002389920.1(1), YP_002389923.1(1), YP_002389926.1(1), YP_002389947.1(1), YP_002389964.1(1), YP_002389974.1(1), YP_002389983.1(1), YP_002389999.1(1), YP_002390000.1(1), YP_002390003.1(1), YP_002390004.1(1), YP_002390007.1(1), YP_002390016.1(1), YP_002390023.1(1), YP_002390024.1(1), YP_002390026.1(1), YP_002390037.1(1), YP_002390038.1(1), YP_002390042.1(1), YP_002390046.1(1), YP_002390049.1(1), YP_002390054.1(1), YP_002390063.1(1), YP_002390064.1(1), YP_002390102.1(1), YP_002390104.1(1), YP_002390105.1(1), YP_002390111.1(1), YP_002390157.1(1), YP_002390163.1(1), YP_002390165.1(1), YP_002390192.1(1), YP_002390227.1(1), YP_002390228.1(1), YP_002390236.1(1), YP_002390238.1(1), YP_002390245.1(1), YP_002390259.1(1), YP_002390268.1(1), YP_002390271.1(1), YP_002390272.1(1), YP_002390297.1(1), YP_002390306.1(1), YP_002390320.1(1), YP_002390325.1(1), YP_002390350.1(1), YP_002390374.1(1), YP_002390388.1(1), YP_002390435.1(1), YP_002390442.1(1), YP_002390476.1(1), YP_002390493.1(1), YP_002390496.1(1), YP_002390521.1(1), YP_002390547.1(1), YP_002390579.1(1), YP_002390584.1(1), YP_002390589.1(1), YP_002390597.1(1), YP_002390612.1(1), YP_002390615.1(1), YP_002390617.1(1), YP_002390627.1(1), YP_002390638.1(1), YP_002390646.1(1), YP_002390672.1(1), YP_002390676.1(1), YP_002390681.1(1), YP_002390696.1(1), YP_002390697.1(1), YP_002390707.1(1), YP_002390708.1(1), YP_002390714.1(1), YP_002390729.1(1), YP_002390732.1(1), YP_002390735.1(1), YP_002390739.1(1), YP_002390748.1(1), YP_002390751.1(1), YP_002390759.1(1), YP_002390760.1(1), YP_002390792.1(1), YP_002390794.1(1), YP_002390813.1(1), YP_002390839.1(1), YP_002390879.1(1), YP_002390880.1(1), YP_002390883.1(1), YP_002390897.1(1), YP_002390908.1(1), YP_002390911.1(1), YP_002390920.1(1), YP_002390928.1(1), YP_002390930.1(1), YP_002390994.1(1), YP_002391035.1(1), YP_002391036.1(1), YP_002391045.1(1), YP_002391046.1(1), YP_002391052.1(1), YP_002391053.1(1), YP_002391058.1(1), YP_002391073.1(1), YP_002391087.1(1), YP_002391088.1(1), YP_002391165.1(1), YP_002391167.1(1), YP_002391179.1(1), YP_002391190.1(1), YP_002391198.1(1), YP_002391199.1(1), YP_002391218.1(1), YP_002391241.1(1), YP_002391243.1(1), YP_002391244.1(1), YP_002391262.1(1), YP_002391284.1(1), YP_002391324.1(1), YP_002391421.1(1), YP_002391436.1(1), YP_002391464.1(1), YP_002391492.1(1), YP_002391496.1(1), YP_002391497.1(1), YP_002391499.1(1), YP_002391500.1(1), YP_002391501.1(1), YP_002391537.1(1), YP_002391567.1(1), YP_002391579.1(1), YP_002391583.1(1), YP_002391584.1(1), YP_002391598.1(1), YP_002391607.1(1), YP_002391611.1(1), YP_002391621.1(1), YP_002391636.1(1), YP_002391644.1(1), YP_002391654.1(1), YP_002391661.1(1), YP_002391673.1(1), YP_002391688.1(1), YP_002391711.1(1), YP_002391745.1(1), YP_002391746.1(1), YP_002391805.1(1), YP_002391814.1(1), YP_002391819.1(1), YP_002391820.1(1), YP_002391946.1(1), YP_002391961.1(1), YP_002391971.1(1), YP_002391978.1(1), YP_002391980.1(1), YP_002392014.1(1), YP_002392017.1(1), YP_002392018.1(1), YP_002392019.1(1), YP_002392031.1(1), YP_002392042.1(1), YP_002392084.1(1), YP_002392098.1(1), YP_002392132.1(1), YP_002392144.1(1), YP_002392156.1(1), YP_002392178.1(1), YP_002392264.1(1), YP_002392282.1(1), YP_002392323.1(1), YP_002392337.1(1), YP_002392344.1(1), YP_002392346.1(1), YP_002392350.1(1), YP_002392353.1(1), YP_002392371.1(1), YP_002392374.1(1), YP_002392380.1(1), YP_002392392.1(1), YP_002392400.1(1), YP_002392401.1(1), YP_002392403.1(1), YP_002392409.1(1), YP_002392410.1(1), YP_002392415.1(1), YP_002392428.1(1), YP_002392439.1(1), YP_002392440.1(1), YP_002392441.1(1), YP_002392442.1(1), YP_002392443.1(1), YP_002392450.1(1), YP_002392452.1(1), YP_002392540.1(1), YP_002392582.1(1), YP_002392591.1(1), YP_002392597.1(1), YP_002392598.1(1), YP_002392613.1(1), YP_002392637.1(1), YP_002392649.1(1), YP_002392651.1(1), YP_002392657.1(1), YP_002392660.1(1), YP_002392683.1(1), YP_002392684.1(1), YP_002392686.1(1), YP_002392692.1(1), YP_002392707.1(1), YP_002392741.1(1), YP_002392744.1(1), YP_002392796.1(1), YP_002392797.1(1), YP_002392798.1(1), YP_002392806.1(1), YP_002392832.1(1), YP_002392849.1(1), YP_002392850.1(1), YP_002392940.1(1), YP_002393026.1(1), YP_002393056.1(1), YP_002393065.1(1), YP_002393066.1(1), YP_002393067.1(1), YP_002393073.1(1), YP_002393107.1(1), YP_002393147.1(1), YP_002393149.1(1), YP_002393150.1(1), YP_002393151.1(1), YP_002393153.1(1), YP_002393154.1(1), YP_002393158.1(1), YP_002393162.1(1), YP_002393163.1(1), YP_002393166.1(1), YP_002393168.1(1), YP_002393169.1(1), YP_002393178.1(1), YP_002393184.1(1), YP_002393185.1(1), YP_002393205.1(1), YP_002393206.1(1), YP_002393221.1(1), YP_002393235.1(1), YP_002393242.1(1), YP_002393253.1(1), YP_002393257.1(1), YP_002393262.1(1), YP_002393263.1(1), YP_002393269.1(1), YP_002393270.1(1), YP_002393271.1(1), YP_002393272.1(1), YP_002393273.1(1), YP_002393274.1(1), YP_002393275.1(1), YP_002393276.1(1), YP_002393277.1(1), YP_002393278.1(1), YP_002393279.1(1), YP_002393280.1(1), YP_002393281.1(1), YP_002393282.1(1), YP_002393283.1(1), YP_002393284.1(1), YP_002393285.1(1), YP_002393286.1(1), YP_002393287.1(1), YP_002393288.1(1), YP_002393289.1(1), YP_002393290.1(1), YP_002393291.1(1), YP_002393292.1(1), YP_002393293.1(1), YP_002393294.1(1), YP_002393295.1(1), YP_002393313.1(1), YP_002393314.1(1), YP_002393315.1(1), YP_002393316.1(1), YP_002393327.1(1), YP_002393354.1(1), YP_002393370.1(1), YP_002393378.1(1), YP_002393390.1(1), YP_002393391.1(1), YP_002393425.1(1), YP_002393429.1(1), YP_002393430.1(1), YP_002393444.1(1), YP_002393445.1(1), YP_002393460.1(1), YP_002393461.1(1), YP_002393471.1(1), YP_002393481.1(1), YP_002393495.1(1), YP_002393526.1(1), YP_002393527.1(1), YP_002393528.1(1), YP_002393531.1(1), YP_002393532.1(1), YP_002393545.1(1), YP_002393546.1(1), YP_002393547.1(1), YP_002393548.1(1), YP_002393556.1(1), YP_002393578.1(1), YP_002393579.1(1), YP_002393594.1(1), YP_002393620.1(1), YP_002393621.1(1), YP_002393622.1(1), YP_002393628.1(1), YP_002393634.1(1), YP_002393636.1(1), YP_002393637.1(1), YP_002393648.1(1), YP_002393666.1(1), YP_002393691.1(1), YP_002393692.1(1), YP_002393695.1(1), YP_002393696.1(1), YP_002393711.1(1), YP_002393713.1(1), YP_002393728.1(1), YP_002393729.1(1), YP_002393737.1(1), YP_002393747.1(1), YP_002393759.1(1), YP_002393762.1(1), YP_002393793.1(1), YP_002393831.1(1), YP_002393849.1(1), YP_002393852.1(1), YP_002393859.1(1), YP_002393870.1(1), YP_002393915.1(1), YP_002393916.1(1), YP_002393953.1(1), YP_002393959.1(1), YP_002393960.1(1), YP_002393962.1(1), YP_002393963.1(1), YP_002393964.1(1), YP_002393965.1(1), YP_002393966.1(1), YP_002393967.1(1), YP_002393974.1(1), YP_002394005.1(1), YP_002394019.1(1), YP_002394030.1(1), YP_002394054.1(1), YP_002394077.1(1), YP_002394078.1(1), YP_002394090.1(1), YP_002394099.1(1), YP_002394100.1(1), YP_002394108.1(1), YP_002394130.1(1), YP_002394160.1(1), YP_002394161.1(1), YP_002394230.1(1), YP_002394236.1(1), YP_002394237.1(1), YP_002394239.1(1), YP_002394243.1(1), YP_002394244.1(1), YP_002394245.1(1), YP_002394251.1(1), YP_002394252.1(1), YP_002394272.1(1), YP_002394274.1(1), YP_002394275.1(1), YP_002394302.1(1), YP_002394316.1(1), YP_002394320.1(1), YP_002394329.1(1), YP_002394343.1(1), YP_002394376.1(1), YP_002394421.1(1), YP_002394435.1(1), YP_002394479.1(1), YP_002394482.1(1), YP_002394483.1(1), YP_002394488.1(1), YP_002394518.1(1), YP_002394537.1(1), YP_002394548.1(1), YP_002401027.1(1), YP_002401094.1(1), YP_002401101.1(1), YP_002401113.1(1), YP_002401121.1(1), YP_002401136.1(1), YP_002406073.1(1), YP_002406075.1(1), YP_002406102.1(1), YP_002406109.1(1), YP_002406110.1(1), YP_002406119.1(1), YP_002406123.1(1), YP_002406125.1(1), YP_002406146.1(1), YP_002406167.1(1), YP_002406176.1(1), YP_002406193.1(1), YP_002406194.1(1), YP_002406197.1(1), YP_002406198.1(1), YP_002406201.1(1), YP_002406210.1(1), YP_002406217.1(1), YP_002406218.1(1), YP_002406221.1(1), YP_002406232.1(1), YP_002406233.1(1), YP_002406237.1(1), YP_002406243.1(1), YP_002406268.1(1), YP_002406269.1(1), YP_002406272.1(1), YP_002406281.1(1), YP_002406297.1(1), YP_002406304.1(1), YP_002406306.1(1), YP_002406318.1(1), YP_002406319.1(1), YP_002406357.1(1), YP_002406398.1(1), YP_002406401.1(1), YP_002406407.1(1), YP_002406432.1(1), YP_002406443.1(1), YP_002406449.1(1), YP_002406450.1(1), YP_002406467.1(1), YP_002406468.1(1), YP_002406477.1(1), YP_002406482.1(1), YP_002406484.1(1), YP_002406488.1(1), YP_002406495.1(1), YP_002406522.1(1), YP_002406591.1(1), YP_002406625.1(1), YP_002406643.1(1), YP_002406646.1(1), YP_002406663.1(1), YP_002406692.1(1), YP_002406713.1(1), YP_002406754.1(1), YP_002406759.1(1), YP_002406765.1(1), YP_002406769.1(1), YP_002406780.1(1), YP_002406795.1(1), YP_002406798.1(1), YP_002406801.1(1), YP_002406812.1(1), YP_002406822.1(1), YP_002406831.1(1), YP_002406860.1(1), YP_002406864.1(1), YP_002406869.1(1), YP_002406890.1(1), YP_002406896.1(1), YP_002406924.1(1), YP_002407015.1(1), YP_002407016.1(1), YP_002407021.1(1), YP_002407028.1(1), YP_002407052.1(1), YP_002407090.1(1), YP_002407091.1(1), YP_002407127.1(1), YP_002407148.1(1), YP_002407163.1(1), YP_002407174.1(1), YP_002407181.1(1), YP_002407191.1(1), YP_002407199.1(1), YP_002407214.1(1), YP_002407223.1(1), YP_002407227.1(1), YP_002407237.1(1), YP_002407252.1(1), YP_002407253.1(1), YP_002407257.1(1), YP_002407274.1(1), YP_002407306.1(1), YP_002407343.1(1), YP_002407344.1(1), YP_002407345.1(1), YP_002407347.1(1), YP_002407348.1(1), YP_002407352.1(1), YP_002407382.1(1), YP_002407408.1(1), YP_002407409.1(1), YP_002407423.1(1), YP_002407487.1(1), YP_002407501.1(1), YP_002407502.1(1), YP_002407507.1(1), YP_002407519.1(1), YP_002407529.1(1), YP_002407530.1(1), YP_002407539.1(1), YP_002407540.1(1), YP_002407546.1(1), YP_002407547.1(1), YP_002407552.1(1), YP_002407553.1(1), YP_002407567.1(1), YP_002407581.1(1), YP_002407582.1(1), YP_002407603.1(1), YP_002407605.1(1), YP_002407617.1(1), YP_002407628.1(1), YP_002407636.1(1), YP_002407641.1(1), YP_002407642.1(1), YP_002407670.1(1), YP_002407690.1(1), YP_002407692.1(1), YP_002407693.1(1), YP_002407701.1(1), YP_002407716.1(1), YP_002407729.1(1), YP_002407742.1(1), YP_002407743.1(1), YP_002407754.1(1), YP_002407768.1(1), YP_002407856.1(1), YP_002407895.1(1), YP_002407971.1(1), YP_002407973.1(1), YP_002407999.1(1), YP_002408001.1(1), YP_002408010.1(1), YP_002408013.1(1), YP_002408024.1(1), YP_002408037.1(1), YP_002408040.1(1), YP_002408041.1(1), YP_002408081.1(1), YP_002408108.1(1), YP_002408127.1(1), YP_002408128.1(1), YP_002408161.1(1), YP_002408162.1(1), YP_002408177.1(1), YP_002408180.1(1), YP_002408189.1(1), YP_002408193.1(1), YP_002408196.1(1), YP_002408199.1(1), YP_002408218.1(1), YP_002408226.1(1), YP_002408227.1(1), YP_002408234.1(1), YP_002408235.1(1), YP_002408241.1(1), YP_002408248.1(1), YP_002408250.1(1), YP_002408281.1(1), YP_002408284.1(1), YP_002408285.1(1), YP_002408286.1(1), YP_002408299.1(1), YP_002408308.1(1), YP_002408358.1(1), YP_002408372.1(1), YP_002408411.1(1), YP_002408423.1(1), YP_002408435.1(1), YP_002408496.1(1), YP_002408517.1(1), YP_002408561.1(1), YP_002408586.1(1), YP_002408593.1(1), YP_002408641.1(1), YP_002408643.1(1), YP_002408645.1(1), YP_002408650.1(1), YP_002408653.1(1), YP_002408671.1(1), YP_002408674.1(1), YP_002408688.1(1), YP_002408701.1(1), YP_002408709.1(1), YP_002408710.1(1), YP_002408712.1(1), YP_002408718.1(1), YP_002408719.1(1), YP_002408725.1(1), YP_002408735.1(1), YP_002408747.1(1), YP_002408748.1(1), YP_002408749.1(1), YP_002408750.1(1), YP_002408751.1(1), YP_002408758.1(1), YP_002408760.1(1), YP_002408801.1(1), YP_002408815.1(1), YP_002408816.1(1), YP_002408831.1(1), YP_002408855.1(1), YP_002408863.1(1), YP_002408865.1(1), YP_002408871.1(1), YP_002408874.1(1), YP_002408888.1(1), YP_002408933.1(1), YP_002408936.1(1), YP_002408948.1(1), YP_002408954.1(1), YP_002408988.1(1), YP_002408989.1(1), YP_002409042.1(1), YP_002409059.1(1), YP_002409066.1(1), YP_002409069.1(1), YP_002409089.1(1), YP_002409114.1(1), YP_002409119.1(1), YP_002409122.1(1), YP_002409127.1(1), YP_002409128.1(1), YP_002409130.1(1), YP_002409137.1(1), YP_002409152.1(1), YP_002409162.1(1), YP_002409228.1(1), YP_002409229.1(1), YP_002409230.1(1), YP_002409238.1(1), YP_002409267.1(1), YP_002409284.1(1), YP_002409285.1(1), YP_002409323.1(1), YP_002409342.1(1), YP_002409355.1(1), YP_002409422.1(1), YP_002409423.1(1), YP_002409456.1(1), YP_002409465.1(1), YP_002409466.1(1), YP_002409467.1(1), YP_002409476.1(1), YP_002409517.1(1), YP_002409562.1(1), YP_002409564.1(1), YP_002409565.1(1), YP_002409566.1(1), YP_002409568.1(1), YP_002409569.1(1), YP_002409573.1(1), YP_002409577.1(1), YP_002409578.1(1), YP_002409581.1(1), YP_002409583.1(1), YP_002409584.1(1), YP_002409593.1(1), YP_002409599.1(1), YP_002409600.1(1), YP_002409623.1(1), YP_002409624.1(1), YP_002409642.1(1), YP_002409654.1(1), YP_002409662.1(1), YP_002409672.1(1), YP_002409675.1(1), YP_002409679.1(1), YP_002409680.1(1), YP_002409686.1(1), YP_002409687.1(1), YP_002409688.1(1), YP_002409689.1(1), YP_002409690.1(1), YP_002409691.1(1), YP_002409692.1(1), YP_002409693.1(1), YP_002409694.1(1), YP_002409695.1(1), YP_002409696.1(1), YP_002409697.1(1), YP_002409698.1(1), YP_002409699.1(1), YP_002409700.1(1), YP_002409701.1(1), YP_002409702.1(1), YP_002409703.1(1), YP_002409704.1(1), YP_002409705.1(1), YP_002409706.1(1), YP_002409707.1(1), YP_002409708.1(1), YP_002409709.1(1), YP_002409710.1(1), YP_002409711.1(1), YP_002409712.1(1), YP_002409716.1(1), YP_002409717.1(1), YP_002409718.1(1), YP_002409719.1(1), YP_002409729.1(1), YP_002409756.1(1), YP_002409772.1(1), YP_002409781.1(1), YP_002409793.1(1), YP_002409794.1(1), YP_002409795.1(1), YP_002409810.1(1), YP_002409823.1(1), YP_002409828.1(1), YP_002409829.1(1), YP_002409837.1(1), YP_002409838.1(1), YP_002409853.1(1), YP_002409854.1(1), YP_002409865.1(1), YP_002409881.1(1), YP_002409901.1(1), YP_002409935.1(1), YP_002409936.1(1), YP_002409949.1(1), YP_002409950.1(1), YP_002409953.1(1), YP_002409954.1(1), YP_002409962.1(1), YP_002409989.1(1), YP_002409990.1(1), YP_002410004.1(1), YP_002410030.1(1), YP_002410031.1(1), YP_002410032.1(1), YP_002410037.1(1), YP_002410039.1(1), YP_002410047.1(1), YP_002410049.1(1), YP_002410050.1(1), YP_002410061.1(1), YP_002410075.1(1), YP_002410135.1(1), YP_002410148.1(1), YP_002410150.1(1), YP_002410181.1(1), YP_002410182.1(1), YP_002410185.1(1), YP_002410186.1(1), YP_002410200.1(1), YP_002410219.1(1), YP_002410220.1(1), YP_002410229.1(1), YP_002410239.1(1), YP_002410240.1(1), YP_002410242.1(1), YP_002410243.1(1), YP_002410244.1(1), YP_002410245.1(1), YP_002410246.1(1), YP_002410247.1(1), YP_002410259.1(1), YP_002410293.1(1), YP_002410329.1(1), YP_002410342.1(1), YP_002410352.1(1), YP_002410382.1(1), YP_002410391.1(1), YP_002410392.1(1), YP_002410400.1(1), YP_002410423.1(1), YP_002410462.1(1), YP_002410486.1(1), YP_002410490.1(1), YP_002410491.1(1), YP_002410493.1(1), YP_002410497.1(1), YP_002410498.1(1), YP_002410499.1(1), YP_002410505.1(1), YP_002410506.1(1), YP_002410526.1(1), YP_002410527.1(1), YP_002410528.1(1), YP_002410555.1(1), YP_002410556.1(1), YP_002410559.1(1), YP_002410576.1(1), YP_002410590.1(1), YP_002410622.1(1), YP_002410667.1(1), YP_002410669.1(1), YP_002410670.1(1), YP_002410713.1(1), YP_002410747.1(1), YP_002410750.1(1), YP_002410769.1(1), YP_002410780.1(1), YP_002791247.1(1), YP_002791248.1(1), YP_002791255.1(1), YP_002791256.1(1), YP_002791257.1(1), YP_006118382.1(1), YP_006118388.1(1), YP_006118390.1(1), YP_006118420.1(1), YP_006118425.1(1), YP_006118426.1(1), YP_006118435.1(1), YP_006118438.1(1), YP_006118441.1(1), YP_006118461.1(1), YP_006118478.1(1), YP_006118480.1(1), YP_006118489.1(1), YP_006118498.1(1), YP_006118514.1(1), YP_006118515.1(1), YP_006118518.1(1), YP_006118519.1(1), YP_006118522.1(1), YP_006118531.1(1), YP_006118538.1(1), YP_006118539.1(1), YP_006118541.1(1), YP_006118552.1(1), YP_006118553.1(1), YP_006118557.1(1), YP_006118561.1(1), YP_006118564.1(1), YP_006118569.1(1), YP_006118578.1(1), YP_006118579.1(1), YP_006118618.1(1), YP_006118620.1(1), YP_006118621.1(1), YP_006118627.1(1), YP_006118643.1(1), YP_006118677.1(1), YP_006118708.1(1), YP_006118743.1(1), YP_006118744.1(1), YP_006118752.1(1), YP_006118754.1(1), YP_006118761.1(1), YP_006118775.1(1), YP_006118784.1(1), YP_006118787.1(1), YP_006118788.1(1), YP_006118812.1(1), YP_006118821.1(1), YP_006118833.1(1), YP_006118838.1(1), YP_006118863.1(1), YP_006118889.1(1), YP_006118891.1(1), YP_006118898.1(1), YP_006118932.1(1), YP_006118950.1(1), YP_006118953.1(1), YP_006118979.1(1), YP_006119003.1(1), YP_006119038.1(1), YP_006119043.1(1), YP_006119049.1(1), YP_006119058.1(1), YP_006119073.1(1), YP_006119076.1(1), YP_006119077.1(1), YP_006119088.1(1), YP_006119100.1(1), YP_006119108.1(1), YP_006119133.1(1), YP_006119137.1(1), YP_006119142.1(1), YP_006119158.1(1), YP_006119159.1(1), YP_006119170.1(1), YP_006119171.1(1), YP_006119177.1(1), YP_006119192.1(1), YP_006119195.1(1), YP_006119198.1(1), YP_006119202.1(1), YP_006119211.1(1), YP_006119214.1(1), YP_006119222.1(1), YP_006119223.1(1), YP_006119255.1(1), YP_006119256.1(1), YP_006119340.1(1), YP_006119369.1(1), YP_006119407.1(1), YP_006119409.1(1), YP_006119412.1(1), YP_006119426.1(1), YP_006119437.1(1), YP_006119440.1(1), YP_006119449.1(1), YP_006119456.1(1), YP_006119458.1(1), YP_006119471.1(1), YP_006119541.1(1), YP_006119585.1(1), YP_006119586.1(1), YP_006119594.1(1), YP_006119595.1(1), YP_006119601.1(1), YP_006119602.1(1), YP_006119607.1(1), YP_006119627.1(1), YP_006119641.1(1), YP_006119642.1(1), YP_006119662.1(1), YP_006119664.1(1), YP_006119674.1(1), YP_006119685.1(1), YP_006119693.1(1), YP_006119694.1(1), YP_006119715.1(1), YP_006119739.1(1), YP_006119740.1(1), YP_006119741.1(1), YP_006119760.1(1), YP_006119783.1(1), YP_006119916.1(1), YP_006119921.1(1), YP_006119922.1(1), YP_006119926.1(1), YP_006119999.1(1), YP_006120014.1(1), YP_006120043.1(1), YP_006120071.1(1), YP_006120075.1(1), YP_006120076.1(1), YP_006120078.1(1), YP_006120079.1(1), YP_006120080.1(1), YP_006120081.1(1), YP_006120117.1(1), YP_006120147.1(1), YP_006120161.1(1), YP_006120165.1(1), YP_006120166.1(1), YP_006120180.1(1), YP_006120190.1(1), YP_006120194.1(1), YP_006120204.1(1), YP_006120219.1(1), YP_006120227.1(1), YP_006120237.1(1), YP_006120244.1(1), YP_006120256.1(1), YP_006120270.1(1), YP_006120295.1(1), YP_006120330.1(1), YP_006120331.1(1), YP_006120351.1(1), YP_006120402.1(1), YP_006120408.1(1), YP_006120413.1(1), YP_006120414.1(1), YP_006120495.1(1), YP_006120509.1(1), YP_006120519.1(1), YP_006120529.1(1), YP_006120531.1(1), YP_006120561.1(1), YP_006120564.1(1), YP_006120565.1(1), YP_006120566.1(1), YP_006120583.1(1), YP_006120594.1(1), YP_006120637.1(1), YP_006120651.1(1), YP_006120686.1(1), YP_006120698.1(1), YP_006120710.1(1), YP_006120759.1(1), YP_006120778.1(1), YP_006120820.1(1), YP_006120834.1(1), YP_006120842.1(1), YP_006120844.1(1), YP_006120849.1(1), YP_006120852.1(1), YP_006120870.1(1), YP_006120873.1(1), YP_006120879.1(1), YP_006120891.1(1), YP_006120898.1(1), YP_006120899.1(1), YP_006120901.1(1), YP_006120908.1(1), YP_006120909.1(1), YP_006120914.1(1), YP_006120929.1(1), YP_006120939.1(1), YP_006120940.1(1), YP_006120941.1(1), YP_006120942.1(1), YP_006120943.1(1), YP_006120950.1(1), YP_006120952.1(1), YP_006120973.1(1), YP_006120985.1(1), YP_006120986.1(1), YP_006121001.1(1), YP_006121024.1(1), YP_006121036.1(1), YP_006121038.1(1), YP_006121044.1(1), YP_006121047.1(1), YP_006121070.1(1), YP_006121071.1(1), YP_006121073.1(1), YP_006121079.1(1), YP_006121095.1(1), YP_006121128.1(1), YP_006121131.1(1), YP_006121184.1(1), YP_006121185.1(1), YP_006121186.1(1), YP_006121194.1(1), YP_006121221.1(1), YP_006121237.1(1), YP_006121238.1(1), YP_006121273.1(1), YP_006121359.1(1), YP_006121388.1(1), YP_006121397.1(1), YP_006121398.1(1), YP_006121399.1(1), YP_006121405.1(1), YP_006121440.1(1), YP_006121482.1(1), YP_006121484.1(1), YP_006121485.1(1), YP_006121486.1(1), YP_006121488.1(1), YP_006121489.1(1), YP_006121493.1(1), YP_006121497.1(1), YP_006121498.1(1), YP_006121501.1(1), YP_006121503.1(1), YP_006121504.1(1), YP_006121513.1(1), YP_006121519.1(1), YP_006121520.1(1), YP_006121541.1(1), YP_006121542.1(1), YP_006121557.1(1), YP_006121570.1(1), YP_006121578.1(1), YP_006121590.1(1), YP_006121594.1(1), YP_006121599.1(1), YP_006121600.1(1), YP_006121606.1(1), YP_006121607.1(1), YP_006121608.1(1), YP_006121609.1(1), YP_006121610.1(1), YP_006121611.1(1), YP_006121612.1(1), YP_006121613.1(1), YP_006121614.1(1), YP_006121615.1(1), YP_006121616.1(1), YP_006121617.1(1), YP_006121618.1(1), YP_006121619.1(1), YP_006121620.1(1), YP_006121621.1(1), YP_006121622.1(1), YP_006121623.1(1), YP_006121624.1(1), YP_006121625.1(1), YP_006121626.1(1), YP_006121627.1(1), YP_006121628.1(1), YP_006121629.1(1), YP_006121630.1(1), YP_006121631.1(1), YP_006121632.1(1), YP_006121633.1(1), YP_006121652.1(1), YP_006121653.1(1), YP_006121654.1(1), YP_006121655.1(1), YP_006121666.1(1), YP_006121693.1(1), YP_006121709.1(1), YP_006121716.1(1), YP_006121728.1(1), YP_006121729.1(1), YP_006121730.1(1), YP_006121764.1(1), YP_006121768.1(1), YP_006121769.1(1), YP_006121777.1(1), YP_006121778.1(1), YP_006121793.1(1), YP_006121794.1(1), YP_006121804.1(1), YP_006121814.1(1), YP_006121826.1(1), YP_006121859.1(1), YP_006121861.1(1), YP_006121862.1(1), YP_006121882.1(1), YP_006121883.1(1), YP_006121884.1(1), YP_006121885.1(1), YP_006121886.1(1), YP_006121894.1(1), YP_006121915.1(1), YP_006121916.1(1), YP_006121931.1(1), YP_006121957.1(1), YP_006121958.1(1), YP_006121959.1(1), YP_006121965.1(1), YP_006121971.1(1), YP_006121973.1(1), YP_006121974.1(1), YP_006121985.1(1), YP_006122031.1(1), YP_006122032.1(1), YP_006122035.1(1), YP_006122050.1(1), YP_006122052.1(1), YP_006122067.1(1), YP_006122068.1(1), YP_006122076.1(1), YP_006122096.1(1), YP_006122099.1(1), YP_006122130.1(1), YP_006122136.1(1), YP_006122172.1(1), YP_006122190.1(1), YP_006122194.1(1), YP_006122203.1(1), YP_006122220.1(1), YP_006122271.1(1), YP_006122272.1(1), YP_006122309.1(1), YP_006122315.1(1), YP_006122316.1(1), YP_006122318.1(1), YP_006122319.1(1), YP_006122320.1(1), YP_006122321.1(1), YP_006122322.1(1), YP_006122323.1(1), YP_006122330.1(1), YP_006122361.1(1), YP_006122376.1(1), YP_006122387.1(1), YP_006122405.1(1), YP_006122428.1(1), YP_006122429.1(1), YP_006122443.1(1), YP_006122452.1(1), YP_006122453.1(1), YP_006122461.1(1), YP_006122485.1(1), YP_006122534.1(1), YP_006122570.1(1), YP_006122574.1(1), YP_006122575.1(1), YP_006122577.1(1), YP_006122581.1(1), YP_006122582.1(1), YP_006122583.1(1), YP_006122589.1(1), YP_006122590.1(1), YP_006122611.1(1), YP_006122613.1(1), YP_006122614.1(1), YP_006122643.1(1), YP_006122658.1(1), YP_006122672.1(1), YP_006122724.1(1), YP_006122725.1(1), YP_006122734.1(1), YP_006122736.1(1), YP_006122737.1(1), YP_006122764.1(1), YP_006122784.1(1), YP_006122795.1(1), YP_006162190.1(1), YP_006162210.1(1), YP_006162211.1(1), YP_006162216.1(1), YP_006162264.1(1), YP_006162271.1(1), YP_006162272.1(1), YP_006162276.1(1), YP_006162277.1(1), YP_006162317.1(1), YP_006776768.1(1), YP_006776799.1(1), YP_006776852.1(1), YP_006776853.1(1), YP_006776855.1(1), YP_006776861.1(1), YP_006776867.1(1), YP_006776868.1(1), YP_006776869.1(1), YP_006776895.1(1), YP_006776914.1(1), YP_006776915.1(1), YP_006776939.1(1), YP_006776947.1(1), YP_006776948.1(1), YP_006776949.1(1), YP_006776950.1(1), YP_006776951.1(1), YP_006776969.1(1), YP_006776970.1(1), YP_006776972.1(1), YP_006776973.1(1), YP_006777017.1(1), YP_006777057.1(1), YP_006777061.1(1), YP_006777062.1(1), YP_006777077.1(1), YP_006777078.1(1), YP_006777087.1(1), YP_006777088.1(1), YP_006777093.1(1), YP_006777121.1(1), YP_006777122.1(1), YP_006777123.1(1), YP_006777135.1(1), YP_006777144.1(1), YP_006777160.1(1), YP_006777191.1(1), YP_006777202.1(1), YP_006777203.1(1), YP_006777204.1(1), YP_006777205.1(1), YP_006777209.1(1), YP_006777210.1(1), YP_006777211.1(1), YP_006777212.1(1), YP_006777213.1(1), YP_006777214.1(1), YP_006777215.1(1), YP_006777216.1(1), YP_006777217.1(1), YP_006777218.1(1), YP_006777219.1(1), YP_006777220.1(1), YP_006777221.1(1), YP_006777222.1(1), YP_006777223.1(1), YP_006777224.1(1), YP_006777225.1(1), YP_006777226.1(1), YP_006777227.1(1), YP_006777228.1(1), YP_006777229.1(1), YP_006777230.1(1), YP_006777231.1(1), YP_006777232.1(1), YP_006777233.1(1), YP_006777234.1(1), YP_006777235.1(1), YP_006777236.1(1), YP_006777242.1(1), YP_006777243.1(1), YP_006777248.1(1), YP_006777252.1(1), YP_006777263.1(1), YP_006777277.1(1), YP_006777292.1(1), YP_006777293.1(1), YP_006777316.1(1), YP_006777317.1(1), YP_006777323.1(1), YP_006777332.1(1), YP_006777333.1(1), YP_006777335.1(1), YP_006777338.1(1), YP_006777339.1(1), YP_006777343.1(1), YP_006777347.1(1), YP_006777348.1(1), YP_006777350.1(1), YP_006777351.1(1), YP_006777352.1(1), YP_006777354.1(1), YP_006777405.1(1), YP_006777447.1(1), YP_006777453.1(1), YP_006777454.1(1), YP_006777455.1(1), YP_006777464.1(1), YP_006777575.1(1), YP_006777636.1(1), YP_006777664.1(1), YP_006777665.1(1), YP_006777682.1(1), YP_006777713.1(1), YP_006777721.1(1), YP_006777722.1(1), YP_006777723.1(1), YP_006777799.1(1), YP_006777809.1(1), YP_006777829.1(1), YP_006777831.1(1), YP_006777832.1(1), YP_006777855.1(1), YP_006777858.1(1), YP_006777859.1(1), YP_006777860.1(1), YP_006777861.1(1), YP_006777862.1(1), YP_006777867.1(1), YP_006777873.1(1), YP_006777875.1(1), YP_006777889.1(1), YP_006777915.1(1), YP_006777931.1(1), YP_006777932.1(1), YP_006777945.1(1), YP_006777975.1(1), YP_006777977.1(1), YP_006777984.1(1), YP_006777985.1(1), YP_006777986.1(1), YP_006777987.1(1), YP_006777988.1(1), YP_006777998.1(1), YP_006778007.1(1), YP_006778013.1(1), YP_006778014.1(1), YP_006778021.1(1), YP_006778023.1(1), YP_006778024.1(1), YP_006778032.1(1), YP_006778044.1(1), YP_006778056.1(1), YP_006778059.1(1), YP_006778077.1(1), YP_006778080.1(1), YP_006778082.1(1), YP_006778084.1(1), YP_006778092.1(1), YP_006778120.1(1), YP_006778121.1(1), YP_006778123.1(1), YP_006778167.1(1), YP_006778186.1(1), YP_006778276.1(1), YP_006778288.1(1), YP_006778300.1(1), YP_006778334.1(1), YP_006778348.1(1), YP_006778383.1(1), YP_006778394.1(1), YP_006778408.1(1), YP_006778409.1(1), YP_006778410.1(1), YP_006778413.1(1), YP_006778445.1(1), YP_006778447.1(1), YP_006778454.1(1), YP_006778465.1(1), YP_006778480.1(1), YP_006778561.1(1), YP_006778562.1(1), YP_006778567.1(1), YP_006778575.1(1), YP_006778640.1(1), YP_006778641.1(1), YP_006778681.1(1), YP_006778704.1(1), YP_006778719.1(1), YP_006778732.1(1), YP_006778740.1(1), YP_006778771.1(1), YP_006778815.1(1), YP_006778823.1(1), YP_006778839.1(1), YP_006778849.1(1), YP_006778853.1(1), YP_006778862.1(1), YP_006778876.1(1), YP_006778877.1(1), YP_006778881.1(1), YP_006778900.1(1), YP_006778931.1(1), YP_006778969.1(1), YP_006778970.1(1), YP_006778971.1(1), YP_006778972.1(1), YP_006778973.1(1), YP_006778974.1(1), YP_006778976.1(1), YP_006779028.1(1), YP_006779056.1(1), YP_006779084.1(1), YP_006779085.1(1), YP_006779100.1(1), YP_006779175.1(1), YP_006779211.1(1), YP_006779267.1(1), YP_006779273.1(1), YP_006779293.1(1), YP_006779305.1(1), YP_006779306.1(1), YP_006779346.1(1), YP_006779366.1(1), YP_006779372.1(1), YP_006779406.1(1), YP_006779407.1(1), YP_006779409.1(1), YP_006779434.1(1), YP_006779461.1(1), YP_006779462.1(1), YP_006779466.1(1), YP_006779478.1(1), YP_006779488.1(1), YP_006779489.1(1), YP_006779544.1(1), YP_006779568.1(1), YP_006779569.1(1), YP_006779581.1(1), YP_006779594.1(1), YP_006779595.1(1), YP_006779596.1(1), YP_006779600.1(1), YP_006779601.1(1), YP_006779602.1(1), YP_006779608.1(1), YP_006779609.1(1), YP_006779656.1(1), YP_006779658.1(1), YP_006779665.1(1), YP_006779674.1(1), YP_006779677.1(1), YP_006779688.1(1), YP_006779702.1(1), YP_006779705.1(1), YP_006779707.1(1), YP_006779745.1(1), YP_006779785.1(1), YP_006779809.1(1), YP_006779814.1(1), YP_006779836.1(1), YP_006779861.1(1), YP_006779874.1(1), YP_006779908.1(1), YP_006779933.1(1), YP_006779954.1(1), YP_006779955.1(1), YP_006779993.1(1), YP_006779994.1(1), YP_006780009.1(1), YP_006780012.1(1), YP_006780023.1(1), YP_006780027.1(1), YP_006780030.1(1), YP_006780033.1(1), YP_006780051.1(1), YP_006780057.1(1), YP_006780058.1(1), YP_006780063.1(1), YP_006780064.1(1), YP_006780079.1(1), YP_006780084.1(1), YP_006780088.1(1), YP_006780114.1(1), YP_006780124.1(1), YP_006780135.1(1), YP_006780145.1(1), YP_006780147.1(1), YP_006780150.1(1), YP_006780166.1(1), YP_006780202.1(1), YP_006780237.1(1), YP_006780243.1(1), YP_006780248.1(1), YP_006780290.1(1), YP_006780322.1(1), YP_006780338.1(1), YP_006780348.1(1), YP_006780367.1(1), YP_006780402.1(1), YP_006780410.1(1), YP_006780412.1(1), YP_006780445.1(1), YP_006780474.1(1), YP_006780479.1(1), YP_006780488.1(1), YP_006780495.1(1), YP_006780520.1(1), YP_006780521.1(1), YP_006780524.1(1), YP_006780533.1(1), YP_006780549.1(1), YP_006780556.1(1), YP_006780558.1(1), YP_006780566.1(1), YP_006780567.1(1), YP_006780601.1(1), YP_006780640.1(1), YP_006780642.1(1), YP_006780647.1(1), YP_006780668.1(1), YP_006780692.1(1), YP_006780717.1(1), YP_006780723.1(1), YP_006780724.1(1), YP_006780766.1(1), YP_006780767.1(1), YP_006780776.1(1), YP_006780781.1(1), YP_006780786.1(1), YP_006780790.1(1), YP_006780794.1(1), YP_006780795.1(1), YP_006780806.1(1), YP_006780808.1(1), YP_006780809.1(1), YP_006780816.1(1), YP_006780825.1(1), YP_006780829.1(1), YP_006780830.1(1), YP_006780833.1(1), YP_006780834.1(1), YP_006780850.1(1), YP_006780859.1(1), YP_006780881.1(1), YP_006780902.1(1), YP_006780906.1(1), YP_006780909.1(1), YP_006780916.1(1), YP_006780917.1(1), YP_006780922.1(1), YP_006780948.1(1), YP_006780951.1(1), YP_006780957.1(1), YP_006780971.1(1), YP_006780982.1(1), YP_006780999.1(1), YP_006781022.1(1), YP_006781023.1(1), YP_006781025.1(1), YP_006781036.1(1), YP_006781070.1(1), YP_006781072.1(1), YP_006781127.1(1), YP_006781136.1(1), YP_006781140.1(1), YP_006781155.1(1), YP_006781158.1(1), YP_006781159.1(1), YP_006781180.1(1), YP_006781181.1(1), YP_006781183.1(1), YP_006781205.1(1), YP_006781206.1(1), YP_006781212.1(1), YP_006781213.1(1), YP_006781214.1(1), YP_006781218.1(1), YP_006781220.1(1), YP_006781221.1(1), YP_006781227.1(1), YP_006781263.1(1), YP_006781280.1(1), YP_006781294.1(1), YP_006781318.1(1), YP_006781326.1(1), YP_006781327.1(1), YP_006781361.1(1), YP_006781371.1(1), YP_006781386.1(1), YP_006781403.1(1), YP_006781425.1(1), YP_006781433.1(1), YP_006781434.1(1), YP_006781435.1(1), YP_006781436.1(1), YP_006781437.1(1), YP_006781438.1(1), YP_006781440.1(1), YP_006781441.1(1), YP_006781447.1(1), YP_006781483.1(1), YP_006781484.1(1), YP_006781537.1(1), YP_006781551.1(1), YP_006781560.1(1), YP_006781564.1(1), YP_006781582.1(1), YP_006781595.1(1), YP_006781600.1(1), YP_006781603.1(1), YP_006781637.1(1), YP_006781640.1(1), YP_006781661.1(1), YP_006781669.1(1), YP_006781670.1(1), YP_006781689.1(1), YP_006781706.1(1), YP_006781707.1(1), YP_006781710.1(1), YP_006781711.1(1), YP_006781751.1(1), YP_006781795.1(1), YP_006792546.1(1), YP_006792580.1(1), YP_006792581.1(1), YP_006792601.1(1), YP_006792618.1(1), YP_025292.1(1), YP_025295.1(1), YP_025297.1(1), YP_025298.1(1), YP_025299.1(1), YP_025300.1(1), YP_025301.1(1), YP_026161.1(1), YP_026219.1(1), YP_026225.1(1), YP_026227.1(1), YP_026229.1(1), YP_026263.3(1), YP_026274.1(1), YP_026282.1(1), YP_026286.1(1), YP_588437.1(1), YP_588451.1(1)
